# Data of synthesis, characterization and luminescence measurements in 1D lanthanide coordination polymers based on lanthanides

**DOI:** 10.1016/j.dib.2019.104709

**Published:** 2019-10-22

**Authors:** R.F. D'Vries, G.E. Gomez, L.P. Mondragon, D. Onna, B.C. Barja, G.J.A.A. Soler-Illia, J. Ellena

**Affiliations:** a Universidad Santiago de Cali, Calle 5 # 62-00, Cali, Colombia; b Centro Atómico Constituyentes, Comisión Nacional de Energía Atómica (CAC-CNEA), Av. Gral. Paz 1499, 1650 San Martín, Buenos Aires, Argentina; c Instituto de Nanosistemas, Universidad Nacional de San Martín (INS-UNSAM), Av. 25 de Mayo 1021, San Martín, Buenos Aires, Argentina; d Instituto de Química, Física de los Materiales, Medioambiente y Energía (INQUIMAE-CONICET), DQIAQF, Facultad de Ciencias Exactas y Naturales, Universidad de Buenos Aires, Pabellón II, Ciudad Universitaria, C1428EHA, Buenos Aires, Argentina; e Instituto de Física de São Carlos, Universidade de São Paulo, USP, São Carlos, SP, Brazil

**Keywords:** Coordination polymers, Supramolecular topology, Lanthanide metals, Luminescence

## Abstract

In this work are presented all the conditions of synthesis explored to obtain a new family of compound with formula [Ln(4-OHBBA)3(H2O)2] (Ln = La, Pr). Powder X ray diffraction was used to identify the different phases obtained in the synthetic study. FT-IR spectroscopy and TG analysis for La and Pr pure phases are also reported. Optical properties of optically active CPs materials, solid state photoluminescence properties of La, Pr, La-(5%Eu) and La-(5%Tb) compounds were explored. We report the absorption, excitation and emission spectrum of the 4′-hydroxi-4-biphenylcarboxylic acid and a comparative description of the radiative (and no-radiative) processes in solid state in Ln-(4-OHBBA) and Ln-BPDC compounds. Finally, a principal component analysis was conducted in order to take in account both signal contributions from the sensor (LCE at 384 nm and the europium emission at 610 nm) and for classifying the type of analytes used to test the sensing response of the materials.

**Table:** Specifications Table

Subject area	*chemistry*
More specific subject area	*Inorganic Chemistry*
Type of data	*Synthesis conditions table; Powder X-ray diffraction patterns; Infrared spectra; TGA analysis; absorption, excitation and emission spectra, PCA graphic.*
How data was acquired	*Fourier Transform Infrared spectra were recorded from KBr pellets Bomem Michelson FT MB-102.* *X-ray powder diffraction (PXRD) patterns were obtained with a Rigaku Ultima IV.* *The emission spectra were recorded on a PTI QuantaMaster QM-1 luminescence spectrometer* *Thermogravimetric analysis (TGA) was performed using Shimadzu TGA-50 equipment*
Data format	*Raw, Filtered and analyzed.*
Experimental factors	*All the measures were made in solid state.*
Experimental features	*Experiment for the characterization of new crystalline materials*
Data source location	*Cali, Colombia, Universidad Santiago de Cali.* *Sao Carlos, Brasil, Instituto de Física de Sao Carlos.* *Buenos Aires, Argentina, CNEA.*
Data accessibility	*Data is with this article and supporting information*
Related research article	*R. F. D'Vries, G. E. Gomez, L. P. Mondragon, D. Onna, B. C. Barja, G. J. A. A. Soler-Illia, J. Ellena.1D lanthanide coordination polymers based on lanthanides and 4′-hydroxi-4-biphenylcarboxylic acid: synthesis, structures and luminescence properties. J. Sol. Stat. Chem.* 2019*, 274, 322–328.*

**Table:** 

**Value of the Data** • The data provide the synthesis condition explored for obtain pure phases and doped phases of coordination polymers. • In this study is showed characterization of the materials by powder X-ray diffraction patterns, FT-IR spectroscopy and thermogravimetric analysis. • In this study is presented a comparison of the radiative (and no-radiative) processes in solid state in Ln-(4-OHBBA) and Ln-BPDC compounds. • The data and results of principal component analysis (PCA) are present in order to take in account both signal contributions and for classifying the type of analytes used to test the sensing response of the materials.

## 1 Data

**Fig. 1:**
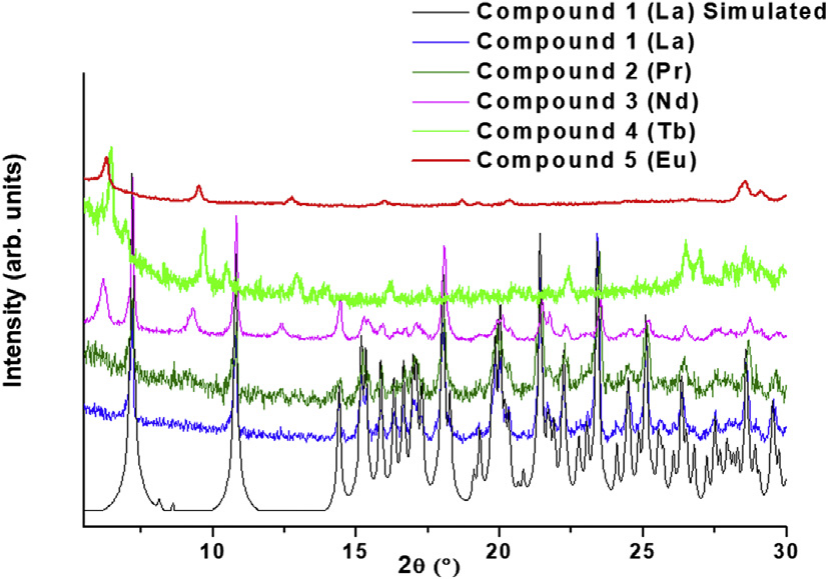
Powder X-ray diffraction of the synthesis of compounds from La, Pr, Nd, Eu and Tb.

**Fig. 2:**
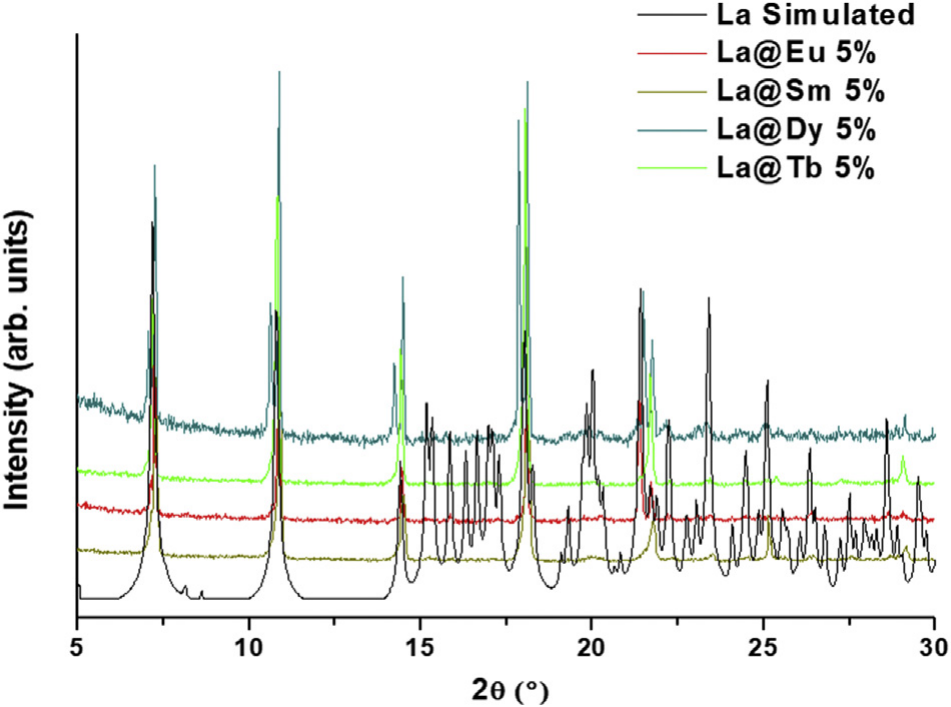
Powder X-ray diffraction of the doped compound [La_0.95_Ln_0.05_(4-OHBBA)_3_(H_2_O)_2_] (Ln = Sm, Eu, Tb and Dy).

**Fig. 3:**
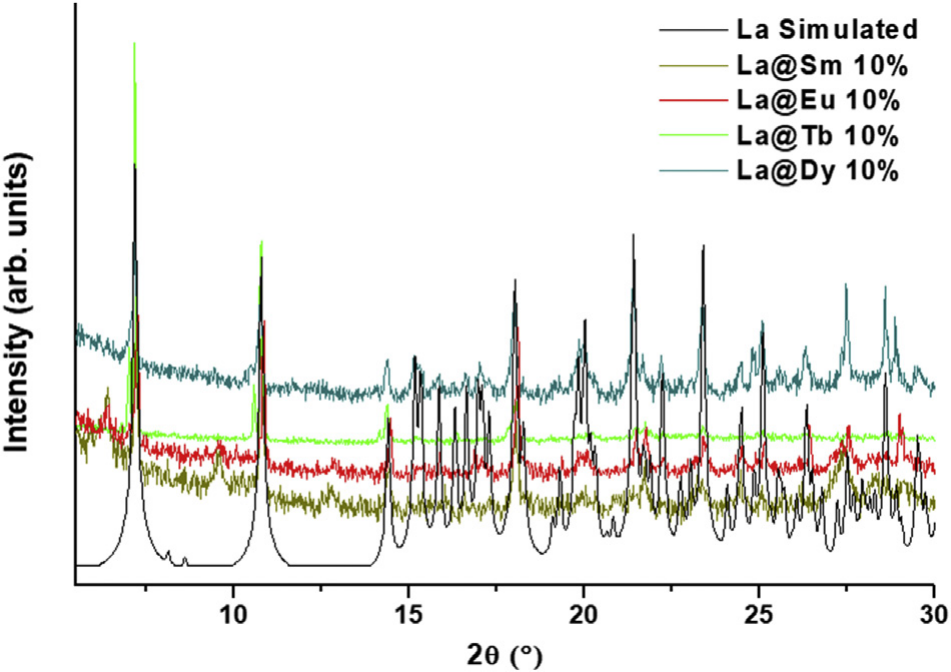
Powder X-ray diffraction of 10% doped compound.

**Fig. 4:**
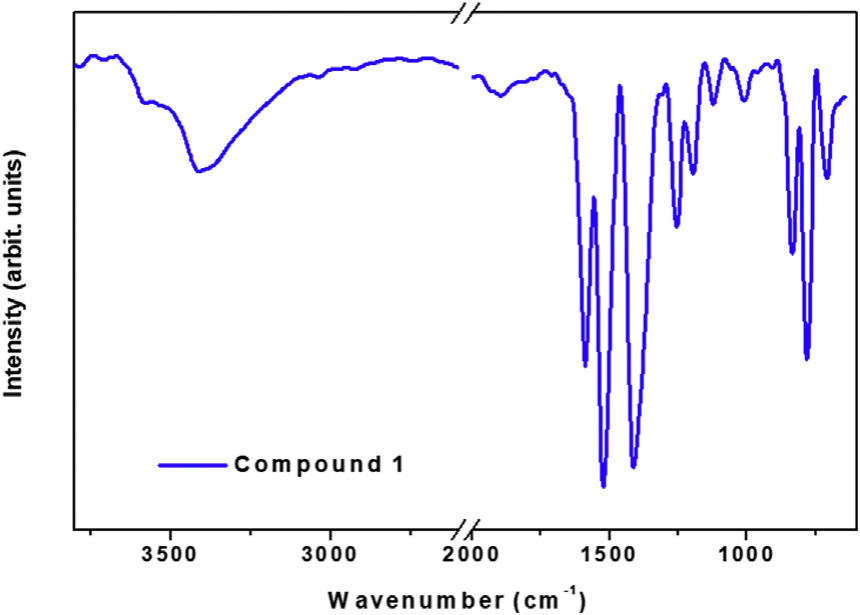
Infrared spectrum for [La(4-OHBBA)_3_(H_2_O)_2_] compound.

**Fig. 5:**
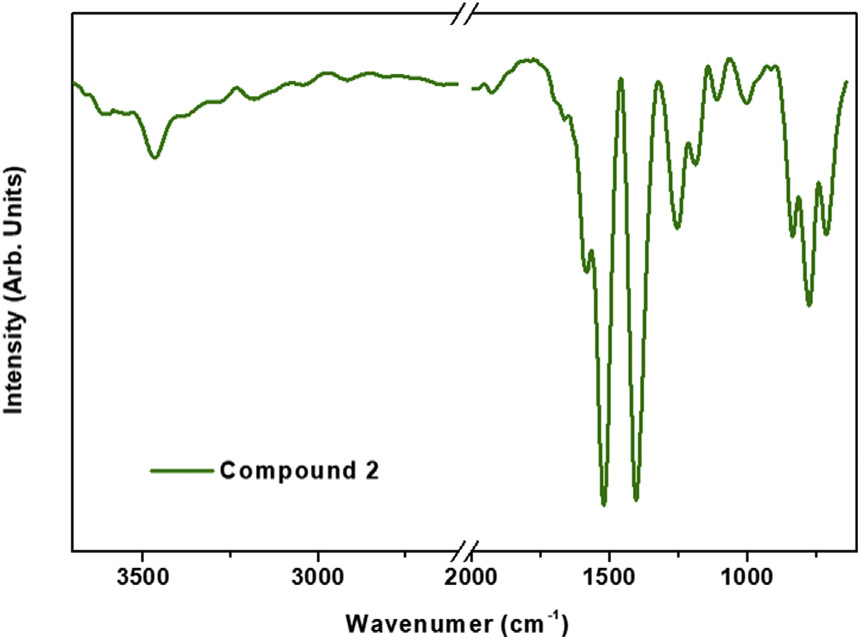
Infrared spectrum for [Pr(4-OHBBA)_3_(H_2_O)_2_] compound.

**Fig. 6:**
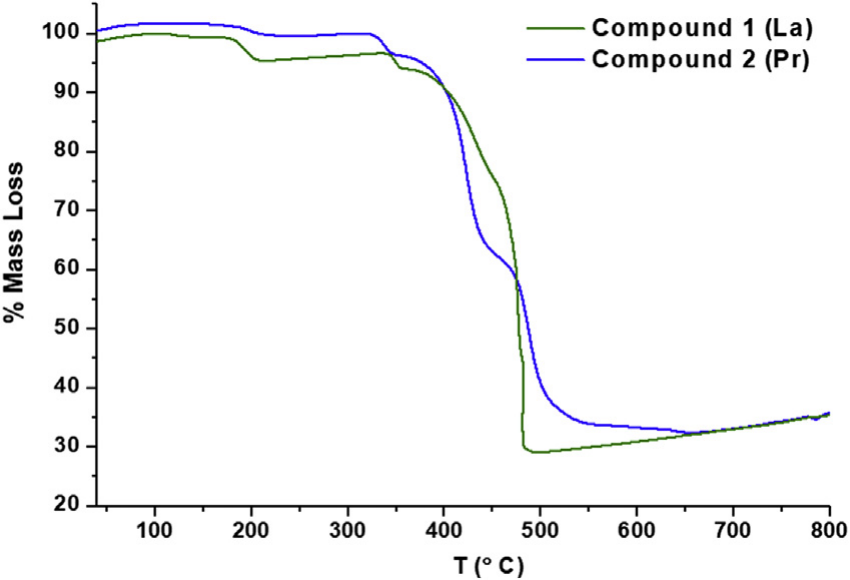
Thermal profile for [Ln(4-OHBBA)_3_(H_2_O)_2_] (Ln = La and Pr) compounds.

This data contains the different syntheses carried out to obtain pure phases of all the series of lanthanide metals and the doped samples (Table 1) [1]. Pure phases of the [Ln(4-OHBBA)_3_(H_2_O)_2_] (phase 1) were obtained for the La and Pr metals. The synthesis of Nd, Tb and Eu compounds results in the appearance of an unidentified phase (Fig. 1, Supp. Inf. Table S1). In the doped samples, the addition of Eu, Tb and Dy metals until 5% does not affect the structure and the phase 1 is obtained (Fig. 2, Supp. Inf. Table S2). This behavior is previously reported by our group [2]. To doped concentration values above 5%, mixture phases are observed (Fig. 3, Supp. Inf. Table S3) [1]. Vibrational and thermal analysis were realized for the compounds 1 and 2 in order to observe the presence of the most important functional groups in the ligand (Fig. 4, Fig. 5, Supp. Inf. Table S4-S5) and the thermal behaviour of the compounds (See Fig. 6).

**Table 1:** Reaction condition evaluated to obtain the [Ln(4-OHBBA)_3_(H_2_O)_2_] (Ln = La and Pr) as pure phases.

METAL	Molar ratio Metal:Ligand	Solvent (10 mL)	Base	Temperature and Time	Product
La	1:1	Water	NaOH 1.0 M	170°C, 24h	Compound 1 (powder)
La	1:1	Water/Ethanol	NaOH 1.0 M	170°C, 24h	Phase 1
Pr	1:1	Water/Ethanol	NaOH 1.0 M	170°C, 24h	Phase 1
Nd	1:1	Water/Ethanol	NaOH 1.0 M	170°C, 24h	Powder (phases mixture)
Sm	1:1	Water/Ethanol	NaOH 1.0 M	170°C, 24h	Powder (phases mixture)
Eu	1:1	Water/Ethanol	NaOH 1,0 M	170°C, 24h	Powder (unknown phase)
Tb	1:1	Water/Ethanol	NaOH 1.0 M	170°C, 24h	Powder (phases mixture)
La	1:1	Water/Ethanol	NaOH 1.0 M	170°C, 96h	Phase 1
La	1:3	Water/Ethanol	NaOH 1.0 M	170°C, 24h	Phase 1 (powder)
La	1:1	Water/Toluene	NaOH 1.0 M	170°C, 24h	Phase 1 (powder)
La	1:1	Water/DMF	–	170°C, 24h	Phase 1 (powder)
La	1:1	Water/Ethanol	Pyridine	170°C, 72h	Compound 1 (powder)
La	1:1	Water/Ethanol	Triethanolamine	110°C, 48h	Phase 1 (powder)
La/Eu 5%	1:1	Water/Ethanol	NaOH 1.0 M	170°C, 24h	Phase 1
La/Eu 10%	1:1	Water/Ethanol	NaOH 1.0 M	170°C, 24h	Phases mixture
La/Tb 5%	1:1	Water/Ethanol	NaOH 1.0 M	170°C, 24h	Phase 1
La/Tb 10%	1:1	Water/Ethanol	NaOH 1.0 M	170°C, 24h	Phases mixture

**Fig. 7:**
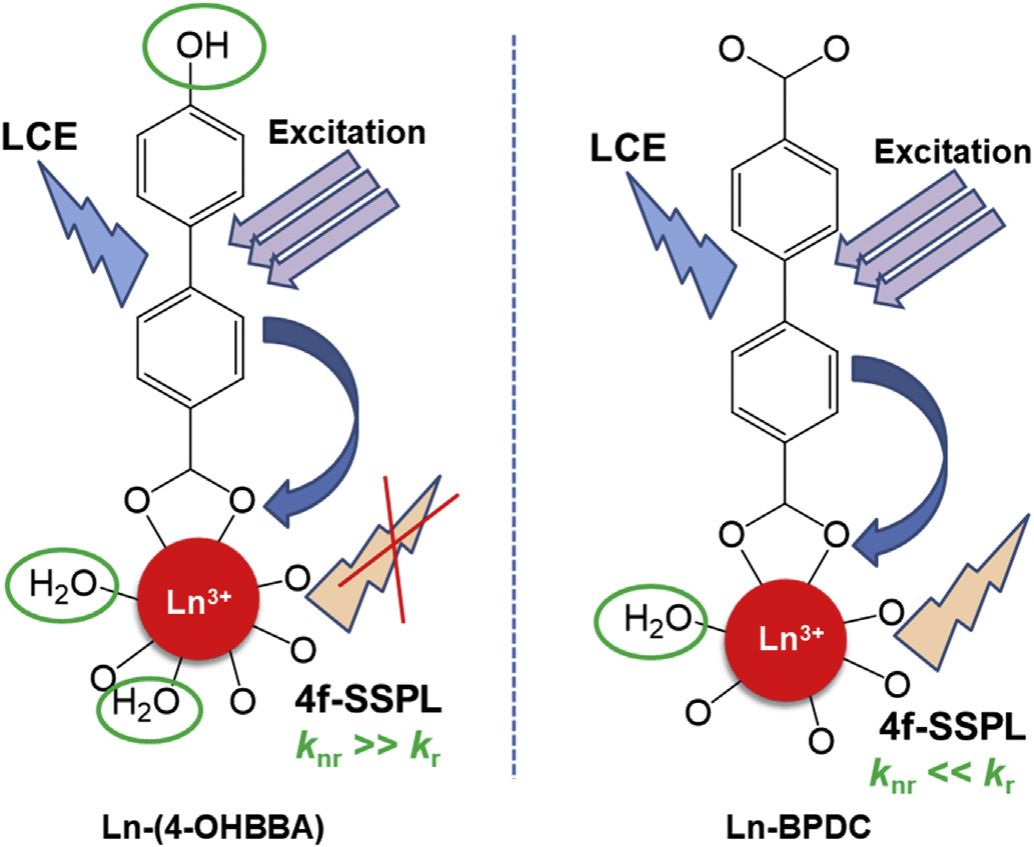
Description of the radiative (and no-radiative) processes in solid state in Ln-(4-OHBBA) and Ln-BPDC compounds.

**Fig. 8:**
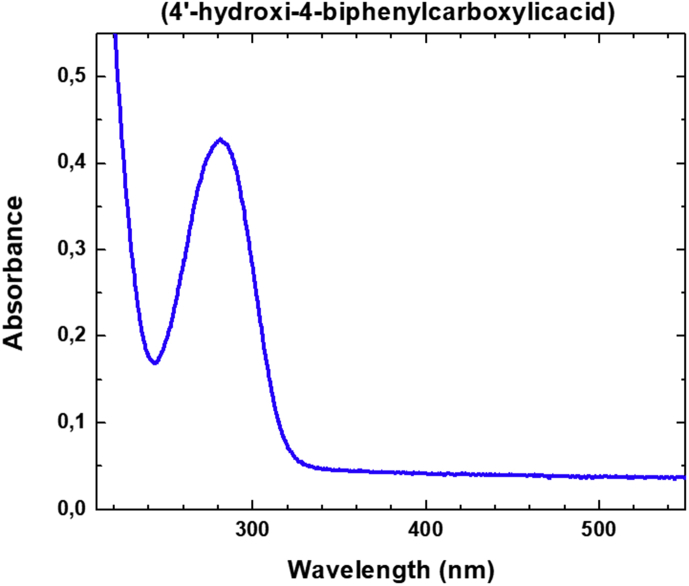
Absorption spectrum of 4′-hydroxi-4-biphenylcarboxylic acid dissolved in ethanol.

**Fig. 9:**
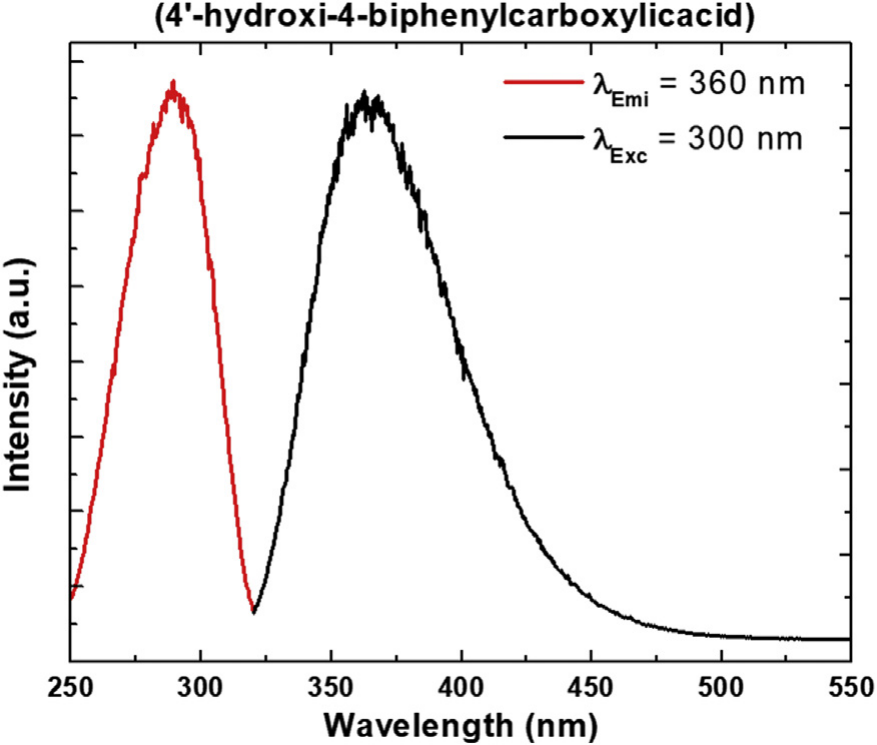
Excitation and emission spectra of 4′-hydroxi-4-biphenylcarboxylic acid dissolved in ethanol.

**Fig. 10:**
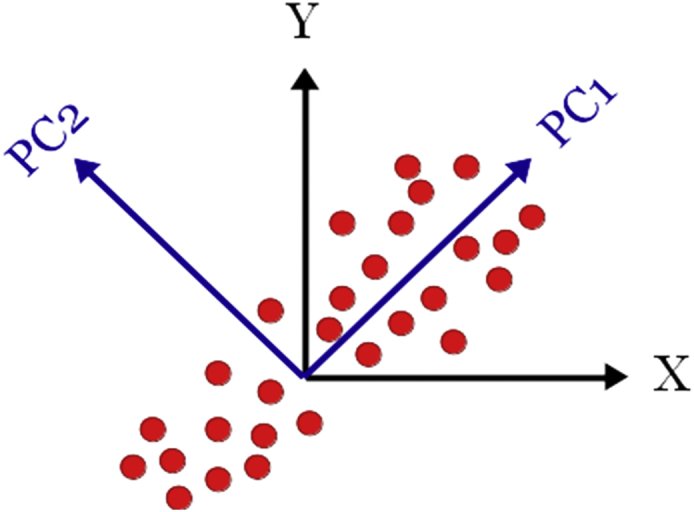
Graphical representation of the PCA method for a 2 dimensional space.

Some structural similarities can be assessed with the compounds reported by Guo et al. [3]. In this work, the authors reported a set of Ln-MOFs based on 4,4′-biphenyldicarboxylic acid in which the aromatic linker showed both ligand and lanthanide emissions, being the former the higher in intensity. Compared to compounds **1** and **2**, the lanthanide signals in Ln-BPDC are not quenched, being their lower number of coordination water and the absence of terminal OH ligands the main reasons for the decrease in non-radiative deactivation (Fig. 7). Electronic transition spectroscopy was realized for the 4-OHBBA ligand. Absortion, excitation and emission spectra were obtained for the ligand. The linker exhibits a wide blue emission band in solid state which is located at 384 nm under excitation with λ_exc_ = 300 nm, ascribed to the typical π *→ π/π*→n transitions of aromatic ligands (Fig. 8, Fig. 9, Supp. Inf. Table S7-S8). The Fig. 10, shows a PCA method for the case of a data set in 2 dimensional space, where the first principal component (PC1) is the coordinate that best preserves the relative distances between the samples and this component has maximum variance of the scores. The second principal component (PC2) is an orthogonal coordinate to PC1 and again possessing the maximum possible variance of the scores.

**Fig. 11:**
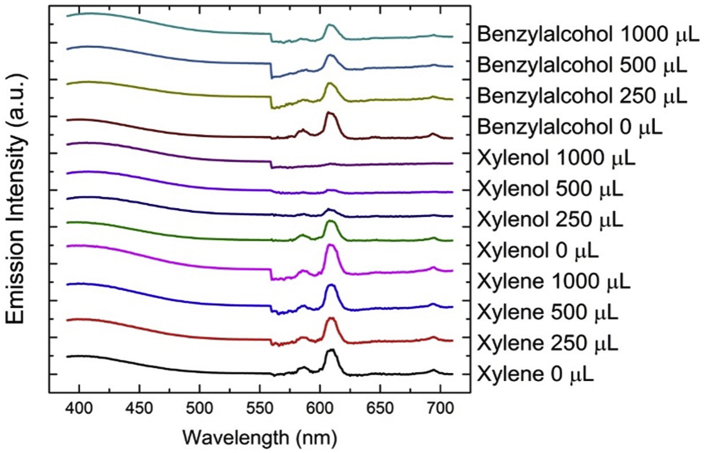
Emission spectra corrected used in PCA.

**Fig. 12:**
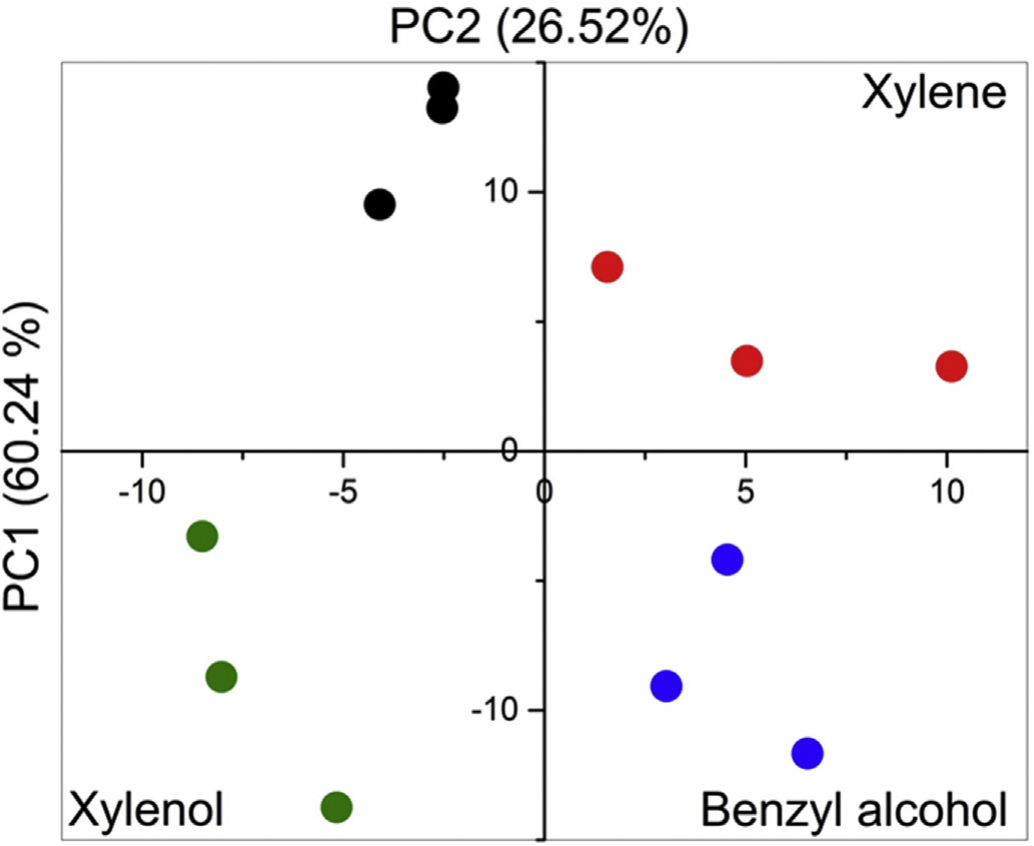
PCA score plot of the emission response of the 1-(5%Eu) (black dots) and the 1 (5%Eu) exposed to xylene (red dots), xylenol (green dots) and benzyl alcohol (blue dots).

The emission spectra were pre-processed, as detailed in the Fig. 11, before applying the PCA routine. The two most relevant components that PCA provided explain 86.76% of the variation. The scores plot for these two components, PC1 versus PC2, is shown in Fig. 12 (Supp. Inf. Table S9). Raw data can be found in the attached supplementary data. PCA analysis revealed a clear discrimination of the analytes between the three groups, which is a function of the principal component variation in the samples, based on their chemical structure. The three groups are: (i) the xylene with only methyl group, (ii) the xylenol with methyl and hydroxyl groups and, (iii) the benzyl alcohol with hydroxymethyl group. These results give promising outlooks in the uses of CPs as sensors for explosives precursors. Raw data can be found in the attached supplementary data.

## 2 Experimental design, materials, and methods

The sample preparation, PXRD, FT-IR, TGA and luminescence analysis methodologies for the data presented here have been previously described and cited [1].

All reagents and solvents employed were commercially available: 4′-hydroxi-4-biphenylcarboxylic acid (4-OHBBA) (99%, Sigma-Aldrich); Ln(NO_3_)_3_·6H_2_O where Ln = La and Pr, (99%, Sigma-Aldrich).

### 2.1 Experimental assembly

[Ln(4-OHBBA)_3_(H_2_O)_2_] compounds were obtained by the addition of 4-OHBBA (0.025 g, 0.115 mmol) in 5 mL of ethanol, into a solution of Ln(NO_3_)_3_·6H_2_O (0.115 mmol) in 5 mL of distilled water. The reaction mixture was adjusted to pH ≈ 6 by the addition of NaOH 1 M, under constant stirring at room temperature for 30 minutes. The reaction mixture was then placed in a Parr Teflon-lined stainless-steel autoclave at 160 °C for 17 hours.

### 2.2 Characterization

Thermogravimetric analysis (TGA) was performed using Shimadzu TGA-50 equipment at 25–900 °C temperature range, under nitrogen atmosphere (100 mL/min flow) and 10 °C min^−1^ heating rate. Fourier Transform Infrared (FT-IR) spectra were recorded from KBr pellets in the 4000-250 cm^−1^ range on a Bomem Michelson FT MB-102. X-ray powder diffraction (PXRD) patterns were obtained with a Rigaku Ultima IV diffractometer of 0.02° step size and 2 second/step exposure time. Single-crystal X-ray data for both compounds were collected at room temperature (298 K) on a Bruker APEX-II CCD diffractometer using MoKα radiation (0.71073 Å). The emission spectra were recorded on a PTI QuantaMaster QM-1 luminescence spectrometer with a 75 W Xenon lamp as excitation source.

### 2.3 Principal component analysis

The aim of principal component analysis (PCA) [4] is a dimension reduction by generating a new coordinate system formed by the components, which is orthogonal, and where only the most informative dimensions are used. A component is a combination of the variables, in our case emission intensities, and the value of a component is called score. These components ideally represent the distances between the samples in the multi-variable space.

The first principal component (PC1) is the coordinate that best preserves the relative distances between the samples and this component has maximum variance of the scores. The second principal component (PC2) is an orthogonal coordinate to PC1 and again possessing the maximum possible variance of the scores. In general, for higher numbers of principal components the variance becomes small or zero. So, the first components containing the main amount of variance. The Fig. 10 shows a PCA method for the case of a data set in 2 dimensional space. Principal component analysis (PCA) was calculated with MATLAB.
