# Supplementary material for: Data of synthesis, characterization and luminescence measurements in 1D lanthanide coordination polymers based on lanthanides
Source: Data Brief. 2019 Oct 22;27:104709. doi: 10.1016/j.dib.2019.104709 (PMC6849083; doi:10.1016/j.dib.2019.104709)
Supplement: Multimedia component 1 [file mmc1.docx]

*Data of synthesis, characterization and luminescence measurements in 1D lanthanide coordination polymers based on lanthanides*

*R. F. D'Vries,^a^* G. E. Gomez, ^b,c^* L. P. Mondragon,^a^ D. Onna,^c,d^ B. C. Barja,^d^ G. J. A. A. Soler-Illia,^c^ J. Ellena.^e^*

*^a^Universidad Santiago de Cali, Calle 5 # 62-00, Cali, Colombia. richard.dvries00@usc.edu.co*

*^b^Centro Atómico Constituyentes, Comisión Nacional de Energía Atómica (CAC-CNEA), Av. Gral. Paz 1499, 1650 San Martín, Buenos Aires, Argentina. gegomez@unsl.edu.ar*

*^c^Instituto de Nanosistemas. Universidad Nacional de San Martín (INS-UNSAM), Av. 25 de Mayo 1021, San Martín, Buenos Aires, Argentina.Departamento de Física, Universidade Federal do Ceará, CP 6030, 60440-900, Fortaleza, CE, Brazil.*

*^d^Instituto de Química, Física de los Materiales, Medioambiente y Energía (INQUIMAE-CONICET), DQIAQF, Facultad de Ciencias Exactas y Naturales, Universidad de Buenos Aires, Pabellón II, Ciudad Universitaria, C1428EHA-Buenos Aires, Argentina.*

*^e^Instituto de Física de São Carlos, Universidade de São Paulo, USP, São Carlos, SP, Brazil.*

Email: [*richard.dvries00@usc.edu.co*](mailto:richard.dvries00@usc.edu.co)

**Supplementary data**

Table S1 – S3 show the raw data of the DRX from Figures 1 - 3.

Table S4 – S5 show the raw data of the FT-IR from Figures 4 - 5.

Table S6 show the raw data of the TGA-DSC from Figure 6.

Table S7 show the raw data of the absorption spectrum from Figure 8.

Table S8 show the raw data of the excitation and emission spectra from Figure 9.

Table S9 show the raw data of the PCA score plot from Figure 12.

Table S1 – S3 show the raw data of the DRX from Figure 1 - 3.

| La (1) | | Pr(2) | | Nd(3) | | Tb(4) | | Eu(5) | |
| --- | --- | --- | --- | --- | --- | --- | --- | --- | --- |
| 2θ | I (A.U) | 2θ | I (A.U) | 2θ | I (A.U) | 2θ | I (A.U) | 2θ | I (A.U) |

3 494 3 527 3 1152 3 587 3 18460

3,02 518 3,02 488 3,02 1259 3,02 618 3,02 18048

3,04 446 3,04 528 3,04 1223 3,04 621 3,04 17578

3,06 492 3,06 464 3,06 1298 3,06 667 3,06 17233

3,08 484 3,08 461 3,08 1357 3,08 664 3,08 16698

3,1 465 3,1 480 3,1 1433 3,1 719 3,1 16228

3,12 504 3,12 525 3,12 1344 3,12 720 3,12 14927

3,14 481 3,14 467 3,14 1301 3,14 707 3,14 13999

3,16 519 3,16 482 3,16 1207 3,16 752 3,16 12545

3,18 498 3,18 453 3,18 1103 3,18 834 3,18 11023

3,2 478 3,2 499 3,2 1085 3,2 813 3,2 10147

3,22 498 3,22 469 3,22 938 3,22 887 3,22 9283

3,24 541 3,24 468 3,24 876 3,24 996 3,24 8291

3,26 505 3,26 436 3,26 818 3,26 1006 3,26 7622

3,28 543 3,28 471 3,28 851 3,28 835 3,28 7059

3,3 543 3,3 472 3,3 832 3,3 745 3,3 6244

3,32 548 3,32 459 3,32 821 3,32 678 3,32 5836

3,34 601 3,34 485 3,34 810 3,34 574 3,34 5471

3,36 584 3,36 491 3,36 872 3,36 604 3,36 4964

3,38 638 3,38 498 3,38 836 3,38 603 3,38 4677

3,4 670 3,4 521 3,4 807 3,4 604 3,4 4425

3,42 633 3,42 515 3,42 926 3,42 564 3,42 4046

3,44 686 3,44 550 3,44 934 3,44 569 3,44 3733

3,46 778 3,46 558 3,46 1075 3,46 574 3,46 3486

3,48 779 3,48 586 3,48 1154 3,48 634 3,48 3356

3,5 898 3,5 623 3,5 1241 3,5 601 3,5 3213

3,52 936 3,52 681 3,52 1473 3,52 603 3,52 2954

3,54 1087 3,54 678 3,54 1672 3,54 591 3,54 2810

3,56 1248 3,56 765 3,56 1956 3,56 493 3,56 2741

3,58 1418 3,58 752 3,58 2450 3,58 529 3,58 2676

3,6 1532 3,6 821 3,6 2797 3,6 480 3,6 2547

3,62 1459 3,62 919 3,62 3086 3,62 448 3,62 2421

3,64 1021 3,64 775 3,64 2736 3,64 471 3,64 2476

3,66 580 3,66 544 3,66 1834 3,66 439 3,66 2380

3,68 430 3,68 403 3,68 1076 3,68 418 3,68 2326

3,7 434 3,7 362 3,7 798 3,7 409 3,7 2366

3,72 381 3,72 381 3,72 687 3,72 445 3,72 2325

3,74 359 3,74 368 3,74 634 3,74 451 3,74 2217

3,76 387 3,76 347 3,76 603 3,76 411 3,76 2173

3,78 336 3,78 354 3,78 487 3,78 437 3,78 2299

3,8 348 3,8 379 3,8 423 3,8 423 3,8 2189

3,82 334 3,82 377 3,82 392 3,82 408 3,82 2232

3,84 353 3,84 340 3,84 347 3,84 428 3,84 2162

3,86 315 3,86 315 3,86 327 3,86 424 3,86 2231

3,88 342 3,88 334 3,88 346 3,88 424 3,88 2194

3,9 355 3,9 326 3,9 366 3,9 406 3,9 2173

3,92 334 3,92 335 3,92 331 3,92 435 3,92 2179

3,94 347 3,94 336 3,94 307 3,94 423 3,94 2131

3,96 314 3,96 317 3,96 345 3,96 425 3,96 2183

3,98 332 3,98 340 3,98 313 3,98 388 3,98 2112

4 312 4 318 4 320 4 448 4 2210

4,02 292 4,02 335 4,02 360 4,02 410 4,02 2172

4,04 335 4,04 318 4,04 342 4,04 416 4,04 2098

4,06 328 4,06 324 4,06 325 4,06 407 4,06 2247

4,08 310 4,08 303 4,08 352 4,08 427 4,08 2191

4,1 329 4,1 295 4,1 325 4,1 386 4,1 2126

4,12 288 4,12 313 4,12 366 4,12 401 4,12 2166

4,14 313 4,14 330 4,14 326 4,14 417 4,14 2132

4,16 319 4,16 288 4,16 318 4,16 374 4,16 2108

4,18 309 4,18 331 4,18 305 4,18 418 4,18 2182

4,2 274 4,2 306 4,2 357 4,2 416 4,2 2141

4,22 323 4,22 281 4,22 341 4,22 365 4,22 2175

4,24 289 4,24 310 4,24 296 4,24 417 4,24 2112

4,26 314 4,26 304 4,26 323 4,26 384 4,26 2196

4,28 270 4,28 310 4,28 341 4,28 376 4,28 2130

4,3 322 4,3 314 4,3 302 4,3 400 4,3 2174

4,32 284 4,32 292 4,32 273 4,32 398 4,32 2168

4,34 304 4,34 298 4,34 300 4,34 369 4,34 2092

4,36 300 4,36 302 4,36 299 4,36 361 4,36 2127

4,38 295 4,38 298 4,38 316 4,38 422 4,38 2155

4,4 273 4,4 304 4,4 305 4,4 381 4,4 2168

4,42 302 4,42 300 4,42 289 4,42 402 4,42 2138

4,44 314 4,44 307 4,44 280 4,44 367 4,44 2110

4,46 293 4,46 300 4,46 296 4,46 375 4,46 2133

4,48 297 4,48 273 4,48 292 4,48 405 4,48 2142

4,5 286 4,5 291 4,5 339 4,5 384 4,5 2149

4,52 292 4,52 298 4,52 268 4,52 377 4,52 2138

4,54 302 4,54 279 4,54 285 4,54 355 4,54 2174

4,56 244 4,56 267 4,56 291 4,56 368 4,56 2103

4,58 277 4,58 283 4,58 253 4,58 380 4,58 2110

4,6 297 4,6 288 4,6 280 4,6 390 4,6 2122

4,62 292 4,62 278 4,62 285 4,62 374 4,62 2093

4,64 268 4,64 267 4,64 287 4,64 407 4,64 2064

4,66 260 4,66 327 4,66 278 4,66 414 4,66 2114

4,68 305 4,68 287 4,68 283 4,68 374 4,68 2036

4,7 251 4,7 287 4,7 270 4,7 370 4,7 2180

4,72 271 4,72 271 4,72 254 4,72 379 4,72 2109

4,74 259 4,74 262 4,74 256 4,74 394 4,74 2167

4,76 278 4,76 274 4,76 271 4,76 390 4,76 2140

4,78 271 4,78 271 4,78 254 4,78 372 4,78 2174

4,8 262 4,8 261 4,8 262 4,8 372 4,8 2124

4,82 274 4,82 262 4,82 248 4,82 365 4,82 2129

4,84 263 4,84 268 4,84 281 4,84 371 4,84 2139

4,86 286 4,86 284 4,86 299 4,86 379 4,86 2162

4,88 244 4,88 245 4,88 284 4,88 396 4,88 2166

4,9 266 4,9 278 4,9 276 4,9 360 4,9 2128

4,92 279 4,92 271 4,92 260 4,92 359 4,92 2089

4,94 278 4,94 259 4,94 274 4,94 365 4,94 2151

4,96 265 4,96 270 4,96 244 4,96 394 4,96 2149

4,98 241 4,98 242 4,98 272 4,98 383 4,98 2133

5 254 5 276 5 267 5 378 5 2243

5,02 257 5,02 263 5,02 243 5,02 364 5,02 2116

5,04 240 5,04 237 5,04 245 5,04 376 5,04 2090

5,06 258 5,06 235 5,06 251 5,06 407 5,06 2207

5,08 258 5,08 274 5,08 241 5,08 395 5,08 2134

5,1 237 5,1 284 5,1 254 5,1 370 5,1 2183

5,12 263 5,12 241 5,12 247 5,12 392 5,12 2084

5,14 251 5,14 271 5,14 250 5,14 367 5,14 2147

5,16 253 5,16 263 5,16 244 5,16 390 5,16 2135

5,18 228 5,18 253 5,18 249 5,18 348 5,18 2174

5,2 255 5,2 251 5,2 229 5,2 355 5,2 2193

5,22 220 5,22 221 5,22 236 5,22 391 5,22 2167

5,24 246 5,24 248 5,24 276 5,24 419 5,24 2194

5,26 224 5,26 248 5,26 246 5,26 407 5,26 2224

5,28 245 5,28 255 5,28 248 5,28 398 5,28 2210

5,3 235 5,3 253 5,3 260 5,3 391 5,3 2108

5,32 249 5,32 250 5,32 247 5,32 364 5,32 2218

5,34 219 5,34 247 5,34 248 5,34 376 5,34 2239

5,36 261 5,36 246 5,36 258 5,36 373 5,36 2166

5,38 208 5,38 242 5,38 266 5,38 371 5,38 2142

5,4 227 5,4 240 5,4 235 5,4 408 5,4 2230

5,42 218 5,42 256 5,42 263 5,42 353 5,42 2131

5,44 222 5,44 244 5,44 227 5,44 374 5,44 2272

5,46 222 5,46 240 5,46 247 5,46 381 5,46 2200

5,48 234 5,48 229 5,48 256 5,48 434 5,48 2177

5,5 251 5,5 229 5,5 248 5,5 371 5,5 2196

5,52 229 5,52 226 5,52 240 5,52 363 5,52 2272

5,54 235 5,54 226 5,54 231 5,54 362 5,54 2205

5,56 243 5,56 233 5,56 244 5,56 389 5,56 2189

5,58 210 5,58 202 5,58 266 5,58 377 5,58 2219

5,6 233 5,6 222 5,6 236 5,6 383 5,6 2148

5,62 231 5,62 244 5,62 272 5,62 394 5,62 2141

5,64 241 5,64 233 5,64 266 5,64 384 5,64 2182

5,66 232 5,66 258 5,66 234 5,66 384 5,66 2232

5,68 238 5,68 219 5,68 250 5,68 352 5,68 2130

5,7 212 5,7 229 5,7 256 5,7 405 5,7 2125

5,72 207 5,72 243 5,72 255 5,72 375 5,72 2165

5,74 223 5,74 212 5,74 273 5,74 356 5,74 2195

5,76 223 5,76 198 5,76 277 5,76 385 5,76 2141

5,78 226 5,78 218 5,78 288 5,78 355 5,78 2254

5,8 212 5,8 244 5,8 285 5,8 374 5,8 2173

5,82 233 5,82 249 5,82 280 5,82 380 5,82 2232

5,84 221 5,84 217 5,84 261 5,84 329 5,84 2163

5,86 256 5,86 196 5,86 314 5,86 343 5,86 2205

5,88 224 5,88 244 5,88 297 5,88 349 5,88 2283

5,9 195 5,9 260 5,9 335 5,9 364 5,9 2243

5,92 206 5,92 239 5,92 322 5,92 356 5,92 2152

5,94 235 5,94 210 5,94 359 5,94 379 5,94 2294

5,96 220 5,96 242 5,96 361 5,96 353 5,96 2233

5,98 219 5,98 222 5,98 402 5,98 305 5,98 2150

6 218 6 230 6 336 6 391 6 2217

6,02 239 6,02 215 6,02 411 6,02 334 6,02 2291

6,04 200 6,04 238 6,04 438 6,04 366 6,04 2239

6,06 220 6,06 225 6,06 457 6,06 358 6,06 2400

6,08 236 6,08 212 6,08 487 6,08 385 6,08 2333

6,1 199 6,1 197 6,1 547 6,1 372 6,1 2289

6,12 230 6,12 248 6,12 550 6,12 362 6,12 2458

6,14 202 6,14 222 6,14 560 6,14 366 6,14 2575

6,16 194 6,16 207 6,16 614 6,16 414 6,16 2505

6,18 223 6,18 233 6,18 623 6,18 371 6,18 2688

6,2 185 6,2 227 6,2 630 6,2 358 6,2 2724

6,22 184 6,22 231 6,22 603 6,22 340 6,22 2871

6,24 230 6,24 203 6,24 526 6,24 375 6,24 2970

6,26 188 6,26 225 6,26 553 6,26 380 6,26 3020

6,28 227 6,28 228 6,28 552 6,28 376 6,28 3318

6,3 185 6,3 232 6,3 436 6,3 386 6,3 3331

6,32 206 6,32 209 6,32 442 6,32 368 6,32 3440

6,34 206 6,34 188 6,34 371 6,34 422 6,34 3065

6,36 191 6,36 200 6,36 341 6,36 422 6,36 3013

6,38 196 6,38 195 6,38 339 6,38 458 6,38 2723

6,4 216 6,4 226 6,4 295 6,4 398 6,4 2469

6,42 189 6,42 196 6,42 274 6,42 496 6,42 2263

6,44 180 6,44 191 6,44 289 6,44 539 6,44 2152

6,46 187 6,46 173 6,46 256 6,46 516 6,46 2066

6,48 200 6,48 179 6,48 241 6,48 538 6,48 1989

6,5 203 6,5 210 6,5 248 6,5 516 6,5 1961

6,52 221 6,52 200 6,52 237 6,52 447 6,52 1869

6,54 198 6,54 195 6,54 221 6,54 423 6,54 1876

6,56 215 6,56 199 6,56 227 6,56 372 6,56 1736

6,58 203 6,58 213 6,58 230 6,58 328 6,58 1789

6,6 187 6,6 227 6,6 237 6,6 354 6,6 1764

6,62 196 6,62 183 6,62 224 6,62 347 6,62 1765

6,64 204 6,64 201 6,64 227 6,64 339 6,64 1704

6,66 203 6,66 201 6,66 220 6,66 311 6,66 1684

6,68 202 6,68 175 6,68 249 6,68 306 6,68 1706

6,7 199 6,7 196 6,7 254 6,7 340 6,7 1647

6,72 217 6,72 192 6,72 207 6,72 302 6,72 1687

6,74 203 6,74 180 6,74 214 6,74 307 6,74 1651

6,76 203 6,76 195 6,76 241 6,76 316 6,76 1668

6,78 204 6,78 211 6,78 238 6,78 312 6,78 1603

6,8 194 6,8 173 6,8 237 6,8 288 6,8 1631

6,82 201 6,82 202 6,82 227 6,82 317 6,82 1627

6,84 221 6,84 188 6,84 235 6,84 289 6,84 1670

6,86 222 6,86 204 6,86 229 6,86 281 6,86 1585

6,88 198 6,88 199 6,88 251 6,88 289 6,88 1545

6,9 249 6,9 193 6,9 240 6,9 338 6,9 1485

6,92 222 6,92 206 6,92 246 6,92 325 6,92 1594

6,94 266 6,94 239 6,94 298 6,94 305 6,94 1538

6,96 228 6,96 178 6,96 301 6,96 345 6,96 1498

6,98 253 6,98 246 6,98 321 6,98 338 6,98 1462

7 259 7 221 7 351 7 325 7 1561

7,02 268 7,02 229 7,02 363 7,02 306 7,02 1549

7,04 292 7,04 233 7,04 413 7,04 313 7,04 1480

7,06 281 7,06 254 7,06 413 7,06 315 7,06 1520

7,08 344 7,08 260 7,08 464 7,08 293 7,08 1497

7,1 350 7,1 268 7,1 471 7,1 291 7,1 1455

7,12 438 7,12 261 7,12 622 7,12 260 7,12 1456

7,14 464 7,14 265 7,14 804 7,14 229 7,14 1488

7,16 519 7,16 305 7,16 889 7,16 240 7,16 1469

7,18 599 7,18 354 7,18 1142 7,18 233 7,18 1465

7,2 663 7,2 364 7,2 1361 7,2 240 7,2 1425

7,22 559 7,22 363 7,22 1541 7,22 248 7,22 1376

7,24 424 7,24 318 7,24 1198 7,24 247 7,24 1391

7,26 275 7,26 242 7,26 836 7,26 233 7,26 1418

7,28 196 7,28 192 7,28 484 7,28 224 7,28 1378

7,3 208 7,3 205 7,3 372 7,3 233 7,3 1413

7,32 201 7,32 183 7,32 321 7,32 266 7,32 1315

7,34 181 7,34 190 7,34 277 7,34 215 7,34 1410

7,36 178 7,36 191 7,36 248 7,36 222 7,36 1358

7,38 188 7,38 207 7,38 238 7,38 242 7,38 1443

7,4 173 7,4 185 7,4 197 7,4 237 7,4 1371

7,42 188 7,42 175 7,42 172 7,42 223 7,42 1283

7,44 174 7,44 155 7,44 189 7,44 214 7,44 1355

7,46 182 7,46 197 7,46 179 7,46 255 7,46 1297

7,48 194 7,48 180 7,48 190 7,48 243 7,48 1252

7,5 191 7,5 163 7,5 207 7,5 215 7,5 1325

7,52 186 7,52 186 7,52 193 7,52 191 7,52 1322

7,54 173 7,54 176 7,54 166 7,54 202 7,54 1310

7,56 187 7,56 163 7,56 191 7,56 215 7,56 1243

7,58 154 7,58 163 7,58 174 7,58 245 7,58 1340

7,6 159 7,6 159 7,6 183 7,6 214 7,6 1307

7,62 166 7,62 185 7,62 177 7,62 219 7,62 1273

7,64 160 7,64 185 7,64 172 7,64 242 7,64 1264

7,66 190 7,66 156 7,66 170 7,66 237 7,66 1251

7,68 164 7,68 161 7,68 187 7,68 224 7,68 1261

7,7 158 7,7 184 7,7 172 7,7 215 7,7 1301

7,72 168 7,72 175 7,72 183 7,72 199 7,72 1174

7,74 165 7,74 146 7,74 160 7,74 223 7,74 1240

7,76 180 7,76 163 7,76 177 7,76 220 7,76 1188

7,78 169 7,78 151 7,78 174 7,78 207 7,78 1213

7,8 165 7,8 161 7,8 143 7,8 225 7,8 1264

7,82 164 7,82 179 7,82 165 7,82 221 7,82 1186

7,84 173 7,84 157 7,84 162 7,84 187 7,84 1156

7,86 168 7,86 184 7,86 166 7,86 226 7,86 1178

7,88 169 7,88 165 7,88 151 7,88 204 7,88 1214

7,9 165 7,9 157 7,9 173 7,9 202 7,9 1191

7,92 178 7,92 174 7,92 141 7,92 197 7,92 1238

7,94 156 7,94 187 7,94 159 7,94 204 7,94 1261

7,96 179 7,96 175 7,96 166 7,96 207 7,96 1134

7,98 167 7,98 155 7,98 153 7,98 193 7,98 1185

8 167 8 161 8 159 8 198 8 1275

8,02 159 8,02 172 8,02 170 8,02 196 8,02 1199

8,04 158 8,04 159 8,04 192 8,04 189 8,04 1193

8,05 189 8,05 149 8,05 145 8,05 193 8,05 1190

8,08 168 8,08 165 8,08 166 8,08 202 8,08 1171

8,09 157 8,09 157 8,09 153 8,09 207 8,09 1145

8,12 160 8,12 181 8,12 153 8,12 194 8,12 1168

8,14 184 8,14 172 8,14 159 8,14 175 8,14 1124

8,16 191 8,16 158 8,16 164 8,16 197 8,16 1102

8,18 156 8,18 185 8,18 143 8,18 187 8,18 1126

8,2 155 8,2 186 8,2 170 8,2 171 8,2 1083

8,22 166 8,22 152 8,22 141 8,22 179 8,22 1151

8,24 172 8,24 174 8,24 158 8,24 172 8,24 1175

8,26 175 8,26 166 8,26 145 8,26 185 8,26 1088

8,28 192 8,28 188 8,28 164 8,28 187 8,28 1172

8,3 161 8,3 167 8,3 143 8,3 231 8,3 1102

8,32 170 8,32 159 8,32 143 8,32 228 8,32 1152

8,34 159 8,34 162 8,34 155 8,34 178 8,34 1132

8,36 163 8,36 155 8,36 144 8,36 165 8,36 1090

8,38 160 8,38 171 8,38 164 8,38 182 8,38 1083

8,4 159 8,4 173 8,4 160 8,4 217 8,4 1096

8,42 165 8,42 156 8,42 166 8,42 212 8,42 1125

8,44 179 8,44 163 8,44 160 8,44 209 8,44 1104

8,46 150 8,46 164 8,46 127 8,46 155 8,46 1133

8,48 146 8,48 156 8,48 178 8,48 198 8,48 1102

8,5 161 8,5 145 8,5 179 8,5 197 8,5 1123

8,52 151 8,52 145 8,52 143 8,52 190 8,52 1059

8,54 157 8,54 139 8,54 159 8,54 178 8,54 1078

8,55 175 8,55 176 8,55 161 8,55 209 8,55 1066

8,58 149 8,58 175 8,58 160 8,58 174 8,58 1005

8,59 182 8,59 147 8,59 162 8,59 190 8,59 1019

8,62 183 8,62 169 8,62 159 8,62 178 8,62 1109

8,64 160 8,64 209 8,64 148 8,64 190 8,64 1050

8,66 166 8,66 159 8,66 155 8,66 201 8,66 1081

8,68 153 8,68 148 8,68 149 8,68 178 8,68 1026

8,7 165 8,7 186 8,7 162 8,7 193 8,7 1035

8,72 173 8,72 149 8,72 164 8,72 195 8,72 1055

8,74 169 8,74 164 8,74 163 8,74 195 8,74 1083

8,76 156 8,76 149 8,76 165 8,76 166 8,76 999

8,78 177 8,78 164 8,78 154 8,78 184 8,78 1077

8,8 180 8,8 178 8,8 142 8,8 176 8,8 1062

8,82 156 8,82 159 8,82 159 8,82 194 8,82 1044

8,84 163 8,84 176 8,84 156 8,84 172 8,84 1034

8,86 145 8,86 140 8,86 173 8,86 163 8,86 984

8,88 160 8,88 150 8,88 170 8,88 151 8,88 1051

8,9 166 8,9 171 8,9 141 8,9 185 8,9 991

8,92 172 8,92 165 8,92 164 8,92 159 8,92 1057

8,94 149 8,94 139 8,94 175 8,94 179 8,94 1040

8,96 158 8,96 184 8,96 189 8,96 180 8,96 1050

8,98 134 8,98 175 8,98 194 8,98 188 8,98 1036

9 144 9 166 9 195 9 173 9 1081

9,02 154 9,02 177 9,02 167 9,02 158 9,02 1079

9,04 161 9,04 185 9,04 159 9,04 181 9,04 1068

9,05 182 9,05 182 9,05 194 9,05 188 9,05 1092

9,08 165 9,08 163 9,08 199 9,08 168 9,08 1065

9,09 180 9,09 186 9,09 212 9,09 152 9,09 1064

9,12 151 9,12 164 9,12 213 9,12 174 9,12 1054

9,13 136 9,13 149 9,13 283 9,13 156 9,13 1057

9,16 156 9,16 171 9,16 254 9,16 180 9,16 1078

9,18 165 9,18 189 9,18 286 9,18 180 9,18 1036

9,2 173 9,2 177 9,2 303 9,2 193 9,2 1113

9,22 164 9,22 180 9,22 289 9,22 175 9,22 1126

9,24 160 9,24 161 9,24 298 9,24 174 9,24 1140

9,26 150 9,26 150 9,26 350 9,26 186 9,26 1171

9,28 149 9,28 145 9,28 354 9,28 174 9,28 1157

9,3 140 9,3 175 9,3 368 9,3 168 9,3 1217

9,32 147 9,32 158 9,32 357 9,32 168 9,32 1198

9,34 153 9,34 134 9,34 339 9,34 169 9,34 1178

9,36 171 9,36 161 9,36 333 9,36 198 9,36 1322

9,38 138 9,38 159 9,38 292 9,38 177 9,38 1263

9,4 146 9,4 175 9,4 257 9,4 174 9,4 1360

9,42 141 9,42 152 9,42 260 9,42 208 9,42 1415

9,44 151 9,44 149 9,44 224 9,44 182 9,44 1467

9,46 168 9,46 159 9,46 206 9,46 185 9,46 1504

9,48 146 9,48 150 9,48 202 9,48 179 9,48 1723

9,5 156 9,5 154 9,5 175 9,5 212 9,5 1818

9,52 159 9,52 151 9,52 166 9,52 205 9,52 1866

9,54 131 9,54 163 9,54 197 9,54 192 9,54 1807

9,55 152 9,55 145 9,55 154 9,55 192 9,55 1759

9,58 168 9,58 150 9,58 171 9,58 191 9,58 1687

9,59 148 9,59 163 9,59 156 9,59 249 9,59 1559

9,62 140 9,62 141 9,62 154 9,62 252 9,62 1495

9,63 133 9,63 148 9,63 164 9,63 254 9,63 1355

9,66 156 9,66 165 9,66 137 9,66 256 9,66 1295

9,68 145 9,68 147 9,68 150 9,68 321 9,68 1190

9,7 145 9,7 148 9,7 142 9,7 310 9,7 1169

9,72 143 9,72 143 9,72 159 9,72 316 9,72 1102

9,74 153 9,74 131 9,74 132 9,74 301 9,74 1094

9,76 153 9,76 149 9,76 140 9,76 230 9,76 1071

9,78 126 9,78 150 9,78 150 9,78 227 9,78 1084

9,8 139 9,8 156 9,8 125 9,8 210 9,8 1049

9,82 142 9,82 153 9,82 135 9,82 196 9,82 1080

9,84 128 9,84 118 9,84 144 9,84 186 9,84 1028

9,86 131 9,86 133 9,86 132 9,86 184 9,86 1052

9,88 156 9,88 153 9,88 117 9,88 175 9,88 972

9,9 145 9,9 149 9,9 142 9,9 174 9,9 991

9,92 149 9,92 157 9,92 110 9,92 194 9,92 1015

9,94 138 9,94 154 9,94 140 9,94 187 9,94 937

9,96 128 9,96 146 9,96 135 9,96 169 9,96 969

9,98 152 9,98 118 9,98 147 9,98 186 9,98 949

10 155 10 151 10 144 10 139 10 993

10,02 128 10,02 130 10,02 143 10,02 174 10,02 985

10,04 134 10,04 137 10,04 123 10,04 190 10,04 980

10,06 140 10,06 137 10,06 127 10,06 211 10,06 955

10,08 148 10,08 150 10,08 127 10,08 145 10,08 953

10,1 153 10,1 160 10,1 132 10,1 185 10,1 972

10,12 146 10,12 133 10,12 136 10,12 185 10,12 1013

10,14 119 10,14 177 10,14 114 10,14 152 10,14 940

10,16 140 10,16 131 10,16 142 10,16 155 10,16 955

10,18 148 10,18 135 10,18 118 10,18 167 10,18 923

10,2 139 10,2 155 10,2 127 10,2 178 10,2 993

10,22 148 10,22 126 10,22 143 10,22 185 10,22 963

10,24 149 10,24 141 10,24 132 10,24 165 10,24 965

10,26 130 10,26 134 10,26 145 10,26 160 10,26 966

10,28 132 10,28 144 10,28 113 10,28 176 10,28 914

10,3 145 10,3 124 10,3 127 10,3 171 10,3 893

10,32 150 10,32 143 10,32 145 10,32 183 10,32 969

10,34 167 10,34 113 10,34 117 10,34 172 10,34 970

10,36 151 10,36 102 10,36 121 10,36 151 10,36 888

10,38 142 10,38 158 10,38 156 10,38 199 10,38 970

10,4 136 10,4 130 10,4 145 10,4 202 10,4 977

10,42 149 10,42 159 10,42 179 10,42 183 10,42 942

10,44 144 10,44 170 10,44 152 10,44 208 10,44 869

10,46 162 10,46 159 10,46 165 10,46 208 10,46 933

10,48 148 10,48 128 10,48 155 10,48 234 10,48 998

10,5 166 10,5 169 10,5 186 10,5 221 10,5 939

10,52 185 10,52 138 10,52 172 10,52 198 10,52 946

10,54 166 10,54 178 10,54 177 10,54 215 10,54 931

10,56 190 10,56 148 10,56 174 10,56 196 10,56 946

10,58 174 10,58 178 10,58 225 10,58 205 10,58 948

10,6 200 10,6 188 10,6 234 10,6 177 10,6 961

10,62 193 10,62 171 10,62 240 10,62 171 10,62 965

10,64 194 10,64 214 10,64 298 10,64 181 10,64 903

10,66 240 10,66 210 10,66 312 10,66 158 10,66 924

10,68 234 10,68 216 10,68 352 10,68 181 10,68 939

10,7 293 10,7 191 10,7 468 10,7 164 10,7 948

10,72 345 10,72 196 10,72 548 10,72 154 10,72 936

10,74 375 10,74 198 10,74 556 10,74 153 10,74 910

10,76 501 10,76 199 10,76 689 10,76 163 10,76 906

10,78 535 10,78 238 10,78 871 10,78 192 10,78 976

10,8 554 10,8 290 10,8 1058 10,8 156 10,8 926

10,82 516 10,82 306 10,82 1197 10,82 147 10,82 947

10,84 382 10,84 261 10,84 1074 10,84 149 10,84 960

10,86 246 10,86 224 10,86 815 10,86 139 10,86 904

10,88 177 10,88 186 10,88 567 10,88 186 10,88 944

10,9 160 10,9 132 10,9 400 10,9 167 10,9 931

10,92 158 10,92 126 10,92 267 10,92 149 10,92 883

10,94 128 10,94 130 10,94 242 10,94 167 10,94 928

10,96 109 10,96 106 10,96 201 10,96 161 10,96 977

10,98 140 10,98 127 10,98 184 10,98 144 10,98 975

11 116 11 138 11 162 11 151 11 890

11,02 144 11,02 144 11,02 146 11,02 153 11,02 979

11,04 133 11,04 132 11,04 123 11,04 146 11,04 900

11,06 119 11,06 133 11,06 148 11,06 159 11,06 892

11,08 147 11,08 137 11,08 111 11,08 132 11,08 960

11,1 151 11,1 123 11,1 143 11,1 160 11,1 899

11,12 137 11,12 144 11,12 125 11,12 130 11,12 925

11,14 123 11,14 128 11,14 130 11,14 161 11,14 978

11,16 109 11,16 113 11,16 140 11,16 166 11,16 1006

11,18 105 11,18 122 11,18 116 11,18 166 11,18 921

11,2 130 11,2 109 11,2 130 11,2 162 11,2 928

11,22 114 11,22 123 11,22 119 11,22 151 11,22 954

11,24 97 11,24 151 11,24 118 11,24 171 11,24 904

11,26 138 11,26 137 11,26 119 11,26 150 11,26 913

11,28 110 11,28 103 11,28 132 11,28 143 11,28 901

11,3 132 11,3 109 11,3 114 11,3 149 11,3 942

11,32 114 11,32 132 11,32 125 11,32 161 11,32 905

11,34 109 11,34 114 11,34 115 11,34 158 11,34 953

11,36 126 11,36 113 11,36 129 11,36 150 11,36 933

11,38 125 11,38 112 11,38 116 11,38 140 11,38 907

11,4 125 11,4 142 11,4 110 11,4 159 11,4 1002

11,42 144 11,42 138 11,42 125 11,42 155 11,42 899

11,44 119 11,44 127 11,44 117 11,44 148 11,44 931

11,46 130 11,46 147 11,46 118 11,46 130 11,46 879

11,48 113 11,48 115 11,48 100 11,48 131 11,48 936

11,5 112 11,5 119 11,5 102 11,5 148 11,5 883

11,52 104 11,52 127 11,52 104 11,52 183 11,52 881

11,54 116 11,54 123 11,54 118 11,54 171 11,54 896

11,56 118 11,56 147 11,56 129 11,56 158 11,56 921

11,58 110 11,58 133 11,58 112 11,58 167 11,58 943

11,6 118 11,6 130 11,6 100 11,6 135 11,6 913

11,62 137 11,62 131 11,62 104 11,62 156 11,62 933

11,64 113 11,64 137 11,64 125 11,64 172 11,64 883

11,66 129 11,66 137 11,66 104 11,66 194 11,66 959

11,68 110 11,68 129 11,68 113 11,68 134 11,68 938

11,7 104 11,7 136 11,7 107 11,7 131 11,7 891

11,72 125 11,72 109 11,72 125 11,72 124 11,72 940

11,74 108 11,74 127 11,74 109 11,74 141 11,74 875

11,76 133 11,76 119 11,76 112 11,76 162 11,76 892

11,78 127 11,78 91 11,78 122 11,78 149 11,78 953

11,8 124 11,8 124 11,8 133 11,8 152 11,8 951

11,82 121 11,82 130 11,82 109 11,82 145 11,82 922

11,84 113 11,84 119 11,84 113 11,84 138 11,84 851

11,86 114 11,86 123 11,86 111 11,86 150 11,86 882

11,88 106 11,88 123 11,88 98 11,88 136 11,88 928

11,9 118 11,9 120 11,9 131 11,9 138 11,9 925

11,92 104 11,92 130 11,92 117 11,92 168 11,92 876

11,94 110 11,94 119 11,94 101 11,94 153 11,94 884

11,96 119 11,96 135 11,96 105 11,96 148 11,96 934

11,98 119 11,98 117 11,98 124 11,98 148 11,98 921

12 125 12 120 12 113 12 169 12 909

12,02 110 12,02 131 12,02 107 12,02 147 12,02 863

12,04 109 12,04 118 12,04 122 12,04 133 12,04 927

12,06 109 12,06 103 12,06 130 12,06 144 12,06 887

12,08 103 12,08 126 12,08 131 12,08 138 12,08 881

12,1 104 12,1 127 12,1 132 12,1 162 12,1 901

12,12 119 12,12 128 12,12 121 12,12 155 12,12 861

12,14 95 12,14 135 12,14 150 12,14 138 12,14 931

12,16 119 12,16 127 12,16 127 12,16 136 12,16 879

12,18 102 12,18 119 12,18 137 12,18 145 12,18 906

12,2 99 12,2 110 12,2 148 12,2 144 12,2 918

12,22 119 12,22 124 12,22 145 12,22 157 12,22 923

12,24 133 12,24 135 12,24 141 12,24 149 12,24 903

12,26 105 12,26 126 12,26 154 12,26 153 12,26 892

12,28 111 12,28 123 12,28 178 12,28 152 12,28 941

12,3 97 12,3 127 12,3 171 12,3 137 12,3 913

12,32 99 12,32 140 12,32 198 12,32 169 12,32 892

12,34 130 12,34 121 12,34 198 12,34 122 12,34 925

12,36 139 12,36 143 12,36 222 12,36 161 12,36 911

12,38 150 12,38 143 12,38 211 12,38 164 12,38 886

12,4 120 12,4 139 12,4 171 12,4 152 12,4 923

12,42 116 12,42 141 12,42 191 12,42 162 12,42 917

12,44 116 12,44 137 12,44 223 12,44 172 12,44 899

12,46 110 12,46 122 12,46 169 12,46 146 12,46 916

12,48 121 12,48 129 12,48 178 12,48 144 12,48 854

12,5 105 12,5 108 12,5 167 12,5 131 12,5 930

12,52 115 12,52 107 12,52 162 12,52 151 12,52 887

12,54 101 12,54 124 12,54 155 12,54 144 12,54 955

12,56 108 12,56 126 12,56 152 12,56 150 12,56 974

12,58 111 12,58 120 12,58 134 12,58 136 12,58 946

12,6 94 12,6 106 12,6 140 12,6 153 12,6 975

12,62 92 12,62 119 12,62 130 12,62 146 12,62 1008

12,64 131 12,64 111 12,64 109 12,64 128 12,64 1099

12,66 132 12,66 98 12,66 127 12,66 167 12,66 1067

12,68 93 12,68 118 12,68 114 12,68 145 12,68 1076

12,7 108 12,7 103 12,7 111 12,7 150 12,7 1108

12,72 116 12,72 116 12,72 116 12,72 156 12,72 1200

12,74 104 12,74 103 12,74 115 12,74 171 12,74 1195

12,76 107 12,76 135 12,76 97 12,76 139 12,76 1173

12,78 94 12,78 122 12,78 106 12,78 147 12,78 1256

12,8 115 12,8 124 12,8 121 12,8 161 12,8 1157

12,82 115 12,82 127 12,82 100 12,82 158 12,82 1105

12,84 114 12,84 121 12,84 100 12,84 188 12,84 1064

12,86 127 12,86 138 12,86 109 12,86 172 12,86 1030

12,88 119 12,88 120 12,88 102 12,88 188 12,88 975

12,9 111 12,9 123 12,9 113 12,9 190 12,9 991

12,92 117 12,92 121 12,92 97 12,92 199 12,92 962

12,94 107 12,94 127 12,94 98 12,94 203 12,94 964

12,96 113 12,96 111 12,96 122 12,96 189 12,96 950

12,98 108 12,98 114 12,98 99 12,98 191 12,98 864

13 123 13 114 13 91 13 193 13 867

13,02 113 13,02 109 13,02 81 13,02 172 13,02 907

13,04 107 13,04 123 13,04 111 13,04 184 13,04 939

13,06 98 13,06 141 13,06 92 13,06 148 13,06 862

13,08 123 13,08 130 13,08 90 13,08 188 13,08 824

13,1 103 13,1 122 13,1 88 13,1 149 13,1 885

13,12 102 13,12 126 13,12 88 13,12 175 13,12 850

13,14 122 13,14 126 13,14 92 13,14 152 13,14 844

13,16 108 13,16 111 13,16 88 13,16 157 13,16 911

13,18 105 13,18 110 13,18 85 13,18 121 13,18 837

13,2 104 13,2 112 13,2 75 13,2 143 13,2 873

13,22 103 13,22 114 13,22 92 13,22 134 13,22 868

13,24 103 13,24 106 13,24 88 13,24 126 13,24 831

13,26 86 13,26 100 13,26 94 13,26 139 13,26 909

13,28 106 13,28 98 13,28 94 13,28 135 13,28 877

13,3 106 13,3 120 13,3 87 13,3 138 13,3 805

13,32 112 13,32 111 13,32 96 13,32 121 13,32 819

13,34 123 13,34 102 13,34 83 13,34 134 13,34 898

13,36 106 13,36 110 13,36 96 13,36 138 13,36 831

13,38 115 13,38 123 13,38 105 13,38 133 13,38 826

13,4 112 13,4 110 13,4 94 13,4 156 13,4 790

13,42 118 13,42 119 13,42 80 13,42 123 13,42 810

13,44 124 13,44 109 13,44 104 13,44 137 13,44 827

13,46 105 13,46 136 13,46 101 13,46 128 13,46 879

13,48 111 13,48 124 13,48 95 13,48 158 13,48 828

13,5 117 13,5 108 13,5 80 13,5 163 13,5 803

13,52 111 13,52 124 13,52 78 13,52 147 13,52 878

13,54 101 13,54 127 13,54 89 13,54 149 13,54 825

13,56 102 13,56 118 13,56 105 13,56 154 13,56 850

13,58 118 13,58 123 13,58 93 13,58 153 13,58 841

13,6 100 13,6 110 13,6 96 13,6 128 13,6 801

13,62 95 13,62 115 13,62 87 13,62 126 13,62 799

13,64 89 13,64 118 13,64 109 13,64 144 13,64 816

13,66 99 13,66 104 13,66 99 13,66 118 13,66 778

13,68 96 13,68 114 13,68 91 13,68 154 13,68 806

13,7 109 13,7 120 13,7 79 13,7 142 13,7 765

13,72 112 13,72 113 13,72 96 13,72 157 13,72 773

13,74 98 13,74 93 13,74 83 13,74 152 13,74 817

13,76 100 13,76 102 13,76 83 13,76 142 13,76 894

13,78 113 13,78 95 13,78 79 13,78 160 13,78 834

13,8 117 13,8 113 13,8 100 13,8 134 13,8 788

13,82 107 13,82 109 13,82 101 13,82 135 13,82 815

13,84 101 13,84 109 13,84 93 13,84 152 13,84 836

13,86 100 13,86 122 13,86 101 13,86 177 13,86 840

13,88 91 13,88 104 13,88 95 13,88 165 13,88 734

13,9 95 13,9 118 13,9 108 13,9 156 13,9 812

13,92 100 13,92 101 13,92 108 13,92 150 13,92 842

13,94 111 13,94 108 13,94 94 13,94 142 13,94 793

13,96 103 13,96 111 13,96 91 13,96 153 13,96 824

13,98 105 13,98 134 13,98 121 13,98 169 13,98 791

14 110 14 112 14 104 14 153 14 837

14,02 113 14,02 111 14,02 113 14,02 155 14,02 765

14,04 106 14,04 131 14,04 117 14,04 169 14,04 831

14,06 104 14,06 126 14,06 110 14,06 156 14,06 767

14,08 96 14,08 119 14,08 112 14,08 154 14,08 784

14,1 94 14,1 131 14,1 118 14,1 140 14,1 778

14,12 102 14,12 107 14,12 103 14,12 125 14,12 792

14,14 107 14,14 121 14,14 119 14,14 134 14,14 806

14,16 105 14,16 111 14,16 133 14,16 131 14,16 796

14,18 115 14,18 133 14,18 110 14,18 143 14,18 783

14,2 112 14,2 147 14,2 133 14,2 141 14,2 792

14,22 124 14,22 122 14,22 136 14,22 112 14,22 797

14,24 117 14,24 122 14,24 151 14,24 126 14,24 840

14,26 127 14,26 113 14,26 164 14,26 116 14,26 801

14,28 145 14,28 150 14,28 195 14,28 121 14,28 776

14,3 157 14,3 131 14,3 183 14,3 131 14,3 807

14,32 156 14,32 147 14,32 211 14,32 147 14,32 845

14,34 183 14,34 146 14,34 237 14,34 132 14,34 813

14,36 215 14,36 159 14,36 265 14,36 136 14,36 769

14,38 227 14,38 141 14,38 326 14,38 144 14,38 848

14,4 245 14,4 163 14,4 354 14,4 130 14,4 790

14,42 251 14,42 160 14,42 455 14,42 128 14,42 797

14,44 222 14,44 168 14,44 476 14,44 133 14,44 804

14,46 174 14,46 155 14,46 481 14,46 144 14,46 795

14,48 131 14,48 160 14,48 363 14,48 127 14,48 838

14,5 109 14,5 132 14,5 269 14,5 144 14,5 843

14,52 117 14,52 139 14,52 210 14,52 134 14,52 848

14,54 113 14,54 111 14,54 159 14,54 145 14,54 810

14,56 140 14,56 115 14,56 148 14,56 130 14,56 844

14,58 112 14,58 105 14,58 119 14,58 132 14,58 794

14,6 109 14,6 109 14,6 126 14,6 147 14,6 800

14,62 87 14,62 108 14,62 96 14,62 133 14,62 768

14,64 96 14,64 102 14,64 119 14,64 141 14,64 811

14,66 95 14,66 119 14,66 105 14,66 120 14,66 771

14,68 93 14,68 90 14,68 99 14,68 141 14,68 740

14,7 94 14,7 122 14,7 81 14,7 133 14,7 799

14,72 108 14,72 113 14,72 103 14,72 117 14,72 792

14,74 107 14,74 116 14,74 74 14,74 129 14,74 729

14,76 99 14,76 127 14,76 83 14,76 151 14,76 787

14,78 101 14,78 110 14,78 79 14,78 107 14,78 771

14,8 104 14,8 99 14,8 79 14,8 124 14,8 803

14,82 112 14,82 114 14,82 95 14,82 127 14,82 731

14,84 115 14,84 100 14,84 101 14,84 136 14,84 733

14,86 97 14,86 127 14,86 95 14,86 123 14,86 750

14,88 110 14,88 129 14,88 87 14,88 113 14,88 810

14,9 115 14,9 108 14,9 93 14,9 143 14,9 747

14,92 117 14,92 107 14,92 106 14,92 115 14,92 760

14,94 101 14,94 134 14,94 96 14,94 141 14,94 803

14,96 139 14,96 120 14,96 86 14,96 119 14,96 770

14,98 123 14,98 138 14,98 97 14,98 115 14,98 767

15 154 15 147 15 108 15 114 15 781

15,02 160 15,02 136 15,02 113 15,02 129 15,02 795

15,04 142 15,04 162 15,04 95 15,04 151 15,04 724

15,06 196 15,06 139 15,06 112 15,06 114 15,06 729

15,08 224 15,08 169 15,08 124 15,08 143 15,08 784

15,1 227 15,1 153 15,1 133 15,1 142 15,1 697

15,12 247 15,12 147 15,12 171 15,12 150 15,12 794

15,14 287 15,14 163 15,14 162 15,14 137 15,14 761

15,16 381 15,16 198 15,16 172 15,16 109 15,16 771

15,18 383 15,18 181 15,18 207 15,18 147 15,18 773

15,2 365 15,2 175 15,2 220 15,2 139 15,2 741

15,22 346 15,22 226 15,22 240 15,22 144 15,22 803

15,24 276 15,24 238 15,24 276 15,24 126 15,24 790

15,26 269 15,26 219 15,26 250 15,26 127 15,26 799

15,28 256 15,28 183 15,28 292 15,28 116 15,28 766

15,3 252 15,3 199 15,3 245 15,3 127 15,3 755

15,32 257 15,32 159 15,32 256 15,32 133 15,32 762

15,34 315 15,34 146 15,34 220 15,34 116 15,34 778

15,36 281 15,36 157 15,36 225 15,36 147 15,36 781

15,38 251 15,38 165 15,38 258 15,38 133 15,38 838

15,4 210 15,4 185 15,4 241 15,4 115 15,4 783

15,42 158 15,42 158 15,42 257 15,42 126 15,42 793

15,44 147 15,44 177 15,44 264 15,44 126 15,44 810

15,46 126 15,46 176 15,46 225 15,46 124 15,46 828

15,48 144 15,48 146 15,48 233 15,48 118 15,48 781

15,5 145 15,5 128 15,5 210 15,5 142 15,5 870

15,52 112 15,52 124 15,52 207 15,52 125 15,52 867

15,54 88 15,54 122 15,54 190 15,54 140 15,54 833

15,56 133 15,56 116 15,56 192 15,56 124 15,56 834

15,58 119 15,58 124 15,58 160 15,58 121 15,58 842

15,6 125 15,6 110 15,6 142 15,6 125 15,6 835

15,62 125 15,62 124 15,62 177 15,62 128 15,62 818

15,64 117 15,64 136 15,64 147 15,64 133 15,64 834

15,66 128 15,66 129 15,66 152 15,66 148 15,66 811

15,68 161 15,68 140 15,68 144 15,68 138 15,68 815

15,7 116 15,7 135 15,7 163 15,7 112 15,7 840

15,72 165 15,72 152 15,72 123 15,72 130 15,72 875

15,74 143 15,74 146 15,74 132 15,74 135 15,74 861

15,76 135 15,76 157 15,76 156 15,76 113 15,76 894

15,78 185 15,78 122 15,78 159 15,78 134 15,78 856

15,8 207 15,8 165 15,8 167 15,8 137 15,8 889

15,82 211 15,82 142 15,82 173 15,82 128 15,82 856

15,84 226 15,84 153 15,84 189 15,84 116 15,84 908

15,86 221 15,86 179 15,86 210 15,86 112 15,86 879

15,88 229 15,88 199 15,88 192 15,88 131 15,88 937

15,9 207 15,9 195 15,9 186 15,9 123 15,9 901

15,92 166 15,92 168 15,92 230 15,92 149 15,92 979

15,94 124 15,94 149 15,94 222 15,94 122 15,94 1016

15,96 147 15,96 146 15,96 193 15,96 138 15,96 986

15,98 135 15,98 134 15,98 181 15,98 126 15,98 1029

16 127 16 125 16 157 16 121 16 1013

16,02 113 16,02 118 16,02 136 16,02 129 16,02 1035

16,04 108 16,04 116 16,04 117 16,04 134 16,04 1030

16,06 99 16,06 118 16,06 111 16,06 161 16,06 1001

16,08 126 16,08 126 16,08 114 16,08 152 16,08 969

16,1 116 16,1 137 16,1 117 16,1 151 16,1 928

16,12 118 16,12 132 16,12 107 16,12 159 16,12 867

16,14 130 16,14 130 16,14 91 16,14 154 16,14 918

16,16 135 16,16 132 16,16 84 16,16 177 16,16 893

16,18 146 16,18 134 16,18 102 16,18 180 16,18 901

16,2 147 16,2 130 16,2 111 16,2 178 16,2 877

16,22 164 16,22 147 16,22 95 16,22 158 16,22 870

16,24 174 16,24 137 16,24 113 16,24 149 16,24 873

16,26 202 16,26 153 16,26 109 16,26 184 16,26 880

16,28 164 16,28 149 16,28 104 16,28 152 16,28 889

16,3 204 16,3 142 16,3 144 16,3 158 16,3 825

16,32 219 16,32 151 16,32 144 16,32 141 16,32 881

16,34 180 16,34 144 16,34 132 16,34 133 16,34 832

16,36 200 16,36 121 16,36 143 16,36 138 16,36 829

16,38 192 16,38 149 16,38 155 16,38 124 16,38 842

16,4 158 16,4 161 16,4 146 16,4 134 16,4 852

16,42 156 16,42 130 16,42 139 16,42 140 16,42 844

16,44 149 16,44 159 16,44 178 16,44 127 16,44 857

16,46 146 16,46 136 16,46 143 16,46 122 16,46 872

16,48 152 16,48 139 16,48 155 16,48 139 16,48 879

16,5 127 16,5 149 16,5 135 16,5 127 16,5 840

16,52 110 16,52 156 16,52 142 16,52 140 16,52 882

16,54 151 16,54 134 16,54 131 16,54 121 16,54 901

16,56 153 16,56 156 16,56 147 16,56 136 16,56 875

16,58 180 16,58 144 16,58 135 16,58 142 16,58 805

16,6 174 16,6 155 16,6 124 16,6 148 16,6 879

16,62 222 16,62 166 16,62 127 16,62 141 16,62 833

16,64 244 16,64 161 16,64 175 16,64 128 16,64 818

16,66 246 16,66 179 16,66 139 16,66 130 16,66 860

16,68 229 16,68 169 16,68 170 16,68 137 16,68 824

16,7 206 16,7 199 16,7 179 16,7 140 16,7 822

16,72 187 16,72 179 16,72 182 16,72 147 16,72 830

16,74 162 16,74 156 16,74 185 16,74 113 16,74 831

16,76 133 16,76 147 16,76 144 16,76 131 16,76 864

16,78 128 16,78 169 16,78 138 16,78 132 16,78 825

16,8 148 16,8 137 16,8 118 16,8 122 16,8 806

16,82 133 16,82 130 16,82 100 16,82 145 16,82 787

16,84 121 16,84 141 16,84 126 16,84 124 16,84 773

16,86 147 16,86 144 16,86 129 16,86 129 16,86 815

16,88 163 16,88 185 16,88 133 16,88 118 16,88 801

16,9 177 16,9 156 16,9 118 16,9 121 16,9 795

16,92 205 16,92 177 16,92 146 16,92 121 16,92 856

16,94 240 16,94 179 16,94 129 16,94 155 16,94 778

16,96 254 16,96 177 16,96 160 16,96 116 16,96 791

16,98 273 16,98 205 16,98 166 16,98 137 16,98 771

17 262 17 195 17 172 17 132 17 815

17,02 251 17,02 223 17,02 200 17,02 129 17,02 776

17,04 267 17,04 205 17,04 204 17,04 122 17,04 786

17,06 264 17,06 221 17,06 213 17,06 128 17,06 782

17,08 224 17,08 212 17,08 228 17,08 138 17,08 794

17,1 255 17,1 211 17,1 240 17,1 139 17,1 814

17,12 223 17,12 213 17,12 209 17,12 146 17,12 774

17,14 217 17,14 199 17,14 174 17,14 123 17,14 797

17,16 196 17,16 178 17,16 203 17,16 122 17,16 779

17,18 195 17,18 179 17,18 201 17,18 150 17,18 812

17,2 189 17,2 204 17,2 190 17,2 137 17,2 756

17,22 191 17,22 126 17,22 216 17,22 138 17,22 761

17,24 186 17,24 164 17,24 171 17,24 114 17,24 810

17,26 196 17,26 163 17,26 171 17,26 150 17,26 787

17,28 196 17,28 158 17,28 175 17,28 142 17,28 794

17,3 172 17,3 165 17,3 135 17,3 155 17,3 851

17,32 188 17,32 167 17,32 136 17,32 124 17,32 773

17,34 176 17,34 130 17,34 163 17,34 147 17,34 813

17,36 138 17,36 156 17,36 157 17,36 140 17,36 787

17,38 124 17,38 144 17,38 164 17,38 133 17,38 797

17,4 123 17,4 128 17,4 123 17,4 127 17,4 752

17,42 116 17,42 116 17,42 145 17,42 124 17,42 819

17,44 121 17,44 130 17,44 136 17,44 126 17,44 808

17,46 119 17,46 126 17,46 120 17,46 133 17,46 797

17,48 117 17,48 150 17,48 112 17,48 169 17,48 783

17,5 96 17,5 129 17,5 92 17,5 156 17,5 838

17,52 96 17,52 115 17,52 86 17,52 152 17,52 744

17,54 108 17,54 119 17,54 100 17,54 163 17,54 817

17,56 104 17,56 135 17,56 117 17,56 137 17,56 767

17,58 95 17,58 134 17,58 127 17,58 154 17,58 795

17,6 106 17,6 114 17,6 106 17,6 151 17,6 792

17,62 96 17,62 137 17,62 82 17,62 152 17,62 850

17,64 111 17,64 123 17,64 105 17,64 136 17,64 769

17,66 115 17,66 131 17,66 94 17,66 135 17,66 770

17,68 120 17,68 136 17,68 101 17,68 120 17,68 810

17,7 123 17,7 163 17,7 89 17,7 130 17,7 816

17,72 137 17,72 131 17,72 118 17,72 124 17,72 822

17,74 154 17,74 135 17,74 132 17,74 126 17,74 798

17,76 150 17,76 130 17,76 124 17,76 140 17,76 810

17,78 176 17,78 146 17,78 127 17,78 135 17,78 863

17,8 144 17,8 142 17,8 134 17,8 131 17,8 795

17,82 172 17,82 162 17,82 146 17,82 118 17,82 786

17,84 173 17,84 161 17,84 153 17,84 124 17,84 782

17,86 199 17,86 188 17,86 167 17,86 136 17,86 807

17,88 193 17,88 194 17,88 208 17,88 123 17,88 857

17,9 256 17,9 212 17,9 229 17,9 149 17,9 815

17,92 284 17,92 203 17,92 249 17,92 138 17,92 798

17,94 343 17,94 202 17,94 300 17,94 125 17,94 827

17,96 382 17,96 212 17,96 411 17,96 140 17,96 826

17,98 478 17,98 225 17,98 428 17,98 125 17,98 826

18 561 18 224 18 573 18 116 18 849

18,02 597 18,02 275 18,02 657 18,02 130 18,02 865

18,04 599 18,04 285 18,04 834 18,04 132 18,04 830

18,06 547 18,06 315 18,06 926 18,06 132 18,06 824

18,08 416 18,08 312 18,08 907 18,08 119 18,08 845

18,1 381 18,1 269 18,1 872 18,1 148 18,1 847

18,12 307 18,12 280 18,12 755 18,12 130 18,12 859

18,14 214 18,14 191 18,14 579 18,14 123 18,14 901

18,16 216 18,16 202 18,16 417 18,16 142 18,16 805

18,18 156 18,18 202 18,18 372 18,18 129 18,18 820

18,2 185 18,2 172 18,2 298 18,2 138 18,2 831

18,22 199 18,22 156 18,22 273 18,22 134 18,22 817

18,24 161 18,24 148 18,24 216 18,24 141 18,24 865

18,26 187 18,26 159 18,26 210 18,26 129 18,26 897

18,28 165 18,28 171 18,28 173 18,28 126 18,28 827

18,3 134 18,3 153 18,3 184 18,3 140 18,3 859

18,32 158 18,32 139 18,32 138 18,32 125 18,32 843

18,34 143 18,34 153 18,34 151 18,34 125 18,34 792

18,36 102 18,36 137 18,36 137 18,36 156 18,36 858

18,38 118 18,38 153 18,38 135 18,38 119 18,38 818

18,4 129 18,4 149 18,4 131 18,4 127 18,4 848

18,42 115 18,42 120 18,42 109 18,42 130 18,42 887

18,44 119 18,44 137 18,44 113 18,44 145 18,44 881

18,46 117 18,46 142 18,46 103 18,46 144 18,46 797

18,48 106 18,48 136 18,48 87 18,48 153 18,48 901

18,5 133 18,5 121 18,5 105 18,5 128 18,5 893

18,52 111 18,52 115 18,52 95 18,52 156 18,52 890

18,54 114 18,54 138 18,54 86 18,54 158 18,54 824

18,56 100 18,56 120 18,56 82 18,56 148 18,56 884

18,58 97 18,58 137 18,58 93 18,58 151 18,58 862

18,6 117 18,6 113 18,6 80 18,6 170 18,6 933

18,62 95 18,62 130 18,62 99 18,62 147 18,62 968

18,64 111 18,64 128 18,64 99 18,64 126 18,64 1016

18,66 99 18,66 136 18,66 82 18,66 142 18,66 1042

18,68 104 18,68 130 18,68 101 18,68 134 18,68 1040

18,7 84 18,7 125 18,7 84 18,7 163 18,7 1089

18,72 81 18,72 108 18,72 93 18,72 144 18,72 1128

18,74 119 18,74 117 18,74 85 18,74 135 18,74 1046

18,76 92 18,76 115 18,76 91 18,76 120 18,76 1046

18,78 96 18,78 147 18,78 75 18,78 144 18,78 1032

18,8 96 18,8 131 18,8 88 18,8 139 18,8 963

18,82 101 18,82 128 18,82 78 18,82 138 18,82 911

18,84 100 18,84 119 18,84 74 18,84 132 18,84 865

18,86 104 18,86 145 18,86 76 18,86 148 18,86 855

18,88 101 18,88 123 18,88 85 18,88 131 18,88 841

18,9 110 18,9 112 18,9 90 18,9 166 18,9 857

18,92 113 18,92 114 18,92 64 18,92 112 18,92 854

18,94 101 18,94 108 18,94 92 18,94 136 18,94 841

18,96 102 18,96 135 18,96 81 18,96 131 18,96 840

18,98 120 18,98 127 18,98 93 18,98 116 18,98 806

19 105 19 129 19 56 19 138 19 828

19,02 118 19,02 136 19,02 71 19,02 127 19,02 837

19,04 111 19,04 130 19,04 82 19,04 110 19,04 820

19,06 130 19,06 118 19,06 93 19,06 151 19,06 811

19,08 111 19,08 141 19,08 74 19,08 141 19,08 869

19,1 113 19,1 135 19,1 85 19,1 133 19,1 862

19,12 120 19,12 129 19,12 82 19,12 135 19,12 866

19,14 122 19,14 125 19,14 110 19,14 122 19,14 887

19,16 117 19,16 159 19,16 99 19,16 153 19,16 843

19,18 144 19,18 124 19,18 94 19,18 127 19,18 865

19,2 117 19,2 129 19,2 115 19,2 138 19,2 943

19,22 149 19,22 133 19,22 101 19,22 130 19,22 930

19,24 149 19,24 139 19,24 117 19,24 132 19,24 937

19,26 168 19,26 154 19,26 130 19,26 143 19,26 913

19,28 141 19,28 153 19,28 116 19,28 141 19,28 905

19,3 136 19,3 140 19,3 114 19,3 147 19,3 886

19,32 120 19,32 164 19,32 128 19,32 140 19,32 903

19,34 137 19,34 134 19,34 113 19,34 138 19,34 918

19,36 135 19,36 146 19,36 126 19,36 159 19,36 835

19,38 107 19,38 132 19,38 123 19,38 152 19,38 874

19,4 123 19,4 143 19,4 126 19,4 119 19,4 872

19,42 128 19,42 119 19,42 116 19,42 152 19,42 842

19,44 120 19,44 116 19,44 110 19,44 155 19,44 828

19,46 111 19,46 147 19,46 122 19,46 152 19,46 851

19,48 121 19,48 118 19,48 122 19,48 150 19,48 840

19,5 124 19,5 136 19,5 100 19,5 153 19,5 863

19,52 149 19,52 133 19,52 100 19,52 141 19,52 826

19,54 120 19,54 151 19,54 106 19,54 137 19,54 817

19,56 138 19,56 143 19,56 124 19,56 130 19,56 792

19,58 125 19,58 146 19,58 99 19,58 153 19,58 793

19,6 131 19,6 154 19,6 120 19,6 141 19,6 821

19,62 176 19,62 150 19,62 125 19,62 141 19,62 870

19,64 151 19,64 171 19,64 116 19,64 121 19,64 827

19,66 171 19,66 172 19,66 120 19,66 126 19,66 785

19,68 223 19,68 180 19,68 119 19,68 143 19,68 864

19,7 209 19,7 196 19,7 124 19,7 133 19,7 821

19,72 209 19,72 183 19,72 140 19,72 135 19,72 853

19,74 283 19,74 207 19,74 164 19,74 136 19,74 838

19,76 265 19,76 229 19,76 156 19,76 134 19,76 756

19,78 307 19,78 236 19,78 183 19,78 158 19,78 853

19,8 327 19,8 219 19,8 173 19,8 140 19,8 850

19,82 410 19,82 243 19,82 203 19,82 129 19,82 876

19,84 426 19,84 245 19,84 181 19,84 137 19,84 875

19,86 449 19,86 248 19,86 193 19,86 145 19,86 877

19,88 377 19,88 255 19,88 226 19,88 142 19,88 881

19,9 329 19,9 279 19,9 250 19,9 125 19,9 900

19,92 311 19,92 302 19,92 260 19,92 160 19,92 846

19,94 315 19,94 251 19,94 263 19,94 125 19,94 831

19,96 319 19,96 251 19,96 234 19,96 117 19,96 867

19,98 365 19,98 260 19,98 231 19,98 150 19,98 890

20 429 20 210 20 217 20 156 20 874

20,02 449 20,02 236 20,02 254 20,02 137 20,02 810

20,04 391 20,04 239 20,04 242 20,04 129 20,04 884

20,06 372 20,06 283 20,06 265 20,06 131 20,06 910

20,08 354 20,08 239 20,08 258 20,08 133 20,08 926

20,1 239 20,1 239 20,1 284 20,1 120 20,1 940

20,12 239 20,12 228 20,12 298 20,12 132 20,12 860

20,14 219 20,14 214 20,14 255 20,14 127 20,14 911

20,16 175 20,16 200 20,16 199 20,16 147 20,16 936

20,18 207 20,18 192 20,18 203 20,18 133 20,18 951

20,2 201 20,2 190 20,2 167 20,2 141 20,2 941

20,22 202 20,22 189 20,22 171 20,22 141 20,22 998

20,24 192 20,24 204 20,24 161 20,24 140 20,24 996

20,26 186 20,26 187 20,26 159 20,26 134 20,26 1069

20,28 232 20,28 177 20,28 153 20,28 130 20,28 1018

20,3 235 20,3 168 20,3 173 20,3 119 20,3 1035

20,32 210 20,32 205 20,32 158 20,32 145 20,32 1033

20,34 235 20,34 187 20,34 180 20,34 160 20,34 1091

20,36 195 20,36 200 20,36 184 20,36 124 20,36 1116

20,38 166 20,38 169 20,38 167 20,38 178 20,38 1069

20,4 149 20,4 178 20,4 169 20,4 133 20,4 1041

20,42 115 20,42 146 20,42 160 20,42 135 20,42 1051

20,44 130 20,44 155 20,44 115 20,44 163 20,44 1044

20,46 120 20,46 139 20,46 138 20,46 150 20,46 970

20,48 129 20,48 151 20,48 144 20,48 167 20,48 983

20,5 125 20,5 152 20,5 117 20,5 152 20,5 963

20,52 111 20,52 127 20,52 108 20,52 158 20,52 949

20,54 120 20,54 136 20,54 107 20,54 172 20,54 833

20,56 106 20,56 136 20,56 96 20,56 145 20,56 862

20,58 116 20,58 153 20,58 90 20,58 155 20,58 853

20,6 111 20,6 133 20,6 80 20,6 153 20,6 872

20,62 94 20,62 136 20,62 87 20,62 152 20,62 884

20,64 118 20,64 135 20,64 91 20,64 153 20,64 883

20,66 110 20,66 139 20,66 78 20,66 148 20,66 867

20,68 103 20,68 134 20,68 90 20,68 157 20,68 864

20,7 114 20,7 143 20,7 93 20,7 166 20,7 819

20,72 109 20,72 129 20,72 80 20,72 145 20,72 858

20,74 104 20,74 114 20,74 86 20,74 150 20,74 836

20,76 124 20,76 152 20,76 91 20,76 138 20,76 830

20,78 114 20,78 156 20,78 92 20,78 138 20,78 888

20,8 131 20,8 134 20,8 81 20,8 140 20,8 843

20,82 136 20,82 125 20,82 75 20,82 141 20,82 862

20,84 119 20,84 155 20,84 79 20,84 165 20,84 827

20,86 127 20,86 135 20,86 71 20,86 143 20,86 837

20,88 115 20,88 137 20,88 83 20,88 145 20,88 790

20,9 113 20,9 154 20,9 91 20,9 128 20,9 822

20,92 116 20,92 132 20,92 93 20,92 134 20,92 871

20,94 113 20,94 144 20,94 84 20,94 130 20,94 819

20,96 109 20,96 143 20,96 99 20,96 135 20,96 834

20,98 100 20,98 150 20,98 102 20,98 163 20,98 865

21 112 21 137 21 73 21 141 21 869

21,02 137 21,02 150 21,02 75 21,02 148 21,02 849

21,04 101 21,04 136 21,04 68 21,04 149 21,04 822

21,06 113 21,06 152 21,06 74 21,06 168 21,06 801

21,08 116 21,08 146 21,08 88 21,08 155 21,08 839

21,1 106 21,1 145 21,1 74 21,1 149 21,1 829

21,12 116 21,12 138 21,12 86 21,12 131 21,12 851

21,14 117 21,14 139 21,14 78 21,14 141 21,14 856

21,16 132 21,16 150 21,16 85 21,16 126 21,16 876

21,18 158 21,18 152 21,18 74 21,18 132 21,18 842

21,2 153 21,2 159 21,2 71 21,2 137 21,2 813

21,22 177 21,22 187 21,22 111 21,22 142 21,22 840

21,24 178 21,24 199 21,24 105 21,24 130 21,24 865

21,26 225 21,26 245 21,26 115 21,26 144 21,26 874

21,28 259 21,28 274 21,28 116 21,28 139 21,28 841

21,3 287 21,3 230 21,3 144 21,3 135 21,3 839

21,32 365 21,32 262 21,32 141 21,32 141 21,32 832

21,34 435 21,34 275 21,34 180 21,34 110 21,34 849

21,36 535 21,36 281 21,36 208 21,36 138 21,36 862

21,38 619 21,38 293 21,38 229 21,38 157 21,38 862

21,4 787 21,4 314 21,4 230 21,4 125 21,4 869

21,42 795 21,42 346 21,42 277 21,42 132 21,42 861

21,44 734 21,44 377 21,44 347 21,44 147 21,44 916

21,46 586 21,46 378 21,46 376 21,46 149 21,46 864

21,48 444 21,48 383 21,48 442 21,48 149 21,48 867

21,5 375 21,5 312 21,5 406 21,5 130 21,5 895

21,52 269 21,52 286 21,52 370 21,52 135 21,52 896

21,54 211 21,54 211 21,54 308 21,54 134 21,54 841

21,56 179 21,56 226 21,56 288 21,56 161 21,56 867

21,58 175 21,58 170 21,58 270 21,58 149 21,58 853

21,6 188 21,6 173 21,6 229 21,6 148 21,6 854

21,62 226 21,62 174 21,62 235 21,62 131 21,62 870

21,64 204 21,64 186 21,64 217 21,64 149 21,64 855

21,66 258 21,66 192 21,66 237 21,66 147 21,66 856

21,68 235 21,68 186 21,68 228 21,68 169 21,68 816

21,7 240 21,7 184 21,7 266 21,7 139 21,7 858

21,72 217 21,72 192 21,72 311 21,72 144 21,72 889

21,74 218 21,74 198 21,74 328 21,74 163 21,74 841

21,76 178 21,76 185 21,76 285 21,76 138 21,76 837

21,78 180 21,78 177 21,78 268 21,78 136 21,78 869

21,8 178 21,8 183 21,8 225 21,8 158 21,8 858

21,82 157 21,82 170 21,82 210 21,82 143 21,82 875

21,84 186 21,84 167 21,84 188 21,84 156 21,84 894

21,86 158 21,86 165 21,86 153 21,86 143 21,86 868

21,88 168 21,88 146 21,88 141 21,88 157 21,88 870

21,9 149 21,9 170 21,9 129 21,9 132 21,9 903

21,92 169 21,92 168 21,92 156 21,92 122 21,92 910

21,94 164 21,94 169 21,94 122 21,94 152 21,94 838

21,96 143 21,96 175 21,96 123 21,96 120 21,96 873

21,98 155 21,98 158 21,98 123 21,98 143 21,98 821

22 135 22 163 22 122 22 126 22 827

22,02 140 22,02 164 22,02 132 22,02 125 22,02 916

22,04 149 22,04 137 22,04 122 22,04 150 22,04 903

22,06 138 22,06 162 22,06 114 22,06 152 22,06 894

22,08 169 22,08 194 22,08 113 22,08 149 22,08 884

22,1 189 22,1 171 22,1 110 22,1 163 22,1 814

22,12 206 22,12 177 22,12 118 22,12 137 22,12 866

22,14 196 22,14 188 22,14 114 22,14 139 22,14 928

22,16 240 22,16 185 22,16 111 22,16 156 22,16 872

22,18 285 22,18 215 22,18 141 22,18 157 22,18 862

22,2 282 22,2 207 22,2 157 22,2 151 22,2 858

22,22 330 22,22 213 22,22 165 22,22 139 22,22 854

22,24 324 22,24 202 22,24 172 22,24 159 22,24 877

22,26 315 22,26 255 22,26 148 22,26 163 22,26 885

22,28 261 22,28 217 22,28 217 22,28 163 22,28 878

22,3 209 22,3 212 22,3 186 22,3 186 22,3 857

22,32 199 22,32 209 22,32 190 22,32 185 22,32 878

22,34 170 22,34 209 22,34 198 22,34 181 22,34 855

22,36 145 22,36 181 22,36 191 22,36 183 22,36 829

22,38 134 22,38 218 22,38 176 22,38 198 22,38 820

22,4 132 22,4 188 22,4 156 22,4 199 22,4 881

22,42 141 22,42 151 22,42 146 22,42 223 22,42 874

22,44 124 22,44 137 22,44 129 22,44 189 22,44 902

22,46 121 22,46 165 22,46 126 22,46 193 22,46 865

22,48 146 22,48 141 22,48 98 22,48 159 22,48 885

22,5 107 22,5 141 22,5 91 22,5 188 22,5 906

22,52 143 22,52 141 22,52 86 22,52 162 22,52 892

22,54 124 22,54 148 22,54 86 22,54 153 22,54 876

22,56 132 22,56 162 22,56 88 22,56 149 22,56 853

22,58 114 22,58 153 22,58 79 22,58 161 22,58 804

22,6 133 22,6 145 22,6 82 22,6 154 22,6 803

22,62 126 22,62 143 22,62 90 22,62 156 22,62 908

22,64 126 22,64 164 22,64 91 22,64 149 22,64 924

22,66 147 22,66 159 22,66 90 22,66 148 22,66 888

22,68 173 22,68 140 22,68 94 22,68 131 22,68 846

22,7 138 22,7 145 22,7 92 22,7 155 22,7 888

22,72 147 22,72 151 22,72 92 22,72 135 22,72 863

22,74 150 22,74 166 22,74 99 22,74 121 22,74 823

22,76 149 22,76 154 22,76 104 22,76 147 22,76 900

22,78 150 22,78 157 22,78 95 22,78 134 22,78 861

22,8 161 22,8 176 22,8 106 22,8 140 22,8 871

22,82 182 22,82 164 22,82 134 22,82 163 22,82 846

22,84 138 22,84 189 22,84 118 22,84 140 22,84 867

22,86 162 22,86 199 22,86 113 22,86 137 22,86 850

22,88 159 22,88 154 22,88 124 22,88 140 22,88 875

22,9 143 22,9 168 22,9 119 22,9 144 22,9 860

22,92 160 22,92 174 22,92 131 22,92 155 22,92 854

22,94 160 22,94 175 22,94 105 22,94 156 22,94 918

22,96 134 22,96 171 22,96 121 22,96 136 22,96 920

22,98 170 22,98 172 22,98 114 22,98 136 22,98 943

23 164 23 186 23 140 23 166 23 858

23,02 188 23,02 169 23,02 135 23,02 124 23,02 906

23,04 183 23,04 183 23,04 133 23,04 139 23,04 924

23,06 221 23,06 186 23,06 137 23,06 155 23,06 869

23,08 187 23,08 181 23,08 121 23,08 149 23,08 882

23,1 166 23,1 203 23,1 125 23,1 159 23,1 858

23,12 149 23,12 185 23,12 131 23,12 153 23,12 864

23,14 180 23,14 180 23,14 137 23,14 146 23,14 896

23,16 186 23,16 185 23,16 141 23,16 153 23,16 902

23,18 161 23,18 178 23,18 109 23,18 163 23,18 886

23,2 217 23,2 181 23,2 119 23,2 141 23,2 825

23,22 215 23,22 227 23,22 110 23,22 145 23,22 887

23,24 250 23,24 209 23,24 106 23,24 162 23,24 933

23,26 289 23,26 250 23,26 97 23,26 152 23,26 879

23,28 332 23,28 228 23,28 148 23,28 148 23,28 953

23,3 404 23,3 251 23,3 136 23,3 150 23,3 885

23,32 489 23,32 263 23,32 146 23,32 136 23,32 854

23,34 588 23,34 264 23,34 154 23,34 141 23,34 917

23,36 738 23,36 288 23,36 190 23,36 147 23,36 914

23,38 862 23,38 297 23,38 191 23,38 143 23,38 932

23,4 985 23,4 270 23,4 209 23,4 145 23,4 860

23,42 958 23,42 385 23,42 284 23,42 169 23,42 918

23,44 864 23,44 388 23,44 304 23,44 145 23,44 950

23,46 697 23,46 432 23,46 326 23,46 138 23,46 900

23,48 542 23,48 453 23,48 400 23,48 155 23,48 973

23,5 411 23,5 478 23,5 433 23,5 149 23,5 905

23,52 264 23,52 375 23,52 444 23,52 150 23,52 927

23,54 202 23,54 306 23,54 387 23,54 143 23,54 942

23,56 150 23,56 264 23,56 383 23,56 150 23,56 929

23,58 136 23,58 216 23,58 289 23,58 134 23,58 901

23,6 132 23,6 202 23,6 237 23,6 135 23,6 896

23,62 143 23,62 169 23,62 222 23,62 144 23,62 942

23,64 129 23,64 147 23,64 160 23,64 157 23,64 894

23,66 108 23,66 155 23,66 142 23,66 152 23,66 934

23,68 98 23,68 130 23,68 132 23,68 169 23,68 976

23,7 99 23,7 130 23,7 114 23,7 152 23,7 939

23,72 112 23,72 152 23,72 111 23,72 138 23,72 908

23,74 86 23,74 177 23,74 102 23,74 140 23,74 900

23,76 112 23,76 159 23,76 105 23,76 164 23,76 908

23,78 92 23,78 157 23,78 104 23,78 153 23,78 912

23,8 111 23,8 172 23,8 92 23,8 137 23,8 907

23,82 104 23,82 178 23,82 65 23,82 151 23,82 927

23,84 107 23,84 147 23,84 83 23,84 153 23,84 905

23,86 111 23,86 159 23,86 102 23,86 142 23,86 906

23,88 118 23,88 153 23,88 98 23,88 153 23,88 945

23,9 113 23,9 143 23,9 94 23,9 160 23,9 939

23,92 138 23,92 140 23,92 95 23,92 153 23,92 914

23,94 113 23,94 159 23,94 84 23,94 176 23,94 903

23,96 116 23,96 137 23,96 80 23,96 147 23,96 926

23,98 135 23,98 137 23,98 92 23,98 151 23,98 889

24 138 24 145 24 93 24 130 24 922

24,02 120 24,02 154 24,02 99 24,02 151 24,02 955

24,04 179 24,04 160 24,04 88 24,04 158 24,04 988

24,06 169 24,06 151 24,06 82 24,06 166 24,06 921

24,08 152 24,08 134 24,08 102 24,08 138 24,08 903

24,1 151 24,1 152 24,1 102 24,1 146 24,1 978

24,12 154 24,12 181 24,12 101 24,12 158 24,12 919

24,14 152 24,14 149 24,14 114 24,14 156 24,14 905

24,16 149 24,16 154 24,16 110 24,16 152 24,16 881

24,18 132 24,18 158 24,18 119 24,18 163 24,18 915

24,2 129 24,2 148 24,2 105 24,2 148 24,2 915

24,22 144 24,22 186 24,22 137 24,22 152 24,22 916

24,24 114 24,24 185 24,24 128 24,24 166 24,24 949

24,26 147 24,26 150 24,26 114 24,26 163 24,26 974

24,28 151 24,28 158 24,28 141 24,28 153 24,28 939

24,3 152 24,3 171 24,3 130 24,3 135 24,3 989

24,32 141 24,32 152 24,32 128 24,32 162 24,32 976

24,34 169 24,34 162 24,34 125 24,34 155 24,34 1065

24,36 176 24,36 162 24,36 131 24,36 157 24,36 991

24,38 229 24,38 187 24,38 129 24,38 161 24,38 1010

24,4 215 24,4 199 24,4 107 24,4 144 24,4 1051

24,42 245 24,42 184 24,42 121 24,42 162 24,42 1034

24,44 274 24,44 199 24,44 147 24,44 155 24,44 1025

24,46 323 24,46 197 24,46 154 24,46 150 24,46 965

24,48 323 24,48 191 24,48 156 24,48 158 24,48 1019

24,5 311 24,5 204 24,5 171 24,5 151 24,5 1034

24,52 316 24,52 203 24,52 179 24,52 153 24,52 1064

24,54 249 24,54 177 24,54 168 24,54 149 24,54 992

24,56 254 24,56 183 24,56 167 24,56 154 24,56 1012

24,58 233 24,58 182 24,58 182 24,58 148 24,58 1031

24,6 199 24,6 184 24,6 176 24,6 148 24,6 948

24,62 168 24,62 184 24,62 161 24,62 163 24,62 985

24,64 150 24,64 174 24,64 172 24,64 165 24,64 1066

24,66 144 24,66 160 24,66 133 24,66 169 24,66 1036

24,68 132 24,68 184 24,68 117 24,68 167 24,68 1042

24,7 141 24,7 161 24,7 116 24,7 161 24,7 1030

24,72 126 24,72 162 24,72 129 24,72 148 24,72 986

24,74 133 24,74 154 24,74 108 24,74 135 24,74 993

24,76 164 24,76 163 24,76 100 24,76 161 24,76 1019

24,78 145 24,78 170 24,78 103 24,78 195 24,78 1085

24,8 162 24,8 174 24,8 118 24,8 152 24,8 1026

24,82 125 24,82 135 24,82 117 24,82 174 24,82 955

24,84 157 24,84 174 24,84 119 24,84 155 24,84 981

24,86 163 24,86 159 24,86 118 24,86 147 24,86 1078

24,88 170 24,88 163 24,88 111 24,88 147 24,88 1019

24,9 172 24,9 167 24,9 123 24,9 153 24,9 996

24,92 176 24,92 183 24,92 123 24,92 171 24,92 977

24,94 210 24,94 172 24,94 157 24,94 166 24,94 964

24,96 195 24,96 203 24,96 144 24,96 169 24,96 1014

24,98 214 24,98 210 24,98 149 24,98 168 24,98 1025

25 259 25 228 25 147 25 149 25 1008

25,02 292 25,02 267 25,02 183 25,02 162 25,02 998

25,04 378 25,04 193 25,04 175 25,04 170 25,04 952

25,06 434 25,06 227 25,06 183 25,06 172 25,06 983

25,08 480 25,08 233 25,08 188 25,08 185 25,08 1098

25,1 544 25,1 256 25,1 215 25,1 148 25,1 965

25,12 487 25,12 296 25,12 242 25,12 161 25,12 948

25,14 472 25,14 270 25,14 245 25,14 160 25,14 946

25,16 377 25,16 297 25,16 249 25,16 169 25,16 1006

25,18 320 25,18 258 25,18 249 25,18 160 25,18 991

25,2 239 25,2 278 25,2 257 25,2 172 25,2 954

25,22 208 25,22 233 25,22 257 25,22 154 25,22 1036

25,24 177 25,24 200 25,24 184 25,24 140 25,24 988

25,26 174 25,26 201 25,26 223 25,26 165 25,26 965

25,28 168 25,28 166 25,28 160 25,28 152 25,28 1016

25,3 151 25,3 171 25,3 163 25,3 145 25,3 1037

25,32 154 25,32 151 25,32 121 25,32 149 25,32 1069

25,34 120 25,34 140 25,34 146 25,34 162 25,34 985

25,36 126 25,36 149 25,36 158 25,36 162 25,36 1000

25,38 152 25,38 150 25,38 137 25,38 176 25,38 982

25,4 142 25,4 138 25,4 105 25,4 161 25,4 1026

25,42 137 25,42 151 25,42 111 25,42 166 25,42 956

25,44 150 25,44 145 25,44 121 25,44 163 25,44 1030

25,46 138 25,46 148 25,46 126 25,46 191 25,46 990

25,48 153 25,48 143 25,48 103 25,48 148 25,48 992

25,5 147 25,5 162 25,5 117 25,5 159 25,5 1010

25,52 170 25,52 169 25,52 106 25,52 152 25,52 956

25,54 156 25,54 163 25,54 102 25,54 155 25,54 1013

25,56 180 25,56 157 25,56 96 25,56 167 25,56 951

25,58 184 25,58 151 25,58 104 25,58 168 25,58 966

25,6 172 25,6 154 25,6 117 25,6 155 25,6 998

25,62 177 25,62 164 25,62 107 25,62 147 25,62 991

25,64 180 25,64 146 25,64 125 25,64 146 25,64 1022

25,66 178 25,66 154 25,66 128 25,66 159 25,66 1002

25,68 171 25,68 167 25,68 112 25,68 170 25,68 1061

25,7 141 25,7 146 25,7 116 25,7 152 25,7 1024

25,72 142 25,72 162 25,72 96 25,72 141 25,72 1007

25,74 160 25,74 140 25,74 103 25,74 179 25,74 978

25,76 158 25,76 171 25,76 97 25,76 156 25,76 968

25,78 160 25,78 165 25,78 102 25,78 154 25,78 998

25,8 136 25,8 159 25,8 96 25,8 176 25,8 1044

25,82 133 25,82 127 25,82 99 25,82 175 25,82 969

25,84 138 25,84 143 25,84 109 25,84 183 25,84 1057

25,86 116 25,86 150 25,86 96 25,86 159 25,86 1026

25,88 129 25,88 149 25,88 86 25,88 146 25,88 993

25,9 138 25,9 135 25,9 96 25,9 157 25,9 978

25,92 120 25,92 140 25,92 86 25,92 159 25,92 1034

25,94 123 25,94 131 25,94 91 25,94 185 25,94 1006

25,96 137 25,96 161 25,96 83 25,96 166 25,96 1079

25,98 121 25,98 161 25,98 81 25,98 177 25,98 1070

26 134 26 136 26 82 26 184 26 1027

26,02 162 26,02 167 26,02 90 26,02 172 26,02 1021

26,04 172 26,04 152 26,04 87 26,04 149 26,04 1008

26,06 168 26,06 161 26,06 82 26,06 164 26,06 985

26,08 170 26,08 148 26,08 85 26,08 175 26,08 1016

26,1 152 26,1 165 26,1 101 26,1 190 26,1 1014

26,12 142 26,12 147 26,12 89 26,12 168 26,12 1036

26,14 160 26,14 155 26,14 114 26,14 161 26,14 1000

26,16 137 26,16 175 26,16 68 26,16 206 26,16 1048

26,18 153 26,18 137 26,18 85 26,18 164 26,18 1007

26,2 137 26,2 152 26,2 84 26,2 162 26,2 994

26,22 169 26,22 160 26,22 96 26,22 171 26,22 1079

26,24 209 26,24 175 26,24 72 26,24 203 26,24 1130

26,26 200 26,26 160 26,26 81 26,26 191 26,26 1039

26,28 214 26,28 174 26,28 90 26,28 200 26,28 1016

26,3 236 26,3 197 26,3 101 26,3 176 26,3 1087

26,32 266 26,32 188 26,32 110 26,32 194 26,32 1089

26,34 283 26,34 187 26,34 102 26,34 214 26,34 1041

26,36 322 26,36 207 26,36 118 26,36 191 26,36 1134

26,38 290 26,38 182 26,38 142 26,38 208 26,38 1124

26,4 272 26,4 217 26,4 156 26,4 233 26,4 1092

26,42 228 26,42 206 26,42 167 26,42 231 26,42 1080

26,44 239 26,44 220 26,44 192 26,44 263 26,44 1046

26,46 204 26,46 206 26,46 201 26,46 250 26,46 997

26,48 177 26,48 195 26,48 206 26,48 256 26,48 1107

26,5 185 26,5 187 26,5 198 26,5 290 26,5 996

26,52 150 26,52 197 26,52 179 26,52 265 26,52 1048

26,54 163 26,54 164 26,54 136 26,54 237 26,54 999

26,56 152 26,56 150 26,56 154 26,56 261 26,56 1057

26,58 157 26,58 151 26,58 126 26,58 237 26,58 1069

26,6 121 26,6 171 26,6 116 26,6 250 26,6 1103

26,62 154 26,62 162 26,62 119 26,62 214 26,62 1071

26,64 133 26,64 151 26,64 115 26,64 232 26,64 1087

26,66 136 26,66 163 26,66 101 26,66 212 26,66 1149

26,68 132 26,68 149 26,68 98 26,68 232 26,68 1125

26,7 130 26,7 154 26,7 110 26,7 221 26,7 1113

26,72 154 26,72 148 26,72 84 26,72 225 26,72 1088

26,74 131 26,74 151 26,74 91 26,74 182 26,74 1158

26,76 142 26,76 161 26,76 82 26,76 189 26,76 1143

26,78 129 26,78 182 26,78 74 26,78 202 26,78 1106

26,8 132 26,8 153 26,8 81 26,8 219 26,8 1095

26,82 152 26,82 148 26,82 66 26,82 227 26,82 1066

26,84 113 26,84 116 26,84 84 26,84 214 26,84 1061

26,86 111 26,86 142 26,86 80 26,86 227 26,86 1046

26,88 116 26,88 142 26,88 89 26,88 225 26,88 1105

26,9 119 26,9 129 26,9 106 26,9 235 26,9 1093

26,92 106 26,92 170 26,92 91 26,92 227 26,92 1060

26,94 98 26,94 127 26,94 79 26,94 245 26,94 1016

26,96 107 26,96 118 26,96 79 26,96 239 26,96 1115

26,98 106 26,98 134 26,98 91 26,98 252 26,98 1101

27 95 27 148 27 76 27 266 27 1055

27,02 99 27,02 133 27,02 80 27,02 263 27,02 1033

27,04 112 27,04 140 27,04 85 27,04 200 27,04 1062

27,06 100 27,06 125 27,06 88 27,06 238 27,06 1033

27,08 110 27,08 125 27,08 76 27,08 243 27,08 1061

27,1 132 27,1 139 27,1 68 27,1 212 27,1 1008

27,12 104 27,12 135 27,12 89 27,12 176 27,12 1008

27,14 132 27,14 126 27,14 76 27,14 173 27,14 1024

27,16 100 27,16 123 27,16 91 27,16 196 27,16 1006

27,18 136 27,18 138 27,18 95 27,18 190 27,18 1085

27,2 142 27,2 136 27,2 86 27,2 192 27,2 1092

27,22 139 27,22 149 27,22 101 27,22 173 27,22 1034

27,24 112 27,24 136 27,24 101 27,24 174 27,24 1071

27,26 131 27,26 140 27,26 92 27,26 173 27,26 1052

27,28 119 27,28 147 27,28 113 27,28 177 27,28 1052

27,3 142 27,3 159 27,3 123 27,3 198 27,3 1050

27,32 136 27,32 147 27,32 123 27,32 169 27,32 1047

27,34 131 27,34 149 27,34 110 27,34 186 27,34 1019

27,36 148 27,36 147 27,36 127 27,36 195 27,36 997

27,38 160 27,38 148 27,38 163 27,38 181 27,38 1063

27,4 158 27,4 154 27,4 113 27,4 190 27,4 1072

27,42 162 27,42 122 27,42 125 27,42 176 27,42 1026

27,44 183 27,44 161 27,44 151 27,44 172 27,44 1026

27,46 181 27,46 155 27,46 168 27,46 198 27,46 1056

27,48 193 27,48 165 27,48 181 27,48 190 27,48 1031

27,5 209 27,5 188 27,5 143 27,5 192 27,5 1045

27,52 223 27,52 170 27,52 174 27,52 179 27,52 1006

27,54 204 27,54 167 27,54 172 27,54 176 27,54 1031

27,56 164 27,56 161 27,56 155 27,56 189 27,56 1023

27,58 158 27,58 190 27,58 170 27,58 168 27,58 1017

27,6 153 27,6 150 27,6 157 27,6 169 27,6 976

27,62 136 27,62 165 27,62 179 27,62 184 27,62 960

27,64 165 27,64 162 27,64 163 27,64 184 27,64 1030

27,66 127 27,66 166 27,66 177 27,66 175 27,66 989

27,68 128 27,68 154 27,68 151 27,68 185 27,68 1061

27,7 143 27,7 148 27,7 188 27,7 180 27,7 997

27,72 140 27,72 148 27,72 144 27,72 169 27,72 1091

27,74 134 27,74 163 27,74 140 27,74 171 27,74 945

27,76 137 27,76 161 27,76 147 27,76 170 27,76 1006

27,78 126 27,78 161 27,78 141 27,78 189 27,78 1069

27,8 145 27,8 160 27,8 138 27,8 185 27,8 959

27,82 142 27,82 167 27,82 129 27,82 194 27,82 1046

27,84 132 27,84 148 27,84 136 27,84 232 27,84 1034

27,86 165 27,86 183 27,86 118 27,86 221 27,86 960

27,88 137 27,88 149 27,88 137 27,88 224 27,88 1049

27,9 164 27,9 156 27,9 120 27,9 190 27,9 999

27,92 164 27,92 167 27,92 125 27,92 226 27,92 1038

27,94 168 27,94 155 27,94 134 27,94 209 27,94 1000

27,96 158 27,96 175 27,96 119 27,96 187 27,96 1050

27,98 168 27,98 167 27,98 131 27,98 214 27,98 1023

28 189 28 157 28 148 28 181 28 1053

28,02 165 28,02 165 28,02 139 28,02 210 28,02 1034

28,04 154 28,04 149 28,04 170 28,04 203 28,04 1047

28,06 160 28,06 157 28,06 169 28,06 189 28,06 1063

28,08 166 28,08 154 28,08 143 28,08 221 28,08 1064

28,1 152 28,1 162 28,1 145 28,1 230 28,1 1067

28,12 176 28,12 177 28,12 151 28,12 226 28,12 1103

28,14 155 28,14 139 28,14 137 28,14 222 28,14 1034

28,16 158 28,16 146 28,16 129 28,16 222 28,16 1176

28,18 165 28,18 155 28,18 145 28,18 210 28,18 1136

28,2 186 28,2 144 28,2 123 28,2 208 28,2 1195

28,22 166 28,22 137 28,22 137 28,22 165 28,22 1262

28,24 168 28,24 137 28,24 98 28,24 198 28,24 1286

28,26 166 28,26 161 28,26 148 28,26 207 28,26 1318

28,28 162 28,28 149 28,28 130 28,28 225 28,28 1293

28,3 147 28,3 122 28,3 129 28,3 203 28,3 1433

28,32 163 28,32 157 28,32 126 28,32 167 28,32 1443

28,34 171 28,34 152 28,34 130 28,34 198 28,34 1553

28,36 140 28,36 146 28,36 126 28,36 214 28,36 1611

28,38 144 28,38 144 28,38 124 28,38 215 28,38 1723

28,4 168 28,4 135 28,4 153 28,4 209 28,4 1775

28,42 165 28,42 138 28,42 137 28,42 195 28,42 1799

28,44 178 28,44 161 28,44 118 28,44 224 28,44 1763

28,46 178 28,46 178 28,46 134 28,46 197 28,46 1832

28,48 210 28,48 178 28,48 129 28,48 234 28,48 1925

28,5 237 28,5 171 28,5 143 28,5 230 28,5 1982

28,52 279 28,52 188 28,52 147 28,52 263 28,52 1979

28,54 318 28,54 185 28,54 141 28,54 244 28,54 2053

28,56 348 28,56 172 28,56 172 28,56 216 28,56 2084

28,58 417 28,58 186 28,58 143 28,58 275 28,58 2081

28,6 441 28,6 217 28,6 169 28,6 241 28,6 2036

28,62 397 28,62 232 28,62 186 28,62 227 28,62 1994

28,64 394 28,64 226 28,64 183 28,64 241 28,64 1818

28,66 302 28,66 260 28,66 194 28,66 199 28,66 1779

28,68 274 28,68 263 28,68 251 28,68 248 28,68 1713

28,7 258 28,7 227 28,7 246 28,7 213 28,7 1681

28,72 188 28,72 235 28,72 285 28,72 221 28,72 1638

28,74 170 28,74 216 28,74 261 28,74 203 28,74 1529

28,76 138 28,76 200 28,76 233 28,76 215 28,76 1469

28,78 134 28,78 191 28,78 225 28,78 226 28,78 1416

28,8 128 28,8 140 28,8 204 28,8 243 28,8 1344

28,82 146 28,82 163 28,82 198 28,82 224 28,82 1346

28,84 155 28,84 169 28,84 185 28,84 212 28,84 1333

28,86 155 28,86 139 28,86 147 28,86 233 28,86 1415

28,88 129 28,88 130 28,88 155 28,88 200 28,88 1319

28,9 149 28,9 166 28,9 143 28,9 188 28,9 1346

28,92 186 28,92 143 28,92 162 28,92 184 28,92 1316

28,94 154 28,94 200 28,94 149 28,94 175 28,94 1268

28,96 140 28,96 160 28,96 157 28,96 211 28,96 1413

28,98 152 28,98 139 28,98 130 28,98 202 28,98 1365

29 137 29 159 29 159 29 198 29 1356

29,02 151 29,02 141 29,02 159 29,02 209 29,02 1475

29,04 152 29,04 139 29,04 139 29,04 182 29,04 1489

29,06 127 29,06 158 29,06 149 29,06 236 29,06 1430

29,08 132 29,08 148 29,08 160 29,08 218 29,08 1607

29,1 93 29,1 145 29,1 158 29,1 213 29,1 1553

29,12 110 29,12 147 29,12 180 29,12 220 29,12 1534

29,14 122 29,14 149 29,14 168 29,14 217 29,14 1482

29,16 118 29,16 133 29,16 114 29,16 218 29,16 1503

29,18 103 29,18 125 29,18 129 29,18 209 29,18 1542

29,2 119 29,2 127 29,2 135 29,2 218 29,2 1460

29,22 96 29,22 131 29,22 143 29,22 210 29,22 1394

29,24 87 29,24 133 29,24 101 29,24 190 29,24 1370

29,26 98 29,26 128 29,26 96 29,26 208 29,26 1326

29,28 106 29,28 113 29,28 110 29,28 193 29,28 1288

29,3 112 29,3 119 29,3 98 29,3 213 29,3 1306

29,32 103 29,32 133 29,32 91 29,32 202 29,32 1249

29,34 107 29,34 133 29,34 93 29,34 190 29,34 1286

29,36 130 29,36 120 29,36 112 29,36 203 29,36 1253

29,38 150 29,38 132 29,38 76 29,38 207 29,38 1197

29,4 153 29,4 134 29,4 77 29,4 185 29,4 1156

29,42 180 29,42 156 29,42 90 29,42 186 29,42 1108

29,44 187 29,44 137 29,44 84 29,44 176 29,44 1084

29,46 193 29,46 171 29,46 100 29,46 209 29,46 1124

29,48 214 29,48 158 29,48 99 29,48 181 29,48 1191

29,5 216 29,5 153 29,5 117 29,5 165 29,5 1090

29,52 236 29,52 141 29,52 121 29,52 165 29,52 1066

29,54 270 29,54 141 29,54 109 29,54 173 29,54 1102

29,56 239 29,56 158 29,56 122 29,56 195 29,56 1084

29,58 226 29,58 173 29,58 115 29,58 196 29,58 1093

29,6 180 29,6 174 29,6 132 29,6 156 29,6 1025

29,62 238 29,62 187 29,62 129 29,62 176 29,62 1085

29,64 166 29,64 155 29,64 145 29,64 179 29,64 1053

29,66 173 29,66 188 29,66 149 29,66 189 29,66 1065

29,68 142 29,68 166 29,68 123 29,68 175 29,68 1065

29,7 153 29,7 135 29,7 139 29,7 185 29,7 1016

29,72 154 29,72 160 29,72 138 29,72 169 29,72 1089

29,74 137 29,74 141 29,74 117 29,74 184 29,74 1112

29,76 153 29,76 171 29,76 142 29,76 200 29,76 1108

29,78 133 29,78 134 29,78 138 29,78 217 29,78 1095

29,8 143 29,8 134 29,8 140 29,8 204 29,8 1151

29,82 136 29,82 136 29,82 118 29,82 220 29,82 1154

29,84 131 29,84 126 29,84 118 29,84 209 29,84 1166

29,86 120 29,86 133 29,86 109 29,86 207 29,86 1165

29,88 112 29,88 148 29,88 97 29,88 226 29,88 1221

29,9 147 29,9 122 29,9 96 29,9 192 29,9 1224

29,92 125 29,92 107 29,92 100 29,92 213 29,92 1234

29,94 129 29,94 128 29,94 104 29,94 205 29,94 1317

29,96 146 29,96 127 29,96 112 29,96 212 29,96 1325

29,98 151 29,98 146 29,98 110 29,98 201 29,98 1309

30 144 30 152 30 85 30 205 30 1342

| La@Eu 5% | | La@Sm 5% | | La@Dy 5% | | La@Tb 5% | |
| --- | --- | --- | --- | --- | --- | --- | --- |
| 2θ | I (A.U) | 2θ | I (A.U) | 2θ | I (A.U) | 2θ | I (A.U) |

3 563 3 448 3 517 3 533

3,02 553 3,02 485 3,02 527 3,02 546

3,04 498 3,04 488 3,04 488 3,04 570

3,06 520 3,06 496 3,06 458 3,06 557

3,08 524 3,08 518 3,08 472 3,08 556

3,1 492 3,1 499 3,1 463 3,1 575

3,12 507 3,12 493 3,12 458 3,12 583

3,14 525 3,14 498 3,14 479 3,14 600

3,16 535 3,16 467 3,16 481 3,16 603

3,18 494 3,18 515 3,18 494 3,18 593

3,2 466 3,2 500 3,2 484 3,2 612

3,22 492 3,22 513 3,22 481 3,22 583

3,24 491 3,24 496 3,24 468 3,24 580

3,26 1583 3,26 531 3,26 459 3,26 536

3,28 817 3,28 488 3,28 459 3,28 591

3,3 468 3,3 561 3,3 470 3,3 602

3,32 530 3,32 584 3,32 473 3,32 574

3,34 511 3,34 560 3,34 427 3,34 607

3,36 501 3,36 559 3,36 452 3,36 665

3,38 580 3,38 555 3,38 450 3,38 662

3,4 563 3,4 575 3,4 461 3,4 699

3,42 674 3,42 645 3,42 486 3,42 738

3,44 733 3,44 785 3,44 540 3,44 744

3,46 719 3,46 689 3,46 451 3,46 839

3,48 674 3,48 814 3,48 504 3,48 894

3,5 640 3,5 1038 3,5 549 3,5 1048

3,52 635 3,52 1443 3,52 550 3,52 1333

3,54 647 3,54 1687 3,54 537 3,54 1900

3,56 860 3,56 1591 3,56 677 3,56 2709

3,58 1145 3,58 1482 3,58 868 3,58 3817

3,6 1544 3,6 1731 3,6 705 3,6 4314

3,62 2393 3,62 1865 3,62 1016 3,62 4097

3,64 3519 3,64 2344 3,64 1492 3,64 2876

3,66 3949 3,66 3529 3,66 1555 3,66 1856

3,68 2177 3,68 5119 3,68 885 3,68 1691

3,7 734 3,7 6018 3,7 495 3,7 1487

3,72 488 3,72 5360 3,72 483 3,72 837

3,74 469 3,74 3701 3,74 456 3,74 529

3,76 424 3,76 1945 3,76 418 3,76 426

3,78 445 3,78 791 3,78 411 3,78 413

3,8 385 3,8 555 3,8 400 3,8 427

3,82 381 3,82 502 3,82 356 3,82 409

3,84 422 3,84 446 3,84 401 3,84 407

3,86 371 3,86 431 3,86 389 3,86 386

3,88 402 3,88 434 3,88 327 3,88 398

3,9 370 3,9 375 3,9 360 3,9 401

3,92 366 3,92 369 3,92 362 3,92 363

3,94 372 3,94 338 3,94 338 3,94 352

3,96 343 3,96 338 3,96 360 3,96 373

3,98 356 3,98 352 3,98 382 3,98 389

4 345 4 353 4 324 4 358

4,02 349 4,02 328 4,02 327 4,02 388

4,04 349 4,04 327 4,04 362 4,04 387

4,06 343 4,06 349 4,06 330 4,06 379

4,08 380 4,08 338 4,08 326 4,08 336

4,1 392 4,1 307 4,1 353 4,1 308

4,12 342 4,12 332 4,12 324 4,12 329

4,14 334 4,14 297 4,14 346 4,14 342

4,16 348 4,16 295 4,16 310 4,16 338

4,18 313 4,18 281 4,18 354 4,18 356

4,2 339 4,2 309 4,2 305 4,2 321

4,22 322 4,22 291 4,22 300 4,22 341

4,24 302 4,24 329 4,24 345 4,24 316

4,26 308 4,26 350 4,26 304 4,26 329

4,28 316 4,28 269 4,28 311 4,28 320

4,3 305 4,3 316 4,3 327 4,3 325

4,32 333 4,32 320 4,32 325 4,32 321

4,34 327 4,34 284 4,34 298 4,34 345

4,36 306 4,36 296 4,36 313 4,36 295

4,38 311 4,38 313 4,38 316 4,38 303

4,4 322 4,4 282 4,4 312 4,4 301

4,42 336 4,42 291 4,42 308 4,42 311

4,44 305 4,44 282 4,44 338 4,44 300

4,46 327 4,46 317 4,46 300 4,46 292

4,48 300 4,48 291 4,48 286 4,48 300

4,5 324 4,5 296 4,5 306 4,5 274

4,52 296 4,52 285 4,52 297 4,52 308

4,54 320 4,54 291 4,54 289 4,54 286

4,56 299 4,56 267 4,56 313 4,56 309

4,58 317 4,58 288 4,58 337 4,58 303

4,6 304 4,6 275 4,6 323 4,6 271

4,62 293 4,62 257 4,62 304 4,62 302

4,64 309 4,64 290 4,64 287 4,64 311

4,66 292 4,66 289 4,66 279 4,66 298

4,68 270 4,68 265 4,68 288 4,68 285

4,7 275 4,7 271 4,7 295 4,7 256

4,72 279 4,72 268 4,72 315 4,72 293

4,74 277 4,74 237 4,74 281 4,74 300

4,76 274 4,76 290 4,76 309 4,76 312

4,78 277 4,78 257 4,78 277 4,78 266

4,8 263 4,8 246 4,8 306 4,8 230

4,82 277 4,82 274 4,82 306 4,82 273

4,84 285 4,84 248 4,84 304 4,84 295

4,86 287 4,86 300 4,86 253 4,86 277

4,88 271 4,88 296 4,88 284 4,88 287

4,9 277 4,9 267 4,9 283 4,9 285

4,92 275 4,92 257 4,92 249 4,92 283

4,94 288 4,94 244 4,94 267 4,94 288

4,96 290 4,96 258 4,96 291 4,96 298

4,98 284 4,98 266 4,98 274 4,98 268

5 256 5 236 5 287 5 296

5,02 269 5,02 269 5,02 278 5,02 270

5,04 272 5,04 247 5,04 272 5,04 282

5,06 263 5,06 247 5,06 235 5,06 239

5,08 263 5,08 225 5,08 241 5,08 268

5,1 256 5,1 230 5,1 255 5,1 258

5,12 250 5,12 239 5,12 274 5,12 272

5,14 261 5,14 230 5,14 271 5,14 257

5,16 274 5,16 237 5,16 261 5,16 236

5,18 272 5,18 258 5,18 279 5,18 260

5,2 293 5,2 237 5,2 261 5,2 249

5,22 236 5,22 252 5,22 244 5,22 209

5,24 252 5,24 238 5,24 240 5,24 263

5,26 297 5,26 257 5,26 242 5,26 264

5,28 245 5,28 259 5,28 265 5,28 274

5,3 275 5,3 251 5,3 226 5,3 248

5,32 229 5,32 230 5,32 265 5,32 257

5,34 230 5,34 227 5,34 246 5,34 269

5,36 267 5,36 236 5,36 236 5,36 265

5,38 233 5,38 248 5,38 268 5,38 276

5,4 240 5,4 242 5,4 244 5,4 230

5,42 241 5,42 251 5,42 236 5,42 235

5,44 249 5,44 255 5,44 252 5,44 225

5,46 241 5,46 220 5,46 291 5,46 251

5,48 257 5,48 261 5,48 251 5,48 250

5,5 255 5,5 213 5,5 237 5,5 233

5,52 250 5,52 225 5,52 241 5,52 249

5,54 231 5,54 210 5,54 271 5,54 244

5,56 240 5,56 233 5,56 235 5,56 224

5,58 228 5,58 223 5,58 255 5,58 237

5,6 229 5,6 244 5,6 249 5,6 221

5,62 255 5,62 196 5,62 259 5,62 229

5,64 261 5,64 222 5,64 242 5,64 216

5,66 241 5,66 204 5,66 270 5,66 241

5,68 213 5,68 230 5,68 219 5,68 228

5,7 241 5,7 209 5,7 257 5,7 216

5,72 241 5,72 212 5,72 252 5,72 244

5,74 235 5,74 196 5,74 220 5,74 257

5,76 224 5,76 225 5,76 242 5,76 243

5,78 218 5,78 210 5,78 243 5,78 212

5,8 239 5,8 223 5,8 238 5,8 224

5,82 239 5,82 216 5,82 210 5,82 223

5,84 209 5,84 200 5,84 231 5,84 218

5,86 236 5,86 230 5,86 237 5,86 224

5,88 197 5,88 205 5,88 231 5,88 234

5,9 210 5,9 227 5,9 231 5,9 238

5,92 207 5,92 217 5,92 201 5,92 238

5,94 218 5,94 215 5,94 220 5,94 226

5,96 214 5,96 205 5,96 212 5,96 245

5,98 216 5,98 204 5,98 236 5,98 236

6 216 6 214 6 243 6 243

6,02 190 6,02 211 6,02 250 6,02 231

6,04 235 6,04 207 6,04 239 6,04 224

6,06 240 6,06 212 6,06 244 6,06 220

6,08 212 6,08 218 6,08 215 6,08 253

6,1 218 6,1 185 6,1 213 6,1 210

6,12 215 6,12 213 6,12 213 6,12 225

6,14 213 6,14 192 6,14 223 6,14 220

6,16 220 6,16 187 6,16 212 6,16 227

6,18 213 6,18 197 6,18 219 6,18 218

6,2 194 6,2 185 6,2 225 6,2 208

6,22 216 6,22 206 6,22 217 6,22 234

6,24 203 6,24 231 6,24 206 6,24 214

6,26 215 6,26 206 6,26 221 6,26 198

6,28 213 6,28 203 6,28 214 6,28 234

6,3 183 6,3 217 6,3 254 6,3 235

6,32 204 6,32 205 6,32 227 6,32 234

6,34 194 6,34 187 6,34 206 6,34 236

6,36 208 6,36 228 6,36 237 6,36 219

6,38 218 6,38 195 6,38 228 6,38 247

6,4 217 6,4 231 6,4 211 6,4 245

6,42 200 6,42 214 6,42 189 6,42 260

6,44 197 6,44 215 6,44 206 6,44 195

6,46 187 6,46 183 6,46 177 6,46 233

6,48 219 6,48 221 6,48 198 6,48 214

6,5 193 6,5 228 6,5 203 6,5 194

6,52 205 6,52 232 6,52 215 6,52 222

6,54 204 6,54 203 6,54 196 6,54 207

6,56 199 6,56 208 6,56 201 6,56 226

6,58 186 6,58 179 6,58 222 6,58 215

6,6 197 6,6 201 6,6 204 6,6 215

6,62 193 6,62 186 6,62 191 6,62 198

6,64 179 6,64 197 6,64 203 6,64 223

6,66 209 6,66 204 6,66 214 6,66 209

6,68 207 6,68 211 6,68 208 6,68 206

6,7 216 6,7 202 6,7 185 6,7 186

6,72 203 6,72 185 6,72 210 6,72 207

6,74 192 6,74 214 6,74 188 6,74 228

6,76 221 6,76 214 6,76 201 6,76 220

6,78 182 6,78 195 6,78 175 6,78 212

6,8 206 6,8 218 6,8 193 6,8 230

6,82 180 6,82 207 6,82 189 6,82 213

6,84 194 6,84 204 6,84 221 6,84 215

6,86 190 6,86 227 6,86 194 6,86 223

6,88 219 6,88 229 6,88 180 6,88 241

6,9 211 6,9 234 6,9 209 6,9 254

6,92 223 6,92 260 6,92 211 6,92 248

6,94 240 6,94 239 6,94 216 6,94 282

6,96 219 6,96 302 6,96 227 6,96 321

6,98 212 6,98 288 6,98 278 6,98 346

7 235 7 343 7 279 7 365

7,02 242 7,02 379 7,02 327 7,02 362

7,04 249 7,04 433 7,04 444 7,04 392

7,06 233 7,06 463 7,06 536 7,06 506

7,08 309 7,08 471 7,08 403 7,08 538

7,1 446 7,1 613 7,1 289 7,1 679

7,12 522 7,12 758 7,12 256 7,12 902

7,14 464 7,14 857 7,14 280 7,14 1337

7,16 428 7,16 885 7,16 348 7,16 1773

7,18 462 7,18 847 7,18 352 7,18 2310

7,2 713 7,2 927 7,2 451 7,2 2538

7,22 1401 7,22 1287 7,22 623 7,22 2369

7,24 2136 7,24 1946 7,24 1005 7,24 1590

7,26 2043 7,26 2706 7,26 1194 7,26 890

7,28 966 7,28 2979 7,28 941 7,28 547

7,3 342 7,3 2662 7,3 445 7,3 389

7,32 234 7,32 2340 7,32 237 7,32 279

7,34 201 7,34 1666 7,34 216 7,34 214

7,36 195 7,36 818 7,36 192 7,36 211

7,38 211 7,38 392 7,38 217 7,38 195

7,4 184 7,4 233 7,4 181 7,4 195

7,42 195 7,42 216 7,42 184 7,42 191

7,44 169 7,44 194 7,44 171 7,44 187

7,46 176 7,46 200 7,46 172 7,46 182

7,48 151 7,48 172 7,48 172 7,48 149

7,5 161 7,5 186 7,5 173 7,5 189

7,52 158 7,52 168 7,52 181 7,52 162

7,54 185 7,54 168 7,54 191 7,54 181

7,56 152 7,56 178 7,56 193 7,56 165

7,58 165 7,58 165 7,58 174 7,58 168

7,6 183 7,6 187 7,6 157 7,6 197

7,62 165 7,62 162 7,62 147 7,62 189

7,64 142 7,64 176 7,64 183 7,64 163

7,66 184 7,66 168 7,66 205 7,66 162

7,68 150 7,68 168 7,68 178 7,68 145

7,7 164 7,7 161 7,7 187 7,7 164

7,72 153 7,72 147 7,72 153 7,72 152

7,74 179 7,74 128 7,74 202 7,74 181

7,76 166 7,76 176 7,76 156 7,76 189

7,78 174 7,78 155 7,78 151 7,78 156

7,8 151 7,8 156 7,8 195 7,8 181

7,82 165 7,82 196 7,82 174 7,82 149

7,84 162 7,84 156 7,84 162 7,84 178

7,86 158 7,86 162 7,86 179 7,86 173

7,88 161 7,88 146 7,88 168 7,88 172

7,9 170 7,9 142 7,9 146 7,9 152

7,92 161 7,92 128 7,92 173 7,92 186

7,94 170 7,94 157 7,94 163 7,94 178

7,96 156 7,96 142 7,96 171 7,96 153

7,98 151 7,98 145 7,98 168 7,98 154

8 151 8 130 8 172 8 123

8,02 162 8,02 178 8,02 161 8,02 165

8,04 157 8,04 163 8,04 156 8,04 171

8,05 172 8,05 144 8,05 172 8,05 154

8,08 165 8,08 147 8,08 181 8,08 150

8,09 172 8,09 152 8,09 176 8,09 191

8,12 153 8,12 148 8,12 134 8,12 160

8,14 173 8,14 152 8,14 161 8,14 162

8,16 167 8,16 175 8,16 172 8,16 159

8,18 154 8,18 134 8,18 163 8,18 148

8,2 158 8,2 142 8,2 150 8,2 145

8,22 190 8,22 165 8,22 167 8,22 148

8,24 153 8,24 163 8,24 172 8,24 150

8,26 150 8,26 156 8,26 154 8,26 179

8,28 156 8,28 135 8,28 135 8,28 168

8,3 160 8,3 153 8,3 155 8,3 159

8,32 154 8,32 136 8,32 157 8,32 158

8,34 149 8,34 141 8,34 168 8,34 160

8,36 147 8,36 148 8,36 159 8,36 157

8,38 158 8,38 159 8,38 184 8,38 136

8,4 135 8,4 135 8,4 164 8,4 157

8,42 166 8,42 152 8,42 139 8,42 156

8,44 140 8,44 159 8,44 164 8,44 160

8,46 155 8,46 151 8,46 175 8,46 147

8,48 126 8,48 151 8,48 148 8,48 138

8,5 165 8,5 129 8,5 163 8,5 161

8,52 163 8,52 158 8,52 155 8,52 166

8,54 127 8,54 152 8,54 164 8,54 152

8,55 151 8,55 149 8,55 141 8,55 155

8,58 144 8,58 141 8,58 153 8,58 150

8,59 147 8,59 157 8,59 148 8,59 143

8,62 133 8,62 144 8,62 159 8,62 151

8,64 146 8,64 149 8,64 172 8,64 146

8,66 154 8,66 144 8,66 145 8,66 164

8,68 147 8,68 155 8,68 164 8,68 146

8,7 157 8,7 133 8,7 165 8,7 167

8,72 151 8,72 149 8,72 152 8,72 150

8,74 157 8,74 148 8,74 147 8,74 160

8,76 131 8,76 154 8,76 161 8,76 156

8,78 154 8,78 127 8,78 158 8,78 153

8,8 171 8,8 152 8,8 161 8,8 142

8,82 151 8,82 150 8,82 155 8,82 133

8,84 147 8,84 173 8,84 148 8,84 148

8,86 124 8,86 129 8,86 155 8,86 158

8,88 155 8,88 155 8,88 148 8,88 159

8,9 145 8,9 134 8,9 158 8,9 152

8,92 161 8,92 155 8,92 156 8,92 146

8,94 153 8,94 139 8,94 153 8,94 138

8,96 151 8,96 116 8,96 135 8,96 150

8,98 134 8,98 146 8,98 136 8,98 149

9 158 9 145 9 159 9 142

9,02 175 9,02 149 9,02 142 9,02 151

9,04 131 9,04 136 9,04 131 9,04 145

9,05 145 9,05 139 9,05 161 9,05 147

9,08 140 9,08 133 9,08 138 9,08 145

9,09 152 9,09 129 9,09 146 9,09 146

9,12 164 9,12 123 9,12 169 9,12 140

9,13 147 9,13 141 9,13 140 9,13 133

9,16 148 9,16 163 9,16 127 9,16 132

9,18 131 9,18 139 9,18 136 9,18 136

9,2 131 9,2 140 9,2 143 9,2 161

9,22 145 9,22 150 9,22 142 9,22 155

9,24 143 9,24 155 9,24 153 9,24 164

9,26 155 9,26 122 9,26 155 9,26 149

9,28 156 9,28 133 9,28 143 9,28 147

9,3 149 9,3 140 9,3 134 9,3 157

9,32 180 9,32 135 9,32 143 9,32 138

9,34 149 9,34 161 9,34 142 9,34 145

9,36 139 9,36 158 9,36 143 9,36 143

9,38 145 9,38 135 9,38 139 9,38 120

9,4 145 9,4 139 9,4 124 9,4 151

9,42 140 9,42 156 9,42 146 9,42 146

9,44 130 9,44 127 9,44 138 9,44 149

9,46 149 9,46 134 9,46 145 9,46 142

9,48 130 9,48 142 9,48 146 9,48 142

9,5 138 9,5 132 9,5 144 9,5 163

9,52 122 9,52 146 9,52 146 9,52 150

9,54 140 9,54 122 9,54 132 9,54 153

9,55 142 9,55 145 9,55 132 9,55 143

9,58 138 9,58 148 9,58 135 9,58 161

9,59 133 9,59 133 9,59 155 9,59 161

9,62 124 9,62 151 9,62 136 9,62 166

9,63 122 9,63 148 9,63 143 9,63 153

9,66 139 9,66 145 9,66 128 9,66 156

9,68 138 9,68 159 9,68 144 9,68 149

9,7 140 9,7 141 9,7 120 9,7 139

9,72 117 9,72 177 9,72 147 9,72 139

9,74 125 9,74 147 9,74 133 9,74 153

9,76 146 9,76 147 9,76 123 9,76 140

9,78 132 9,78 153 9,78 142 9,78 126

9,8 156 9,8 176 9,8 149 9,8 140

9,82 144 9,82 139 9,82 136 9,82 132

9,84 131 9,84 152 9,84 148 9,84 146

9,86 135 9,86 155 9,86 141 9,86 144

9,88 147 9,88 126 9,88 123 9,88 131

9,9 139 9,9 146 9,9 128 9,9 146

9,92 122 9,92 144 9,92 160 9,92 150

9,94 144 9,94 144 9,94 130 9,94 144

9,96 132 9,96 129 9,96 143 9,96 121

9,98 133 9,98 148 9,98 130 9,98 162

10 128 10 125 10 150 10 157

10,02 126 10,02 120 10,02 148 10,02 135

10,04 134 10,04 150 10,04 123 10,04 112

10,06 139 10,06 132 10,06 128 10,06 138

10,08 131 10,08 125 10,08 146 10,08 126

10,1 131 10,1 142 10,1 132 10,1 143

10,12 133 10,12 137 10,12 141 10,12 132

10,14 129 10,14 142 10,14 133 10,14 142

10,16 139 10,16 148 10,16 117 10,16 120

10,18 129 10,18 132 10,18 131 10,18 122

10,2 130 10,2 143 10,2 160 10,2 129

10,22 135 10,22 145 10,22 130 10,22 141

10,24 132 10,24 125 10,24 150 10,24 121

10,26 149 10,26 127 10,26 132 10,26 138

10,28 127 10,28 141 10,28 151 10,28 140

10,3 125 10,3 143 10,3 140 10,3 141

10,32 146 10,32 165 10,32 116 10,32 129

10,34 119 10,34 143 10,34 172 10,34 113

10,36 159 10,36 154 10,36 141 10,36 152

10,38 158 10,38 140 10,38 135 10,38 143

10,4 122 10,4 142 10,4 125 10,4 156

10,42 123 10,42 153 10,42 157 10,42 164

10,44 134 10,44 178 10,44 158 10,44 173

10,46 121 10,46 187 10,46 149 10,46 183

10,48 138 10,48 205 10,48 144 10,48 183

10,5 154 10,5 199 10,5 165 10,5 208

10,52 159 10,52 205 10,52 210 10,52 216

10,54 153 10,54 221 10,54 252 10,54 234

10,56 155 10,56 246 10,56 256 10,56 274

10,58 168 10,58 263 10,58 326 10,58 285

10,6 197 10,6 345 10,6 466 10,6 335

10,62 199 10,62 318 10,62 649 10,62 364

10,64 235 10,64 386 10,64 557 10,64 427

10,66 264 10,66 391 10,66 416 10,66 471

10,68 221 10,68 414 10,68 258 10,68 545

10,7 226 10,7 459 10,7 251 10,7 768

10,72 232 10,72 631 10,72 242 10,72 1032

10,74 268 10,74 680 10,74 270 10,74 1346

10,76 312 10,76 744 10,76 242 10,76 1902

10,78 395 10,78 831 10,78 249 10,78 2629

10,8 741 10,8 1000 10,8 398 10,8 3682

10,82 1380 10,82 1721 10,82 595 10,82 3698

10,84 2020 10,84 2421 10,84 1118 10,84 2792

10,86 1790 10,86 2869 10,86 1563 10,86 1610

10,88 1023 10,88 2916 10,88 1367 10,88 862

10,9 476 10,9 3077 10,9 726 10,9 470

10,92 225 10,92 2753 10,92 295 10,92 278

10,94 163 10,94 1963 10,94 153 10,94 184

10,96 143 10,96 1065 10,96 163 10,96 170

10,98 140 10,98 491 10,98 147 10,98 142

11 135 11 278 11 140 11 141

11,02 149 11,02 179 11,02 128 11,02 147

11,04 125 11,04 161 11,04 122 11,04 153

11,06 122 11,06 167 11,06 142 11,06 101

11,08 139 11,08 138 11,08 109 11,08 124

11,1 146 11,1 134 11,1 131 11,1 128

11,12 119 11,12 113 11,12 134 11,12 125

11,14 129 11,14 136 11,14 129 11,14 116

11,16 123 11,16 120 11,16 117 11,16 137

11,18 122 11,18 121 11,18 132 11,18 149

11,2 112 11,2 113 11,2 130 11,2 124

11,22 130 11,22 105 11,22 121 11,22 129

11,24 115 11,24 125 11,24 123 11,24 113

11,26 131 11,26 117 11,26 147 11,26 129

11,28 132 11,28 125 11,28 129 11,28 124

11,3 118 11,3 99 11,3 100 11,3 128

11,32 111 11,32 103 11,32 128 11,32 98

11,34 109 11,34 107 11,34 136 11,34 125

11,36 100 11,36 112 11,36 123 11,36 122

11,38 107 11,38 108 11,38 123 11,38 118

11,4 141 11,4 98 11,4 117 11,4 117

11,42 119 11,42 110 11,42 125 11,42 114

11,44 118 11,44 119 11,44 123 11,44 102

11,46 123 11,46 107 11,46 111 11,46 141

11,48 117 11,48 114 11,48 115 11,48 124

11,5 114 11,5 114 11,5 106 11,5 132

11,52 119 11,52 111 11,52 91 11,52 132

11,54 104 11,54 112 11,54 109 11,54 117

11,56 97 11,56 118 11,56 116 11,56 104

11,58 116 11,58 110 11,58 136 11,58 115

11,6 104 11,6 114 11,6 122 11,6 108

11,62 113 11,62 115 11,62 116 11,62 121

11,64 109 11,64 105 11,64 119 11,64 110

11,66 112 11,66 121 11,66 110 11,66 145

11,68 109 11,68 103 11,68 118 11,68 84

11,7 115 11,7 99 11,7 95 11,7 128

11,72 140 11,72 107 11,72 145 11,72 111

11,74 104 11,74 110 11,74 109 11,74 116

11,76 124 11,76 92 11,76 134 11,76 102

11,78 114 11,78 103 11,78 123 11,78 107

11,8 100 11,8 103 11,8 110 11,8 120

11,82 129 11,82 99 11,82 118 11,82 101

11,84 98 11,84 106 11,84 95 11,84 103

11,86 115 11,86 113 11,86 131 11,86 114

11,88 108 11,88 120 11,88 111 11,88 106

11,9 132 11,9 93 11,9 126 11,9 124

11,92 109 11,92 113 11,92 120 11,92 106

11,94 122 11,94 108 11,94 113 11,94 117

11,96 113 11,96 114 11,96 116 11,96 96

11,98 127 11,98 116 11,98 126 11,98 123

12 129 12 111 12 119 12 117

12,02 121 12,02 111 12,02 115 12,02 123

12,04 106 12,04 102 12,04 112 12,04 111

12,06 127 12,06 96 12,06 119 12,06 106

12,08 118 12,08 120 12,08 127 12,08 121

12,1 106 12,1 102 12,1 117 12,1 111

12,12 110 12,12 111 12,12 129 12,12 120

12,14 106 12,14 104 12,14 127 12,14 113

12,16 118 12,16 110 12,16 117 12,16 102

12,18 123 12,18 122 12,18 121 12,18 113

12,2 112 12,2 99 12,2 124 12,2 103

12,22 109 12,22 108 12,22 123 12,22 110

12,24 115 12,24 101 12,24 124 12,24 111

12,26 113 12,26 103 12,26 121 12,26 108

12,28 136 12,28 110 12,28 136 12,28 130

12,3 111 12,3 116 12,3 129 12,3 105

12,32 127 12,32 123 12,32 115 12,32 103

12,34 101 12,34 109 12,34 127 12,34 110

12,36 105 12,36 100 12,36 113 12,36 114

12,38 115 12,38 93 12,38 121 12,38 109

12,4 102 12,4 93 12,4 123 12,4 111

12,42 131 12,42 97 12,42 105 12,42 112

12,44 107 12,44 101 12,44 126 12,44 105

12,46 116 12,46 88 12,46 98 12,46 82

12,48 115 12,48 85 12,48 111 12,48 115

12,5 108 12,5 108 12,5 128 12,5 101

12,52 100 12,52 87 12,52 114 12,52 94

12,54 119 12,54 116 12,54 111 12,54 119

12,56 83 12,56 105 12,56 123 12,56 95

12,58 118 12,58 104 12,58 108 12,58 112

12,6 142 12,6 102 12,6 112 12,6 117

12,62 130 12,62 102 12,62 114 12,62 113

12,64 133 12,64 88 12,64 137 12,64 115

12,66 109 12,66 122 12,66 137 12,66 103

12,68 126 12,68 86 12,68 111 12,68 97

12,7 84 12,7 97 12,7 129 12,7 100

12,72 98 12,72 92 12,72 115 12,72 111

12,74 102 12,74 114 12,74 122 12,74 137

12,76 97 12,76 100 12,76 101 12,76 99

12,78 114 12,78 103 12,78 111 12,78 123

12,8 111 12,8 89 12,8 105 12,8 108

12,82 119 12,82 105 12,82 116 12,82 108

12,84 111 12,84 98 12,84 102 12,84 115

12,86 122 12,86 94 12,86 104 12,86 115

12,88 129 12,88 102 12,88 125 12,88 96

12,9 115 12,9 94 12,9 98 12,9 98

12,92 102 12,92 83 12,92 103 12,92 115

12,94 99 12,94 102 12,94 91 12,94 93

12,96 91 12,96 90 12,96 103 12,96 109

12,98 112 12,98 110 12,98 116 12,98 92

13 100 13 100 13 125 13 100

13,02 100 13,02 95 13,02 113 13,02 114

13,04 113 13,04 97 13,04 102 13,04 95

13,06 119 13,06 105 13,06 103 13,06 91

13,08 102 13,08 90 13,08 111 13,08 91

13,1 104 13,1 102 13,1 125 13,1 110

13,12 124 13,12 96 13,12 105 13,12 104

13,14 117 13,14 87 13,14 102 13,14 93

13,16 119 13,16 96 13,16 117 13,16 119

13,18 88 13,18 93 13,18 110 13,18 99

13,2 110 13,2 93 13,2 87 13,2 90

13,22 86 13,22 94 13,22 99 13,22 85

13,24 106 13,24 103 13,24 94 13,24 105

13,26 118 13,26 98 13,26 104 13,26 115

13,28 93 13,28 108 13,28 104 13,28 80

13,3 105 13,3 108 13,3 113 13,3 114

13,32 111 13,32 105 13,32 114 13,32 86

13,34 112 13,34 97 13,34 125 13,34 110

13,36 76 13,36 97 13,36 74 13,36 76

13,38 81 13,38 96 13,38 113 13,38 94

13,4 99 13,4 87 13,4 105 13,4 104

13,42 104 13,42 100 13,42 106 13,42 96

13,44 115 13,44 94 13,44 114 13,44 101

13,46 96 13,46 96 13,46 105 13,46 112

13,48 105 13,48 76 13,48 105 13,48 86

13,5 120 13,5 104 13,5 94 13,5 99

13,52 94 13,52 96 13,52 99 13,52 106

13,54 133 13,54 92 13,54 95 13,54 91

13,56 106 13,56 76 13,56 113 13,56 93

13,58 106 13,58 95 13,58 92 13,58 98

13,6 99 13,6 104 13,6 115 13,6 99

13,62 101 13,62 89 13,62 98 13,62 105

13,64 113 13,64 90 13,64 96 13,64 118

13,66 101 13,66 92 13,66 105 13,66 112

13,68 104 13,68 111 13,68 101 13,68 97

13,7 103 13,7 101 13,7 107 13,7 106

13,72 76 13,72 74 13,72 102 13,72 97

13,74 112 13,74 78 13,74 98 13,74 85

13,76 97 13,76 92 13,76 123 13,76 82

13,78 90 13,78 96 13,78 103 13,78 95

13,8 103 13,8 85 13,8 119 13,8 99

13,82 98 13,82 87 13,82 125 13,82 127

13,84 101 13,84 88 13,84 118 13,84 108

13,86 111 13,86 95 13,86 91 13,86 116

13,88 96 13,88 91 13,88 94 13,88 87

13,9 104 13,9 107 13,9 125 13,9 85

13,92 101 13,92 103 13,92 116 13,92 106

13,94 109 13,94 87 13,94 127 13,94 98

13,96 124 13,96 91 13,96 119 13,96 99

13,98 103 13,98 100 13,98 116 13,98 122

14 104 14 97 14 115 14 107

14,02 78 14,02 105 14,02 99 14,02 109

14,04 89 14,04 90 14,04 107 14,04 108

14,06 93 14,06 106 14,06 127 14,06 148

14,08 113 14,08 97 14,08 136 14,08 126

14,1 98 14,1 115 14,1 137 14,1 140

14,12 113 14,12 131 14,12 158 14,12 143

14,14 109 14,14 131 14,14 130 14,14 145

14,16 107 14,16 140 14,16 187 14,16 186

14,18 126 14,18 151 14,18 202 14,18 179

14,2 114 14,2 165 14,2 278 14,2 198

14,22 124 14,22 154 14,22 411 14,22 222

14,24 120 14,24 176 14,24 394 14,24 204

14,26 142 14,26 186 14,26 346 14,26 243

14,28 119 14,28 246 14,28 233 14,28 259

14,3 124 14,3 242 14,3 178 14,3 327

14,32 124 14,32 242 14,32 147 14,32 414

14,34 136 14,34 271 14,34 131 14,34 479

14,36 146 14,36 314 14,36 146 14,36 703

14,38 149 14,38 399 14,38 149 14,38 1011

14,4 270 14,4 483 14,4 176 14,4 1534

14,42 448 14,42 706 14,42 236 14,42 1780

14,44 647 14,44 932 14,44 376 14,44 1637

14,46 605 14,46 992 14,46 635 14,46 1177

14,48 474 14,48 1106 14,48 750 14,48 758

14,5 315 14,5 1096 14,5 559 14,5 427

14,52 177 14,52 1080 14,52 384 14,52 257

14,54 129 14,54 854 14,54 188 14,54 188

14,56 99 14,56 630 14,56 125 14,56 162

14,58 110 14,58 393 14,58 89 14,58 132

14,6 121 14,6 230 14,6 124 14,6 117

14,62 128 14,62 129 14,62 119 14,62 106

14,64 100 14,64 107 14,64 117 14,64 101

14,66 98 14,66 108 14,66 100 14,66 105

14,68 120 14,68 113 14,68 109 14,68 86

14,7 102 14,7 89 14,7 104 14,7 100

14,72 102 14,72 100 14,72 104 14,72 93

14,74 108 14,74 100 14,74 94 14,74 100

14,76 107 14,76 99 14,76 84 14,76 86

14,78 96 14,78 83 14,78 84 14,78 104

14,8 96 14,8 70 14,8 101 14,8 94

14,82 87 14,82 88 14,82 107 14,82 93

14,84 107 14,84 87 14,84 117 14,84 115

14,86 104 14,86 87 14,86 103 14,86 95

14,88 106 14,88 89 14,88 105 14,88 93

14,9 112 14,9 96 14,9 106 14,9 91

14,92 104 14,92 89 14,92 109 14,92 79

14,94 112 14,94 88 14,94 119 14,94 108

14,96 90 14,96 87 14,96 104 14,96 86

14,98 108 14,98 88 14,98 109 14,98 87

15 97 15 109 15 99 15 103

15,02 92 15,02 95 15,02 142 15,02 104

15,04 122 15,04 91 15,04 101 15,04 97

15,06 99 15,06 94 15,06 107 15,06 107

15,08 122 15,08 90 15,08 122 15,08 84

15,1 143 15,1 107 15,1 108 15,1 105

15,12 112 15,12 103 15,12 100 15,12 109

15,14 106 15,14 78 15,14 101 15,14 125

15,16 135 15,16 113 15,16 128 15,16 121

15,18 126 15,18 122 15,18 120 15,18 123

15,2 118 15,2 126 15,2 107 15,2 121

15,22 122 15,22 125 15,22 109 15,22 117

15,24 120 15,24 116 15,24 120 15,24 119

15,26 115 15,26 127 15,26 135 15,26 110

15,28 127 15,28 136 15,28 119 15,28 106

15,3 108 15,3 113 15,3 138 15,3 116

15,32 121 15,32 112 15,32 107 15,32 95

15,34 117 15,34 115 15,34 111 15,34 118

15,36 101 15,36 109 15,36 127 15,36 103

15,38 105 15,38 102 15,38 106 15,38 132

15,4 113 15,4 98 15,4 120 15,4 87

15,42 110 15,42 111 15,42 112 15,42 103

15,44 105 15,44 108 15,44 112 15,44 93

15,46 113 15,46 121 15,46 131 15,46 93

15,48 115 15,48 94 15,48 115 15,48 102

15,5 123 15,5 87 15,5 113 15,5 108

15,52 94 15,52 84 15,52 102 15,52 89

15,54 101 15,54 105 15,54 104 15,54 112

15,56 104 15,56 81 15,56 105 15,56 102

15,58 87 15,58 99 15,58 111 15,58 99

15,6 102 15,6 82 15,6 100 15,6 97

15,62 105 15,62 82 15,62 116 15,62 98

15,64 95 15,64 77 15,64 118 15,64 96

15,66 109 15,66 87 15,66 96 15,66 103

15,68 121 15,68 95 15,68 121 15,68 94

15,7 102 15,7 75 15,7 136 15,7 92

15,72 117 15,72 90 15,72 127 15,72 104

15,74 120 15,74 96 15,74 140 15,74 85

15,76 106 15,76 92 15,76 126 15,76 109

15,78 107 15,78 104 15,78 108 15,78 103

15,8 97 15,8 92 15,8 122 15,8 105

15,82 141 15,82 99 15,82 95 15,82 119

15,84 143 15,84 95 15,84 90 15,84 96

15,86 182 15,86 98 15,86 106 15,86 99

15,88 141 15,88 99 15,88 119 15,88 119

15,9 142 15,9 103 15,9 105 15,9 104

15,92 143 15,92 85 15,92 100 15,92 101

15,94 117 15,94 103 15,94 106 15,94 107

15,96 105 15,96 129 15,96 105 15,96 102

15,98 91 15,98 98 15,98 91 15,98 95

16 124 16 84 16 102 16 86

16,02 110 16,02 68 16,02 124 16,02 81

16,04 106 16,04 89 16,04 101 16,04 96

16,06 85 16,06 82 16,06 101 16,06 94

16,08 107 16,08 82 16,08 117 16,08 90

16,1 104 16,1 101 16,1 83 16,1 86

16,12 80 16,12 81 16,12 119 16,12 103

16,14 109 16,14 87 16,14 111 16,14 99

16,16 101 16,16 91 16,16 98 16,16 92

16,18 94 16,18 86 16,18 99 16,18 105

16,2 109 16,2 97 16,2 110 16,2 108

16,22 118 16,22 85 16,22 111 16,22 116

16,24 122 16,24 97 16,24 108 16,24 89

16,26 103 16,26 94 16,26 110 16,26 97

16,28 108 16,28 78 16,28 106 16,28 91

16,3 110 16,3 101 16,3 122 16,3 103

16,32 93 16,32 89 16,32 135 16,32 111

16,34 122 16,34 82 16,34 112 16,34 110

16,36 104 16,36 71 16,36 110 16,36 116

16,38 108 16,38 91 16,38 102 16,38 117

16,4 104 16,4 97 16,4 93 16,4 105

16,42 117 16,42 111 16,42 122 16,42 101

16,44 87 16,44 90 16,44 92 16,44 96

16,46 88 16,46 100 16,46 99 16,46 83

16,48 109 16,48 106 16,48 105 16,48 99

16,5 89 16,5 105 16,5 108 16,5 86

16,52 122 16,52 93 16,52 133 16,52 99

16,54 89 16,54 83 16,54 123 16,54 92

16,56 106 16,56 87 16,56 115 16,56 82

16,58 95 16,58 82 16,58 122 16,58 89

16,6 99 16,6 76 16,6 125 16,6 103

16,62 79 16,62 95 16,62 119 16,62 100

16,64 95 16,64 106 16,64 108 16,64 87

16,66 99 16,66 92 16,66 136 16,66 92

16,68 97 16,68 97 16,68 122 16,68 99

16,7 118 16,7 117 16,7 107 16,7 99

16,72 97 16,72 95 16,72 97 16,72 91

16,74 108 16,74 74 16,74 101 16,74 96

16,76 99 16,76 101 16,76 124 16,76 91

16,78 107 16,78 96 16,78 94 16,78 84

16,8 121 16,8 101 16,8 117 16,8 92

16,82 98 16,82 94 16,82 101 16,82 90

16,84 128 16,84 82 16,84 114 16,84 121

16,86 123 16,86 87 16,86 116 16,86 96

16,88 109 16,88 98 16,88 118 16,88 104

16,9 97 16,9 96 16,9 116 16,9 112

16,92 110 16,92 87 16,92 109 16,92 119

16,94 118 16,94 100 16,94 121 16,94 120

16,96 111 16,96 90 16,96 117 16,96 125

16,98 104 16,98 73 16,98 117 16,98 122

17 124 17 95 17 106 17 96

17,02 88 17,02 110 17,02 120 17,02 114

17,04 118 17,04 109 17,04 132 17,04 122

17,06 114 17,06 104 17,06 131 17,06 104

17,08 126 17,08 105 17,08 127 17,08 99

17,1 103 17,1 106 17,1 140 17,1 113

17,12 105 17,12 101 17,12 127 17,12 96

17,14 103 17,14 108 17,14 138 17,14 93

17,16 121 17,16 114 17,16 113 17,16 107

17,18 105 17,18 97 17,18 129 17,18 87

17,2 108 17,2 102 17,2 102 17,2 99

17,22 113 17,22 94 17,22 96 17,22 101

17,24 133 17,24 91 17,24 125 17,24 130

17,26 142 17,26 83 17,26 97 17,26 98

17,28 115 17,28 81 17,28 119 17,28 88

17,3 125 17,3 85 17,3 128 17,3 88

17,32 113 17,32 98 17,32 101 17,32 100

17,34 112 17,34 89 17,34 108 17,34 94

17,36 103 17,36 87 17,36 114 17,36 94

17,38 102 17,38 103 17,38 131 17,38 75

17,4 117 17,4 83 17,4 125 17,4 103

17,42 96 17,42 99 17,42 102 17,42 77

17,44 100 17,44 85 17,44 124 17,44 90

17,46 99 17,46 89 17,46 113 17,46 95

17,48 95 17,48 94 17,48 110 17,48 109

17,5 108 17,5 95 17,5 120 17,5 83

17,52 96 17,52 75 17,52 106 17,52 107

17,54 101 17,54 92 17,54 124 17,54 105

17,56 107 17,56 95 17,56 124 17,56 98

17,58 96 17,58 99 17,58 120 17,58 112

17,6 94 17,6 100 17,6 124 17,6 125

17,62 92 17,62 86 17,62 153 17,62 123

17,64 98 17,64 113 17,64 152 17,64 152

17,66 92 17,66 116 17,66 150 17,66 178

17,68 105 17,68 118 17,68 141 17,68 166

17,7 88 17,7 111 17,7 172 17,7 153

17,72 116 17,72 139 17,72 185 17,72 186

17,74 99 17,74 142 17,74 174 17,74 208

17,76 110 17,76 167 17,76 165 17,76 254

17,78 150 17,78 184 17,78 193 17,78 261

17,8 164 17,8 219 17,8 258 17,8 317

17,82 120 17,82 246 17,82 652 17,82 326

17,84 142 17,84 275 17,84 1251 17,84 414

17,86 157 17,86 321 17,86 1369 17,86 461

17,88 156 17,88 378 17,88 975 17,88 561

17,9 162 17,9 429 17,9 787 17,9 693

17,92 153 17,92 460 17,92 494 17,92 886

17,94 183 17,94 509 17,94 283 17,94 948

17,96 186 17,96 546 17,96 242 17,96 1193

17,98 228 17,98 700 17,98 233 17,98 1488

18 278 18 916 18 271 18 2230

18,02 420 18,02 1210 18,02 267 18,02 3435

18,04 804 18,04 1599 18,04 348 18,04 4794

18,06 1314 18,06 1942 18,06 598 18,06 4463

18,08 1339 18,08 2072 18,08 1094 18,08 3615

18,1 995 18,1 2355 18,1 1521 18,1 2519

18,12 828 18,12 2420 18,12 1349 18,12 1612

18,14 469 18,14 2517 18,14 738 18,14 829

18,16 278 18,16 2208 18,16 658 18,16 462

18,18 193 18,18 1764 18,18 374 18,18 300

18,2 151 18,2 1134 18,2 175 18,2 221

18,22 142 18,22 766 18,22 122 18,22 171

18,24 140 18,24 391 18,24 144 18,24 146

18,26 149 18,26 204 18,26 127 18,26 132

18,28 140 18,28 153 18,28 117 18,28 105

18,3 134 18,3 144 18,3 123 18,3 98

18,32 120 18,32 118 18,32 116 18,32 94

18,34 122 18,34 110 18,34 120 18,34 87

18,36 114 18,36 117 18,36 108 18,36 90

18,38 101 18,38 97 18,38 119 18,38 99

18,4 100 18,4 91 18,4 105 18,4 89

18,42 116 18,42 97 18,42 96 18,42 95

18,44 104 18,44 83 18,44 125 18,44 91

18,46 109 18,46 91 18,46 113 18,46 95

18,48 106 18,48 97 18,48 118 18,48 93

18,5 107 18,5 84 18,5 110 18,5 86

18,52 87 18,52 87 18,52 110 18,52 96

18,54 114 18,54 88 18,54 120 18,54 105

18,56 101 18,56 84 18,56 124 18,56 79

18,58 122 18,58 68 18,58 88 18,58 82

18,6 98 18,6 81 18,6 118 18,6 82

18,62 103 18,62 76 18,62 108 18,62 81

18,64 108 18,64 92 18,64 100 18,64 86

18,66 96 18,66 85 18,66 97 18,66 93

18,68 95 18,68 75 18,68 109 18,68 89

18,7 103 18,7 83 18,7 104 18,7 82

18,72 98 18,72 66 18,72 103 18,72 94

18,74 106 18,74 78 18,74 109 18,74 86

18,76 110 18,76 71 18,76 111 18,76 77

18,78 81 18,78 66 18,78 94 18,78 104

18,8 106 18,8 74 18,8 102 18,8 75

18,82 116 18,82 81 18,82 94 18,82 102

18,84 93 18,84 72 18,84 110 18,84 80

18,86 108 18,86 88 18,86 101 18,86 80

18,88 96 18,88 70 18,88 113 18,88 86

18,9 101 18,9 72 18,9 100 18,9 85

18,92 107 18,92 96 18,92 94 18,92 96

18,94 96 18,94 80 18,94 92 18,94 84

18,96 124 18,96 64 18,96 100 18,96 87

18,98 86 18,98 76 18,98 96 18,98 103

19 88 19 68 19 103 19 102

19,02 102 19,02 74 19,02 96 19,02 82

19,04 116 19,04 76 19,04 115 19,04 105

19,06 93 19,06 71 19,06 120 19,06 96

19,08 101 19,08 78 19,08 119 19,08 112

19,1 100 19,1 76 19,1 106 19,1 80

19,12 109 19,12 70 19,12 106 19,12 75

19,14 111 19,14 71 19,14 118 19,14 79

19,16 116 19,16 79 19,16 128 19,16 104

19,18 106 19,18 82 19,18 128 19,18 82

19,2 121 19,2 95 19,2 108 19,2 102

19,22 118 19,22 96 19,22 126 19,22 83

19,24 140 19,24 74 19,24 131 19,24 93

19,26 140 19,26 93 19,26 136 19,26 102

19,28 118 19,28 109 19,28 110 19,28 85

19,3 90 19,3 100 19,3 128 19,3 101

19,32 129 19,32 73 19,32 125 19,32 113

19,34 113 19,34 81 19,34 140 19,34 81

19,36 127 19,36 91 19,36 136 19,36 99

19,38 137 19,38 99 19,38 112 19,38 87

19,4 115 19,4 88 19,4 106 19,4 85

19,42 100 19,42 92 19,42 120 19,42 104

19,44 121 19,44 97 19,44 131 19,44 82

19,46 105 19,46 86 19,46 109 19,46 80

19,48 117 19,48 98 19,48 133 19,48 90

19,5 123 19,5 67 19,5 103 19,5 91

19,52 102 19,52 90 19,52 124 19,52 110

19,54 96 19,54 86 19,54 109 19,54 107

19,56 114 19,56 82 19,56 114 19,56 91

19,58 105 19,58 66 19,58 129 19,58 81

19,6 112 19,6 107 19,6 135 19,6 77

19,62 117 19,62 80 19,62 142 19,62 92

19,64 132 19,64 88 19,64 125 19,64 94

19,66 125 19,66 65 19,66 141 19,66 108

19,68 132 19,68 97 19,68 143 19,68 100

19,7 128 19,7 101 19,7 134 19,7 110

19,72 126 19,72 103 19,72 130 19,72 113

19,74 158 19,74 92 19,74 124 19,74 103

19,76 119 19,76 74 19,76 145 19,76 101

19,78 120 19,78 108 19,78 144 19,78 115

19,8 124 19,8 109 19,8 146 19,8 118

19,82 113 19,82 103 19,82 148 19,82 115

19,84 137 19,84 113 19,84 121 19,84 132

19,86 144 19,86 123 19,86 126 19,86 121

19,88 115 19,88 139 19,88 161 19,88 119

19,9 133 19,9 129 19,9 161 19,9 97

19,92 145 19,92 117 19,92 160 19,92 109

19,94 129 19,94 124 19,94 142 19,94 132

19,96 129 19,96 137 19,96 127 19,96 125

19,98 113 19,98 138 19,98 147 19,98 126

20 125 20 126 20 125 20 110

20,02 120 20,02 129 20,02 116 20,02 131

20,04 136 20,04 122 20,04 132 20,04 132

20,06 145 20,06 126 20,06 116 20,06 120

20,08 132 20,08 121 20,08 132 20,08 132

20,1 137 20,1 143 20,1 130 20,1 120

20,12 112 20,12 110 20,12 126 20,12 101

20,14 127 20,14 104 20,14 118 20,14 96

20,16 132 20,16 125 20,16 126 20,16 93

20,18 144 20,18 104 20,18 144 20,18 81

20,2 159 20,2 117 20,2 135 20,2 95

20,22 156 20,22 128 20,22 139 20,22 93

20,24 147 20,24 120 20,24 134 20,24 118

20,26 153 20,26 102 20,26 141 20,26 108

20,28 168 20,28 105 20,28 142 20,28 92

20,3 132 20,3 123 20,3 113 20,3 93

20,32 127 20,32 91 20,32 129 20,32 105

20,34 126 20,34 102 20,34 130 20,34 96

20,36 150 20,36 96 20,36 104 20,36 81

20,38 123 20,38 94 20,38 125 20,38 87

20,4 137 20,4 81 20,4 115 20,4 89

20,42 128 20,42 85 20,42 114 20,42 89

20,44 132 20,44 102 20,44 107 20,44 86

20,46 123 20,46 90 20,46 126 20,46 84

20,48 113 20,48 82 20,48 108 20,48 83

20,5 116 20,5 88 20,5 117 20,5 93

20,52 120 20,52 72 20,52 97 20,52 112

20,54 134 20,54 65 20,54 103 20,54 107

20,56 125 20,56 70 20,56 116 20,56 87

20,58 116 20,58 85 20,58 108 20,58 94

20,6 109 20,6 72 20,6 113 20,6 76

20,62 88 20,62 76 20,62 109 20,62 91

20,64 125 20,64 97 20,64 129 20,64 103

20,66 95 20,66 83 20,66 105 20,66 84

20,68 118 20,68 70 20,68 125 20,68 92

20,7 105 20,7 75 20,7 106 20,7 85

20,72 111 20,72 93 20,72 102 20,72 99

20,74 116 20,74 95 20,74 108 20,74 100

20,76 118 20,76 86 20,76 123 20,76 94

20,78 111 20,78 83 20,78 104 20,78 86

20,8 121 20,8 75 20,8 121 20,8 89

20,82 114 20,82 74 20,82 110 20,82 86

20,84 118 20,84 85 20,84 118 20,84 79

20,86 96 20,86 83 20,86 118 20,86 93

20,88 110 20,88 77 20,88 100 20,88 76

20,9 122 20,9 81 20,9 96 20,9 114

20,92 104 20,92 87 20,92 107 20,92 94

20,94 106 20,94 89 20,94 96 20,94 102

20,96 100 20,96 89 20,96 112 20,96 81

20,98 122 20,98 89 20,98 107 20,98 86

21 124 21 92 21 116 21 81

21,02 112 21,02 94 21,02 81 21,02 96

21,04 122 21,04 93 21,04 106 21,04 97

21,06 114 21,06 79 21,06 104 21,06 117

21,08 107 21,08 72 21,08 124 21,08 79

21,1 100 21,1 76 21,1 112 21,1 74

21,12 125 21,12 82 21,12 134 21,12 99

21,14 126 21,14 83 21,14 114 21,14 95

21,16 108 21,16 98 21,16 108 21,16 87

21,18 115 21,18 93 21,18 129 21,18 81

21,2 123 21,2 100 21,2 148 21,2 103

21,22 141 21,22 93 21,22 130 21,22 90

21,24 146 21,24 88 21,24 140 21,24 98

21,26 153 21,26 108 21,26 157 21,26 108

21,28 243 21,28 123 21,28 173 21,28 103

21,3 335 21,3 114 21,3 148 21,3 122

21,32 498 21,32 124 21,32 189 21,32 158

21,34 847 21,34 135 21,34 157 21,34 147

21,36 1331 21,36 131 21,36 167 21,36 182

21,38 1463 21,38 161 21,38 182 21,38 194

21,4 1178 21,4 128 21,4 163 21,4 191

21,42 902 21,42 155 21,42 155 21,42 186

21,44 720 21,44 195 21,44 183 21,44 185

21,46 467 21,46 200 21,46 335 21,46 172

21,48 310 21,48 187 21,48 531 21,48 219

21,5 212 21,5 227 21,5 693 21,5 187

21,52 172 21,52 256 21,52 479 21,52 276

21,54 170 21,54 219 21,54 352 21,54 330

21,56 132 21,56 262 21,56 348 21,56 353

21,58 149 21,58 243 21,58 205 21,58 373

21,6 163 21,6 265 21,6 175 21,6 404

21,62 146 21,62 302 21,62 141 21,62 495

21,64 162 21,64 326 21,64 175 21,64 646

21,66 195 21,66 403 21,66 161 21,66 961

21,68 301 21,68 445 21,68 210 21,68 1485

21,7 401 21,7 521 21,7 215 21,7 1400

21,72 504 21,72 539 21,72 348 21,72 1046

21,74 387 21,74 628 21,74 500 21,74 1010

21,76 353 21,76 685 21,76 503 21,76 776

21,78 286 21,78 739 21,78 371 21,78 469

21,8 227 21,8 708 21,8 290 21,8 303

21,82 153 21,82 582 21,82 273 21,82 226

21,84 131 21,84 493 21,84 182 21,84 169

21,86 130 21,86 328 21,86 153 21,86 119

21,88 128 21,88 225 21,88 122 21,88 127

21,9 120 21,9 187 21,9 125 21,9 111

21,92 129 21,92 136 21,92 130 21,92 92

21,94 138 21,94 127 21,94 132 21,94 97

21,96 104 21,96 103 21,96 128 21,96 95

21,98 126 21,98 106 21,98 154 21,98 102

22 130 22 93 22 143 22 97

22,02 130 22,02 103 22,02 152 22,02 93

22,04 117 22,04 104 22,04 143 22,04 102

22,06 121 22,06 105 22,06 147 22,06 84

22,08 122 22,08 107 22,08 150 22,08 101

22,1 128 22,1 98 22,1 169 22,1 105

22,12 128 22,12 96 22,12 167 22,12 108

22,14 111 22,14 91 22,14 154 22,14 104

22,16 110 22,16 91 22,16 161 22,16 98

22,18 134 22,18 108 22,18 185 22,18 99

22,2 176 22,2 119 22,2 157 22,2 101

22,22 132 22,22 105 22,22 180 22,22 143

22,24 145 22,24 102 22,24 155 22,24 144

22,26 151 22,26 108 22,26 160 22,26 139

22,28 158 22,28 103 22,28 154 22,28 112

22,3 157 22,3 124 22,3 136 22,3 112

22,32 152 22,32 123 22,32 122 22,32 95

22,34 136 22,34 122 22,34 114 22,34 89

22,36 129 22,36 103 22,36 129 22,36 98

22,38 120 22,38 129 22,38 126 22,38 105

22,4 132 22,4 108 22,4 106 22,4 107

22,42 120 22,42 86 22,42 110 22,42 83

22,44 102 22,44 111 22,44 117 22,44 85

22,46 108 22,46 90 22,46 122 22,46 75

22,48 112 22,48 99 22,48 115 22,48 77

22,5 121 22,5 78 22,5 108 22,5 86

22,52 117 22,52 75 22,52 133 22,52 86

22,54 115 22,54 91 22,54 117 22,54 77

22,56 112 22,56 81 22,56 120 22,56 100

22,58 128 22,58 80 22,58 122 22,58 101

22,6 104 22,6 90 22,6 113 22,6 98

22,62 113 22,62 96 22,62 127 22,62 90

22,64 126 22,64 91 22,64 113 22,64 83

22,66 128 22,66 79 22,66 117 22,66 115

22,68 118 22,68 78 22,68 105 22,68 102

22,7 144 22,7 94 22,7 118 22,7 85

22,72 120 22,72 85 22,72 112 22,72 99

22,74 106 22,74 84 22,74 116 22,74 91

22,76 133 22,76 90 22,76 132 22,76 102

22,78 120 22,78 90 22,78 127 22,78 109

22,8 140 22,8 104 22,8 119 22,8 94

22,82 121 22,82 81 22,82 116 22,82 105

22,84 114 22,84 78 22,84 124 22,84 81

22,86 98 22,86 111 22,86 134 22,86 107

22,88 127 22,88 94 22,88 122 22,88 96

22,9 127 22,9 117 22,9 112 22,9 92

22,92 124 22,92 106 22,92 123 22,92 94

22,94 130 22,94 91 22,94 120 22,94 85

22,96 119 22,96 68 22,96 126 22,96 85

22,98 143 22,98 111 22,98 154 22,98 99

23 125 23 102 23 130 23 109

23,02 119 23,02 114 23,02 119 23,02 92

23,04 129 23,04 102 23,04 114 23,04 111

23,06 164 23,06 98 23,06 155 23,06 95

23,08 163 23,08 82 23,08 167 23,08 83

23,1 148 23,1 98 23,1 155 23,1 101

23,12 127 23,12 86 23,12 144 23,12 103

23,14 136 23,14 107 23,14 161 23,14 113

23,16 154 23,16 99 23,16 134 23,16 86

23,18 120 23,18 92 23,18 151 23,18 111

23,2 115 23,2 84 23,2 121 23,2 114

23,22 124 23,22 97 23,22 122 23,22 98

23,24 116 23,24 103 23,24 156 23,24 108

23,26 126 23,26 108 23,26 157 23,26 130

23,28 127 23,28 122 23,28 165 23,28 118

23,3 144 23,3 102 23,3 178 23,3 109

23,32 151 23,32 118 23,32 175 23,32 142

23,34 113 23,34 121 23,34 154 23,34 142

23,36 139 23,36 109 23,36 175 23,36 146

23,38 141 23,38 124 23,38 168 23,38 143

23,4 159 23,4 135 23,4 154 23,4 116

23,42 166 23,42 136 23,42 150 23,42 150

23,44 167 23,44 163 23,44 155 23,44 169

23,46 159 23,46 161 23,46 118 23,46 131

23,48 154 23,48 148 23,48 146 23,48 116

23,5 148 23,5 180 23,5 112 23,5 96

23,52 130 23,52 182 23,52 119 23,52 108

23,54 119 23,54 179 23,54 139 23,54 111

23,56 121 23,56 147 23,56 104 23,56 108

23,58 114 23,58 148 23,58 129 23,58 97

23,6 115 23,6 85 23,6 123 23,6 93

23,62 125 23,62 91 23,62 121 23,62 111

23,64 122 23,64 108 23,64 114 23,64 78

23,66 125 23,66 91 23,66 104 23,66 97

23,68 119 23,68 83 23,68 104 23,68 98

23,7 107 23,7 88 23,7 103 23,7 98

23,72 122 23,72 89 23,72 125 23,72 88

23,74 130 23,74 73 23,74 125 23,74 77

23,76 119 23,76 75 23,76 115 23,76 94

23,78 114 23,78 71 23,78 139 23,78 113

23,8 108 23,8 84 23,8 109 23,8 94

23,82 98 23,82 87 23,82 110 23,82 97

23,84 113 23,84 82 23,84 109 23,84 72

23,86 108 23,86 68 23,86 126 23,86 72

23,88 106 23,88 86 23,88 111 23,88 79

23,9 104 23,9 71 23,9 117 23,9 114

23,92 111 23,92 80 23,92 107 23,92 99

23,94 100 23,94 77 23,94 113 23,94 80

23,96 130 23,96 73 23,96 112 23,96 89

23,98 127 23,98 90 23,98 123 23,98 92

24 124 24 92 24 96 24 100

24,02 112 24,02 64 24,02 110 24,02 99

24,04 121 24,04 74 24,04 118 24,04 96

24,06 139 24,06 77 24,06 131 24,06 74

24,08 152 24,08 85 24,08 119 24,08 98

24,1 129 24,1 82 24,1 114 24,1 98

24,12 128 24,12 80 24,12 129 24,12 95

24,14 122 24,14 85 24,14 128 24,14 107

24,16 100 24,16 97 24,16 123 24,16 111

24,18 105 24,18 97 24,18 127 24,18 100

24,2 124 24,2 89 24,2 149 24,2 90

24,22 119 24,22 91 24,22 115 24,22 96

24,24 121 24,24 97 24,24 135 24,24 111

24,26 131 24,26 113 24,26 130 24,26 90

24,28 125 24,28 89 24,28 127 24,28 111

24,3 132 24,3 109 24,3 150 24,3 111

24,32 131 24,32 105 24,32 133 24,32 106

24,34 141 24,34 106 24,34 144 24,34 108

24,36 142 24,36 101 24,36 129 24,36 95

24,38 166 24,38 116 24,38 119 24,38 108

24,4 149 24,4 116 24,4 139 24,4 115

24,42 145 24,42 106 24,42 138 24,42 140

24,44 144 24,44 109 24,44 130 24,44 144

24,46 156 24,46 120 24,46 141 24,46 137

24,48 162 24,48 126 24,48 138 24,48 135

24,5 136 24,5 133 24,5 127 24,5 141

24,52 145 24,52 128 24,52 135 24,52 109

24,54 134 24,54 121 24,54 120 24,54 122

24,56 149 24,56 124 24,56 140 24,56 107

24,58 114 24,58 139 24,58 100 24,58 98

24,6 148 24,6 96 24,6 103 24,6 94

24,62 128 24,62 143 24,62 126 24,62 93

24,64 127 24,64 164 24,64 106 24,64 90

24,66 126 24,66 139 24,66 102 24,66 102

24,68 129 24,68 102 24,68 140 24,68 107

24,7 127 24,7 113 24,7 131 24,7 94

24,72 143 24,72 124 24,72 119 24,72 103

24,74 114 24,74 104 24,74 124 24,74 91

24,76 124 24,76 91 24,76 117 24,76 107

24,78 135 24,78 91 24,78 113 24,78 111

24,8 128 24,8 92 24,8 131 24,8 117

24,82 126 24,82 114 24,82 98 24,82 114

24,84 119 24,84 92 24,84 128 24,84 125

24,86 138 24,86 104 24,86 129 24,86 114

24,88 152 24,88 103 24,88 123 24,88 96

24,9 158 24,9 123 24,9 135 24,9 96

24,92 137 24,92 111 24,92 150 24,92 102

24,94 131 24,94 99 24,94 149 24,94 124

24,96 154 24,96 118 24,96 164 24,96 121

24,98 133 24,98 106 24,98 166 24,98 121

25 147 25 127 25 169 25 149

25,02 128 25,02 129 25,02 168 25,02 125

25,04 114 25,04 128 25,04 136 25,04 156

25,06 155 25,06 137 25,06 196 25,06 144

25,08 129 25,08 167 25,08 159 25,08 136

25,1 167 25,1 302 25,1 159 25,1 147

25,12 137 25,12 587 25,12 148 25,12 156

25,14 161 25,14 796 25,14 147 25,14 145

25,16 150 25,16 604 25,16 165 25,16 127

25,18 153 25,18 328 25,18 141 25,18 135

25,2 138 25,2 394 25,2 140 25,2 133

25,22 139 25,22 398 25,22 114 25,22 119

25,24 122 25,24 190 25,24 131 25,24 132

25,26 138 25,26 133 25,26 120 25,26 128

25,28 119 25,28 122 25,28 128 25,28 122

25,3 125 25,3 157 25,3 117 25,3 147

25,32 141 25,32 131 25,32 125 25,32 122

25,34 130 25,34 127 25,34 135 25,34 190

25,36 128 25,36 113 25,36 130 25,36 171

25,38 161 25,38 119 25,38 133 25,38 185

25,4 133 25,4 120 25,4 156 25,4 156

25,42 116 25,42 149 25,42 145 25,42 152

25,44 135 25,44 118 25,44 121 25,44 137

25,46 134 25,46 119 25,46 130 25,46 131

25,48 132 25,48 145 25,48 130 25,48 114

25,5 133 25,5 123 25,5 143 25,5 91

25,52 134 25,52 100 25,52 124 25,52 110

25,54 114 25,54 113 25,54 145 25,54 92

25,56 118 25,56 118 25,56 134 25,56 106

25,58 136 25,58 113 25,58 124 25,58 94

25,6 136 25,6 101 25,6 126 25,6 109

25,62 137 25,62 147 25,62 122 25,62 99

25,64 134 25,64 130 25,64 121 25,64 110

25,66 137 25,66 131 25,66 123 25,66 107

25,68 132 25,68 117 25,68 104 25,68 87

25,7 113 25,7 98 25,7 113 25,7 101

25,72 115 25,72 97 25,72 128 25,72 108

25,74 135 25,74 100 25,74 107 25,74 93

25,76 122 25,76 105 25,76 121 25,76 82

25,78 125 25,78 106 25,78 123 25,78 99

25,8 123 25,8 114 25,8 129 25,8 84

25,82 130 25,82 91 25,82 106 25,82 80

25,84 121 25,84 94 25,84 118 25,84 79

25,86 133 25,86 84 25,86 121 25,86 113

25,88 134 25,88 79 25,88 129 25,88 92

25,9 126 25,9 88 25,9 121 25,9 95

25,92 130 25,92 82 25,92 124 25,92 98

25,94 155 25,94 91 25,94 136 25,94 105

25,96 131 25,96 77 25,96 124 25,96 87

25,98 137 25,98 86 25,98 117 25,98 99

26 121 26 99 26 125 26 89

26,02 131 26,02 85 26,02 145 26,02 100

26,04 137 26,04 75 26,04 130 26,04 95

26,06 144 26,06 84 26,06 109 26,06 109

26,08 134 26,08 86 26,08 119 26,08 105

26,1 125 26,1 86 26,1 118 26,1 106

26,12 130 26,12 74 26,12 128 26,12 107

26,14 111 26,14 84 26,14 110 26,14 109

26,16 129 26,16 79 26,16 127 26,16 96

26,18 128 26,18 105 26,18 134 26,18 99

26,2 124 26,2 84 26,2 110 26,2 109

26,22 116 26,22 97 26,22 136 26,22 134

26,24 128 26,24 101 26,24 126 26,24 115

26,26 126 26,26 92 26,26 116 26,26 121

26,28 139 26,28 124 26,28 121 26,28 140

26,3 152 26,3 110 26,3 112 26,3 119

26,32 168 26,32 137 26,32 141 26,32 142

26,34 174 26,34 138 26,34 130 26,34 148

26,36 178 26,36 131 26,36 149 26,36 152

26,38 172 26,38 110 26,38 148 26,38 146

26,4 209 26,4 125 26,4 149 26,4 131

26,42 164 26,42 152 26,42 133 26,42 118

26,44 168 26,44 164 26,44 129 26,44 111

26,46 151 26,46 152 26,46 139 26,46 107

26,48 134 26,48 138 26,48 131 26,48 115

26,5 143 26,5 128 26,5 110 26,5 107

26,52 133 26,52 132 26,52 129 26,52 97

26,54 127 26,54 108 26,54 114 26,54 102

26,56 135 26,56 91 26,56 93 26,56 117

26,58 132 26,58 86 26,58 142 26,58 123

26,6 120 26,6 88 26,6 120 26,6 130

26,62 131 26,62 79 26,62 103 26,62 100

26,64 113 26,64 91 26,64 124 26,64 86

26,66 123 26,66 74 26,66 133 26,66 111

26,68 135 26,68 71 26,68 119 26,68 84

26,7 123 26,7 93 26,7 116 26,7 91

26,72 125 26,72 70 26,72 125 26,72 95

26,74 144 26,74 88 26,74 114 26,74 97

26,76 117 26,76 77 26,76 105 26,76 89

26,78 105 26,78 96 26,78 105 26,78 97

26,8 132 26,8 96 26,8 114 26,8 97

26,82 120 26,82 101 26,82 125 26,82 106

26,84 136 26,84 87 26,84 101 26,84 105

26,86 124 26,86 92 26,86 120 26,86 88

26,88 96 26,88 105 26,88 118 26,88 111

26,9 126 26,9 86 26,9 112 26,9 110

26,92 126 26,92 102 26,92 123 26,92 101

26,94 116 26,94 100 26,94 108 26,94 88

26,96 117 26,96 83 26,96 129 26,96 101

26,98 107 26,98 83 26,98 121 26,98 96

27 120 27 82 27 113 27 92

27,02 139 27,02 87 27,02 135 27,02 98

27,04 136 27,04 77 27,04 116 27,04 95

27,06 122 27,06 88 27,06 114 27,06 108

27,08 114 27,08 104 27,08 106 27,08 112

27,1 127 27,1 88 27,1 104 27,1 107

27,12 129 27,12 90 27,12 140 27,12 102

27,14 129 27,14 86 27,14 120 27,14 123

27,16 117 27,16 109 27,16 114 27,16 105

27,18 123 27,18 95 27,18 162 27,18 124

27,2 119 27,2 88 27,2 131 27,2 146

27,22 122 27,22 102 27,22 131 27,22 137

27,24 136 27,24 93 27,24 122 27,24 157

27,26 121 27,26 117 27,26 128 27,26 156

27,28 131 27,28 128 27,28 130 27,28 164

27,3 99 27,3 116 27,3 133 27,3 136

27,32 123 27,32 109 27,32 121 27,32 122

27,34 139 27,34 109 27,34 113 27,34 110

27,36 143 27,36 113 27,36 121 27,36 124

27,38 124 27,38 98 27,38 142 27,38 137

27,4 125 27,4 96 27,4 125 27,4 120

27,42 147 27,42 121 27,42 135 27,42 108

27,44 124 27,44 118 27,44 127 27,44 119

27,46 137 27,46 118 27,46 128 27,46 153

27,48 120 27,48 131 27,48 137 27,48 130

27,5 155 27,5 114 27,5 134 27,5 126

27,52 112 27,52 131 27,52 135 27,52 119

27,54 162 27,54 138 27,54 143 27,54 118

27,56 120 27,56 141 27,56 135 27,56 123

27,58 126 27,58 134 27,58 112 27,58 103

27,6 120 27,6 138 27,6 136 27,6 107

27,62 132 27,62 122 27,62 113 27,62 126

27,64 130 27,64 119 27,64 106 27,64 103

27,66 124 27,66 126 27,66 115 27,66 108

27,68 140 27,68 103 27,68 128 27,68 111

27,7 130 27,7 92 27,7 119 27,7 106

27,72 140 27,72 101 27,72 120 27,72 117

27,74 126 27,74 111 27,74 114 27,74 100

27,76 123 27,76 126 27,76 103 27,76 133

27,78 120 27,78 93 27,78 141 27,78 110

27,8 141 27,8 101 27,8 114 27,8 98

27,82 122 27,82 118 27,82 98 27,82 94

27,84 128 27,84 88 27,84 144 27,84 84

27,86 145 27,86 90 27,86 140 27,86 122

27,88 143 27,88 87 27,88 135 27,88 110

27,9 108 27,9 84 27,9 122 27,9 110

27,92 120 27,92 89 27,92 142 27,92 91

27,94 121 27,94 91 27,94 128 27,94 123

27,96 144 27,96 99 27,96 121 27,96 116

27,98 121 27,98 88 27,98 137 27,98 92

28 128 28 119 28 149 28 131

28,02 126 28,02 103 28,02 117 28,02 101

28,04 118 28,04 105 28,04 119 28,04 111

28,06 131 28,06 100 28,06 107 28,06 124

28,08 136 28,08 112 28,08 110 28,08 105

28,1 128 28,1 86 28,1 126 28,1 125

28,12 130 28,12 109 28,12 112 28,12 101

28,14 125 28,14 97 28,14 149 28,14 99

28,16 158 28,16 95 28,16 128 28,16 96

28,18 117 28,18 86 28,18 112 28,18 116

28,2 146 28,2 92 28,2 114 28,2 95

28,22 93 28,22 100 28,22 131 28,22 125

28,24 149 28,24 81 28,24 139 28,24 122

28,26 117 28,26 103 28,26 97 28,26 120

28,28 108 28,28 105 28,28 150 28,28 93

28,3 115 28,3 105 28,3 141 28,3 122

28,32 124 28,32 92 28,32 121 28,32 128

28,34 117 28,34 100 28,34 137 28,34 130

28,36 111 28,36 90 28,36 123 28,36 110

28,38 113 28,38 79 28,38 116 28,38 121

28,4 119 28,4 95 28,4 141 28,4 123

28,42 134 28,42 97 28,42 125 28,42 118

28,44 125 28,44 106 28,44 122 28,44 119

28,46 147 28,46 116 28,46 139 28,46 99

28,48 147 28,48 106 28,48 116 28,48 108

28,5 117 28,5 96 28,5 132 28,5 122

28,52 154 28,52 116 28,52 124 28,52 125

28,54 142 28,54 98 28,54 120 28,54 122

28,56 136 28,56 108 28,56 136 28,56 139

28,58 148 28,58 116 28,58 147 28,58 112

28,6 159 28,6 115 28,6 136 28,6 130

28,62 182 28,62 124 28,62 150 28,62 137

28,64 209 28,64 125 28,64 145 28,64 150

28,66 167 28,66 145 28,66 138 28,66 131

28,68 152 28,68 165 28,68 133 28,68 129

28,7 159 28,7 134 28,7 110 28,7 128

28,72 162 28,72 146 28,72 145 28,72 160

28,74 125 28,74 121 28,74 132 28,74 170

28,76 133 28,76 147 28,76 113 28,76 116

28,78 137 28,78 138 28,78 120 28,78 111

28,8 152 28,8 129 28,8 127 28,8 136

28,82 142 28,82 119 28,82 127 28,82 157

28,84 124 28,84 134 28,84 146 28,84 139

28,86 157 28,86 112 28,86 165 28,86 150

28,88 128 28,88 140 28,88 145 28,88 150

28,9 154 28,9 158 28,9 144 28,9 126

28,92 132 28,92 136 28,92 132 28,92 153

28,94 141 28,94 122 28,94 155 28,94 167

28,96 140 28,96 149 28,96 117 28,96 168

28,98 150 28,98 174 28,98 117 28,98 207

29 172 29 151 29 129 29 247

29,02 142 29,02 163 29,02 128 29,02 337

29,04 151 29,04 184 29,04 127 29,04 434

29,06 205 29,06 194 29,06 130 29,06 448

29,08 184 29,08 242 29,08 181 29,08 343

29,1 166 29,1 226 29,1 192 29,1 298

29,12 169 29,12 266 29,12 206 29,12 288

29,14 139 29,14 268 29,14 157 29,14 295

29,16 169 29,16 304 29,16 135 29,16 166

29,18 125 29,18 258 29,18 145 29,18 151

29,2 136 29,2 202 29,2 134 29,2 146

29,22 124 29,22 182 29,22 124 29,22 119

29,24 135 29,24 155 29,24 107 29,24 119

29,26 126 29,26 141 29,26 128 29,26 99

29,28 130 29,28 118 29,28 130 29,28 129

29,3 120 29,3 94 29,3 129 29,3 101

29,32 100 29,32 87 29,32 118 29,32 95

29,34 116 29,34 100 29,34 118 29,34 100

29,36 102 29,36 76 29,36 111 29,36 106

29,38 109 29,38 90 29,38 103 29,38 92

29,4 113 29,4 90 29,4 138 29,4 108

29,42 142 29,42 114 29,42 111 29,42 105

29,44 99 29,44 85 29,44 130 29,44 107

29,46 124 29,46 87 29,46 134 29,46 94

29,48 113 29,48 81 29,48 134 29,48 120

29,5 119 29,5 84 29,5 120 29,5 115

29,52 119 29,52 114 29,52 120 29,52 127

29,54 118 29,54 92 29,54 143 29,54 122

29,56 132 29,56 98 29,56 124 29,56 86

29,58 117 29,58 90 29,58 113 29,58 128

29,6 130 29,6 108 29,6 104 29,6 101

29,62 141 29,62 94 29,62 100 29,62 100

29,64 147 29,64 103 29,64 104 29,64 101

29,66 127 29,66 101 29,66 105 29,66 96

29,68 133 29,68 87 29,68 121 29,68 86

29,7 128 29,7 100 29,7 104 29,7 106

29,72 122 29,72 125 29,72 115 29,72 84

29,74 135 29,74 98 29,74 105 29,74 117

29,76 132 29,76 91 29,76 103 29,76 106

29,78 91 29,78 84 29,78 104 29,78 95

29,8 100 29,8 110 29,8 120 29,8 104

29,82 115 29,82 96 29,82 106 29,82 113

29,84 99 29,84 92 29,84 105 29,84 98

29,86 124 29,86 96 29,86 122 29,86 116

29,88 122 29,88 107 29,88 125 29,88 97

29,9 102 29,9 101 29,9 110 29,9 114

29,92 125 29,92 83 29,92 159 29,92 111

29,94 123 29,94 78 29,94 132 29,94 100

29,96 123 29,96 79 29,96 115 29,96 116

29,98 110 29,98 107 29,98 121 29,98 106

30 116 30 107 30 137 30 104

| La@Sm 10% | | La@Eu 10% | | La@Tb 10% | | La@Dy 10% | |
| --- | --- | --- | --- | --- | --- | --- | --- |
| 2θ | I (A.U) | 2θ | I (A.U) | 2θ | I (A.U) | 2θ | I (A.U) |

3 595 3 574 3 537 3 605

3,02 595 3,02 547 3,02 519 3,02 558

3,04 580 3,04 549 3,04 528 3,04 605

3,06 617 3,06 552 3,06 532 3,06 562

3,08 623 3,08 550 3,08 536 3,08 607

3,1 644 3,1 553 3,1 501 3,1 562

3,12 633 3,12 550 3,12 545 3,12 562

3,14 684 3,14 581 3,14 514 3,14 582

3,16 754 3,16 574 3,16 544 3,16 562

3,18 696 3,18 547 3,18 505 3,18 551

3,2 718 3,2 572 3,2 526 3,2 646

3,22 748 3,22 611 3,22 511 3,22 646

3,24 759 3,24 641 3,24 501 3,24 652

3,26 675 3,26 617 3,26 546 3,26 604

3,28 556 3,28 587 3,28 522 3,28 564

3,3 609 3,3 558 3,3 502 3,3 532

3,32 524 3,32 509 3,32 474 3,32 575

3,34 566 3,34 500 3,34 514 3,34 532

3,36 509 3,36 485 3,36 644 3,36 593

3,38 485 3,38 490 3,38 841 3,38 551

3,4 446 3,4 487 3,4 843 3,4 555

3,42 500 3,42 489 3,42 771 3,42 565

3,44 472 3,44 488 3,44 598 3,44 575

3,46 522 3,46 470 3,46 573 3,46 655

3,48 474 3,48 463 3,48 682 3,48 742

3,5 529 3,5 507 3,5 753 3,5 866

3,52 511 3,52 524 3,52 699 3,52 926

3,54 495 3,54 520 3,54 733 3,54 967

3,56 597 3,56 593 3,56 1200 3,56 985

3,58 585 3,58 614 3,58 2082 3,58 1051

3,6 680 3,6 648 3,6 2896 3,6 992

3,62 760 3,62 721 3,62 2386 3,62 855

3,64 708 3,64 956 3,64 1388 3,64 576

3,66 577 3,66 1069 3,66 1383 3,66 475

3,68 449 3,68 1018 3,68 1127 3,68 499

3,7 444 3,7 587 3,7 662 3,7 411

3,72 442 3,72 474 3,72 594 3,72 449

3,74 438 3,74 461 3,74 449 3,74 395

3,76 385 3,76 424 3,76 394 3,76 425

3,78 337 3,78 371 3,78 367 3,78 421

3,8 395 3,8 378 3,8 387 3,8 431

3,82 361 3,82 402 3,82 401 3,82 403

3,84 371 3,84 377 3,84 374 3,84 399

3,86 372 3,86 364 3,86 346 3,86 373

3,88 348 3,88 373 3,88 386 3,88 396

3,9 369 3,9 372 3,9 371 3,9 368

3,92 399 3,92 370 3,92 361 3,92 401

3,94 358 3,94 346 3,94 387 3,94 389

3,96 330 3,96 367 3,96 369 3,96 395

3,98 319 3,98 366 3,98 363 3,98 388

4 317 4 376 4 357 4 379

4,02 331 4,02 349 4,02 342 4,02 383

4,04 334 4,04 372 4,04 328 4,04 382

4,06 354 4,06 333 4,06 325 4,06 356

4,08 325 4,08 327 4,08 363 4,08 381

4,1 326 4,1 347 4,1 342 4,1 391

4,12 308 4,12 341 4,12 376 4,12 363

4,14 327 4,14 331 4,14 360 4,14 353

4,16 331 4,16 318 4,16 317 4,16 335

4,18 309 4,18 329 4,18 349 4,18 358

4,2 330 4,2 354 4,2 348 4,2 316

4,22 339 4,22 328 4,22 308 4,22 346

4,24 320 4,24 314 4,24 282 4,24 324

4,26 290 4,26 307 4,26 319 4,26 336

4,28 339 4,28 309 4,28 362 4,28 352

4,3 310 4,3 334 4,3 322 4,3 337

4,32 286 4,32 327 4,32 301 4,32 323

4,34 314 4,34 329 4,34 290 4,34 310

4,36 312 4,36 326 4,36 300 4,36 301

4,38 309 4,38 294 4,38 326 4,38 339

4,4 302 4,4 335 4,4 302 4,4 319

4,42 289 4,42 305 4,42 303 4,42 323

4,44 296 4,44 328 4,44 313 4,44 300

4,46 273 4,46 313 4,46 318 4,46 318

4,48 296 4,48 290 4,48 309 4,48 344

4,5 314 4,5 325 4,5 291 4,5 328

4,52 277 4,52 291 4,52 309 4,52 309

4,54 302 4,54 307 4,54 265 4,54 351

4,56 255 4,56 285 4,56 294 4,56 334

4,58 297 4,58 299 4,58 311 4,58 317

4,6 262 4,6 289 4,6 277 4,6 310

4,62 298 4,62 285 4,62 275 4,62 332

4,64 307 4,64 290 4,64 295 4,64 318

4,66 280 4,66 284 4,66 281 4,66 297

4,68 291 4,68 282 4,68 289 4,68 299

4,7 291 4,7 321 4,7 280 4,7 276

4,72 275 4,72 296 4,72 269 4,72 292

4,74 274 4,74 288 4,74 282 4,74 283

4,76 269 4,76 262 4,76 287 4,76 291

4,78 268 4,78 266 4,78 284 4,78 296

4,8 262 4,8 315 4,8 299 4,8 310

4,82 274 4,82 299 4,82 281 4,82 283

4,84 273 4,84 254 4,84 265 4,84 283

4,86 244 4,86 269 4,86 279 4,86 332

4,88 270 4,88 291 4,88 268 4,88 308

4,9 288 4,9 240 4,9 277 4,9 297

4,92 271 4,92 248 4,92 295 4,92 284

4,94 271 4,94 243 4,94 275 4,94 294

4,96 272 4,96 285 4,96 293 4,96 270

4,98 284 4,98 271 4,98 278 4,98 290

5 268 5 250 5 266 5 265

5,02 251 5,02 278 5,02 296 5,02 239

5,04 268 5,04 256 5,04 280 5,04 267

5,06 252 5,06 252 5,06 270 5,06 294

5,08 245 5,08 271 5,08 272 5,08 312

5,1 255 5,1 279 5,1 288 5,1 282

5,12 243 5,12 275 5,12 269 5,12 278

5,14 270 5,14 247 5,14 248 5,14 260

5,16 247 5,16 241 5,16 278 5,16 280

5,18 228 5,18 233 5,18 257 5,18 306

5,2 267 5,2 287 5,2 242 5,2 293

5,22 253 5,22 231 5,22 271 5,22 249

5,24 246 5,24 263 5,24 275 5,24 271

5,26 255 5,26 264 5,26 254 5,26 256

5,28 231 5,28 244 5,28 257 5,28 275

5,3 273 5,3 261 5,3 253 5,3 265

5,32 239 5,32 230 5,32 260 5,32 256

5,34 242 5,34 241 5,34 255 5,34 249

5,36 228 5,36 248 5,36 246 5,36 257

5,38 238 5,38 230 5,38 253 5,38 245

5,4 276 5,4 239 5,4 260 5,4 279

5,42 234 5,42 247 5,42 239 5,42 268

5,44 247 5,44 221 5,44 265 5,44 273

5,46 250 5,46 232 5,46 236 5,46 245

5,48 236 5,48 212 5,48 242 5,48 244

5,5 231 5,5 233 5,5 268 5,5 240

5,52 241 5,52 258 5,52 237 5,52 261

5,54 239 5,54 249 5,54 240 5,54 228

5,56 226 5,56 254 5,56 244 5,56 264

5,58 238 5,58 247 5,58 243 5,58 279

5,6 214 5,6 226 5,6 245 5,6 239

5,62 230 5,62 239 5,62 214 5,62 225

5,64 217 5,64 247 5,64 235 5,64 274

5,66 197 5,66 213 5,66 245 5,66 240

5,68 216 5,68 243 5,68 240 5,68 238

5,7 223 5,7 246 5,7 244 5,7 264

5,72 204 5,72 241 5,72 256 5,72 227

5,74 233 5,74 253 5,74 232 5,74 252

5,76 210 5,76 204 5,76 218 5,76 249

5,78 182 5,78 211 5,78 246 5,78 245

5,8 221 5,8 242 5,8 260 5,8 243

5,82 214 5,82 220 5,82 224 5,82 252

5,84 231 5,84 204 5,84 246 5,84 251

5,86 218 5,86 264 5,86 227 5,86 242

5,88 224 5,88 223 5,88 232 5,88 251

5,9 243 5,9 237 5,9 234 5,9 240

5,92 230 5,92 211 5,92 225 5,92 219

5,94 241 5,94 229 5,94 230 5,94 241

5,96 225 5,96 205 5,96 251 5,96 247

5,98 209 5,98 234 5,98 232 5,98 231

6 233 6 210 6 245 6 227

6,02 209 6,02 206 6,02 214 6,02 241

6,04 216 6,04 214 6,04 242 6,04 223

6,06 222 6,06 206 6,06 202 6,06 232

6,08 224 6,08 206 6,08 252 6,08 214

6,1 232 6,1 213 6,1 209 6,1 253

6,12 230 6,12 206 6,12 199 6,12 216

6,14 255 6,14 214 6,14 239 6,14 246

6,16 247 6,16 192 6,16 227 6,16 245

6,18 251 6,18 238 6,18 225 6,18 234

6,2 242 6,2 222 6,2 256 6,2 250

6,22 263 6,22 209 6,22 244 6,22 223

6,24 236 6,24 240 6,24 202 6,24 227

6,26 240 6,26 204 6,26 238 6,26 221

6,28 257 6,28 250 6,28 226 6,28 201

6,3 255 6,3 256 6,3 206 6,3 213

6,32 254 6,32 216 6,32 231 6,32 224

6,34 290 6,34 261 6,34 212 6,34 219

6,36 295 6,36 240 6,36 215 6,36 218

6,38 275 6,38 264 6,38 237 6,38 236

6,4 304 6,4 272 6,4 193 6,4 216

6,42 311 6,42 271 6,42 197 6,42 243

6,44 278 6,44 274 6,44 233 6,44 221

6,46 243 6,46 222 6,46 193 6,46 206

6,48 246 6,48 206 6,48 188 6,48 216

6,5 219 6,5 240 6,5 230 6,5 219

6,52 229 6,52 216 6,52 212 6,52 200

6,54 220 6,54 213 6,54 201 6,54 207

6,56 240 6,56 182 6,56 190 6,56 195

6,58 190 6,58 219 6,58 215 6,58 237

6,6 224 6,6 200 6,6 232 6,6 191

6,62 175 6,62 241 6,62 195 6,62 219

6,64 194 6,64 190 6,64 211 6,64 214

6,66 218 6,66 185 6,66 215 6,66 211

6,68 175 6,68 189 6,68 201 6,68 201

6,7 230 6,7 190 6,7 196 6,7 192

6,72 194 6,72 210 6,72 183 6,72 205

6,74 191 6,74 190 6,74 191 6,74 208

6,76 190 6,76 193 6,76 231 6,76 183

6,78 192 6,78 165 6,78 202 6,78 197

6,8 205 6,8 183 6,8 268 6,8 211

6,82 217 6,82 223 6,82 252 6,82 196

6,84 186 6,84 202 6,84 267 6,84 222

6,86 170 6,86 175 6,86 207 6,86 213

6,88 198 6,88 188 6,88 189 6,88 197

6,9 208 6,9 191 6,9 205 6,9 212

6,92 170 6,92 194 6,92 268 6,92 237

6,94 187 6,94 204 6,94 267 6,94 232

6,96 187 6,96 198 6,96 324 6,96 232

6,98 209 6,98 195 6,98 498 6,98 224

7 205 7 222 7 782 7 243

7,02 193 7,02 229 7,02 851 7,02 261

7,04 188 7,04 227 7,04 649 7,04 273

7,06 186 7,06 207 7,06 351 7,06 280

7,08 209 7,08 206 7,08 387 7,08 292

7,1 229 7,1 235 7,1 394 7,1 302

7,12 209 7,12 247 7,12 421 7,12 313

7,14 265 7,14 245 7,14 968 7,14 370

7,16 310 7,16 258 7,16 2248 7,16 418

7,18 360 7,18 315 7,18 3233 7,18 475

7,2 410 7,2 317 7,2 2635 7,2 429

7,22 382 7,22 345 7,22 1061 7,22 410

7,24 292 7,24 472 7,24 628 7,24 278

7,26 252 7,26 494 7,26 549 7,26 220

7,28 227 7,28 430 7,28 443 7,28 170

7,3 234 7,3 281 7,3 379 7,3 198

7,32 232 7,32 211 7,32 329 7,32 190

7,34 182 7,34 164 7,34 245 7,34 170

7,36 186 7,36 175 7,36 202 7,36 169

7,38 170 7,38 179 7,38 191 7,38 173

7,4 184 7,4 174 7,4 175 7,4 196

7,42 167 7,42 198 7,42 194 7,42 177

7,44 172 7,44 171 7,44 188 7,44 178

7,46 146 7,46 174 7,46 185 7,46 173

7,48 143 7,48 161 7,48 167 7,48 180

7,5 180 7,5 172 7,5 167 7,5 190

7,52 175 7,52 199 7,52 193 7,52 166

7,54 145 7,54 182 7,54 160 7,54 181

7,56 163 7,56 175 7,56 188 7,56 146

7,58 172 7,58 154 7,58 183 7,58 167

7,6 163 7,6 160 7,6 157 7,6 167

7,62 162 7,62 151 7,62 186 7,62 171

7,64 174 7,64 161 7,64 152 7,64 187

7,66 183 7,66 156 7,66 166 7,66 174

7,68 158 7,68 174 7,68 187 7,68 169

7,7 171 7,7 155 7,7 181 7,7 155

7,72 181 7,72 153 7,72 188 7,72 172

7,74 180 7,74 164 7,74 170 7,74 184

7,76 171 7,76 166 7,76 163 7,76 161

7,78 168 7,78 171 7,78 195 7,78 173

7,8 177 7,8 141 7,8 162 7,8 178

7,82 144 7,82 183 7,82 170 7,82 162

7,84 175 7,84 151 7,84 156 7,84 172

7,86 175 7,86 157 7,86 198 7,86 167

7,88 175 7,88 156 7,88 175 7,88 146

7,9 166 7,9 151 7,9 165 7,9 173

7,92 145 7,92 168 7,92 185 7,92 177

7,94 144 7,94 167 7,94 180 7,94 157

7,96 142 7,96 150 7,96 168 7,96 183

7,98 143 7,98 178 7,98 174 7,98 174

8 163 8 173 8 181 8 158

8,02 158 8,02 161 8,02 156 8,02 172

8,04 168 8,04 156 8,04 176 8,04 166

8,05 155 8,05 158 8,05 182 8,05 156

8,08 146 8,08 166 8,08 166 8,08 169

8,09 188 8,09 155 8,09 155 8,09 150

8,12 179 8,12 177 8,12 160 8,12 169

8,14 163 8,14 166 8,14 173 8,14 180

8,16 153 8,16 150 8,16 158 8,16 147

8,18 142 8,18 150 8,18 173 8,18 146

8,2 153 8,2 172 8,2 172 8,2 178

8,22 142 8,22 143 8,22 162 8,22 158

8,24 146 8,24 173 8,24 175 8,24 175

8,26 139 8,26 145 8,26 178 8,26 173

8,28 142 8,28 159 8,28 187 8,28 171

8,3 153 8,3 179 8,3 169 8,3 160

8,32 148 8,32 174 8,32 166 8,32 164

8,34 151 8,34 170 8,34 181 8,34 174

8,36 162 8,36 145 8,36 163 8,36 163

8,38 141 8,38 136 8,38 151 8,38 157

8,4 155 8,4 169 8,4 176 8,4 143

8,42 150 8,42 166 8,42 161 8,42 157

8,44 147 8,44 173 8,44 159 8,44 160

8,46 159 8,46 116 8,46 153 8,46 171

8,48 160 8,48 143 8,48 154 8,48 153

8,5 163 8,5 156 8,5 147 8,5 141

8,52 130 8,52 153 8,52 159 8,52 156

8,54 161 8,54 144 8,54 157 8,54 175

8,55 170 8,55 131 8,55 153 8,55 166

8,58 136 8,58 151 8,58 155 8,58 151

8,59 149 8,59 134 8,59 145 8,59 177

8,62 173 8,62 175 8,62 159 8,62 176

8,64 161 8,64 136 8,64 165 8,64 146

8,66 132 8,66 153 8,66 169 8,66 166

8,68 144 8,68 149 8,68 137 8,68 153

8,7 141 8,7 162 8,7 134 8,7 152

8,72 129 8,72 154 8,72 152 8,72 170

8,74 137 8,74 139 8,74 144 8,74 146

8,76 142 8,76 123 8,76 164 8,76 150

8,78 156 8,78 145 8,78 160 8,78 151

8,8 137 8,8 152 8,8 154 8,8 145

8,82 141 8,82 150 8,82 165 8,82 169

8,84 155 8,84 145 8,84 146 8,84 139

8,86 167 8,86 157 8,86 168 8,86 145

8,88 160 8,88 142 8,88 144 8,88 155

8,9 156 8,9 158 8,9 179 8,9 149

8,92 142 8,92 136 8,92 166 8,92 162

8,94 160 8,94 157 8,94 153 8,94 145

8,96 129 8,96 140 8,96 169 8,96 178

8,98 124 8,98 157 8,98 131 8,98 154

9 156 9 166 9 151 9 167

9,02 144 9,02 139 9,02 153 9,02 162

9,04 142 9,04 136 9,04 154 9,04 161

9,05 143 9,05 124 9,05 123 9,05 153

9,08 119 9,08 144 9,08 167 9,08 159

9,09 158 9,09 151 9,09 161 9,09 143

9,12 150 9,12 137 9,12 140 9,12 163

9,13 139 9,13 147 9,13 132 9,13 146

9,16 159 9,16 149 9,16 150 9,16 151

9,18 139 9,18 155 9,18 163 9,18 155

9,2 147 9,2 174 9,2 147 9,2 156

9,22 126 9,22 145 9,22 166 9,22 144

9,24 147 9,24 155 9,24 147 9,24 150

9,26 153 9,26 169 9,26 132 9,26 131

9,28 148 9,28 141 9,28 157 9,28 164

9,3 141 9,3 138 9,3 141 9,3 135

9,32 154 9,32 149 9,32 165 9,32 133

9,34 164 9,34 152 9,34 163 9,34 144

9,36 150 9,36 153 9,36 165 9,36 165

9,38 154 9,38 130 9,38 163 9,38 139

9,4 160 9,4 153 9,4 156 9,4 151

9,42 153 9,42 157 9,42 135 9,42 142

9,44 180 9,44 155 9,44 159 9,44 174

9,46 164 9,46 150 9,46 172 9,46 146

9,48 171 9,48 160 9,48 154 9,48 169

9,5 162 9,5 169 9,5 123 9,5 151

9,52 206 9,52 167 9,52 135 9,52 141

9,54 177 9,54 168 9,54 177 9,54 124

9,55 195 9,55 185 9,55 130 9,55 161

9,58 201 9,58 166 9,58 142 9,58 145

9,59 192 9,59 165 9,59 156 9,59 149

9,62 193 9,62 186 9,62 150 9,62 133

9,63 202 9,63 149 9,63 165 9,63 142

9,66 155 9,66 173 9,66 137 9,66 153

9,68 175 9,68 149 9,68 149 9,68 142

9,7 163 9,7 172 9,7 156 9,7 149

9,72 159 9,72 133 9,72 158 9,72 148

9,74 152 9,74 170 9,74 142 9,74 132

9,76 144 9,76 157 9,76 123 9,76 132

9,78 147 9,78 130 9,78 165 9,78 139

9,8 150 9,8 120 9,8 154 9,8 147

9,82 145 9,82 136 9,82 143 9,82 129

9,84 145 9,84 146 9,84 154 9,84 153

9,86 143 9,86 115 9,86 138 9,86 126

9,88 126 9,88 158 9,88 128 9,88 150

9,9 141 9,9 138 9,9 135 9,9 121

9,92 128 9,92 148 9,92 146 9,92 129

9,94 158 9,94 146 9,94 135 9,94 134

9,96 140 9,96 139 9,96 144 9,96 139

9,98 133 9,98 129 9,98 145 9,98 145

10 168 10 142 10 144 10 144

10,02 153 10,02 138 10,02 163 10,02 149

10,04 131 10,04 159 10,04 131 10,04 140

10,06 138 10,06 151 10,06 125 10,06 133

10,08 149 10,08 175 10,08 158 10,08 139

10,1 144 10,1 175 10,1 149 10,1 132

10,12 157 10,12 143 10,12 138 10,12 129

10,14 126 10,14 142 10,14 145 10,14 123

10,16 141 10,16 146 10,16 150 10,16 141

10,18 147 10,18 123 10,18 153 10,18 138

10,2 158 10,2 145 10,2 143 10,2 138

10,22 140 10,22 146 10,22 144 10,22 127

10,24 164 10,24 136 10,24 144 10,24 134

10,26 158 10,26 152 10,26 135 10,26 140

10,28 167 10,28 130 10,28 174 10,28 146

10,3 141 10,3 146 10,3 131 10,3 143

10,32 140 10,32 135 10,32 140 10,32 163

10,34 141 10,34 141 10,34 139 10,34 166

10,36 124 10,36 158 10,36 154 10,36 137

10,38 137 10,38 139 10,38 143 10,38 141

10,4 131 10,4 162 10,4 151 10,4 143

10,42 159 10,42 131 10,42 158 10,42 130

10,44 145 10,44 163 10,44 151 10,44 164

10,46 158 10,46 138 10,46 165 10,46 175

10,48 144 10,48 145 10,48 163 10,48 179

10,5 139 10,5 142 10,5 181 10,5 177

10,52 158 10,52 132 10,52 200 10,52 171

10,54 163 10,54 147 10,54 359 10,54 174

10,56 155 10,56 132 10,56 480 10,56 156

10,58 140 10,58 136 10,58 567 10,58 167

10,6 159 10,6 134 10,6 434 10,6 171

10,62 144 10,62 159 10,62 334 10,62 165

10,64 136 10,64 141 10,64 208 10,64 205

10,66 149 10,66 142 10,66 195 10,66 224

10,68 153 10,68 159 10,68 271 10,68 221

10,7 157 10,7 182 10,7 316 10,7 262

10,72 216 10,72 168 10,72 453 10,72 255

10,74 291 10,74 188 10,74 1054 10,74 323

10,76 386 10,76 192 10,76 1622 10,76 327

10,78 398 10,78 192 10,78 1690 10,78 335

10,8 381 10,8 260 10,8 1147 10,8 335

10,82 309 10,82 354 10,82 725 10,82 305

10,84 282 10,84 434 10,84 571 10,84 193

10,86 208 10,86 482 10,86 388 10,86 170

10,88 195 10,88 381 10,88 332 10,88 125

10,9 211 10,9 272 10,9 278 10,9 125

10,92 164 10,92 177 10,92 200 10,92 121

10,94 134 10,94 167 10,94 173 10,94 122

10,96 145 10,96 140 10,96 152 10,96 107

10,98 138 10,98 162 10,98 136 10,98 125

11 128 11 118 11 155 11 133

11,02 109 11,02 139 11,02 143 11,02 126

11,04 134 11,04 125 11,04 144 11,04 106

11,06 122 11,06 117 11,06 115 11,06 115

11,08 121 11,08 108 11,08 129 11,08 125

11,1 124 11,1 121 11,1 136 11,1 124

11,12 132 11,12 134 11,12 124 11,12 134

11,14 130 11,14 114 11,14 133 11,14 114

11,16 122 11,16 129 11,16 131 11,16 128

11,18 120 11,18 106 11,18 136 11,18 123

11,2 124 11,2 125 11,2 122 11,2 126

11,22 98 11,22 121 11,22 136 11,22 130

11,24 125 11,24 114 11,24 127 11,24 122

11,26 121 11,26 116 11,26 122 11,26 114

11,28 120 11,28 116 11,28 129 11,28 132

11,3 119 11,3 115 11,3 125 11,3 112

11,32 142 11,32 131 11,32 126 11,32 128

11,34 117 11,34 100 11,34 130 11,34 103

11,36 103 11,36 125 11,36 128 11,36 127

11,38 115 11,38 112 11,38 123 11,38 121

11,4 124 11,4 106 11,4 128 11,4 129

11,42 128 11,42 105 11,42 115 11,42 128

11,44 95 11,44 129 11,44 118 11,44 118

11,46 124 11,46 105 11,46 125 11,46 111

11,48 108 11,48 132 11,48 116 11,48 136

11,5 117 11,5 132 11,5 145 11,5 129

11,52 126 11,52 102 11,52 129 11,52 118

11,54 98 11,54 138 11,54 112 11,54 110

11,56 116 11,56 110 11,56 134 11,56 135

11,58 100 11,58 103 11,58 117 11,58 106

11,6 114 11,6 128 11,6 122 11,6 114

11,62 114 11,62 133 11,62 109 11,62 119

11,64 111 11,64 128 11,64 126 11,64 125

11,66 107 11,66 98 11,66 130 11,66 135

11,68 132 11,68 134 11,68 113 11,68 148

11,7 105 11,7 98 11,7 108 11,7 135

11,72 115 11,72 98 11,72 157 11,72 124

11,74 128 11,74 111 11,74 125 11,74 133

11,76 107 11,76 112 11,76 144 11,76 117

11,78 119 11,78 109 11,78 115 11,78 119

11,8 112 11,8 121 11,8 136 11,8 133

11,82 121 11,82 106 11,82 114 11,82 123

11,84 105 11,84 113 11,84 111 11,84 127

11,86 121 11,86 124 11,86 150 11,86 125

11,88 129 11,88 102 11,88 117 11,88 116

11,9 108 11,9 122 11,9 129 11,9 122

11,92 111 11,92 97 11,92 148 11,92 116

11,94 123 11,94 94 11,94 127 11,94 112

11,96 120 11,96 115 11,96 122 11,96 116

11,98 103 11,98 111 11,98 123 11,98 119

12 114 12 127 12 121 12 127

12,02 105 12,02 128 12,02 131 12,02 128

12,04 95 12,04 105 12,04 121 12,04 131

12,06 122 12,06 119 12,06 118 12,06 94

12,08 108 12,08 117 12,08 129 12,08 135

12,1 114 12,1 89 12,1 149 12,1 113

12,12 108 12,12 116 12,12 136 12,12 125

12,14 107 12,14 111 12,14 114 12,14 124

12,16 131 12,16 111 12,16 125 12,16 122

12,18 124 12,18 109 12,18 120 12,18 112

12,2 117 12,2 108 12,2 121 12,2 124

12,22 98 12,22 112 12,22 135 12,22 137

12,24 114 12,24 118 12,24 125 12,24 115

12,26 90 12,26 97 12,26 137 12,26 116

12,28 120 12,28 114 12,28 125 12,28 131

12,3 87 12,3 101 12,3 141 12,3 135

12,32 92 12,32 104 12,32 111 12,32 128

12,34 108 12,34 114 12,34 102 12,34 126

12,36 108 12,36 127 12,36 115 12,36 109

12,38 109 12,38 99 12,38 108 12,38 112

12,4 102 12,4 136 12,4 130 12,4 126

12,42 94 12,42 114 12,42 104 12,42 140

12,44 119 12,44 106 12,44 122 12,44 128

12,46 110 12,46 104 12,46 124 12,46 96

12,48 96 12,48 114 12,48 108 12,48 109

12,5 133 12,5 108 12,5 122 12,5 113

12,52 114 12,52 108 12,52 106 12,52 143

12,54 130 12,54 111 12,54 109 12,54 111

12,56 115 12,56 117 12,56 117 12,56 135

12,58 111 12,58 110 12,58 112 12,58 120

12,6 115 12,6 104 12,6 140 12,6 100

12,62 127 12,62 104 12,62 129 12,62 120

12,64 119 12,64 123 12,64 137 12,64 136

12,66 117 12,66 104 12,66 127 12,66 105

12,68 105 12,68 117 12,68 108 12,68 112

12,7 146 12,7 109 12,7 118 12,7 117

12,72 129 12,72 112 12,72 112 12,72 115

12,74 119 12,74 110 12,74 112 12,74 95

12,76 137 12,76 115 12,76 124 12,76 117

12,78 138 12,78 124 12,78 116 12,78 108

12,8 141 12,8 117 12,8 115 12,8 107

12,82 126 12,82 143 12,82 122 12,82 121

12,84 132 12,84 128 12,84 104 12,84 92

12,86 123 12,86 115 12,86 112 12,86 114

12,88 125 12,88 139 12,88 111 12,88 109

12,9 106 12,9 128 12,9 101 12,9 105

12,92 120 12,92 117 12,92 128 12,92 107

12,94 119 12,94 121 12,94 121 12,94 125

12,96 116 12,96 104 12,96 119 12,96 109

12,98 131 12,98 106 12,98 107 12,98 97

13 115 13 137 13 126 13 118

13,02 107 13,02 111 13,02 122 13,02 127

13,04 107 13,04 94 13,04 112 13,04 97

13,06 89 13,06 119 13,06 139 13,06 104

13,08 102 13,08 102 13,08 120 13,08 104

13,1 115 13,1 102 13,1 103 13,1 94

13,12 95 13,12 107 13,12 112 13,12 120

13,14 111 13,14 112 13,14 122 13,14 126

13,16 117 13,16 101 13,16 130 13,16 108

13,18 113 13,18 100 13,18 124 13,18 97

13,2 96 13,2 84 13,2 97 13,2 106

13,22 110 13,22 120 13,22 124 13,22 118

13,24 88 13,24 114 13,24 95 13,24 113

13,26 103 13,26 89 13,26 130 13,26 108

13,28 108 13,28 101 13,28 120 13,28 105

13,3 120 13,3 112 13,3 108 13,3 117

13,32 104 13,32 104 13,32 140 13,32 99

13,34 101 13,34 114 13,34 113 13,34 98

13,36 123 13,36 126 13,36 121 13,36 86

13,38 109 13,38 90 13,38 111 13,38 111

13,4 90 13,4 124 13,4 129 13,4 106

13,42 104 13,42 93 13,42 101 13,42 96

13,44 92 13,44 112 13,44 124 13,44 111

13,46 106 13,46 106 13,46 130 13,46 103

13,48 97 13,48 116 13,48 109 13,48 117

13,5 92 13,5 114 13,5 108 13,5 112

13,52 113 13,52 85 13,52 107 13,52 89

13,54 103 13,54 86 13,54 116 13,54 115

13,56 96 13,56 106 13,56 110 13,56 121

13,58 105 13,58 107 13,58 125 13,58 99

13,6 125 13,6 98 13,6 121 13,6 109

13,62 121 13,62 104 13,62 122 13,62 108

13,64 111 13,64 117 13,64 96 13,64 106

13,66 89 13,66 102 13,66 127 13,66 93

13,68 106 13,68 113 13,68 104 13,68 113

13,7 107 13,7 92 13,7 114 13,7 96

13,72 114 13,72 109 13,72 115 13,72 109

13,74 119 13,74 99 13,74 119 13,74 120

13,76 102 13,76 101 13,76 112 13,76 108

13,78 93 13,78 123 13,78 107 13,78 108

13,8 117 13,8 118 13,8 124 13,8 108

13,82 115 13,82 121 13,82 140 13,82 109

13,84 90 13,84 104 13,84 98 13,84 102

13,86 99 13,86 109 13,86 110 13,86 126

13,88 93 13,88 96 13,88 127 13,88 106

13,9 120 13,9 116 13,9 130 13,9 111

13,92 106 13,92 94 13,92 120 13,92 99

13,94 98 13,94 112 13,94 135 13,94 113

13,96 104 13,96 115 13,96 111 13,96 108

13,98 96 13,98 101 13,98 107 13,98 108

14 97 14 76 14 123 14 127

14,02 115 14,02 98 14,02 113 14,02 113

14,04 97 14,04 93 14,04 127 14,04 114

14,06 92 14,06 110 14,06 115 14,06 126

14,08 87 14,08 99 14,08 118 14,08 114

14,1 80 14,1 111 14,1 152 14,1 130

14,12 127 14,12 81 14,12 162 14,12 124

14,14 84 14,14 103 14,14 171 14,14 102

14,16 122 14,16 92 14,16 207 14,16 104

14,18 121 14,18 113 14,18 169 14,18 94

14,2 97 14,2 130 14,2 146 14,2 124

14,22 102 14,22 105 14,22 141 14,22 127

14,24 113 14,24 131 14,24 163 14,24 109

14,26 112 14,26 97 14,26 169 14,26 120

14,28 101 14,28 93 14,28 154 14,28 141

14,3 127 14,3 103 14,3 225 14,3 143

14,32 132 14,32 132 14,32 225 14,32 165

14,34 113 14,34 113 14,34 299 14,34 165

14,36 163 14,36 111 14,36 391 14,36 187

14,38 155 14,38 123 14,38 408 14,38 193

14,4 160 14,4 154 14,4 310 14,4 175

14,42 171 14,42 180 14,42 280 14,42 169

14,44 164 14,44 184 14,44 191 14,44 137

14,46 171 14,46 212 14,46 227 14,46 125

14,48 121 14,48 242 14,48 247 14,48 107

14,5 128 14,5 185 14,5 190 14,5 111

14,52 134 14,52 159 14,52 167 14,52 118

14,54 120 14,54 126 14,54 144 14,54 101

14,56 89 14,56 103 14,56 130 14,56 98

14,58 115 14,58 102 14,58 116 14,58 99

14,6 108 14,6 98 14,6 122 14,6 94

14,62 107 14,62 106 14,62 138 14,62 123

14,64 91 14,64 111 14,64 139 14,64 104

14,66 110 14,66 93 14,66 115 14,66 106

14,68 99 14,68 101 14,68 115 14,68 91

14,7 116 14,7 105 14,7 116 14,7 103

14,72 98 14,72 91 14,72 116 14,72 106

14,74 105 14,74 90 14,74 120 14,74 101

14,76 94 14,76 102 14,76 113 14,76 91

14,78 91 14,78 111 14,78 135 14,78 113

14,8 81 14,8 94 14,8 125 14,8 103

14,82 86 14,82 98 14,82 118 14,82 117

14,84 96 14,84 94 14,84 107 14,84 95

14,86 101 14,86 95 14,86 126 14,86 116

14,88 97 14,88 102 14,88 121 14,88 124

14,9 89 14,9 105 14,9 106 14,9 111

14,92 93 14,92 103 14,92 123 14,92 111

14,94 104 14,94 124 14,94 110 14,94 108

14,96 118 14,96 83 14,96 124 14,96 109

14,98 123 14,98 103 14,98 131 14,98 105

15 108 15 105 15 123 15 135

15,02 103 15,02 99 15,02 121 15,02 121

15,04 118 15,04 86 15,04 136 15,04 131

15,06 95 15,06 95 15,06 140 15,06 135

15,08 111 15,08 109 15,08 109 15,08 155

15,1 110 15,1 117 15,1 120 15,1 167

15,12 103 15,12 126 15,12 126 15,12 190

15,14 95 15,14 106 15,14 124 15,14 202

15,16 102 15,16 90 15,16 156 15,16 177

15,18 101 15,18 110 15,18 157 15,18 190

15,2 108 15,2 119 15,2 160 15,2 165

15,22 95 15,22 112 15,22 144 15,22 181

15,24 108 15,24 122 15,24 160 15,24 168

15,26 111 15,26 118 15,26 144 15,26 174

15,28 125 15,28 113 15,28 138 15,28 168

15,3 105 15,3 115 15,3 138 15,3 178

15,32 89 15,32 121 15,32 154 15,32 174

15,34 104 15,34 114 15,34 142 15,34 170

15,36 130 15,36 122 15,36 132 15,36 134

15,38 123 15,38 116 15,38 142 15,38 152

15,4 102 15,4 130 15,4 112 15,4 163

15,42 97 15,42 146 15,42 128 15,42 136

15,44 112 15,44 125 15,44 125 15,44 100

15,46 106 15,46 131 15,46 139 15,46 99

15,48 83 15,48 88 15,48 113 15,48 109

15,5 98 15,5 99 15,5 128 15,5 120

15,52 117 15,52 102 15,52 117 15,52 112

15,54 105 15,54 110 15,54 123 15,54 103

15,56 117 15,56 108 15,56 109 15,56 117

15,58 97 15,58 127 15,58 113 15,58 110

15,6 109 15,6 95 15,6 125 15,6 97

15,62 127 15,62 100 15,62 132 15,62 124

15,64 102 15,64 89 15,64 121 15,64 124

15,66 105 15,66 115 15,66 142 15,66 125

15,68 111 15,68 96 15,68 128 15,68 121

15,7 106 15,7 102 15,7 121 15,7 141

15,72 117 15,72 124 15,72 129 15,72 131

15,74 111 15,74 98 15,74 122 15,74 123

15,76 116 15,76 96 15,76 98 15,76 127

15,78 98 15,78 109 15,78 139 15,78 141

15,8 101 15,8 107 15,8 123 15,8 146

15,82 100 15,82 101 15,82 127 15,82 144

15,84 116 15,84 104 15,84 146 15,84 144

15,86 107 15,86 156 15,86 144 15,86 156

15,88 103 15,88 145 15,88 127 15,88 132

15,9 103 15,9 151 15,9 136 15,9 125

15,92 117 15,92 139 15,92 147 15,92 111

15,94 101 15,94 107 15,94 115 15,94 112

15,96 104 15,96 120 15,96 132 15,96 141

15,98 107 15,98 99 15,98 120 15,98 121

16 124 16 100 16 101 16 106

16,02 116 16,02 117 16,02 118 16,02 98

16,04 144 16,04 124 16,04 125 16,04 127

16,06 109 16,06 110 16,06 118 16,06 109

16,08 104 16,08 116 16,08 143 16,08 117

16,1 116 16,1 103 16,1 134 16,1 109

16,12 117 16,12 115 16,12 95 16,12 113

16,14 113 16,14 95 16,14 129 16,14 124

16,16 108 16,16 94 16,16 98 16,16 109

16,18 86 16,18 108 16,18 154 16,18 101

16,2 81 16,2 107 16,2 139 16,2 116

16,22 116 16,22 91 16,22 129 16,22 135

16,24 102 16,24 108 16,24 129 16,24 133

16,26 96 16,26 109 16,26 119 16,26 135

16,28 97 16,28 110 16,28 128 16,28 138

16,3 90 16,3 103 16,3 129 16,3 113

16,32 91 16,32 106 16,32 154 16,32 124

16,34 89 16,34 98 16,34 189 16,34 105

16,36 88 16,36 112 16,36 193 16,36 125

16,38 90 16,38 105 16,38 155 16,38 118

16,4 82 16,4 111 16,4 184 16,4 111

16,42 104 16,42 119 16,42 118 16,42 112

16,44 89 16,44 110 16,44 140 16,44 108

16,46 79 16,46 100 16,46 145 16,46 105

16,48 115 16,48 100 16,48 133 16,48 123

16,5 91 16,5 97 16,5 130 16,5 120

16,52 97 16,52 97 16,52 132 16,52 138

16,54 114 16,54 112 16,54 124 16,54 136

16,56 109 16,56 98 16,56 144 16,56 128

16,58 123 16,58 104 16,58 142 16,58 149

16,6 130 16,6 110 16,6 133 16,6 160

16,62 102 16,62 111 16,62 132 16,62 158

16,64 112 16,64 97 16,64 127 16,64 153

16,66 104 16,66 92 16,66 147 16,66 141

16,68 99 16,68 99 16,68 133 16,68 129

16,7 105 16,7 117 16,7 122 16,7 149

16,72 94 16,72 123 16,72 141 16,72 117

16,74 101 16,74 103 16,74 127 16,74 105

16,76 107 16,76 103 16,76 142 16,76 115

16,78 103 16,78 104 16,78 103 16,78 105

16,8 110 16,8 124 16,8 127 16,8 117

16,82 112 16,82 115 16,82 132 16,82 99

16,84 103 16,84 140 16,84 110 16,84 124

16,86 110 16,86 160 16,86 127 16,86 118

16,88 101 16,88 169 16,88 139 16,88 111

16,9 102 16,9 138 16,9 135 16,9 151

16,92 91 16,92 148 16,92 117 16,92 140

16,94 93 16,94 122 16,94 116 16,94 147

16,96 103 16,96 108 16,96 153 16,96 147

16,98 106 16,98 124 16,98 124 16,98 157

17 112 17 121 17 133 17 158

17,02 104 17,02 116 17,02 140 17,02 187

17,04 99 17,04 140 17,04 142 17,04 170

17,06 84 17,06 125 17,06 145 17,06 165

17,08 106 17,08 137 17,08 136 17,08 160

17,1 92 17,1 147 17,1 131 17,1 152

17,12 111 17,12 125 17,12 139 17,12 155

17,14 94 17,14 118 17,14 156 17,14 124

17,16 109 17,16 123 17,16 145 17,16 131

17,18 98 17,18 130 17,18 141 17,18 140

17,2 99 17,2 121 17,2 152 17,2 138

17,22 110 17,22 138 17,22 133 17,22 134

17,24 115 17,24 98 17,24 142 17,24 130

17,26 118 17,26 100 17,26 120 17,26 148

17,28 117 17,28 114 17,28 129 17,28 143

17,3 123 17,3 110 17,3 134 17,3 138

17,32 100 17,32 113 17,32 135 17,32 141

17,34 116 17,34 127 17,34 126 17,34 125

17,36 114 17,36 127 17,36 118 17,36 126

17,38 119 17,38 124 17,38 140 17,38 126

17,4 101 17,4 122 17,4 124 17,4 109

17,42 117 17,42 94 17,42 112 17,42 103

17,44 106 17,44 84 17,44 99 17,44 131

17,46 95 17,46 97 17,46 143 17,46 122

17,48 101 17,48 111 17,48 113 17,48 121

17,5 109 17,5 96 17,5 126 17,5 92

17,52 114 17,52 88 17,52 136 17,52 114

17,54 107 17,54 90 17,54 118 17,54 121

17,56 100 17,56 95 17,56 157 17,56 108

17,58 109 17,58 109 17,58 122 17,58 105

17,6 88 17,6 97 17,6 126 17,6 105

17,62 82 17,62 98 17,62 128 17,62 114

17,64 89 17,64 131 17,64 163 17,64 129

17,66 98 17,66 107 17,66 140 17,66 122

17,68 101 17,68 102 17,68 148 17,68 127

17,7 107 17,7 101 17,7 126 17,7 112

17,72 107 17,72 107 17,72 158 17,72 101

17,74 99 17,74 114 17,74 160 17,74 121

17,76 100 17,76 113 17,76 158 17,76 130

17,78 123 17,78 101 17,78 175 17,78 133

17,8 107 17,8 106 17,8 197 17,8 147

17,82 123 17,82 93 17,82 220 17,82 137

17,84 106 17,84 120 17,84 226 17,84 172

17,86 116 17,86 135 17,86 241 17,86 158

17,88 127 17,88 118 17,88 221 17,88 189

17,9 135 17,9 154 17,9 261 17,9 208

17,92 150 17,92 148 17,92 261 17,92 206

17,94 150 17,94 120 17,94 303 17,94 275

17,96 190 17,96 160 17,96 350 17,96 299

17,98 200 17,98 173 17,98 350 17,98 315

18 219 18 199 18 395 18 329

18,02 208 18,02 205 18,02 404 18,02 356

18,04 252 18,04 244 18,04 403 18,04 298

18,06 253 18,06 329 18,06 366 18,06 248

18,08 217 18,08 414 18,08 418 18,08 222

18,1 181 18,1 465 18,1 442 18,1 179

18,12 207 18,12 395 18,12 344 18,12 165

18,14 132 18,14 302 18,14 321 18,14 141

18,16 135 18,16 263 18,16 238 18,16 145

18,18 152 18,18 189 18,18 197 18,18 122

18,2 110 18,2 156 18,2 153 18,2 102

18,22 123 18,22 142 18,22 149 18,22 120

18,24 125 18,24 147 18,24 150 18,24 149

18,26 130 18,26 138 18,26 154 18,26 108

18,28 113 18,28 133 18,28 146 18,28 117

18,3 126 18,3 136 18,3 124 18,3 115

18,32 126 18,32 122 18,32 134 18,32 116

18,34 139 18,34 131 18,34 139 18,34 124

18,36 129 18,36 136 18,36 152 18,36 111

18,38 120 18,38 129 18,38 132 18,38 102

18,4 121 18,4 127 18,4 135 18,4 108

18,42 109 18,42 132 18,42 121 18,42 96

18,44 102 18,44 103 18,44 127 18,44 111

18,46 123 18,46 106 18,46 146 18,46 118

18,48 131 18,48 131 18,48 131 18,48 103

18,5 92 18,5 119 18,5 130 18,5 100

18,52 100 18,52 123 18,52 135 18,52 117

18,54 100 18,54 90 18,54 149 18,54 102

18,56 94 18,56 109 18,56 145 18,56 102

18,58 111 18,58 94 18,58 143 18,58 96

18,6 112 18,6 130 18,6 133 18,6 112

18,62 121 18,62 108 18,62 117 18,62 101

18,64 120 18,64 96 18,64 129 18,64 121

18,66 120 18,66 108 18,66 117 18,66 102

18,68 125 18,68 98 18,68 115 18,68 130

18,7 111 18,7 119 18,7 130 18,7 114

18,72 97 18,72 114 18,72 100 18,72 105

18,74 104 18,74 131 18,74 122 18,74 99

18,76 105 18,76 97 18,76 136 18,76 83

18,78 94 18,78 120 18,78 125 18,78 96

18,8 120 18,8 106 18,8 139 18,8 97

18,82 108 18,82 108 18,82 130 18,82 119

18,84 104 18,84 83 18,84 133 18,84 111

18,86 96 18,86 98 18,86 117 18,86 122

18,88 88 18,88 99 18,88 123 18,88 105

18,9 97 18,9 112 18,9 128 18,9 107

18,92 122 18,92 108 18,92 136 18,92 86

18,94 98 18,94 88 18,94 134 18,94 99

18,96 99 18,96 119 18,96 124 18,96 111

18,98 93 18,98 87 18,98 151 18,98 101

19 115 19 129 19 140 19 100

19,02 110 19,02 101 19,02 144 19,02 112

19,04 97 19,04 99 19,04 142 19,04 128

19,06 94 19,06 100 19,06 167 19,06 134

19,08 121 19,08 98 19,08 148 19,08 97

19,1 116 19,1 127 19,1 130 19,1 121

19,12 106 19,12 112 19,12 145 19,12 139

19,14 104 19,14 108 19,14 128 19,14 126

19,16 91 19,16 108 19,16 131 19,16 117

19,18 98 19,18 114 19,18 150 19,18 139

19,2 110 19,2 90 19,2 142 19,2 120

19,22 121 19,22 106 19,22 122 19,22 138

19,24 107 19,24 106 19,24 140 19,24 111

19,26 104 19,26 121 19,26 144 19,26 118

19,28 126 19,28 111 19,28 118 19,28 115

19,3 93 19,3 94 19,3 152 19,3 155

19,32 116 19,32 108 19,32 152 19,32 126

19,34 107 19,34 118 19,34 152 19,34 124

19,36 106 19,36 146 19,36 138 19,36 123

19,38 125 19,38 132 19,38 138 19,38 113

19,4 97 19,4 116 19,4 151 19,4 109

19,42 104 19,42 112 19,42 140 19,42 112

19,44 106 19,44 105 19,44 139 19,44 118

19,46 105 19,46 95 19,46 152 19,46 125

19,48 114 19,48 111 19,48 143 19,48 119

19,5 101 19,5 101 19,5 141 19,5 121

19,52 129 19,52 115 19,52 155 19,52 112

19,54 95 19,54 96 19,54 127 19,54 108

19,56 128 19,56 112 19,56 124 19,56 126

19,58 100 19,58 108 19,58 136 19,58 127

19,6 98 19,6 113 19,6 141 19,6 158

19,62 109 19,62 116 19,62 111 19,62 133

19,64 98 19,64 112 19,64 143 19,64 119

19,66 94 19,66 100 19,66 143 19,66 145

19,68 97 19,68 101 19,68 154 19,68 149

19,7 112 19,7 112 19,7 161 19,7 161

19,72 113 19,72 126 19,72 142 19,72 161

19,74 97 19,74 102 19,74 146 19,74 163

19,76 119 19,76 114 19,76 147 19,76 193

19,78 127 19,78 116 19,78 151 19,78 173

19,8 123 19,8 160 19,8 149 19,8 195

19,82 114 19,82 143 19,82 169 19,82 201

19,84 108 19,84 125 19,84 177 19,84 221

19,86 118 19,86 144 19,86 162 19,86 239

19,88 129 19,88 144 19,88 165 19,88 198

19,9 116 19,9 157 19,9 146 19,9 218

19,92 122 19,92 142 19,92 151 19,92 186

19,94 133 19,94 138 19,94 170 19,94 214

19,96 130 19,96 154 19,96 154 19,96 220

19,98 108 19,98 124 19,98 156 19,98 233

20 117 20 146 20 134 20 238

20,02 130 20,02 134 20,02 163 20,02 228

20,04 141 20,04 153 20,04 189 20,04 218

20,06 115 20,06 164 20,06 182 20,06 203

20,08 105 20,08 132 20,08 157 20,08 161

20,1 112 20,1 159 20,1 170 20,1 149

20,12 107 20,12 145 20,12 158 20,12 161

20,14 114 20,14 138 20,14 166 20,14 146

20,16 122 20,16 122 20,16 162 20,16 163

20,18 111 20,18 137 20,18 180 20,18 147

20,2 113 20,2 115 20,2 147 20,2 148

20,22 112 20,22 117 20,22 188 20,22 144

20,24 115 20,24 120 20,24 177 20,24 125

20,26 110 20,26 101 20,26 182 20,26 160

20,28 102 20,28 121 20,28 188 20,28 128

20,3 119 20,3 124 20,3 169 20,3 145

20,32 133 20,32 124 20,32 152 20,32 155

20,34 118 20,34 101 20,34 164 20,34 135

20,36 127 20,36 113 20,36 162 20,36 136

20,38 96 20,38 105 20,38 150 20,38 112

20,4 100 20,4 107 20,4 145 20,4 120

20,42 111 20,42 115 20,42 156 20,42 85

20,44 109 20,44 107 20,44 167 20,44 114

20,46 99 20,46 99 20,46 152 20,46 99

20,48 93 20,48 102 20,48 128 20,48 112

20,5 95 20,5 103 20,5 141 20,5 123

20,52 87 20,52 107 20,52 143 20,52 107

20,54 110 20,54 111 20,54 143 20,54 109

20,56 107 20,56 107 20,56 142 20,56 105

20,58 99 20,58 104 20,58 132 20,58 111

20,6 102 20,6 113 20,6 148 20,6 93

20,62 102 20,62 113 20,62 122 20,62 113

20,64 101 20,64 108 20,64 151 20,64 109

20,66 102 20,66 102 20,66 146 20,66 99

20,68 99 20,68 110 20,68 134 20,68 89

20,7 93 20,7 118 20,7 141 20,7 114

20,72 109 20,72 93 20,72 149 20,72 112

20,74 107 20,74 108 20,74 141 20,74 99

20,76 102 20,76 113 20,76 153 20,76 138

20,78 107 20,78 109 20,78 152 20,78 114

20,8 121 20,8 112 20,8 135 20,8 128

20,82 125 20,82 107 20,82 146 20,82 108

20,84 122 20,84 110 20,84 146 20,84 94

20,86 113 20,86 103 20,86 159 20,86 101

20,88 115 20,88 119 20,88 140 20,88 106

20,9 125 20,9 123 20,9 141 20,9 83

20,92 119 20,92 109 20,92 150 20,92 123

20,94 118 20,94 105 20,94 149 20,94 108

20,96 112 20,96 119 20,96 143 20,96 119

20,98 125 20,98 100 20,98 145 20,98 119

21 121 21 114 21 136 21 109

21,02 107 21,02 115 21,02 124 21,02 139

21,04 113 21,04 114 21,04 164 21,04 100

21,06 125 21,06 105 21,06 148 21,06 109

21,08 99 21,08 111 21,08 134 21,08 128

21,1 101 21,1 116 21,1 137 21,1 111

21,12 107 21,12 109 21,12 135 21,12 129

21,14 133 21,14 138 21,14 143 21,14 129

21,16 100 21,16 107 21,16 155 21,16 136

21,18 113 21,18 117 21,18 155 21,18 161

21,2 97 21,2 121 21,2 169 21,2 161

21,22 99 21,22 122 21,22 161 21,22 138

21,24 97 21,24 116 21,24 149 21,24 165

21,26 114 21,26 118 21,26 147 21,26 191

21,28 128 21,28 141 21,28 163 21,28 182

21,3 135 21,3 121 21,3 167 21,3 189

21,32 118 21,32 131 21,32 141 21,32 213

21,34 119 21,34 139 21,34 166 21,34 262

21,36 117 21,36 129 21,36 151 21,36 343

21,38 142 21,38 122 21,38 174 21,38 382

21,4 128 21,4 165 21,4 201 21,4 430

21,42 119 21,42 156 21,42 204 21,42 342

21,44 124 21,44 173 21,44 226 21,44 297

21,46 125 21,46 191 21,46 231 21,46 271

21,48 126 21,48 212 21,48 221 21,48 235

21,5 128 21,5 174 21,5 218 21,5 182

21,52 126 21,52 158 21,52 197 21,52 161

21,54 114 21,54 150 21,54 221 21,54 168

21,56 116 21,56 167 21,56 205 21,56 156

21,58 103 21,58 142 21,58 178 21,58 141

21,6 143 21,6 128 21,6 179 21,6 150

21,62 136 21,62 148 21,62 196 21,62 164

21,64 134 21,64 142 21,64 190 21,64 179

21,66 154 21,66 162 21,66 196 21,66 174

21,68 136 21,68 153 21,68 212 21,68 204

21,7 162 21,7 169 21,7 218 21,7 168

21,72 186 21,72 213 21,72 234 21,72 151

21,74 147 21,74 203 21,74 272 21,74 142

21,76 140 21,76 219 21,76 214 21,76 130

21,78 128 21,78 176 21,78 209 21,78 141

21,8 137 21,8 178 21,8 206 21,8 146

21,82 120 21,82 161 21,82 166 21,82 127

21,84 127 21,84 158 21,84 200 21,84 118

21,86 116 21,86 122 21,86 173 21,86 138

21,88 130 21,88 126 21,88 185 21,88 132

21,9 99 21,9 108 21,9 210 21,9 130

21,92 102 21,92 130 21,92 185 21,92 110

21,94 119 21,94 119 21,94 163 21,94 154

21,96 118 21,96 123 21,96 171 21,96 138

21,98 101 21,98 117 21,98 170 21,98 90

22 103 22 116 22 159 22 120

22,02 106 22,02 121 22,02 146 22,02 128

22,04 92 22,04 108 22,04 176 22,04 125

22,06 85 22,06 119 22,06 151 22,06 134

22,08 93 22,08 125 22,08 169 22,08 145

22,1 98 22,1 124 22,1 126 22,1 127

22,12 111 22,12 120 22,12 161 22,12 162

22,14 121 22,14 128 22,14 168 22,14 147

22,16 95 22,16 140 22,16 180 22,16 163

22,18 115 22,18 124 22,18 153 22,18 202

22,2 107 22,2 123 22,2 164 22,2 193

22,22 108 22,22 144 22,22 195 22,22 175

22,24 110 22,24 132 22,24 167 22,24 152

22,26 116 22,26 142 22,26 174 22,26 159

22,28 110 22,28 141 22,28 211 22,28 119

22,3 114 22,3 131 22,3 162 22,3 141

22,32 116 22,32 121 22,32 135 22,32 127

22,34 96 22,34 117 22,34 172 22,34 117

22,36 97 22,36 131 22,36 162 22,36 137

22,38 110 22,38 125 22,38 182 22,38 145

22,4 96 22,4 93 22,4 179 22,4 143

22,42 119 22,42 108 22,42 149 22,42 130

22,44 109 22,44 102 22,44 156 22,44 93

22,46 81 22,46 108 22,46 149 22,46 127

22,48 98 22,48 111 22,48 151 22,48 120

22,5 117 22,5 112 22,5 169 22,5 110

22,52 100 22,52 110 22,52 170 22,52 119

22,54 92 22,54 112 22,54 156 22,54 117

22,56 97 22,56 102 22,56 167 22,56 104

22,58 116 22,58 122 22,58 154 22,58 143

22,6 97 22,6 116 22,6 152 22,6 119

22,62 110 22,62 112 22,62 178 22,62 112

22,64 104 22,64 96 22,64 160 22,64 129

22,66 91 22,66 93 22,66 149 22,66 111

22,68 93 22,68 111 22,68 155 22,68 146

22,7 102 22,7 115 22,7 178 22,7 120

22,72 113 22,72 108 22,72 171 22,72 133

22,74 115 22,74 97 22,74 142 22,74 155

22,76 75 22,76 121 22,76 169 22,76 112

22,78 98 22,78 115 22,78 178 22,78 140

22,8 109 22,8 122 22,8 160 22,8 140

22,82 96 22,82 106 22,82 152 22,82 123

22,84 82 22,84 126 22,84 154 22,84 124

22,86 117 22,86 109 22,86 173 22,86 120

22,88 122 22,88 122 22,88 164 22,88 112

22,9 109 22,9 107 22,9 170 22,9 121

22,92 102 22,92 109 22,92 153 22,92 101

22,94 112 22,94 140 22,94 162 22,94 117

22,96 100 22,96 129 22,96 163 22,96 123

22,98 128 22,98 106 22,98 176 22,98 141

23 129 23 117 23 178 23 130

23,02 130 23,02 110 23,02 157 23,02 141

23,04 92 23,04 131 23,04 163 23,04 136

23,06 143 23,06 132 23,06 178 23,06 140

23,08 102 23,08 130 23,08 173 23,08 148

23,1 123 23,1 128 23,1 168 23,1 131

23,12 113 23,12 145 23,12 166 23,12 111

23,14 124 23,14 145 23,14 161 23,14 118

23,16 114 23,16 116 23,16 163 23,16 137

23,18 128 23,18 145 23,18 151 23,18 136

23,2 160 23,2 147 23,2 175 23,2 161

23,22 153 23,22 129 23,22 149 23,22 157

23,24 147 23,24 123 23,24 163 23,24 171

23,26 133 23,26 142 23,26 174 23,26 208

23,28 140 23,28 132 23,28 159 23,28 231

23,3 131 23,3 143 23,3 194 23,3 252

23,32 150 23,32 142 23,32 209 23,32 249

23,34 128 23,34 122 23,34 180 23,34 288

23,36 154 23,36 163 23,36 216 23,36 349

23,38 142 23,38 197 23,38 241 23,38 344

23,4 140 23,4 177 23,4 225 23,4 297

23,42 139 23,42 196 23,42 228 23,42 235

23,44 131 23,44 171 23,44 213 23,44 234

23,46 117 23,46 161 23,46 187 23,46 180

23,48 136 23,48 177 23,48 165 23,48 162

23,5 119 23,5 161 23,5 179 23,5 146

23,52 129 23,52 150 23,52 157 23,52 129

23,54 126 23,54 147 23,54 157 23,54 108

23,56 108 23,56 131 23,56 164 23,56 132

23,58 118 23,58 143 23,58 170 23,58 126

23,6 107 23,6 109 23,6 161 23,6 121

23,62 106 23,62 128 23,62 148 23,62 134

23,64 111 23,64 129 23,64 137 23,64 107

23,66 112 23,66 114 23,66 158 23,66 117

23,68 103 23,68 113 23,68 133 23,68 115

23,7 118 23,7 107 23,7 142 23,7 119

23,72 112 23,72 119 23,72 149 23,72 111

23,74 110 23,74 110 23,74 160 23,74 118

23,76 103 23,76 102 23,76 162 23,76 113

23,78 114 23,78 113 23,78 173 23,78 106

23,8 82 23,8 106 23,8 145 23,8 119

23,82 100 23,82 97 23,82 159 23,82 104

23,84 107 23,84 96 23,84 145 23,84 103

23,86 112 23,86 118 23,86 136 23,86 96

23,88 98 23,88 116 23,88 157 23,88 108

23,9 117 23,9 111 23,9 162 23,9 105

23,92 91 23,92 110 23,92 150 23,92 115

23,94 116 23,94 113 23,94 137 23,94 108

23,96 104 23,96 108 23,96 150 23,96 110

23,98 111 23,98 126 23,98 131 23,98 92

24 106 24 109 24 148 24 121

24,02 105 24,02 104 24,02 149 24,02 134

24,04 137 24,04 110 24,04 147 24,04 117

24,06 124 24,06 108 24,06 130 24,06 129

24,08 108 24,08 110 24,08 153 24,08 133

24,1 112 24,1 124 24,1 148 24,1 128

24,12 91 24,12 109 24,12 158 24,12 139

24,14 116 24,14 115 24,14 126 24,14 119

24,16 121 24,16 113 24,16 180 24,16 130

24,18 117 24,18 122 24,18 151 24,18 104

24,2 118 24,2 127 24,2 140 24,2 123

24,22 116 24,22 100 24,22 169 24,22 122

24,24 112 24,24 104 24,24 171 24,24 112

24,26 104 24,26 114 24,26 164 24,26 143

24,28 118 24,28 97 24,28 144 24,28 123

24,3 123 24,3 148 24,3 147 24,3 114

24,32 118 24,32 129 24,32 156 24,32 147

24,34 134 24,34 125 24,34 155 24,34 121

24,36 117 24,36 117 24,36 152 24,36 157

24,38 130 24,38 139 24,38 140 24,38 149

24,4 140 24,4 153 24,4 141 24,4 170

24,42 121 24,42 139 24,42 177 24,42 177

24,44 129 24,44 133 24,44 168 24,44 163

24,46 169 24,46 143 24,46 156 24,46 187

24,48 122 24,48 196 24,48 168 24,48 187

24,5 140 24,5 167 24,5 142 24,5 165

24,52 116 24,52 156 24,52 179 24,52 149

24,54 113 24,54 134 24,54 153 24,54 126

24,56 118 24,56 153 24,56 125 24,56 135

24,58 120 24,58 131 24,58 188 24,58 132

24,6 121 24,6 130 24,6 150 24,6 123

24,62 115 24,62 134 24,62 143 24,62 133

24,64 123 24,64 124 24,64 172 24,64 114

24,66 117 24,66 109 24,66 152 24,66 127

24,68 111 24,68 123 24,68 153 24,68 133

24,7 114 24,7 120 24,7 158 24,7 118

24,72 100 24,72 124 24,72 136 24,72 126

24,74 109 24,74 123 24,74 157 24,74 151

24,76 93 24,76 113 24,76 154 24,76 172

24,78 92 24,78 117 24,78 162 24,78 217

24,8 98 24,8 122 24,8 148 24,8 189

24,82 105 24,82 110 24,82 142 24,82 222

24,84 98 24,84 125 24,84 148 24,84 193

24,86 103 24,86 130 24,86 145 24,86 211

24,88 114 24,88 151 24,88 176 24,88 182

24,9 96 24,9 155 24,9 158 24,9 154

24,92 107 24,92 128 24,92 185 24,92 158

24,94 115 24,94 131 24,94 165 24,94 156

24,96 89 24,96 140 24,96 186 24,96 164

24,98 128 24,98 118 24,98 161 24,98 179

25 117 25 126 25 171 25 223

25,02 119 25,02 136 25,02 167 25,02 232

25,04 134 25,04 119 25,04 180 25,04 260

25,06 120 25,06 117 25,06 179 25,06 249

25,08 142 25,08 156 25,08 199 25,08 286

25,1 100 25,1 145 25,1 215 25,1 277

25,12 126 25,12 171 25,12 162 25,12 226

25,14 116 25,14 184 25,14 180 25,14 211

25,16 117 25,16 183 25,16 159 25,16 188

25,18 117 25,18 165 25,18 159 25,18 170

25,2 117 25,2 147 25,2 148 25,2 135

25,22 84 25,22 139 25,22 178 25,22 141

25,24 92 25,24 126 25,24 177 25,24 110

25,26 109 25,26 125 25,26 154 25,26 134

25,28 95 25,28 129 25,28 154 25,28 137

25,3 104 25,3 125 25,3 155 25,3 135

25,32 114 25,32 117 25,32 127 25,32 141

25,34 111 25,34 128 25,34 170 25,34 119

25,36 101 25,36 110 25,36 139 25,36 136

25,38 113 25,38 130 25,38 190 25,38 115

25,4 95 25,4 134 25,4 126 25,4 145

25,42 90 25,42 101 25,42 150 25,42 144

25,44 121 25,44 119 25,44 165 25,44 119

25,46 104 25,46 135 25,46 183 25,46 137

25,48 112 25,48 125 25,48 149 25,48 160

25,5 104 25,5 106 25,5 140 25,5 167

25,52 95 25,52 113 25,52 157 25,52 151

25,54 105 25,54 100 25,54 155 25,54 154

25,56 106 25,56 93 25,56 136 25,56 132

25,58 121 25,58 112 25,58 165 25,58 176

25,6 112 25,6 130 25,6 158 25,6 161

25,62 96 25,62 109 25,62 133 25,62 156

25,64 111 25,64 113 25,64 129 25,64 147

25,66 129 25,66 113 25,66 172 25,66 131

25,68 120 25,68 99 25,68 172 25,68 140

25,7 119 25,7 112 25,7 166 25,7 159

25,72 123 25,72 131 25,72 175 25,72 163

25,74 125 25,74 119 25,74 154 25,74 146

25,76 110 25,76 133 25,76 169 25,76 131

25,78 100 25,78 107 25,78 145 25,78 117

25,8 116 25,8 110 25,8 159 25,8 128

25,82 97 25,82 130 25,82 145 25,82 115

25,84 105 25,84 113 25,84 131 25,84 120

25,86 107 25,86 118 25,86 167 25,86 133

25,88 119 25,88 104 25,88 138 25,88 130

25,9 125 25,9 112 25,9 156 25,9 122

25,92 115 25,92 121 25,92 142 25,92 122

25,94 104 25,94 118 25,94 119 25,94 144

25,96 99 25,96 126 25,96 152 25,96 151

25,98 104 25,98 111 25,98 157 25,98 137

26 93 26 117 26 158 26 153

26,02 112 26,02 126 26,02 152 26,02 133

26,04 121 26,04 116 26,04 162 26,04 158

26,06 104 26,06 109 26,06 144 26,06 106

26,08 120 26,08 134 26,08 142 26,08 170

26,1 113 26,1 135 26,1 143 26,1 130

26,12 131 26,12 145 26,12 146 26,12 120

26,14 142 26,14 113 26,14 172 26,14 139

26,16 117 26,16 114 26,16 149 26,16 131

26,18 127 26,18 138 26,18 144 26,18 137

26,2 101 26,2 140 26,2 162 26,2 150

26,22 150 26,22 112 26,22 183 26,22 145

26,24 117 26,24 123 26,24 166 26,24 186

26,26 118 26,26 116 26,26 174 26,26 205

26,28 120 26,28 144 26,28 176 26,28 199

26,3 113 26,3 129 26,3 174 26,3 204

26,32 108 26,32 130 26,32 179 26,32 224

26,34 124 26,34 132 26,34 172 26,34 187

26,36 113 26,36 125 26,36 180 26,36 172

26,38 96 26,38 160 26,38 155 26,38 182

26,4 116 26,4 224 26,4 157 26,4 169

26,42 128 26,42 223 26,42 151 26,42 182

26,44 109 26,44 220 26,44 159 26,44 157

26,46 112 26,46 173 26,46 143 26,46 163

26,48 98 26,48 163 26,48 151 26,48 119

26,5 101 26,5 143 26,5 162 26,5 133

26,52 131 26,52 121 26,52 182 26,52 140

26,54 98 26,54 105 26,54 164 26,54 152

26,56 102 26,56 123 26,56 170 26,56 161

26,58 114 26,58 101 26,58 165 26,58 137

26,6 107 26,6 127 26,6 163 26,6 121

26,62 98 26,62 114 26,62 148 26,62 138

26,64 98 26,64 109 26,64 127 26,64 123

26,66 103 26,66 103 26,66 143 26,66 121

26,68 116 26,68 123 26,68 145 26,68 144

26,7 121 26,7 101 26,7 170 26,7 125

26,72 113 26,72 113 26,72 145 26,72 145

26,74 103 26,74 124 26,74 160 26,74 122

26,76 118 26,76 106 26,76 128 26,76 135

26,78 112 26,78 120 26,78 148 26,78 146

26,8 125 26,8 110 26,8 159 26,8 120

26,82 109 26,82 117 26,82 154 26,82 120

26,84 136 26,84 111 26,84 153 26,84 119

26,86 126 26,86 121 26,86 154 26,86 110

26,88 116 26,88 121 26,88 138 26,88 120

26,9 123 26,9 105 26,9 153 26,9 140

26,92 121 26,92 117 26,92 137 26,92 128

26,94 122 26,94 127 26,94 134 26,94 135

26,96 145 26,96 123 26,96 149 26,96 118

26,98 121 26,98 131 26,98 136 26,98 163

27 168 27 127 27 155 27 114

27,02 150 27,02 142 27,02 137 27,02 134

27,04 152 27,04 148 27,04 128 27,04 125

27,06 153 27,06 139 27,06 128 27,06 129

27,08 138 27,08 141 27,08 152 27,08 135

27,1 153 27,1 143 27,1 130 27,1 124

27,12 157 27,12 156 27,12 131 27,12 135

27,14 176 27,14 160 27,14 149 27,14 156

27,16 171 27,16 135 27,16 156 27,16 127

27,18 148 27,18 135 27,18 155 27,18 123

27,2 155 27,2 163 27,2 158 27,2 166

27,22 172 27,22 154 27,22 185 27,22 163

27,24 179 27,24 142 27,24 153 27,24 145

27,26 181 27,26 161 27,26 143 27,26 158

27,28 167 27,28 160 27,28 141 27,28 151

27,3 204 27,3 173 27,3 151 27,3 154

27,32 170 27,32 177 27,32 119 27,32 193

27,34 198 27,34 186 27,34 137 27,34 220

27,36 204 27,36 160 27,36 140 27,36 206

27,38 228 27,38 155 27,38 143 27,38 180

27,4 176 27,4 175 27,4 151 27,4 211

27,42 210 27,42 161 27,42 149 27,42 191

27,44 208 27,44 169 27,44 174 27,44 305

27,46 199 27,46 146 27,46 179 27,46 376

27,48 204 27,48 186 27,48 170 27,48 351

27,5 173 27,5 220 27,5 191 27,5 299

27,52 154 27,52 191 27,52 157 27,52 275

27,54 154 27,54 221 27,54 173 27,54 248

27,56 166 27,56 221 27,56 147 27,56 224

27,58 149 27,58 159 27,58 164 27,58 194

27,6 150 27,6 186 27,6 158 27,6 168

27,62 153 27,62 176 27,62 145 27,62 142

27,64 138 27,64 164 27,64 174 27,64 152

27,66 158 27,66 156 27,66 155 27,66 167

27,68 134 27,68 130 27,68 155 27,68 147

27,7 140 27,7 131 27,7 170 27,7 163

27,72 123 27,72 145 27,72 161 27,72 161

27,74 114 27,74 132 27,74 162 27,74 150

27,76 139 27,76 148 27,76 127 27,76 166

27,78 124 27,78 133 27,78 166 27,78 140

27,8 117 27,8 104 27,8 155 27,8 146

27,82 120 27,82 139 27,82 142 27,82 157

27,84 116 27,84 130 27,84 158 27,84 155

27,86 122 27,86 113 27,86 149 27,86 137

27,88 87 27,88 127 27,88 146 27,88 145

27,9 120 27,9 114 27,9 144 27,9 150

27,92 140 27,92 137 27,92 148 27,92 147

27,94 130 27,94 121 27,94 141 27,94 165

27,96 143 27,96 125 27,96 122 27,96 160

27,98 110 27,98 118 27,98 146 27,98 145

28 127 28 119 28 159 28 155

28,02 111 28,02 134 28,02 179 28,02 152

28,04 117 28,04 127 28,04 158 28,04 118

28,06 133 28,06 137 28,06 148 28,06 147

28,08 144 28,08 138 28,08 154 28,08 159

28,1 149 28,1 153 28,1 151 28,1 156

28,12 128 28,12 141 28,12 153 28,12 148

28,14 143 28,14 141 28,14 176 28,14 140

28,16 143 28,16 130 28,16 145 28,16 124

28,18 147 28,18 131 28,18 160 28,18 149

28,2 140 28,2 153 28,2 152 28,2 142

28,22 162 28,22 148 28,22 170 28,22 165

28,24 152 28,24 144 28,24 162 28,24 164

28,26 142 28,26 145 28,26 156 28,26 134

28,28 156 28,28 132 28,28 171 28,28 154

28,3 157 28,3 132 28,3 134 28,3 157

28,32 149 28,32 126 28,32 156 28,32 152

28,34 163 28,34 134 28,34 157 28,34 149

28,36 149 28,36 152 28,36 141 28,36 166

28,38 142 28,38 113 28,38 157 28,38 143

28,4 165 28,4 147 28,4 144 28,4 161

28,42 132 28,42 156 28,42 154 28,42 151

28,44 122 28,44 141 28,44 157 28,44 156

28,46 127 28,46 141 28,46 160 28,46 186

28,48 151 28,48 137 28,48 172 28,48 172

28,5 129 28,5 126 28,5 179 28,5 179

28,52 146 28,52 154 28,52 169 28,52 193

28,54 145 28,54 142 28,54 164 28,54 251

28,56 156 28,56 147 28,56 159 28,56 307

28,58 126 28,58 157 28,58 170 28,58 369

28,6 131 28,6 140 28,6 162 28,6 288

28,62 131 28,62 138 28,62 219 28,62 240

28,64 161 28,64 153 28,64 223 28,64 253

28,66 149 28,66 170 28,66 204 28,66 242

28,68 141 28,68 155 28,68 146 28,68 213

28,7 126 28,7 142 28,7 164 28,7 175

28,72 126 28,72 134 28,72 189 28,72 137

28,74 134 28,74 129 28,74 169 28,74 167

28,76 124 28,76 136 28,76 141 28,76 152

28,78 122 28,78 136 28,78 145 28,78 167

28,8 137 28,8 122 28,8 129 28,8 180

28,82 120 28,82 131 28,82 158 28,82 196

28,84 129 28,84 129 28,84 155 28,84 186

28,86 133 28,86 135 28,86 162 28,86 294

28,88 121 28,88 124 28,88 138 28,88 253

28,9 144 28,9 146 28,9 184 28,9 226

28,92 138 28,92 132 28,92 173 28,92 176

28,94 154 28,94 156 28,94 176 28,94 217

28,96 106 28,96 204 28,96 169 28,96 196

28,98 130 28,98 249 28,98 180 28,98 141

29 149 29 251 29 148 29 161

29,02 129 29,02 219 29,02 176 29,02 169

29,04 130 29,04 177 29,04 184 29,04 154

29,06 144 29,06 208 29,06 168 29,06 135

29,08 148 29,08 218 29,08 184 29,08 135

29,1 146 29,1 183 29,1 166 29,1 136

29,12 152 29,12 160 29,12 186 29,12 159

29,14 151 29,14 137 29,14 156 29,14 128

29,16 144 29,16 144 29,16 153 29,16 142

29,18 146 29,18 149 29,18 150 29,18 128

29,2 128 29,2 143 29,2 153 29,2 116

29,22 147 29,22 150 29,22 144 29,22 133

29,24 118 29,24 141 29,24 142 29,24 131

29,26 131 29,26 137 29,26 149 29,26 114

29,28 109 29,28 134 29,28 132 29,28 128

29,3 146 29,3 146 29,3 142 29,3 128

29,32 142 29,32 159 29,32 151 29,32 113

29,34 142 29,34 141 29,34 134 29,34 129

29,36 140 29,36 147 29,36 151 29,36 119

29,38 127 29,38 113 29,38 133 29,38 137

29,4 123 29,4 119 29,4 147 29,4 144

29,42 149 29,42 114 29,42 152 29,42 175

29,44 147 29,44 142 29,44 149 29,44 156

29,46 121 29,46 134 29,46 127 29,46 137

29,48 125 29,48 152 29,48 144 29,48 164

29,5 116 29,5 132 29,5 151 29,5 169

29,52 108 29,52 111 29,52 138 29,52 176

29,54 122 29,54 142 29,54 152 29,54 169

29,56 127 29,56 126 29,56 158 29,56 156

29,58 129 29,58 120 29,58 135 29,58 170

29,6 114 29,6 117 29,6 142 29,6 148

29,62 111 29,62 133 29,62 144 29,62 161

29,64 118 29,64 133 29,64 160 29,64 153

29,66 105 29,66 132 29,66 166 29,66 142

29,68 128 29,68 129 29,68 148 29,68 133

29,7 124 29,7 108 29,7 138 29,7 146

29,72 128 29,72 110 29,72 152 29,72 136

29,74 110 29,74 95 29,74 153 29,74 130

29,76 124 29,76 113 29,76 135 29,76 146

29,78 103 29,78 137 29,78 123 29,78 134

29,8 104 29,8 114 29,8 132 29,8 125

29,82 104 29,82 143 29,82 146 29,82 131

29,84 98 29,84 119 29,84 154 29,84 124

29,86 107 29,86 127 29,86 142 29,86 115

29,88 120 29,88 127 29,88 134 29,88 102

29,9 116 29,9 129 29,9 146 29,9 124

29,92 111 29,92 109 29,92 164 29,92 135

29,94 123 29,94 129 29,94 142 29,94 136

29,96 101 29,96 109 29,96 152 29,96 122

29,98 117 29,98 114 29,98 126 29,98 121

30 102 30 102 30 141 30 130

Table S4 – S5 show the raw data of the FT-IR from Figures 4 - 5.

| La(1) | | Pr(2) | |
| --- | --- | --- | --- |
| Wave number (cm^-1^) | I (A.U) | Wave number (cm^-1^) | I (A.U) |

642,3 0,90246 640,37 0,94871

644,22882 0,90212 642,29882 0,94475

646,15763 0,9023 644,22763 0,94058

648,08645 0,90236 646,15645 0,93652

650,01526 0,90255 648,08526 0,93324

651,94408 0,90198 650,01408 0,92962

653,8729 0,90018 651,9429 0,92568

655,80171 0,89794 653,87171 0,92123

657,73053 0,8951 655,80053 0,91591

659,65934 0,89216 657,72934 0,91046

661,58816 0,88967 659,65816 0,90472

663,51698 0,88774 661,58698 0,89809

665,44579 0,88685 663,51579 0,8913

667,37461 0,88665 665,44461 0,88521

669,30342 0,88706 667,37342 0,87861

671,23224 0,88695 669,30224 0,87106

673,16106 0,88639 671,23106 0,8625

675,08987 0,88465 673,15987 0,85204

677,01869 0,88213 675,08869 0,84035

678,9475 0,87915 677,0175 0,82855

680,87632 0,87579 678,94632 0,81532

682,80514 0,8718 680,87514 0,80249

684,73395 0,86637 682,80395 0,78927

686,66277 0,85907 684,73277 0,77581

688,59158 0,84987 686,66158 0,76219

690,5204 0,83796 688,5904 0,74689

692,44922 0,82284 690,51922 0,73111

694,37803 0,80536 692,44803 0,71502

696,30685 0,78673 694,37685 0,69889

698,23566 0,76839 696,30566 0,68364

700,16448 0,75156 698,23448 0,66887

702,0933 0,73711 700,1633 0,65492

704,02211 0,72605 702,09211 0,64312

705,95093 0,71867 704,02093 0,63291

707,87974 0,71468 705,94974 0,62419

709,80856 0,71424 707,87856 0,61727

711,73738 0,71726 709,80738 0,61208

713,66619 0,72301 711,73619 0,6088

715,59501 0,73097 713,66501 0,60705

717,52382 0,74126 715,59382 0,60686

719,45264 0,75336 717,52264 0,60957

721,38146 0,76715 719,45146 0,61551

723,31027 0,78245 721,38027 0,62419

725,23909 0,79899 723,30909 0,63462

727,1679 0,81589 725,2379 0,64641

729,09672 0,83317 727,16672 0,65857

731,02554 0,85027 729,09554 0,67136

732,95435 0,86743 731,02435 0,68401

734,88317 0,88429 732,95317 0,69584

736,81198 0,90094 734,88198 0,70631

738,7408 0,91651 736,8108 0,71465

740,66962 0,93081 738,73962 0,72019

742,59843 0,94503 740,66843 0,72275

744,52725 0,95725 742,59725 0,72149

746,45606 0,96522 744,52606 0,71593

748,38488 0,96738 746,45488 0,70612

750,3137 0,96125 748,3837 0,69203

752,24251 0,94473 750,31251 0,67419

754,17133 0,91624 752,24133 0,65299

756,10014 0,87572 754,17014 0,62992

758,02896 0,82401 756,09896 0,60638

759,95778 0,7632 758,02778 0,58254

761,88659 0,69621 759,95659 0,5584

763,81541 0,62749 761,88541 0,53467

765,74422 0,56139 763,81422 0,51273

767,67304 0,49928 765,74304 0,4933

769,60186 0,44365 767,67186 0,47724

771,53067 0,39682 769,60067 0,46469

773,45949 0,35891 771,52949 0,45577

775,3883 0,32989 773,4583 0,45066

777,31712 0,30963 775,38712 0,44898

779,24594 0,29804 777,31594 0,45058

781,17475 0,29509 779,24475 0,4553

783,10357 0,30092 781,17357 0,46218

785,03238 0,31523 783,10238 0,4711

786,9612 0,33716 785,0312 0,48209

788,89002 0,36604 786,96002 0,49448

790,81883 0,40366 788,88883 0,50831

792,74765 0,45073 790,81765 0,52338

794,67646 0,50509 792,74646 0,54015

796,60528 0,56326 794,67528 0,55856

798,5341 0,62219 796,6041 0,57737

800,46291 0,67903 798,53291 0,59561

802,39173 0,73137 800,46173 0,61313

804,32054 0,77619 802,39054 0,63001

806,24936 0,80937 804,31936 0,64591

808,17818 0,82865 806,24818 0,66042

810,10699 0,83289 808,17699 0,67276

812,03581 0,82249 810,10581 0,68225

813,96462 0,80121 812,03462 0,68857

815,89344 0,77201 813,96344 0,69113

817,82226 0,73652 815,89226 0,68996

819,75107 0,69754 817,82107 0,68521

821,67989 0,65871 819,74989 0,67718

823,6087 0,62368 821,6787 0,66672

825,53752 0,59494 823,60752 0,65484

827,46634 0,57282 825,53634 0,64226

829,39515 0,55658 827,46515 0,63037

831,32397 0,5467 829,39397 0,61992

833,25278 0,54217 831,32278 0,61194

835,1816 0,54188 833,2516 0,60624

837,11042 0,5459 835,18042 0,60267

839,03923 0,55472 837,10923 0,60206

840,96805 0,56826 839,03805 0,60536

842,89686 0,58662 840,96686 0,61306

844,82568 0,6098 842,89568 0,62481

846,7545 0,63775 844,8245 0,6398

848,68331 0,67009 846,75331 0,65723

850,61213 0,70641 848,68213 0,67635

852,54094 0,74626 850,61094 0,69669

854,46976 0,78635 852,53976 0,71745

856,39858 0,82213 854,46858 0,73824

858,32739 0,85167 856,39739 0,75862

860,25621 0,87443 858,32621 0,77823

862,18502 0,89084 860,25502 0,79694

864,11384 0,90169 862,18384 0,81467

866,04266 0,90843 864,11266 0,8311

867,97147 0,91282 866,04147 0,84648

869,90029 0,91689 867,97029 0,8607

871,8291 0,92267 869,8991 0,87403

873,75792 0,93171 871,82792 0,8866

875,68674 0,9438 873,75674 0,89878

877,61555 0,956 875,68555 0,91069

879,54437 0,96601 877,61437 0,92244

881,47318 0,97409 879,54318 0,93379

883,402 0,98037 881,472 0,94442

885,33082 0,98496 883,40082 0,95394

887,25963 0,98764 885,32963 0,96226

889,18845 0,98846 887,25845 0,96917

891,11726 0,98759 889,18726 0,97473

893,04608 0,98548 891,11608 0,9788

894,9749 0,98262 893,0449 0,98161

896,90371 0,97946 894,97371 0,98318

898,83253 0,97632 896,90253 0,98377

900,76134 0,97366 898,83134 0,98354

902,69016 0,97173 900,76016 0,98277

904,61898 0,97081 902,68898 0,98151

906,54779 0,9709 904,61779 0,98005

908,47661 0,9719 906,54661 0,97855

910,40542 0,97327 908,47542 0,97725

912,33424 0,97482 910,40424 0,97626

914,26306 0,97634 912,33306 0,97583

916,19187 0,97774 914,26187 0,97593

918,12069 0,97896 916,19069 0,97668

920,0495 0,97994 918,1195 0,97791

921,97832 0,98066 920,04832 0,97936

923,90714 0,98113 921,97714 0,98059

925,83595 0,98131 923,90595 0,98163

927,76477 0,9812 925,83477 0,98229

929,69358 0,98084 927,76358 0,98262

931,6224 0,98029 929,6924 0,98249

933,55122 0,97955 931,62122 0,98198

935,48003 0,97874 933,55003 0,98103

937,40885 0,97776 935,47885 0,97978

939,33766 0,97666 937,40766 0,97815

941,26648 0,97546 939,33648 0,97631

943,1953 0,97416 941,2653 0,97418

945,12411 0,97276 943,19411 0,97196

947,05293 0,97125 945,12293 0,96958

948,98174 0,96966 947,05174 0,96718

950,91056 0,96807 948,98056 0,96491

952,83938 0,96647 950,90938 0,96297

954,76819 0,96502 952,83819 0,96121

956,69701 0,96378 954,76701 0,95966

958,62582 0,9628 956,69582 0,9582

960,55464 0,96179 958,62464 0,95683

962,48346 0,96118 960,55346 0,95553

964,41227 0,9615 962,48227 0,95436

966,34109 0,96241 964,41109 0,95318

968,2699 0,9635 966,3399 0,952

970,19872 0,96437 968,26872 0,95072

972,12754 0,9647 970,19754 0,94937

974,05635 0,96441 972,12635 0,94777

975,98517 0,96355 974,05517 0,94584

977,91398 0,96224 975,98398 0,94318

979,8428 0,96047 977,9128 0,93993

981,77162 0,95803 979,84162 0,93611

983,70043 0,95472 981,77043 0,93188

985,62925 0,95099 983,69925 0,92735

987,55806 0,94693 985,62806 0,92277

989,48688 0,94204 987,55688 0,91839

991,4157 0,93632 989,4857 0,91431

993,34451 0,92977 991,41451 0,91051

995,27333 0,9228 993,34333 0,90716

997,20214 0,9159 995,27214 0,90428

999,13096 0,90964 997,20096 0,90207

1001,05978 0,9044 999,12978 0,90054

1002,98859 0,90041 1001,05859 0,89975

1004,91741 0,89754 1002,98741 0,89958

1006,84622 0,8957 1004,91622 0,90007

1008,77504 0,89474 1006,84504 0,90115

1010,70386 0,89465 1008,77386 0,90274

1012,63267 0,89541 1010,70267 0,90465

1014,56149 0,89702 1012,63149 0,90677

1016,4903 0,89943 1014,5603 0,90893

1018,41912 0,90272 1016,48912 0,91145

1020,34794 0,90681 1018,41794 0,91428

1022,27675 0,91173 1020,34675 0,9175

1024,20557 0,91774 1022,27557 0,92109

1026,13438 0,92448 1024,20438 0,92515

1028,0632 0,93101 1026,1332 0,92974

1029,99202 0,93708 1028,06202 0,93472

1031,92083 0,94256 1029,99083 0,93983

1033,84965 0,94757 1031,91965 0,94506

1035,77846 0,95219 1033,84846 0,95031

1037,70728 0,95647 1035,77728 0,9556

1039,6361 0,96023 1037,7061 0,96081

1041,56491 0,96338 1039,63491 0,96583

1043,49373 0,96586 1041,56373 0,97053

1045,42254 0,96781 1043,49254 0,97492

1047,35136 0,96949 1045,42136 0,97896

1049,28018 0,97052 1047,35018 0,98263

1051,20899 0,97057 1049,27899 0,98587

1053,13781 0,9702 1051,20781 0,98872

1055,06662 0,96976 1053,13662 0,99122

1056,99544 0,96976 1055,06544 0,99344

1058,92426 0,97044 1056,99426 0,99533

1060,85307 0,97189 1058,92307 0,99697

1062,78189 0,97383 1060,85189 0,99832

1064,7107 0,97617 1062,7807 0,99937

1066,63952 0,97865 1064,70952 1

1068,56834 0,98122 1066,63834 0,99996

1070,49715 0,98372 1068,56715 0,99915

1072,42597 0,98614 1070,49597 0,99766

1074,35478 0,98832 1072,42478 0,99544

1076,2836 0,99017 1074,3536 0,99251

1078,21242 0,99148 1076,28242 0,98878

1080,14123 0,99215 1078,21123 0,98428

1082,07005 0,99196 1080,14005 0,97911

1083,99886 0,99079 1082,06886 0,9734

1085,92768 0,98854 1083,99768 0,96728

1087,8565 0,98532 1085,9265 0,9609

1089,78531 0,98104 1087,85531 0,95439

1091,71413 0,97599 1089,78413 0,94797

1093,64294 0,97037 1091,71294 0,94178

1095,57176 0,96449 1093,64176 0,93596

1097,50058 0,95837 1095,57058 0,93057

1099,42939 0,95204 1097,49939 0,9257

1101,35821 0,94537 1099,42821 0,92138

1103,28702 0,93843 1101,35702 0,91766

1105,21584 0,93125 1103,28584 0,91457

1107,14466 0,92397 1105,21466 0,91212

1109,07347 0,91663 1107,14347 0,91033

1111,00229 0,90952 1109,07229 0,90926

1112,9311 0,90283 1111,0011 0,9089

1114,85992 0,89708 1112,92992 0,90929

1116,78874 0,89258 1114,85874 0,91053

1118,71755 0,88962 1116,78755 0,91268

1120,64637 0,88821 1118,71637 0,91585

1122,57518 0,88844 1120,64518 0,92006

1124,504 0,89025 1122,574 0,92518

1126,43282 0,89371 1124,50282 0,93106

1128,36163 0,89869 1126,43163 0,93755

1130,29045 0,90505 1128,36045 0,94452

1132,21926 0,91244 1130,28926 0,95181

1134,14808 0,92067 1132,21808 0,95914

1136,0769 0,92935 1134,1469 0,96615

1138,00571 0,9383 1136,07571 0,97246

1139,93453 0,947 1138,00453 0,97768

1141,86334 0,95513 1139,93334 0,98144

1143,79216 0,96232 1141,86216 0,98342

1145,72098 0,96849 1143,79098 0,98337

1147,64979 0,97356 1145,71979 0,9811

1149,57861 0,97754 1147,64861 0,97654

1151,50742 0,9803 1149,57742 0,96971

1153,43624 0,98187 1151,50624 0,96073

1155,36506 0,98203 1153,43506 0,94987

1157,29387 0,98057 1155,36387 0,9374

1159,22269 0,97722 1157,29269 0,92371

1161,1515 0,97205 1159,2215 0,90903

1163,08032 0,96504 1161,15032 0,89371

1165,00914 0,95671 1163,07914 0,87814

1166,93795 0,94737 1165,00795 0,86274

1168,86677 0,93694 1166,93677 0,84789

1170,79558 0,92497 1168,86558 0,83393

1172,7244 0,91114 1170,7944 0,82099

1174,65322 0,89515 1172,72322 0,80924

1176,58203 0,8771 1174,65203 0,79877

1178,51085 0,85702 1176,58085 0,78966

1180,43966 0,83541 1178,50966 0,78195

1182,36848 0,813 1180,43848 0,77567

1184,2973 0,79117 1182,3673 0,77075

1186,22611 0,7712 1184,29611 0,76718

1188,15493 0,75448 1186,22493 0,76486

1190,08374 0,74169 1188,15374 0,76374

1192,01256 0,73291 1190,08256 0,76371

1193,94138 0,72777 1192,01138 0,76472

1195,87019 0,72611 1193,94019 0,76664

1197,79901 0,72752 1195,86901 0,7694

1199,72782 0,73174 1197,79782 0,7728

1201,65664 0,73817 1199,72664 0,7769

1203,58546 0,74666 1201,65546 0,78162

1205,51427 0,75689 1203,58427 0,78697

1207,44309 0,7685 1205,51309 0,79269

1209,3719 0,78066 1207,4419 0,79839

1211,30072 0,7927 1209,37072 0,8035

1213,22954 0,80395 1211,29954 0,80757

1215,15835 0,81406 1213,22835 0,81017

1217,08717 0,82261 1215,15717 0,81103

1219,01598 0,82965 1217,08598 0,80989

1220,9448 0,83521 1219,0148 0,80665

1222,87362 0,83967 1220,94362 0,80119

1224,80243 0,84309 1222,87243 0,79352

1226,73125 0,84543 1224,80125 0,7837

1228,66006 0,84548 1226,73006 0,7719

1230,58888 0,84156 1228,65888 0,7583

1232,5177 0,83218 1230,5877 0,74332

1234,44651 0,8168 1232,51651 0,72746

1236,37533 0,7957 1234,44533 0,7113

1238,30414 0,77004 1236,37414 0,69541

1240,23296 0,74123 1238,30296 0,68047

1242,16178 0,71129 1240,23178 0,66695

1244,09059 0,68229 1242,16059 0,65522

1246,01941 0,65647 1244,08941 0,64532

1247,94822 0,63529 1246,01822 0,63722

1249,87704 0,61958 1247,94704 0,63085

1251,80586 0,60904 1249,87586 0,62621

1253,73467 0,60334 1251,80467 0,62329

1255,66349 0,60214 1253,73349 0,62204

1257,5923 0,60555 1255,6623 0,62231

1259,52112 0,61335 1257,59112 0,62411

1261,44994 0,62533 1259,51994 0,6272

1263,37875 0,64084 1261,44875 0,63147

1265,30757 0,65923 1263,37757 0,6369

1267,23638 0,67951 1265,30638 0,64345

1269,1652 0,70102 1267,2352 0,65104

1271,09402 0,72302 1269,16402 0,65978

1273,02283 0,74545 1271,09283 0,66974

1274,95165 0,76812 1273,02165 0,68096

1276,88046 0,79097 1274,95046 0,69346

1278,80928 0,81356 1276,87928 0,70721

1280,7381 0,8358 1278,8081 0,72196

1282,66691 0,85723 1280,73691 0,73734

1284,59573 0,87755 1282,66573 0,75318

1286,52454 0,89588 1284,59454 0,7695

1288,45336 0,9113 1286,52336 0,78592

1290,38218 0,92259 1288,45218 0,80222

1292,31099 0,9295 1290,38099 0,81808

1294,23981 0,93221 1292,30981 0,83331

1296,16862 0,93174 1294,23862 0,84773

1298,09744 0,92888 1296,16744 0,86114

1300,02626 0,9246 1298,09626 0,87335

1301,95507 0,91962 1300,02507 0,88436

1303,88389 0,91492 1301,95389 0,89418

1305,8127 0,9112 1303,8827 0,90267

1307,74152 0,90903 1305,81152 0,91004

1309,67034 0,90821 1307,74034 0,91686

1311,59915 0,90842 1309,66915 0,92308

1313,52797 0,90897 1311,59797 0,9288

1315,45678 0,90963 1313,52678 0,93402

1317,3856 0,90993 1315,4556 0,93857

1319,31442 0,90974 1317,38442 0,94223

1321,24323 0,90833 1319,31323 0,94511

1323,17205 0,90545 1321,24205 0,94707

1325,10086 0,90083 1323,17086 0,94817

1327,02968 0,89451 1325,09968 0,94786

1328,9585 0,88653 1327,0285 0,9461

1330,88731 0,87734 1328,95731 0,94239

1332,81613 0,86674 1330,88613 0,93694

1334,74494 0,85486 1332,81494 0,92961

1336,67376 0,84124 1334,74376 0,92025

1338,60258 0,82543 1336,67258 0,9084

1340,53139 0,80686 1338,60139 0,89446

1342,46021 0,78546 1340,53021 0,87845

1344,38902 0,76096 1342,45902 0,8606

1346,31784 0,73353 1344,38784 0,84142

1348,24666 0,70354 1346,31666 0,8208

1350,17547 0,67177 1348,24547 0,79858

1352,10429 0,63894 1350,17429 0,775

1354,0331 0,60611 1352,1031 0,75005

1355,96192 0,57378 1354,03192 0,72374

1357,89074 0,54261 1355,96074 0,69633

1359,81955 0,51276 1357,88955 0,66793

1361,74837 0,48465 1359,81837 0,63847

1363,67718 0,45824 1361,74718 0,60814

1365,606 0,43363 1363,676 0,5771

1367,53482 0,41056 1365,60482 0,54527

1369,46363 0,38869 1367,53363 0,51275

1371,39245 0,36774 1369,46245 0,48007

1373,32126 0,3476 1371,39126 0,44691

1375,25008 0,32788 1373,32008 0,41345

1377,1789 0,30845 1375,2489 0,3799

1379,10771 0,28909 1377,17771 0,34673

1381,03653 0,27004 1379,10653 0,31356

1382,96534 0,2512 1381,03534 0,28104

1384,89416 0,2331 1382,96416 0,2493

1386,82298 0,21582 1384,89298 0,21811

1388,75179 0,19947 1386,82179 0,18752

1390,68061 0,18389 1388,75061 0,15775

1392,60942 0,16914 1390,67942 0,12907

1394,53824 0,15502 1392,60824 0,10223

1396,46706 0,14116 1394,53706 0,07795

1398,39587 0,12718 1396,46587 0,05678

1400,32469 0,1129 1398,39469 0,03851

1402,2535 0,09832 1400,3235 0,02463

1404,18232 0,08397 1402,25232 0,01613

1406,11114 0,07011 1404,18114 0,01336

1408,03995 0,05784 1406,10995 0,01613

1409,96877 0,04838 1408,03877 0,02505

1411,89758 0,04282 1409,96758 0,03976

1413,8264 0,04246 1411,8964 0,06024

1415,75522 0,04788 1413,82522 0,08555

1417,68403 0,05946 1415,75403 0,11607

1419,61285 0,07751 1417,68285 0,15141

1421,54166 0,10233 1419,61166 0,19163

1423,47048 0,13353 1421,54048 0,23547

1425,3993 0,17042 1423,4693 0,28146

1427,32811 0,21244 1425,39811 0,32923

1429,25693 0,25884 1427,32693 0,37812

1431,18574 0,30864 1429,25574 0,42742

1433,11456 0,36115 1431,18456 0,47674

1435,04338 0,41543 1433,11338 0,52582

1436,97219 0,4706 1435,04219 0,57454

1438,90101 0,52549 1436,97101 0,62205

1440,82982 0,57934 1438,89982 0,66912

1442,75864 0,6319 1440,82864 0,71514

1444,68746 0,68241 1442,75746 0,75885

1446,61627 0,73019 1444,68627 0,79943

1448,54509 0,7743 1446,61509 0,83651

1450,4739 0,81406 1448,5439 0,8674

1452,40272 0,84893 1450,47272 0,89405

1454,33154 0,87832 1452,40154 0,91631

1456,26035 0,90146 1454,33035 0,93462

1458,18917 0,91788 1456,25917 0,94794

1460,11798 0,92741 1458,18798 0,95592

1462,0468 0,93013 1460,1168 0,95784

1463,97562 0,92604 1462,04562 0,95407

1465,90443 0,91572 1463,97443 0,94454

1467,83325 0,89927 1465,90325 0,93109

1469,76206 0,87829 1467,83206 0,91416

1471,69088 0,85357 1469,76088 0,89506

1473,6197 0,82664 1471,6897 0,87419

1475,54851 0,7982 1473,61851 0,85203

1477,47733 0,76921 1475,54733 0,82751

1479,40614 0,73971 1477,47614 0,8027

1481,33496 0,71071 1479,40496 0,77656

1483,26378 0,68195 1481,33378 0,74706

1485,19259 0,65309 1483,26259 0,71409

1487,12141 0,62324 1485,19141 0,67832

1489,05022 0,59174 1487,12022 0,6392

1490,97904 0,55782 1489,04904 0,59942

1492,90786 0,52075 1490,97786 0,55697

1494,83667 0,48025 1492,90667 0,5122

1496,76549 0,43674 1494,83549 0,46489

1498,6943 0,38981 1496,7643 0,41478

1500,62312 0,34046 1498,69312 0,36268

1502,55194 0,29001 1500,62194 0,30634

1504,48075 0,24065 1502,55075 0,253

1506,40957 0,19365 1504,47957 0,20497

1508,33838 0,15065 1506,40838 0,16136

1510,2672 0,11246 1508,3372 0,12306

1512,19602 0,08015 1510,26602 0,08998

1514,12483 0,05376 1512,19483 0,06193

1516,05365 0,03392 1514,12365 0,03912

1517,98246 0,01827 1516,05246 0,02259

1519,91128 0,00718 1517,98128 0,01027

1521,8401 8,75142E-4 1519,9101 0,0022

1523,76891 0 1521,83891 0

1525,69773 0,00563 1523,76773 0,00435

1527,62654 0,01859 1525,69654 0,01473

1529,55536 0,03984 1527,62536 0,03315

1531,48418 0,07018 1529,55418 0,0569

1533,41299 0,10994 1531,48299 0,08501

1535,34181 0,15876 1533,41181 0,11565

1537,27062 0,21569 1535,34062 0,14786

1539,19944 0,27969 1537,26944 0,18176

1541,12826 0,34553 1539,19826 0,21667

1543,05707 0,4123 1541,12707 0,25754

1544,98589 0,47673 1543,05589 0,30035

1546,9147 0,53575 1544,9847 0,34232

1548,84352 0,58621 1546,91352 0,38293

1550,77234 0,62573 1548,84234 0,42042

1552,70115 0,65296 1550,77115 0,45356

1554,62997 0,66757 1552,69997 0,48226

1556,55878 0,67102 1554,62878 0,50693

1558,4876 0,66509 1556,5576 0,52647

1560,41642 0,65187 1558,48642 0,54099

1562,34523 0,63384 1560,41523 0,55256

1564,27405 0,61266 1562,34405 0,56163

1566,20286 0,5898 1564,27286 0,56785

1568,13168 0,56548 1566,20168 0,57172

1570,0605 0,53967 1568,1305 0,57065

1571,98931 0,51176 1570,05931 0,56566

1573,91813 0,48173 1571,98813 0,55814

1575,84694 0,44903 1573,91694 0,54899

1577,77576 0,41464 1575,84576 0,53969

1579,70458 0,37914 1577,77458 0,53012

1581,63339 0,34642 1579,70339 0,5267

1583,56221 0,31824 1581,63221 0,52519

1585,49102 0,29663 1583,56102 0,52368

1587,41984 0,28333 1585,48984 0,52353

1589,34866 0,27893 1587,41866 0,5242

1591,27747 0,28409 1589,34747 0,52597

1593,20629 0,29871 1591,27629 0,53126

1595,1351 0,3219 1593,2051 0,53958

1597,06392 0,35196 1595,13392 0,54806

1598,99274 0,38731 1597,06274 0,56176

1600,92155 0,42562 1598,99155 0,58116

1602,85037 0,46442 1600,92037 0,60356

1604,77918 0,50438 1602,84918 0,62708

1606,708 0,54278 1604,778 0,65069

1608,63682 0,57851 1606,70682 0,67366

1610,56563 0,61238 1608,63563 0,69549

1612,49445 0,64206 1610,56445 0,71582

1614,42326 0,66897 1612,49326 0,73434

1616,35208 0,69466 1614,42208 0,75209

1618,2809 0,72022 1616,3509 0,76907

1620,20971 0,74607 1618,27971 0,78722

1622,13853 0,77219 1620,20853 0,79968

1624,06734 0,79754 1622,13734 0,80887

1625,99616 0,82179 1624,06616 0,81555

1627,92498 0,84379 1625,99498 0,81843

1629,85379 0,86234 1627,92379 0,82122

1631,78261 0,87752 1629,85261 0,82536

1633,71142 0,88949 1631,78142 0,82908

1635,64024 0,89616 1633,71024 0,83332

1637,56906 0,89991 1635,63906 0,84123

1639,49787 0,90217 1637,56787 0,84948

1641,42669 0,90454 1639,49669 0,85685

1643,3555 0,90576 1641,4255 0,86277

1645,28432 0,90617 1643,35432 0,86709

1647,21314 0,90681 1645,28314 0,87071

1649,14195 0,90795 1647,21195 0,87241

1651,07077 0,90959 1649,14077 0,87308

1652,99958 0,91179 1651,06958 0,87294

1654,9284 0,91449 1652,9984 0,87237

1656,85722 0,91724 1654,92722 0,87115

1658,78603 0,92008 1656,85603 0,87008

1660,71485 0,92282 1658,78485 0,86854

1662,64366 0,92588 1660,71366 0,86636

1664,57248 0,92897 1662,64248 0,86464

1666,5013 0,93053 1664,5713 0,86316

1668,43011 0,93138 1666,50011 0,86479

1670,35893 0,93224 1668,42893 0,86822

1672,28774 0,93309 1670,35774 0,87169

1674,21656 0,93263 1672,28656 0,87506

1676,14538 0,93301 1674,21538 0,88092

1678,07419 0,9339 1676,14419 0,88532

1680,00301 0,93539 1678,07301 0,88917

1681,93182 0,93772 1680,00182 0,89235

1683,86064 0,94043 1681,93064 0,89507

1685,78946 0,94388 1683,85946 0,89774

1687,71827 0,94757 1685,78827 0,89914

1689,64709 0,95101 1687,71709 0,90036

1691,5759 0,95417 1689,6459 0,90099

1693,50472 0,95677 1691,57472 0,90163

1695,43354 0,95811 1693,50354 0,90216

1697,36235 0,95765 1695,43235 0,90323

1699,29117 0,95773 1697,36117 0,90405

1701,21998 0,95738 1699,28998 0,90492

1703,1488 0,95677 1701,2188 0,90704

1705,07762 0,95524 1703,14762 0,90976

1707,00643 0,95414 1705,07643 0,91562

1708,93525 0,9539 1707,00525 0,92232

1710,86406 0,95416 1708,93406 0,92836

1712,79288 0,95485 1710,86288 0,9349

1714,7217 0,95621 1712,7917 0,94212

1716,65051 0,95735 1714,72051 0,94613

1718,57933 0,95872 1716,64933 0,95107

1720,50814 0,96008 1718,57814 0,9558

1722,43696 0,96116 1720,50696 0,9604

1724,36578 0,96221 1722,43578 0,96481

1726,29459 0,96272 1724,36459 0,9681

1728,22341 0,96265 1726,29341 0,97122

1730,15222 0,96332 1728,22222 0,9738

1732,08104 0,96389 1730,15104 0,97582

1734,00986 0,96408 1732,07986 0,97689

1735,93867 0,96396 1734,00867 0,97746

1737,86749 0,9635 1735,93749 0,9781

1739,7963 0,96248 1737,8663 0,979

1741,72512 0,96163 1739,79512 0,98095

1743,65394 0,96069 1741,72394 0,98281

1745,58275 0,95981 1743,65275 0,98538

1747,51157 0,95941 1745,58157 0,98602

1749,44038 0,95903 1747,51038 0,98586

1751,3692 0,9586 1749,4392 0,98534

1753,29802 0,95795 1751,36802 0,98478

1755,22683 0,95641 1753,29683 0,98477

1757,15565 0,95481 1755,22565 0,98698

1759,08446 0,95308 1757,15446 0,98908

1761,01328 0,95118 1759,08328 0,99104

1762,9421 0,94952 1761,0121 0,99282

1764,87091 0,94832 1762,94091 0,99415

1766,79973 0,94758 1764,86973 0,99538

1768,72854 0,94714 1766,79854 0,99576

1770,65736 0,94697 1768,72736 0,99608

1772,58618 0,94681 1770,65618 0,99617

1774,51499 0,94667 1772,58499 0,99639

1776,44381 0,94651 1774,51381 0,99726

1778,37262 0,94613 1776,44262 0,99795

1780,30144 0,9463 1778,37144 0,99835

1782,23026 0,94655 1780,30026 0,99774

1784,15907 0,94651 1782,22907 0,99712

1786,08789 0,94655 1784,15789 0,99628

1788,0167 0,94634 1786,0867 0,99494

1789,94552 0,94585 1788,01552 0,99391

1791,87434 0,94495 1789,94434 0,99347

1793,80315 0,94366 1791,87315 0,99377

1795,73197 0,94247 1793,80197 0,9953

1797,66078 0,94167 1795,73078 0,99595

1799,5896 0,94112 1797,6596 0,99645

1801,51842 0,94083 1799,58842 0,99675

1803,44723 0,94076 1801,51723 0,99684

1805,37605 0,94076 1803,44605 0,99677

1807,30486 0,94066 1805,37486 0,99648

1809,23368 0,94066 1807,30368 0,99632

1811,1625 0,94044 1809,2325 0,9956

1813,09131 0,93987 1811,16131 0,99476

1815,02013 0,93934 1813,09013 0,99446

1816,94894 0,93886 1815,01894 0,9943

1818,87776 0,93872 1816,94776 0,99427

1820,80658 0,93876 1818,87658 0,994

1822,73539 0,93895 1820,80539 0,99384

1824,66421 0,93933 1822,73421 0,99339

1826,59302 0,93974 1824,66302 0,99272

1828,52184 0,93993 1826,59184 0,99232

1830,45066 0,93979 1828,52066 0,99206

1832,37947 0,9394 1830,44947 0,99188

1834,30829 0,93884 1832,37829 0,99163

1836,2371 0,9382 1834,3071 0,99074

1838,16592 0,93762 1836,23592 0,9894

1840,09474 0,93699 1838,16474 0,98771

1842,02355 0,9363 1840,09355 0,9859

1843,95237 0,93561 1842,02237 0,98371

1845,88118 0,93484 1843,95118 0,98147

1847,81 0,93417 1845,88 0,97931

1849,73882 0,93337 1847,80882 0,97725

1851,66763 0,9324 1849,73763 0,97589

1853,59645 0,93145 1851,66645 0,97537

1855,52526 0,93055 1853,59526 0,97473

1857,45408 0,92975 1855,52408 0,97415

1859,3829 0,92897 1857,4529 0,97354

1861,31171 0,92806 1859,38171 0,9729

1863,24053 0,92691 1861,31053 0,97211

1865,16934 0,92535 1863,23934 0,97125

1867,09816 0,92366 1865,16816 0,9705

1869,02698 0,92173 1867,09698 0,96994

1870,95579 0,91975 1869,02579 0,96929

1872,88461 0,9178 1870,95461 0,9685

1874,81342 0,91618 1872,88342 0,96736

1876,74224 0,91488 1874,81224 0,96588

1878,67106 0,91358 1876,74106 0,96419

1880,59987 0,91249 1878,66987 0,96234

1882,52869 0,91156 1880,59869 0,96036

1884,4575 0,91067 1882,5275 0,95833

1886,38632 0,90972 1884,45632 0,95619

1888,31514 0,90868 1886,38514 0,95404

1890,24395 0,90768 1888,31395 0,95227

1892,17277 0,90687 1890,24277 0,95085

1894,10158 0,90653 1892,17158 0,94924

1896,0304 0,90659 1894,1004 0,94758

1897,95922 0,90712 1896,02922 0,94578

1899,88803 0,90793 1897,95803 0,94395

1901,81685 0,90868 1899,88685 0,94223

1903,74566 0,90964 1901,81566 0,94077

1905,67448 0,91059 1903,74448 0,93938

1907,6033 0,91148 1905,6733 0,93804

1909,53211 0,91224 1907,60211 0,93646

1911,46093 0,91289 1909,53093 0,93478

1913,38974 0,9135 1911,45974 0,93318

1915,31856 0,91424 1913,38856 0,93156

1917,24738 0,91513 1915,31738 0,92999

1919,17619 0,91598 1917,24619 0,92851

1921,10501 0,91683 1919,17501 0,92705

1923,03382 0,91752 1921,10382 0,9257

1924,96264 0,91788 1923,03264 0,92452

1926,89146 0,91808 1924,96146 0,924

1928,82027 0,91812 1926,89027 0,92403

1930,74909 0,91814 1928,81909 0,92448

1932,6779 0,91843 1930,7479 0,92505

1934,60672 0,91901 1932,67672 0,92585

1936,53554 0,91997 1934,60554 0,92679

1938,46435 0,92107 1936,53435 0,92811

1940,39317 0,92251 1938,46317 0,92977

1942,32198 0,9244 1940,39198 0,93166

1944,2508 0,92652 1942,3208 0,93366

1946,17962 0,92898 1944,24962 0,93592

1948,10843 0,93172 1946,17843 0,93795

1950,03725 0,93464 1948,10725 0,93994

1951,96606 0,93748 1950,03606 0,94168

1953,89488 0,94037 1951,96488 0,94299

1955,8237 0,94326 1953,8937 0,94392

1957,75251 0,94573 1955,82251 0,94433

1959,68133 0,94792 1957,75133 0,94405

1961,61014 0,94945 1959,68014 0,94323

1963,53896 0,95047 1961,60896 0,94206

1965,46778 0,95121 1963,53778 0,94096

1967,39659 0,95157 1965,46659 0,9397

1969,32541 0,95202 1967,39541 0,93842

1971,25422 0,95256 1969,32422 0,93709

1973,18304 0,95311 1971,25304 0,93576

1975,11186 0,95358 1973,18186 0,93446

1977,04067 0,95404 1975,11067 0,93354

1978,96949 0,95428 1977,03949 0,9328

1980,8983 0,95419 1978,9683 0,93223

1982,82712 0,95393 1980,89712 0,93181

1984,75594 0,95322 1982,82594 0,93162

1986,68475 0,95216 1984,75475 0,93137

1988,61357 0,95104 1986,68357 0,93137

1990,54238 0,95013 1988,61238 0,93152

1992,4712 0,94977 1990,5412 0,9318

1994,40002 0,94989 1992,47002 0,93209

1996,32883 0,95023 1994,39883 0,93244

1998,25765 0,95105 1996,32765 0,93283

2000,18646 0,95209 1998,25646 0,93347

2002,11528 0,95327 2000,18528 0,9345

2004,0441 0,95448 2002,1141 0,93594

2005,97291 0,95569 2004,04291 0,93736

2007,90173 0,95675 2005,97173 0,93889

2009,83054 0,95755 2007,90054 0,94042

2011,75936 0,95805 2009,82936 0,94203

2013,68818 0,95833 2011,75818 0,94354

2015,61699 0,95846 2013,68699 0,94507

2017,54581 0,95826 2015,61581 0,94629

2019,47462 0,95765 2017,54462 0,94727

2021,40344 0,95701 2019,47344 0,94793

2023,33226 0,95614 2021,40226 0,94826

2025,26107 0,95509 2023,33107 0,94815

2027,18989 0,95393 2025,25989 0,94771

2029,1187 0,95269 2027,1887 0,94679

2031,04752 0,95143 2029,11752 0,94543

2032,97634 0,94996 2031,04634 0,94363

2034,90515 0,94819 2032,97515 0,94155

2036,83397 0,94615 2034,90397 0,93923

2038,76278 0,94398 2036,83278 0,93673

2040,6916 0,94166 2038,7616 0,93413

2042,62042 0,93938 2040,69042 0,93142

2044,54923 0,93723 2042,61923 0,92849

2046,47805 0,93508 2044,54805 0,92555

2048,40686 0,93313 2046,47686 0,92261

2050,33568 0,93153 2048,40568 0,91976

2052,2645 0,93028 2050,3345 0,91695

2054,19331 0,92925 2052,26331 0,91431

2056,12213 0,92832 2054,19213 0,91166

2058,05094 0,92748 2056,12094 0,90919

2059,97976 0,92677 2058,04976 0,90684

2061,90858 0,92611 2059,97858 0,90469

2063,83739 0,92559 2061,90739 0,90272

2065,76621 0,9253 2063,83621 0,90101

2067,69502 0,92519 2065,76502 0,89947

2069,62384 0,92521 2067,69384 0,89821

2071,55266 0,92556 2069,62266 0,89707

2073,48147 0,92611 2071,55147 0,89618

2075,41029 0,92683 2073,48029 0,89542

2077,3391 0,92757 2075,4091 0,89479

2079,26792 0,92833 2077,33792 0,89417

2081,19674 0,92908 2079,26674 0,89352

2083,12555 0,92981 2081,19555 0,89281

2085,05437 0,93035 2083,12437 0,89221

2086,98318 0,93078 2085,05318 0,89163

2088,912 0,93125 2086,982 0,89114

2090,84082 0,93167 2088,91082 0,89062

2092,76963 0,93211 2090,83963 0,89017

2094,69845 0,93261 2092,76845 0,88973

2096,62726 0,93321 2094,69726 0,88944

2098,55608 0,93397 2096,62608 0,8892

2100,4849 0,93491 2098,5549 0,88915

2102,41371 0,93609 2100,48371 0,88921

2104,34253 0,93745 2102,41253 0,88932

2106,27134 0,93895 2104,34134 0,88941

2108,20016 0,94047 2106,27016 0,8897

2110,12898 0,94214 2108,19898 0,8901

2112,05779 0,94383 2110,12779 0,89076

2113,98661 0,94537 2112,05661 0,89162

2115,91542 0,94673 2113,98542 0,89276

2117,84424 0,94777 2115,91424 0,89419

2119,77306 0,94872 2117,84306 0,89598

2121,70187 0,94966 2119,77187 0,89813

2123,63069 0,95062 2121,70069 0,90068

2125,5595 0,95158 2123,6295 0,90349

2127,48832 0,95248 2125,55832 0,90655

2129,41714 0,95331 2127,48714 0,90975

2131,34595 0,95398 2129,41595 0,91318

2133,27477 0,95449 2131,34477 0,91673

2135,20358 0,95458 2133,27358 0,92039

2137,1324 0,95419 2135,2024 0,92403

2139,06122 0,95326 2137,13122 0,92767

2140,99003 0,95184 2139,06003 0,93108

2142,91885 0,95019 2140,98885 0,9342

2144,84766 0,94837 2142,91766 0,93701

2146,77648 0,94636 2144,84648 0,93963

2148,7053 0,9443 2146,7753 0,94186

2150,63411 0,94225 2148,70411 0,94379

2152,56293 0,94027 2150,63293 0,94534

2154,49174 0,93836 2152,56174 0,94657

2156,42056 0,9366 2154,49056 0,94739

2158,34938 0,93498 2156,41938 0,94786

2160,27819 0,93357 2158,34819 0,94792

2162,20701 0,93235 2160,27701 0,9477

2164,13582 0,93134 2162,20582 0,94706

2166,06464 0,93052 2164,13464 0,94612

2167,99346 0,92991 2166,06346 0,94482

2169,92227 0,92939 2167,99227 0,94333

2171,85109 0,92903 2169,92109 0,94163

2173,7799 0,92865 2171,8499 0,93981

2175,70872 0,92825 2173,77872 0,93784

2177,63754 0,9279 2175,70754 0,93579

2179,56635 0,92766 2177,63635 0,93355

2181,49517 0,92775 2179,56517 0,93123

2183,42398 0,92812 2181,49398 0,92897

2185,3528 0,9286 2183,4228 0,92697

2187,28162 0,92912 2185,35162 0,9251

2189,21043 0,92969 2187,28043 0,92346

2191,13925 0,9303 2189,20925 0,92207

2193,06806 0,93081 2191,13806 0,92094

2194,99688 0,93119 2193,06688 0,91997

2196,9257 0,93133 2194,9957 0,91923

2198,85451 0,93138 2196,92451 0,91862

2200,78333 0,93146 2198,85333 0,91818

2202,71214 0,93162 2200,78214 0,91773

2204,64096 0,93201 2202,71096 0,91739

2206,56978 0,93244 2204,63978 0,9171

2208,49859 0,93283 2206,56859 0,91695

2210,42741 0,93325 2208,49741 0,91675

2212,35622 0,93364 2210,42622 0,9165

2214,28504 0,93398 2212,35504 0,91616

2216,21386 0,93422 2214,28386 0,91576

2218,14267 0,93443 2216,21267 0,91522

2220,07149 0,93459 2218,14149 0,9147

2222,0003 0,93479 2220,0703 0,9143

2223,92912 0,935 2221,99912 0,91398

2225,85794 0,93518 2223,92794 0,91356

2227,78675 0,93532 2225,85675 0,91321

2229,71557 0,93542 2227,78557 0,91285

2231,64438 0,93544 2229,71438 0,91246

2233,5732 0,93546 2231,6432 0,91196

2235,50202 0,93546 2233,57202 0,9114

2237,43083 0,93547 2235,50083 0,91078

2239,35965 0,93552 2237,42965 0,9102

2241,28846 0,93558 2239,35846 0,90954

2243,21728 0,93565 2241,28728 0,9089

2245,1461 0,93578 2243,2161 0,90824

2247,07491 0,9359 2245,14491 0,90756

2249,00373 0,93605 2247,07373 0,90681

2250,93254 0,93627 2249,00254 0,90603

2252,86136 0,93654 2250,93136 0,90517

2254,79018 0,93682 2252,86018 0,90424

2256,71899 0,93717 2254,78899 0,90323

2258,64781 0,93761 2256,71781 0,90219

2260,57662 0,93813 2258,64662 0,90109

2262,50544 0,93872 2260,57544 0,89993

2264,43426 0,93933 2262,50426 0,89869

2266,36307 0,94005 2264,43307 0,89743

2268,29189 0,94083 2266,36189 0,8961

2270,2207 0,94167 2268,2907 0,8948

2272,14952 0,94253 2270,21952 0,89353

2274,07834 0,94342 2272,14834 0,89233

2276,00715 0,9443 2274,07715 0,89119

2277,93597 0,94518 2276,00597 0,89013

2279,86478 0,94604 2277,93478 0,88901

2281,7936 0,94689 2279,8636 0,88793

2283,72242 0,94774 2281,79242 0,88684

2285,65123 0,94852 2283,72123 0,88574

2287,58005 0,94915 2285,65005 0,8846

2289,50886 0,94961 2287,57886 0,88348

2291,43768 0,94985 2289,50768 0,88235

2293,3665 0,94981 2291,4365 0,88126

2295,29531 0,94941 2293,36531 0,88023

2297,22413 0,94868 2295,29413 0,87925

2299,15294 0,9476 2297,22294 0,87823

2301,08176 0,9462 2299,15176 0,87731

2303,01058 0,94452 2301,08058 0,87641

2304,93939 0,94266 2303,00939 0,87557

2306,86821 0,94056 2304,93821 0,87481

2308,79702 0,93808 2306,86702 0,87427

2310,72584 0,93526 2308,79584 0,87379

2312,65466 0,9322 2310,72466 0,87344

2314,58347 0,92913 2312,65347 0,87323

2316,51229 0,92619 2314,58229 0,8731

2318,4411 0,92358 2316,5111 0,87299

2320,36992 0,92139 2318,43992 0,87295

2322,29874 0,91966 2320,36874 0,87292

2324,22755 0,91836 2322,29755 0,87299

2326,15637 0,91737 2324,22637 0,87318

2328,08518 0,91663 2326,15518 0,8735

2330,014 0,91573 2328,084 0,8739

2331,94282 0,91442 2330,01282 0,87439

2333,87163 0,9126 2331,94163 0,87498

2335,80045 0,91023 2333,87045 0,87565

2337,72926 0,90749 2335,79926 0,87635

2339,65808 0,9045 2337,72808 0,87717

2341,5869 0,90142 2339,6569 0,87807

2343,51571 0,89839 2341,58571 0,87909

2345,44453 0,89558 2343,51453 0,88026

2347,37334 0,89304 2345,44334 0,88162

2349,30216 0,89091 2347,37216 0,88303

2351,23098 0,88938 2349,30098 0,88455

2353,15979 0,88846 2351,22979 0,88612

2355,08861 0,88818 2353,15861 0,88765

2357,01742 0,8886 2355,08742 0,88915

2358,94624 0,88975 2357,01624 0,89073

2360,87506 0,89165 2358,94506 0,89231

2362,80387 0,89434 2360,87387 0,89401

2364,73269 0,89767 2362,80269 0,89575

2366,6615 0,90158 2364,7315 0,89753

2368,59032 0,90591 2366,66032 0,89928

2370,51914 0,91063 2368,58914 0,901

2372,44795 0,91586 2370,51795 0,90274

2374,37677 0,92164 2372,44677 0,90442

2376,30558 0,92801 2374,37558 0,90605

2378,2344 0,9348 2376,3044 0,90775

2380,16322 0,94189 2378,23322 0,9094

2382,09203 0,94902 2380,16203 0,91106

2384,02085 0,95587 2382,09085 0,91276

2385,94966 0,96219 2384,01966 0,91444

2387,87848 0,96768 2385,94848 0,91596

2389,8073 0,97212 2387,8773 0,91739

2391,73611 0,97528 2389,80611 0,9187

2393,66493 0,97724 2391,73493 0,91987

2395,59374 0,9782 2393,66374 0,92095

2397,52256 0,97829 2395,59256 0,922

2399,45138 0,97778 2397,52138 0,92298

2401,38019 0,97675 2399,45019 0,92394

2403,30901 0,97539 2401,37901 0,92477

2405,23782 0,97379 2403,30782 0,92547

2407,16664 0,97211 2405,23664 0,926

2409,09546 0,97048 2407,16546 0,9264

2411,02427 0,969 2409,09427 0,92676

2412,95309 0,96769 2411,02309 0,92696

2414,8819 0,96649 2412,9519 0,92707

2416,81072 0,96543 2414,88072 0,92717

2418,73954 0,96453 2416,80954 0,92717

2420,66835 0,96376 2418,73835 0,92721

2422,59717 0,9631 2420,66717 0,92721

2424,52598 0,96255 2422,59598 0,92715

2426,4548 0,962 2424,5248 0,92708

2428,38362 0,96148 2426,45362 0,92703

2430,31243 0,96103 2428,38243 0,92698

2432,24125 0,96061 2430,31125 0,92703

2434,17006 0,96022 2432,24006 0,92718

2436,09888 0,9599 2434,16888 0,92751

2438,0277 0,95961 2436,0977 0,92789

2439,95651 0,95941 2438,02651 0,9283

2441,88533 0,95929 2439,95533 0,9287

2443,81414 0,95923 2441,88414 0,9291

2445,74296 0,95926 2443,81296 0,9295

2447,67178 0,95937 2445,74178 0,92996

2449,60059 0,9595 2447,67059 0,93046

2451,52941 0,95966 2449,59941 0,93093

2453,45822 0,95982 2451,52822 0,93146

2455,38704 0,95997 2453,45704 0,93202

2457,31586 0,96013 2455,38586 0,93252

2459,24467 0,96031 2457,31467 0,93299

2461,17349 0,96049 2459,24349 0,93344

2463,1023 0,96069 2461,1723 0,93384

2465,03112 0,96089 2463,10112 0,93421

2466,95994 0,9611 2465,02994 0,93455

2468,88875 0,96134 2466,95875 0,93494

2470,81757 0,96158 2468,88757 0,93536

2472,74638 0,96179 2470,81638 0,93578

2474,6752 0,96197 2472,7452 0,93625

2476,60402 0,96215 2474,67402 0,9366

2478,53283 0,96234 2476,60283 0,93689

2480,46165 0,96253 2478,53165 0,93714

2482,39046 0,96272 2480,46046 0,93731

2484,31928 0,96293 2482,38928 0,93749

2486,2481 0,96314 2484,3181 0,93766

2488,17691 0,96333 2486,24691 0,9378

2490,10573 0,96355 2488,17573 0,93789

2492,03454 0,96379 2490,10454 0,93795

2493,96336 0,96402 2492,03336 0,93795

2495,89218 0,96423 2493,96218 0,93788

2497,82099 0,96445 2495,89099 0,93775

2499,74981 0,96468 2497,81981 0,93758

2501,67862 0,96497 2499,74862 0,93737

2503,60744 0,96525 2501,67744 0,93718

2505,53626 0,96554 2503,60626 0,937

2507,46507 0,96587 2505,53507 0,93685

2509,39389 0,96617 2507,46389 0,93669

2511,3227 0,96644 2509,3927 0,93651

2513,25152 0,9667 2511,32152 0,93639

2515,18034 0,96694 2513,25034 0,93615

2517,10915 0,96714 2515,17915 0,93589

2519,03797 0,96729 2517,10797 0,93568

2520,96678 0,9674 2519,03678 0,93546

2522,8956 0,96747 2520,9656 0,93534

2524,82442 0,96748 2522,89442 0,93526

2526,75323 0,96741 2524,82323 0,93515

2528,68205 0,96729 2526,75205 0,93507

2530,61086 0,9671 2528,68086 0,93508

2532,53968 0,96681 2530,60968 0,93503

2534,4685 0,9665 2532,5385 0,93496

2536,39731 0,9662 2534,46731 0,93493

2538,32613 0,96597 2536,39613 0,93498

2540,25494 0,96574 2538,32494 0,93511

2542,18376 0,96553 2540,25376 0,93531

2544,11258 0,96535 2542,18258 0,93558

2546,04139 0,96516 2544,11139 0,93592

2547,97021 0,96491 2546,04021 0,9363

2549,89902 0,96466 2547,96902 0,93669

2551,82784 0,96438 2549,89784 0,93714

2553,75666 0,96411 2551,82666 0,93753

2555,68547 0,96383 2553,75547 0,93799

2557,61429 0,96362 2555,68429 0,93848

2559,5431 0,9635 2557,6131 0,93896

2561,47192 0,96348 2559,54192 0,93958

2563,40074 0,96351 2561,47074 0,94014

2565,32955 0,96364 2563,39955 0,94068

2567,25837 0,96385 2565,32837 0,94124

2569,18718 0,96411 2567,25718 0,94172

2571,116 0,96437 2569,186 0,94208

2573,04482 0,96468 2571,11482 0,94242

2574,97363 0,96501 2573,04363 0,94271

2576,90245 0,96535 2574,97245 0,94301

2578,83126 0,96565 2576,90126 0,94326

2580,76008 0,96597 2578,83008 0,94345

2582,6889 0,96627 2580,7589 0,94365

2584,61771 0,96657 2582,68771 0,94381

2586,54653 0,96687 2584,61653 0,94393

2588,47534 0,96722 2586,54534 0,944

2590,40416 0,96762 2588,47416 0,94407

2592,33298 0,96808 2590,40298 0,94406

2594,26179 0,96859 2592,33179 0,94406

2596,19061 0,96921 2594,26061 0,94406

2598,11942 0,96991 2596,18942 0,94403

2600,04824 0,97065 2598,11824 0,94405

2601,97706 0,9714 2600,04706 0,94398

2603,90587 0,97218 2601,97587 0,94387

2605,83469 0,97295 2603,90469 0,94376

2607,7635 0,97369 2605,8335 0,94361

2609,69232 0,97442 2607,76232 0,94341

2611,62114 0,97516 2609,69114 0,94325

2613,54995 0,97589 2611,61995 0,94308

2615,47877 0,97663 2613,54877 0,94297

2617,40758 0,97734 2615,47758 0,94284

2619,3364 0,97806 2617,4064 0,9427

2621,26522 0,97875 2619,33522 0,9426

2623,19403 0,97941 2621,26403 0,94249

2625,12285 0,98 2623,19285 0,94238

2627,05166 0,98055 2625,12166 0,94225

2628,98048 0,98103 2627,05048 0,94218

2630,9093 0,98143 2628,9793 0,94207

2632,83811 0,98178 2630,90811 0,94201

2634,76693 0,98211 2632,83693 0,94197

2636,69574 0,98242 2634,76574 0,94193

2638,62456 0,9827 2636,69456 0,94197

2640,55338 0,98295 2638,62338 0,94193

2642,48219 0,98322 2640,55219 0,94188

2644,41101 0,98349 2642,48101 0,94189

2646,33982 0,98379 2644,40982 0,94186

2648,26864 0,98409 2646,33864 0,94189

2650,19746 0,9844 2648,26746 0,94202

2652,12627 0,98471 2650,19627 0,94215

2654,05509 0,98505 2652,12509 0,94236

2655,9839 0,98539 2654,0539 0,94256

2657,91272 0,98579 2655,98272 0,94277

2659,84154 0,98623 2657,91154 0,94304

2661,77035 0,98671 2659,84035 0,9433

2663,69917 0,98726 2661,76917 0,9436

2665,62798 0,98784 2663,69798 0,94395

2667,5568 0,98845 2665,6268 0,94439

2669,48562 0,98907 2667,55562 0,94486

2671,41443 0,98966 2669,48443 0,94543

2673,34325 0,99023 2671,41325 0,94604

2675,27206 0,9908 2673,34206 0,94666

2677,20088 0,99133 2675,27088 0,94731

2679,1297 0,99181 2677,1997 0,9479

2681,05851 0,99227 2679,12851 0,94849

2682,98733 0,99271 2681,05733 0,94908

2684,91614 0,9931 2682,98614 0,94965

2686,84496 0,99348 2684,91496 0,95025

2688,77378 0,99377 2686,84378 0,95082

2690,70259 0,99401 2688,77259 0,95135

2692,63141 0,99419 2690,70141 0,95188

2694,56022 0,99431 2692,63022 0,95237

2696,48904 0,99441 2694,55904 0,95283

2698,41786 0,99446 2696,48786 0,95326

2700,34667 0,99446 2698,41667 0,95364

2702,27549 0,9944 2700,34549 0,95404

2704,2043 0,99429 2702,2743 0,95443

2706,13312 0,99411 2704,20312 0,95483

2708,06194 0,99385 2706,13194 0,95521

2709,99075 0,99355 2708,06075 0,95556

2711,91957 0,99316 2709,98957 0,95587

2713,84838 0,99271 2711,91838 0,95611

2715,7772 0,99225 2713,8472 0,95633

2717,70602 0,99176 2715,77602 0,95646

2719,63483 0,9913 2717,70483 0,95655

2721,56365 0,99083 2719,63365 0,95664

2723,49246 0,99038 2721,56246 0,9567

2725,42128 0,98995 2723,49128 0,95675

2727,3501 0,98952 2725,4201 0,9568

2729,27891 0,98912 2727,34891 0,95685

2731,20773 0,98874 2729,27773 0,95693

2733,13654 0,9884 2731,20654 0,95699

2735,06536 0,98809 2733,13536 0,95706

2736,99418 0,98782 2735,06418 0,95719

2738,92299 0,98759 2736,99299 0,95736

2740,85181 0,98741 2738,92181 0,95761

2742,78062 0,9873 2740,85062 0,95791

2744,70944 0,98724 2742,77944 0,95826

2746,63826 0,98724 2744,70826 0,95862

2748,56707 0,9873 2746,63707 0,95898

2750,49589 0,98741 2748,56589 0,95933

2752,4247 0,98761 2750,4947 0,95967

2754,35352 0,98788 2752,42352 0,96001

2756,28234 0,98823 2754,35234 0,96031

2758,21115 0,98867 2756,28115 0,96063

2760,13997 0,98914 2758,20997 0,96098

2762,06878 0,98964 2760,13878 0,96133

2763,9976 0,99016 2762,0676 0,96166

2765,92642 0,99069 2763,99642 0,96201

2767,85523 0,9912 2765,92523 0,96232

2769,78405 0,99169 2767,85405 0,96259

2771,71286 0,99216 2769,78286 0,96279

2773,64168 0,99254 2771,71168 0,96296

2775,5705 0,99286 2773,6405 0,96308

2777,49931 0,99313 2775,56931 0,96314

2779,42813 0,99334 2777,49813 0,96315

2781,35694 0,99348 2779,42694 0,96305

2783,28576 0,99356 2781,35576 0,96288

2785,21458 0,99356 2783,28458 0,96262

2787,14339 0,9935 2785,21339 0,96226

2789,07221 0,99339 2787,14221 0,96185

2791,00102 0,99323 2789,07102 0,96137

2792,92984 0,99302 2790,99984 0,96086

2794,85866 0,99278 2792,92866 0,96037

2796,78747 0,99248 2794,85747 0,95994

2798,71629 0,99217 2796,78629 0,9596

2800,6451 0,99187 2798,7151 0,95932

2802,57392 0,99158 2800,64392 0,95914

2804,50274 0,99133 2802,57274 0,95907

2806,43155 0,99111 2804,50155 0,95905

2808,36037 0,99095 2806,43037 0,95906

2810,28918 0,9909 2808,35918 0,95914

2812,218 0,99091 2810,288 0,95929

2814,14682 0,991 2812,21682 0,95951

2816,07563 0,99111 2814,14563 0,95979

2818,00445 0,99126 2816,07445 0,96017

2819,93326 0,99138 2818,00326 0,96058

2821,86208 0,99144 2819,93208 0,96104

2823,7909 0,99145 2821,8609 0,96151

2825,71971 0,99138 2823,78971 0,96199

2827,64853 0,99124 2825,71853 0,96248

2829,57734 0,991 2827,64734 0,96296

2831,50616 0,99071 2829,57616 0,96349

2833,43498 0,99036 2831,50498 0,96402

2835,36379 0,98996 2833,43379 0,96459

2837,29261 0,98952 2835,36261 0,96519

2839,22142 0,98906 2837,29142 0,96577

2841,15024 0,98858 2839,22024 0,96635

2843,07906 0,98805 2841,14906 0,96685

2845,00787 0,9875 2843,07787 0,96725

2846,93669 0,98696 2845,00669 0,96756

2848,8655 0,98643 2846,9355 0,96779

2850,79432 0,98594 2848,86432 0,96794

2852,72314 0,98544 2850,79314 0,96801

2854,65195 0,98496 2852,72195 0,96801

2856,58077 0,9845 2854,65077 0,96796

2858,50958 0,98407 2856,57958 0,96783

2860,4384 0,98369 2858,5084 0,96761

2862,36722 0,98333 2860,43722 0,96731

2864,29603 0,98302 2862,36603 0,96692

2866,22485 0,98275 2864,29485 0,96644

2868,15366 0,98253 2866,22366 0,96594

2870,08248 0,98239 2868,15248 0,96547

2872,0113 0,98227 2870,0813 0,96504

2873,94011 0,98216 2872,01011 0,96467

2875,86893 0,98203 2873,93893 0,9643

2877,79774 0,98185 2875,86774 0,96392

2879,72656 0,98161 2877,79656 0,96355

2881,65538 0,9813 2879,72538 0,96312

2883,58419 0,98093 2881,65419 0,96266

2885,51301 0,98052 2883,58301 0,96215

2887,44182 0,98008 2885,51182 0,96162

2889,37064 0,9796 2887,44064 0,96107

2891,29946 0,97908 2889,36946 0,96051

2893,22827 0,97851 2891,29827 0,95994

2895,15709 0,97788 2893,22709 0,95934

2897,0859 0,9772 2895,1559 0,95868

2899,01472 0,97645 2897,08472 0,95797

2900,94354 0,97565 2899,01354 0,95722

2902,87235 0,97482 2900,94235 0,95643

2904,80117 0,97397 2902,87117 0,95564

2906,72998 0,97315 2904,79998 0,95496

2908,6588 0,97238 2906,7288 0,95438

2910,58762 0,97165 2908,65762 0,95387

2912,51643 0,97101 2910,58643 0,95345

2914,44525 0,97042 2912,51525 0,95311

2916,37406 0,96992 2914,44406 0,95284

2918,30288 0,96953 2916,37288 0,95268

2920,2317 0,96926 2918,3017 0,95258

2922,16051 0,9691 2920,23051 0,95263

2924,08933 0,96904 2922,15933 0,95278

2926,01814 0,96907 2924,08814 0,95308

2927,94696 0,96916 2926,01696 0,95347

2929,87578 0,96931 2927,94578 0,95398

2931,80459 0,9695 2929,87459 0,95456

2933,73341 0,96973 2931,80341 0,95521

2935,66222 0,96999 2933,73222 0,95589

2937,59104 0,97025 2935,66104 0,95659

2939,51986 0,97051 2937,58986 0,95732

2941,44867 0,97078 2939,51867 0,95809

2943,37749 0,97104 2941,44749 0,95894

2945,3063 0,97128 2943,3763 0,95987

2947,23512 0,97147 2945,30512 0,96084

2949,16394 0,9716 2947,23394 0,9618

2951,09275 0,97164 2949,16275 0,96276

2953,02157 0,97158 2951,09157 0,96365

2954,95038 0,97144 2953,02038 0,96447

2956,8792 0,97123 2954,9492 0,96523

2958,80802 0,97097 2956,87802 0,9659

2960,73683 0,97066 2958,80683 0,96652

2962,66565 0,97034 2960,73565 0,96705

2964,59446 0,97001 2962,66446 0,96752

2966,52328 0,96967 2964,59328 0,96789

2968,4521 0,96934 2966,5221 0,96817

2970,38091 0,96905 2968,45091 0,96835

2972,30973 0,96881 2970,37973 0,96846

2974,23854 0,96861 2972,30854 0,96845

2976,16736 0,96843 2974,23736 0,96837

2978,09618 0,96831 2976,16618 0,96829

2980,02499 0,96824 2978,09499 0,96816

2981,95381 0,9682 2980,02381 0,96801

2983,88262 0,96819 2981,95262 0,96782

2985,81144 0,96818 2983,88144 0,96756

2987,74026 0,96813 2985,81026 0,96719

2989,66907 0,96809 2987,73907 0,96674

2991,59789 0,96806 2989,66789 0,96617

2993,5267 0,96806 2991,5967 0,96548

2995,45552 0,96807 2993,52552 0,96469

2997,38434 0,96805 2995,45434 0,96377

2999,31315 0,96793 2997,38315 0,96274

3001,24197 0,9677 2999,31197 0,96158

3003,17078 0,96735 3001,24078 0,96036

3005,0996 0,96684 3003,1696 0,95906

3007,02842 0,96615 3005,09842 0,95775

3008,95723 0,96529 3007,02723 0,95644

3010,88605 0,96426 3008,95605 0,95516

3012,81486 0,96311 3010,88486 0,95387

3014,74368 0,96185 3012,81368 0,95266

3016,6725 0,96054 3014,7425 0,9515

3018,60131 0,95918 3016,67131 0,95039

3020,53013 0,9578 3018,60013 0,94933

3022,45894 0,95645 3020,52894 0,94829

3024,38776 0,95516 3022,45776 0,94731

3026,31658 0,95398 3024,38658 0,94642

3028,24539 0,95293 3026,31539 0,94563

3030,17421 0,95204 3028,24421 0,9449

3032,10302 0,95135 3030,17302 0,94422

3034,03184 0,95085 3032,10184 0,94362

3035,96066 0,95057 3034,03066 0,94307

3037,88947 0,95049 3035,95947 0,94255

3039,81829 0,95058 3037,88829 0,94209

3041,7471 0,9508 3039,8171 0,94176

3043,67592 0,95113 3041,74592 0,94152

3045,60474 0,95155 3043,67474 0,94143

3047,53355 0,95199 3045,60355 0,94146

3049,46237 0,95248 3047,53237 0,94162

3051,39118 0,95299 3049,46118 0,94185

3053,32 0,95351 3051,39 0,94218

3055,24882 0,95402 3053,31882 0,94254

3057,17763 0,95448 3055,24763 0,94291

3059,10645 0,9549 3057,17645 0,94322

3061,03526 0,95519 3059,10526 0,94352

3062,96408 0,95536 3061,03408 0,94384

3064,8929 0,95541 3062,9629 0,94418

3066,82171 0,95534 3064,89171 0,94447

3068,75053 0,95512 3066,82053 0,94473

3070,67934 0,95482 3068,74934 0,94491

3072,60816 0,95449 3070,67816 0,94505

3074,53698 0,95412 3072,60698 0,94509

3076,46579 0,95371 3074,53579 0,94504

3078,39461 0,95328 3076,46461 0,94495

3080,32342 0,95283 3078,39342 0,94488

3082,25224 0,95237 3080,32224 0,9448

3084,18106 0,95193 3082,25106 0,94472

3086,10987 0,95153 3084,17987 0,94462

3088,03869 0,95117 3086,10869 0,94449

3089,9675 0,95087 3088,0375 0,94428

3091,89632 0,9506 3089,96632 0,94398

3093,82514 0,95039 3091,89514 0,94354

3095,75395 0,95018 3093,82395 0,94297

3097,68277 0,94995 3095,75277 0,94228

3099,61158 0,94963 3097,68158 0,94153

3101,5404 0,9492 3099,6104 0,94077

3103,46922 0,94863 3101,53922 0,93997

3105,39803 0,94794 3103,46803 0,9391

3107,32685 0,94714 3105,39685 0,93817

3109,25566 0,94621 3107,32566 0,93718

3111,18448 0,94522 3109,25448 0,93615

3113,1133 0,94418 3111,1833 0,93507

3115,04211 0,94309 3113,11211 0,93401

3116,97093 0,94197 3115,04093 0,933

3118,89974 0,94083 3116,96974 0,93209

3120,82856 0,93969 3118,89856 0,93122

3122,75738 0,93851 3120,82738 0,93042

3124,68619 0,9373 3122,75619 0,9297

3126,61501 0,93609 3124,68501 0,92904

3128,54382 0,93487 3126,61382 0,92839

3130,47264 0,93368 3128,54264 0,92775

3132,40146 0,93249 3130,47146 0,92713

3134,33027 0,93132 3132,40027 0,92656

3136,25909 0,93011 3134,32909 0,926

3138,1879 0,9289 3136,2579 0,92554

3140,11672 0,92767 3138,18672 0,92514

3142,04554 0,92642 3140,11554 0,92473

3143,97435 0,92516 3142,04435 0,92431

3145,90317 0,92388 3143,97317 0,92387

3147,83198 0,92264 3145,90198 0,92338

3149,7608 0,92145 3147,8308 0,92281

3151,68962 0,92027 3149,75962 0,92214

3153,61843 0,91913 3151,68843 0,92139

3155,54725 0,918 3153,61725 0,92061

3157,47606 0,91687 3155,54606 0,9198

3159,40488 0,91575 3157,47488 0,91893

3161,3337 0,91462 3159,4037 0,91806

3163,26251 0,91346 3161,33251 0,91717

3165,19133 0,91225 3163,26133 0,91626

3167,12014 0,91104 3165,19014 0,91533

3169,04896 0,9098 3167,11896 0,91443

3170,97778 0,90853 3169,04778 0,91355

3172,90659 0,90725 3170,97659 0,91271

3174,83541 0,90594 3172,90541 0,91195

3176,76422 0,9046 3174,83422 0,91133

3178,69304 0,90324 3176,76304 0,91083

3180,62186 0,90183 3178,69186 0,91046

3182,55067 0,90039 3180,62067 0,91023

3184,47949 0,8989 3182,54949 0,91013

3186,4083 0,89738 3184,4783 0,91013

3188,33712 0,89587 3186,40712 0,91019

3190,26594 0,89437 3188,33594 0,91033

3192,19475 0,89292 3190,26475 0,9106

3194,12357 0,89148 3192,19357 0,91101

3196,05238 0,89013 3194,12238 0,91155

3197,9812 0,88883 3196,0512 0,91221

3199,91002 0,88759 3197,98002 0,91303

3201,83883 0,88636 3199,90883 0,91399

3203,76765 0,88508 3201,83765 0,91507

3205,69646 0,88382 3203,76646 0,91626

3207,62528 0,88254 3205,69528 0,91753

3209,5541 0,88128 3207,6241 0,91885

3211,48291 0,88003 3209,55291 0,92021

3213,41173 0,87878 3211,48173 0,92161

3215,34054 0,87757 3213,41054 0,92302

3217,26936 0,87637 3215,33936 0,92442

3219,19818 0,87517 3217,26818 0,9258

3221,12699 0,87396 3219,19699 0,92731

3223,05581 0,8727 3221,12581 0,92883

3224,98462 0,87137 3223,05462 0,93026

3226,91344 0,86998 3224,98344 0,93148

3228,84226 0,86855 3226,91226 0,9325

3230,77107 0,86705 3228,84107 0,93331

3232,69989 0,86553 3230,76989 0,93382

3234,6287 0,86396 3232,6987 0,93406

3236,55752 0,86235 3234,62752 0,93401

3238,48634 0,86073 3236,55634 0,9337

3240,41515 0,85906 3238,48515 0,93309

3242,34397 0,85735 3240,41397 0,93219

3244,27278 0,85561 3242,34278 0,93103

3246,2016 0,85386 3244,2716 0,92959

3248,13042 0,85213 3246,20042 0,92788

3250,05923 0,85043 3248,12923 0,92597

3251,98805 0,84877 3250,05805 0,92392

3253,91686 0,84712 3251,98686 0,92182

3255,84568 0,84553 3253,91568 0,91975

3257,7745 0,84394 3255,8445 0,9178

3259,70331 0,84235 3257,77331 0,91608

3261,63213 0,84073 3259,70213 0,91445

3263,56094 0,83905 3261,63094 0,91287

3265,48976 0,83735 3263,55976 0,91136

3267,41858 0,83564 3265,48858 0,90998

3269,34739 0,83393 3267,41739 0,90873

3271,27621 0,83226 3269,34621 0,90756

3273,20502 0,83063 3271,27502 0,90655

3275,13384 0,82906 3273,20384 0,90566

3277,06266 0,82754 3275,13266 0,90493

3278,99147 0,82607 3277,06147 0,90429

3280,92029 0,82461 3278,99029 0,90379

3282,8491 0,82311 3280,9191 0,90341

3284,77792 0,8216 3282,84792 0,90309

3286,70674 0,82004 3284,77674 0,90283

3288,63555 0,81847 3286,70555 0,90266

3290,56437 0,81686 3288,63437 0,90253

3292,49318 0,81524 3290,56318 0,90246

3294,422 0,81363 3292,492 0,90244

3296,35082 0,81199 3294,42082 0,90251

3298,27963 0,81036 3296,34963 0,90267

3300,20845 0,8087 3298,27845 0,90283

3302,13726 0,80698 3300,20726 0,90299

3304,06608 0,80522 3302,13608 0,90315

3305,9949 0,8034 3304,0649 0,90329

3307,92371 0,80158 3305,99371 0,90341

3309,85253 0,79973 3307,92253 0,90343

3311,78134 0,79784 3309,85134 0,90345

3313,71016 0,79592 3311,78016 0,90342

3315,63898 0,79399 3313,70898 0,90336

3317,56779 0,79204 3315,63779 0,90322

3319,49661 0,79 3317,56661 0,90302

3321,42542 0,78792 3319,49542 0,90274

3323,35424 0,78579 3321,42424 0,90232

3325,28306 0,78359 3323,35306 0,90178

3327,21187 0,7814 3325,28187 0,90112

3329,14069 0,77919 3327,21069 0,90033

3331,0695 0,77699 3329,1395 0,89949

3332,99832 0,77487 3331,06832 0,89859

3334,92714 0,77279 3332,99714 0,89764

3336,85595 0,77079 3334,92595 0,8966

3338,78477 0,76882 3336,85477 0,89549

3340,71358 0,76689 3338,78358 0,89431

3342,6424 0,76494 3340,7124 0,89311

3344,57122 0,76301 3342,64122 0,89188

3346,50003 0,76111 3344,57003 0,89063

3348,42885 0,75925 3346,49885 0,88934

3350,35766 0,75746 3348,42766 0,8881

3352,28648 0,75568 3350,35648 0,88686

3354,2153 0,754 3352,2853 0,88562

3356,14411 0,7524 3354,21411 0,88438

3358,07293 0,75085 3356,14293 0,88316

3360,00174 0,74939 3358,07174 0,88196

3361,93056 0,74801 3360,00056 0,88079

3363,85938 0,74669 3361,92938 0,87959

3365,78819 0,74549 3363,85819 0,87845

3367,71701 0,74438 3365,78701 0,87741

3369,64582 0,74339 3367,71582 0,87651

3371,57464 0,74245 3369,64464 0,8757

3373,50346 0,74157 3371,57346 0,87497

3375,43227 0,74077 3373,50227 0,87431

3377,36109 0,74005 3375,43109 0,87379

3379,2899 0,73937 3377,3599 0,87336

3381,21872 0,73872 3379,28872 0,87304

3383,14754 0,73816 3381,21754 0,87281

3385,07635 0,73766 3383,14635 0,87263

3387,00517 0,73723 3385,07517 0,87244

3388,93398 0,73692 3387,00398 0,87234

3390,8628 0,73658 3388,9328 0,87216

3392,79162 0,7362 3390,86162 0,87205

3394,72043 0,73578 3392,79043 0,872

3396,64925 0,73526 3394,71925 0,87197

3398,57806 0,73471 3396,64806 0,8719

3400,50688 0,7341 3398,57688 0,87175

3402,4357 0,73345 3400,5057 0,87148

3404,36451 0,73285 3402,43451 0,87116

3406,29333 0,73236 3404,36333 0,87074

3408,22214 0,73204 3406,29214 0,87026

3410,15096 0,73193 3408,22096 0,86958

3412,07978 0,73207 3410,14978 0,86874

3414,00859 0,73244 3412,07859 0,86773

3415,93741 0,73318 3414,00741 0,86661

3417,86622 0,7343 3415,93622 0,86526

3419,79504 0,73575 3417,86504 0,86367

3421,72386 0,73759 3419,79386 0,86184

3423,65267 0,73973 3421,72267 0,85968

3425,58149 0,74224 3423,65149 0,85715

3427,5103 0,74513 3425,5803 0,85437

3429,43912 0,74833 3427,50912 0,85128

3431,36794 0,75186 3429,43794 0,84797

3433,29675 0,75563 3431,36675 0,84442

3435,22557 0,75964 3433,29557 0,84058

3437,15438 0,76397 3435,22438 0,83649

3439,0832 0,76856 3437,1532 0,83215

3441,01202 0,77338 3439,08202 0,82754

3442,94083 0,77833 3441,01083 0,82285

3444,86965 0,78338 3442,93965 0,81803

3446,79846 0,7884 3444,86846 0,8131

3448,72728 0,79343 3446,79728 0,80814

3450,6561 0,79839 3448,7261 0,80346

3452,58491 0,80321 3450,65491 0,799

3454,51373 0,80787 3452,58373 0,79488

3456,44254 0,81228 3454,51254 0,79112

3458,37136 0,81647 3456,44136 0,78784

3460,30018 0,82053 3458,37018 0,785

3462,22899 0,82437 3460,29899 0,78255

3464,15781 0,82799 3462,22781 0,7806

3466,08662 0,83144 3464,15662 0,77922

3468,01544 0,83477 3466,08544 0,77833

3469,94426 0,83792 3468,01426 0,77818

3471,87307 0,841 3469,94307 0,77866

3473,80189 0,84398 3471,87189 0,77982

3475,7307 0,84691 3473,8007 0,78164

3477,65952 0,84977 3475,72952 0,78411

3479,58834 0,85253 3477,65834 0,78704

3481,51715 0,85518 3479,58715 0,79049

3483,44597 0,85767 3481,51597 0,79427

3485,37478 0,86002 3483,44478 0,79838

3487,3036 0,86216 3485,3736 0,80276

3489,23242 0,86412 3487,30242 0,80735

3491,16123 0,8659 3489,23123 0,81208

3493,09005 0,86753 3491,16005 0,81712

3495,01886 0,86903 3493,08886 0,82229

3496,94768 0,87043 3495,01768 0,82743

3498,8765 0,87188 3496,9465 0,83259

3500,80531 0,87329 3498,87531 0,83758

3502,73413 0,87469 3500,80413 0,84238

3504,66294 0,87595 3502,73294 0,84688

3506,59176 0,877 3504,66176 0,85107

3508,52058 0,87795 3506,59058 0,85495

3510,44939 0,87881 3508,51939 0,85818

3512,37821 0,87955 3510,44821 0,86131

3514,30702 0,88021 3512,37702 0,86451

3516,23584 0,88081 3514,30584 0,8676

3518,16466 0,88135 3516,23466 0,87037

3520,09347 0,8819 3518,16347 0,87286

3522,02229 0,88254 3520,09229 0,87492

3523,9511 0,88315 3522,0211 0,87654

3525,87992 0,88364 3523,94992 0,87776

3527,80874 0,88385 3525,87874 0,87866

3529,73755 0,8839 3527,80755 0,87913

3531,66637 0,88397 3529,73637 0,87918

3533,59518 0,88424 3531,66518 0,87921

3535,524 0,88462 3533,594 0,87924

3537,45282 0,88519 3535,52282 0,87915

3539,38163 0,88596 3537,45163 0,8788

3541,31045 0,8869 3539,38045 0,87837

3543,23926 0,88799 3541,30926 0,87782

3545,16808 0,88922 3543,23808 0,87724

3547,0969 0,89029 3545,1669 0,87685

3549,02571 0,891 3547,09571 0,87665

3550,95453 0,89151 3549,02453 0,87689

3552,88334 0,89192 3550,95334 0,87731

3554,81216 0,89222 3552,88216 0,87777

3556,74098 0,8925 3554,81098 0,878

3558,66979 0,8927 3556,73979 0,87821

3560,59861 0,89279 3558,66861 0,8785

3562,52742 0,89269 3560,59742 0,87868

3564,45624 0,89253 3562,52624 0,87891

3566,38506 0,89222 3564,45506 0,87931

3568,31387 0,89181 3566,38387 0,8798

3570,24269 0,89136 3568,31269 0,88038

3572,1715 0,89092 3570,2415 0,88087

3574,10032 0,89054 3572,17032 0,88117

3576,02914 0,89033 3574,09914 0,88165

3577,95795 0,89025 3576,02795 0,88212

3579,88677 0,89032 3577,95677 0,88272

3581,81558 0,89064 3579,88558 0,8832

3583,7444 0,89123 3581,8144 0,88365

3585,67322 0,89222 3583,74322 0,88391

3587,60203 0,89353 3585,67203 0,88378

3589,53085 0,89498 3587,60085 0,88347

3591,45966 0,89699 3589,52966 0,88236

3593,38848 0,89969 3591,45848 0,88078

3595,3173 0,90288 3593,3873 0,87901

3597,24611 0,90641 3595,31611 0,87787

3599,17493 0,91048 3597,24493 0,87699

3601,10374 0,91485 3599,17374 0,87626

3603,03256 0,91942 3601,10256 0,87613

3604,96138 0,92436 3603,03138 0,87638

3606,89019 0,92924 3604,96019 0,87662

3608,81901 0,93367 3606,88901 0,87674

3610,74782 0,93746 3608,81782 0,87692

3612,67664 0,94084 3610,74664 0,87704

3614,60546 0,94407 3612,67546 0,87705

3616,53427 0,94699 3614,60427 0,87723

3618,46309 0,94985 3616,53309 0,87752

3620,3919 0,95264 3618,4619 0,87785

3622,32072 0,95554 3620,39072 0,87886

3624,24954 0,95861 3622,31954 0,8804

3626,17835 0,96193 3624,24835 0,88237

3628,10717 0,96536 3626,17717 0,88446

3630,03598 0,96826 3628,10598 0,88633

3631,9648 0,97075 3630,0348 0,88844

3633,89362 0,97288 3631,96362 0,89093

3635,82243 0,97451 3633,89243 0,89428

3637,75125 0,9758 3635,82125 0,89808

3639,68006 0,97678 3637,75006 0,9021

3641,60888 0,97788 3639,67888 0,90633

3643,5377 0,97917 3641,6077 0,91022

3645,46651 0,98087 3643,53651 0,91328

3647,39533 0,98286 3645,46533 0,91566

3649,32414 0,98483 3647,39414 0,91739

3651,25296 0,98654 3649,32296 0,91833

3653,18178 0,9882 3651,25178 0,91885

3655,11059 0,98991 3653,18059 0,91897

3657,03941 0,99152 3655,10941 0,91881

3658,96822 0,99293 3657,03822 0,91882

3660,89704 0,99437 3658,96704 0,91931

3662,82586 0,99561 3660,89586 0,91962

3664,75467 0,99686 3662,82467 0,91974

3666,68349 0,99806 3664,75349 0,91964

3668,6123 0,99888 3666,6823 0,91987

3670,54112 0,99899 3668,61112 0,92087

3672,46994 0,9983 3670,53994 0,923

3674,39875 0,99737 3672,46875 0,92614

3676,32757 0,99654 3674,39757 0,92903

3678,25638 0,99578 3676,32638 0,93154

3680,1852 0,99547 3678,2552 0,93373

3682,11402 0,99541 3680,18402 0,9356

3684,04283 0,99565 3682,11283 0,93704

3685,97165 0,99585 3684,04165 0,93841

3687,90046 0,99608 3685,97046 0,93954

3689,82928 0,99613 3687,89928 0,94042

3691,7581 0,99569 3689,8281 0,94111

3693,68691 0,99504 3691,75691 0,94164

3695,61573 0,9939 3693,68573 0,94239

3697,54454 0,99255 3695,61454 0,94365

3699,47336 0,9913 3697,54336 0,94524

3701,40218 0,99038 3699,47218 0,94622

3703,33099 0,98997 3701,40099 0,94662

3705,25981 0,98975 3703,32981 0,94637

3707,18862 0,98994 3705,25862 0,94586

3709,11744 0,99005 3707,18744 0,94473

3711,04626 0,99018 3709,11626 0,94282

3712,97507 0,99031 3711,04507 0,93952

3714,90389 0,99066 3712,97389 0,93445

3716,8327 0,99126 3714,9027 0,9283

3718,76152 0,99175 3716,83152 0,92104

3720,69034 0,99233 3718,76034 0,91293

3722,61915 0,99335 3720,68915 0,90375

3724,54797 0,99464 3722,61797 0,8941

3726,47678 0,99612 3724,54678 0,88442

3728,4056 0,99753 3726,4756 0,87507

3730,33442 0,99868 3728,40442 0,86679

3732,26323 0,99916 3730,33323 0,85979

3734,19205 0,99931 3732,26205 0,85448

3736,12086 0,99943 3734,19086 0,85096

3738,04968 0,9997 3736,11968 0,84818

3739,9785 0,99984 3738,0485 0,84551

3741,90731 1 3739,97731 0,84366

3743,83613 0,99994 3741,90613 0,84289

3745,76494 0,99983 3743,83494 0,84327

3747,69376 0,99942 3745,76376 0,84531

3749,62258 0,99894 3747,69258 0,84932

3751,55139 0,99816 3749,62139 0,8543

3753,48021 0,99698 3751,55021 0,86032

3755,40902 0,99537 3753,47902 0,86749

3757,33784 0,99346 3755,40784 0,87551

3759,26666 0,99175 3757,33666 0,88402

3761,19547 0,98992 3759,26547 0,89251

3763,12429 0,98794 3761,19429 0,9012

3765,0531 0,98601 3763,1231 0,90948

3766,98192 0,9839 3765,05192 0,91799

3768,91074 0,98195 3766,98074 0,92703

3770,83955 0,98002 3768,90955 0,93602

3772,76837 0,97851 3770,83837 0,94482

3774,69718 0,97736 3772,76718 0,95238

3776,626 0,97652 3774,696 0,95806

3778,55482 0,97598 3776,62482 0,96188

3780,48363 0,97546 3778,55363 0,96416

3782,41245 0,97528 3780,48245 0,96498

3784,34126 0,9752 3782,41126 0,96452

3786,27008 0,97516 3784,34008 0,96344

3788,1989 0,97524 3786,2689 0,96158

3790,12771 0,97529 3788,19771 0,95887

3792,05653 0,97544 3790,12653 0,95575

3793,98534 0,97573 3792,05534 0,95183

3795,91416 0,97628 3793,98416 0,94646

3797,84298 0,97699 3795,91298 0,93982

3799,77179 0,97758 3797,84179 0,93269

3801,70061 0,97807 3799,77061 0,92586

3803,62942 0,97824 3801,69942 0,91956

3805,55824 0,97826 3803,62824 0,91401

3807,48706 0,97837 3805,55706 0,90889

3809,41587 0,97847 3807,48587 0,90386

3811,34469 0,97884 3809,41469 0,89937

3813,2735 0,97897 3811,3435 0,89438

3815,20232 0,97894 3813,27232 0,88913

3817,13114 0,9788 3815,20114 0,88415

3819,05995 0,97858 3817,12995 0,87975

3820,98877 0,97829 3819,05877 0,87625

3822,91758 0,97769 3820,98758 0,87336

3824,8464 0,97705 3822,9164 0,87146

3826,77522 0,97671 3824,84522 0,8699

3828,70403 0,97645 3826,77403 0,8683

3830,63285 0,97648 3828,70285 0,86659

3832,56166 0,97682 3830,63166 0,86445

3834,49048 0,97724 3832,56048 0,8617

3836,4193 0,97734 3834,4893 0,85914

3838,34811 0,97727 3836,41811 0,85764

3840,27693 0,97699 3838,34693 0,85673

3842,20574 0,97649 3840,27574 0,85576

3844,13456 0,97605 3842,20456 0,85488

3846,06338 0,97557 3844,13338 0,85397

3847,99219 0,97534 3846,06219 0,85281

3849,92101 0,97548 3847,99101 0,852

3851,84982 0,97553 3849,91982 0,85136

3853,77864 0,97564 3851,84864 0,85144

3855,70746 0,97577 3853,77746 0,85219

3857,63627 0,97569 3855,70627 0,85352

3859,56509 0,97532 3857,63509 0,85534

3861,4939 0,97465 3859,5639 0,85734

3863,42272 0,97367 3861,49272 0,85974

3865,35154 0,97235 3863,42154 0,86168

3867,28035 0,97122 3865,35035 0,86335

3869,20917 0,97019 3867,27917 0,86463

3871,13798 0,96932 3869,20798 0,86566

3873,0668 0,96855 3871,1368 0,86664

3874,99562 0,96759 3873,06562 0,86838

3876,92443 0,96686 3874,99443 0,87116

3878,85325 0,9663 3876,92325 0,87414

3880,78206 0,96572 3878,85206 0,87703

3882,71088 0,96514 3880,78088 0,87988

3884,6397 0,96459 3882,7097 0,88239

3886,56851 0,96415 3884,63851 0,88492

3888,49733 0,9638 3886,56733 0,88793

3890,42614 0,96366 3888,49614 0,89084

3890,42496 0,89397

3892,35378 0,89712

3894,28259 0,90015

3896,21141 0,9031

3898,14022 0,9056

3900,06904 0,90774

3901,99786 0,90949

3903,92667 0,91089

3905,85549 0,91214

3907,7843 0,91372

3909,71312 0,91529

3911,64194 0,9173

3913,57075 0,91941

3915,49957 0,92102

3917,42838 0,92226

3919,3572 0,92354

3921,28602 0,92524

3923,21483 0,92721

3925,14365 0,92953

3927,07246 0,93172

3929,00128 0,93381

3930,9301 0,93541

3932,85891 0,93658

3934,78773 0,93745

3936,71654 0,93808

3938,64536 0,93893

3940,57418 0,93982

3942,50299 0,94057

3944,43181 0,94139

3946,36062 0,94223

3948,28944 0,94281

3950,21826 0,94364

3952,14707 0,94454

3954,07589 0,94546

3956,0047 0,9465

3957,93352 0,94782

3959,86234 0,94928

3961,79115 0,95075

3963,71997 0,95225

3965,64878 0,95341

3967,5776 0,95422

3969,50642 0,95503

3971,43523 0,95581

3973,36405 0,95663

3975,29286 0,9575

3977,22168 0,9584

3979,1505 0,95923

3981,07931 0,96007

3983,00813 0,96095

3984,93694 0,96182

3986,86576 0,96254

3988,79458 0,96333

3990,72339 0,96404

3992,65221 0,96457

3994,58102 0,96487

3996,50984 0,96508

3998,43866 0,96526

4000,36747 0,96545

Table S6 show the raw data of the TGA-DSC from Figure 6.

| La(1) | | Pr(2) | |
| --- | --- | --- | --- |
| Temperature (°C) | Mass Loss (%) | Temperature (°C) | Mass Loss (%) |

21,75963 98,20767 21,37504 96,81806

21,733 98,21821 21,35996 96,82359

21,75857 98,22923 21,3576 96,82933

21,74608 98,24071 21,34229 96,83511

21,737 98,25268 21,34311 96,84103

21,74914 98,26515 21,35736 96,84755

21,74643 98,27813 21,3774 96,85399

21,74596 98,2916 21,35418 96,8604

21,7482 98,30557 21,36915 96,86738

21,77247 98,32004 21,36573 96,87459

21,78048 98,33502 21,31506 96,88164

21,80134 98,3505 21,3292 96,88878

21,80994 98,36648 21,37139 96,89631

21,81995 98,38295 21,38399 96,90333

21,82773 98,39989 21,40108 96,91132

21,85954 98,41731 21,42323 96,91914

21,90927 98,43514 21,42841 96,92751

21,95263 98,45335 21,45881 96,93601

21,98079 98,4719 21,47861 96,94461

22,00082 98,49076 21,49393 96,95384

22,03416 98,50989 21,49369 96,96232

22,06362 98,52928 21,53269 96,9715

22,12383 98,54892 21,58218 96,98085

22,14669 98,56878 21,61329 96,99045

22,16212 98,58887 21,63308 97,00018

22,1752 98,60918 21,65499 97,00978

22,19064 98,62971 21,68056 97,01917

22,19441 98,65011 21,72793 97,02964

22,23671 98,6704 21,7548 97,04041

22,2439 98,69051 21,75208 97,05168

22,27382 98,71043 21,76823 97,06319

22,28761 98,7302 21,79745 97,07485

22,35241 98,74979 21,84764 97,08661

22,39542 98,76918 21,88276 97,09853

22,42405 98,7884 21,93248 97,10993

22,45752 98,80743 21,92989 97,12088

22,49946 98,82627 21,97843 97,13123

22,51183 98,84491 21,99257 97,14085

22,56769 98,86336 22,03358 97,15051

22,60551 98,88163 22,06126 97,16022

22,64097 98,8997 22,11287 97,16958

22,67562 98,91757 22,16307 97,17941

22,74348 98,93526 22,21538 97,18868

22,75208 98,95275 22,25226 97,19709

22,76941 98,97005 22,3047 97,20614

22,80452 98,98716 22,33733 97,21517

22,86331 99,00409 22,36019 97,22438

22,92447 99,02082 22,4323 97,23275

22,98291 99,03735 22,45481 97,24144

23,01861 99,05366 22,46023 97,25001

23,05066 99,06979 22,52939 97,25885

23,082 99,0857 22,55095 97,26735

23,12147 99,10143 22,6134 97,27598

23,14504 99,11696 22,67644 97,28509

23,18663 99,13229 22,7158 97,29435

23,22128 99,14742 22,71945 97,30359

23,31954 99,16231 22,75433 97,31258

23,37975 99,17698 22,82785 97,32187

23,43537 99,19143 22,86614 97,33139

23,49758 99,20569 22,89572 97,34092

23,56062 99,21974 22,92341 97,3502

23,60139 99,23358 22,98208 97,36043

23,65405 99,24724 23,004 97,3705

23,69847 99,26069 23,06303 97,38132

23,78378 99,27395 23,12454 97,39245

23,84399 99,28705 23,15399 97,40274

23,88075 99,29998 23,1818 97,4138

23,94014 99,31281 23,24401 97,42442

23,98892 99,32558 23,31388 97,43508

24,04889 99,33831 23,38387 97,44613

24,12572 99,35106 23,4508 97,45681

24,21102 99,36388 23,5341 97,46867

24,26428 99,37675 23,60715 97,48034

24,32826 99,38971 23,67833 97,49198

24,39801 99,40277 23,74254 97,50374

24,45163 99,41593 23,8368 97,51595

24,51655 99,4292 23,89949 97,52859

24,56804 99,44257 23,9399 97,54147

24,64922 99,45607 24,03853 97,55437

24,72734 99,46966 24,11758 97,56781

24,83739 99,48338 24,16955 97,58125

24,92211 99,49724 24,22257 97,59536

25,01107 99,51121 24,30564 97,60973

25,10509 99,52527 24,40261 97,62397

25,16825 99,53945 24,50311 97,63848

25,25332 99,55376 24,58453 97,65307

25,34853 99,56816 24,68622 97,66819

25,43796 99,58262 24,79509 97,68273

25,54577 99,59716 24,88747 97,69798

25,66666 99,61177 24,97454 97,71341

25,78708 99,62642 25,08188 97,7286

25,89795 99,64115 25,17697 97,74418

26,01307 99,65592 25,27206 97,75955

26,12819 99,67074 25,39742 97,77513

26,25273 99,6856 25,49381 97,79096

26,37951 99,70048 25,60492 97,80684

26,49652 99,71537 25,76033 97,82233

26,59019 99,73026 25,88782 97,83809

26,72593 99,74517 26,02921 97,8546

26,83762 99,7601 26,14975 97,87036

26,97218 99,77503 26,29868 97,88607

27,12548 99,78992 26,42888 97,90245

27,27689 99,80479 26,56768 97,91878

27,41522 99,81963 26,68905 97,93553

27,56721 99,83442 26,83515 97,95157

27,70696 99,84915 26,98149 97,9677

27,8361 99,86383 27,13054 97,98376

27,9789 99,87844 27,25049 97,99984

28,11735 99,89298 27,40838 98,01576

28,27571 99,90744 27,58583 98,03209

28,42876 99,9218 27,75326 98,0478

28,61634 99,93604 27,89407 98,06426

28,811 99,95017 28,06551 98,07985

28,95675 99,96415 28,23482 98,09562

29,11169 99,97801 28,39047 98,11053

29,27147 99,99174 28,55449 98,12542

29,44844 100,00533 28,72828 98,1405

29,60786 100,01878 28,91363 98,15537

29,78955 100,03206 29,10957 98,16998

29,95376 100,04519 29,30905 98,18474

30,16526 100,05817 29,51866 98,19931

30,34361 100,07096 29,71779 98,21425

30,54254 100,08357 29,90467 98,22794

30,74415 100,09597 30,09011 98,24187

30,90474 100,10816 30,28049 98,25553

31,08005 100,12016 30,48644 98,26906

31,26063 100,13197 30,65205 98,28276

31,43033 100,14355 30,83239 98,29546

31,64819 100,1549 31,03798 98,30813

31,84081 100,16604 31,21552 98,32053

32,03903 100,17697 31,40789 98,33281

32,23152 100,18767 31,59513 98,34497

32,41864 100,19814 31,79604 98,35649

32,60799 100,2084 32,01799 98,36816

32,80376 100,21847 32,21411 98,3799

33,01425 100,22833 32,41607 98,3912

33,22451 100,23798 32,61079 98,40247

33,43652 100,24742 32,82736 98,41313

33,65871 100,25669 33,04125 98,4241

33,83087 100,26577 33,23141 98,43475

34,04919 100,27469 33,43933 98,44496

34,28213 100,28343 33,62762 98,45515

34,48106 100,29199 33,83017 98,46452

34,68968 100,30037 34,05515 98,47357

34,89819 100,30857 34,23783 98,48288

35,10074 100,3166 34,41958 98,49174

35,32082 100,32446 34,61593 98,50027

35,52664 100,33216 34,83449 98,50895

35,71002 100,33971 35,03073 98,51681

35,90999 100,34708 35,25712 98,52432

36,11172 100,35429 35,4897 98,53268

36,31053 100,36135 35,7092 98,54049

36,51144 100,36827 35,92075 98,54751

36,70289 100,37505 36,10436 98,555

36,90181 100,38169 36,29826 98,56166

37,10401 100,38819 36,49777 98,56852

37,30621 100,39455 36,70534 98,57514

37,50502 100,40077 36,89305 98,58198

37,69763 100,40688 37,10716 98,58823

37,89363 100,41283 37,33625 98,59424

38,09431 100,41866 37,52734 98,59942

38,27068 100,4244 37,71855 98,60522

38,46574 100,43005 37,90882 98,6105

38,63662 100,4356 38,11055 98,61566

38,82455 100,44105 38,2916 98,62085

39,00981 100,44643 38,46656 98,62544

39,21458 100,45171 38,67426 98,63038

39,37587 100,45692 38,85869 98,63525

39,57713 100,46204 39,05083 98,63997

39,7549 100,46709 39,26775 98,64427

39,92693 100,47206 39,46271 98,64864

40,11891 100,47697 39,65123 98,65307

40,29109 100,48182 39,82724 98,65658

40,47301 100,48662 40,01343 98,66135

40,63556 100,49137 40,17971 98,66492

40,8183 100,4961 40,35409 98,66859

41,02052 100,50078 40,52893 98,67219

41,18087 100,50543 40,71283 98,67549

41,35165 100,51007 40,89533 98,67943

41,53253 100,5147 41,08329 98,68355

41,70494 100,51933 41,27914 98,68736

41,86853 100,52395 41,45248 98,69097

42,03236 100,52855 41,62315 98,69462

42,20059 100,53317 41,81342 98,6984

42,37126 100,5378 41,99187 98,70205

42,53253 100,54242 42,15279 98,70573

42,70285 100,54704 42,31302 98,70936

42,87236 100,55166 42,48717 98,71267

43,03421 100,5563 42,66236 98,71606

43,21022 100,56094 42,82282 98,71937

43,36488 100,56556 42,99268 98,72275

43,52336 100,57019 43,14049 98,72611

43,67628 100,57483 43,29492 98,72908

43,83199 100,5795 43,42765 98,73297

44,00266 100,58418 43,58405 98,73682

44,17147 100,58888 43,72293 98,74015

44,33704 100,59361 43,89348 98,74412

44,49633 100,59835 44,08805 98,748

44,64415 100,60313 44,26011 98,75142

44,79509 100,60793 44,4179 98,75541

44,95416 100,61275 44,55678 98,75915

45,12367 100,61762 44,71237 98,76235

45,27937 100,62252 44,86981 98,76623

45,4295 100,62746 45,03699 98,77046

45,59101 100,63243 45,18736 98,77439

45,75901 100,63743 45,3245 98,77811

45,90763 100,64248 45,48635 98,78179

46,05196 100,64758 45,63858 98,78548

46,20419 100,65273 45,79103 98,78916

46,35223 100,65792 45,93687 98,79331

46,5026 100,66315 46,08782 98,79781

46,66271 100,66843 46,24503 98,80214

46,82932 100,67374 46,41895 98,80641

46,97945 100,6791 46,55341 98,80998

47,14618 100,68449 46,72038 98,81432

47,2919 100,68994 46,87619 98,81888

47,449 100,69545 47,0283 98,82355

47,59588 100,70098 47,1782 98,82825

47,73964 100,70656 47,33575 98,83285

47,89255 100,71219 47,47046 98,83744

48,03978 100,71787 47,6525 98,84253

48,18191 100,72359 47,80727 98,84763

48,32416 100,72936 47,94371 98,85231

48,4744 100,73518 48,10127 98,85747

48,62953 100,74103 48,2608 98,86193

48,78639 100,74693 48,41373 98,86691

48,93768 100,75286 48,55736 98,87254

49,09164 100,75884 48,70181 98,87792

49,25234 100,76485 48,84034 98,88287

49,41918 100,7709 48,98931 98,88775

49,56165 100,77698 49,13991 98,89242

49,70622 100,78308 49,27832 98,89779

49,86238 100,78923 49,4331 98,90366

50,03446 100,79541 49,57592 98,90933

50,17997 100,8016 49,74056 98,91494

50,35822 100,80783 49,91297 98,9205

50,50749 100,81407 50,06925 98,92584

50,64464 100,82034 50,21933 98,93174

50,80534 100,82663 50,38535 98,93734

50,95589 100,83294 50,52158 98,94328

51,09904 100,83927 50,67789 98,94879

51,27429 100,84564 50,8192 98,95429

51,42818 100,85203 50,95289 98,96016

51,58161 100,85843 51,11024 98,96632

51,74288 100,86484 51,28815 98,972

51,90879 100,87124 51,43488 98,97771

52,05609 100,87764 51,56452 98,98367

52,237 100,88405 51,70779 98,98949

52,39112 100,89046 51,85013 98,99541

52,53705 100,89687 52,00587 99,00167

52,70583 100,90328 52,13898 99,0075

52,87819 100,90971 52,2938 99,01368

53,04282 100,91611 52,47528 99,01985

53,169 100,92249 52,6264 99,02612

53,34725 100,92886 52,77406 99,03181

53,49998 100,93522 52,96408 99,03798

53,66923 100,94157 53,11012 99,04407

53,82116 100,9479 53,24185 99,05001

53,98925 100,95423 53,4209 99,05596

54,16438 100,96051 53,5913 99,06206

54,31331 100,96678 53,75536 99,06793

54,47805 100,97303 53,91559 99,07334

54,6533 100,97927 54,06648 99,07962

54,82393 100,98547 54,21576 99,08522

54,99791 100,99164 54,36676 99,09179

55,15423 100,99778 54,53555 99,09808

55,326 101,00388 54,70422 99,10356

55,48878 101,00992 54,86122 99,10968

55,65653 101,01594 55,04836 99,11587

55,82243 101,02192 55,19255 99,1218

55,9986 101,02787 55,35498 99,12748

56,15676 101,03378 55,54524 99,13325

56,34205 101,03963 55,72337 99,13874

56,50702 101,04543 55,91455 99,14446

56,69232 101,05119 56,09523 99,15028

56,86918 101,05692 56,25755 99,15624

57,02111 101,0626 56,42264 99,16261

57,20768 101,06825 56,56059 99,16801

57,37957 101,07385 56,74393 99,17315

57,56487 101,07939 56,93834 99,17854

57,70548 101,08487 57,11855 99,1842

57,88281 101,0903 57,28375 99,19016

58,05748 101,09567 57,42274 99,19539

58,23907 101,10098 57,57317 99,20089

58,40508 101,10621 57,77579 99,20628

58,59396 101,1114 57,98082 99,2118

58,76285 101,11652 58,15076 99,21669

58,9291 101,1216 58,34436 99,2219

59,08506 101,1266 58,52353 99,22646

59,27001 101,13155 58,66946 99,23163

59,46165 101,13643 58,814 99,23639

59,6287 101,14126 58,9971 99,24173

59,78941 101,14604 59,1855 99,24691

59,97546 101,15074 59,34251 99,25151

60,14546 101,15537 59,51418 99,25645

60,29507 101,15996 59,70087 99,26152

60,48733 101,1645 59,87126 99,26629

60,64833 101,16901 60,04907 99,27108

60,80426 101,17345 60,22549 99,27597

60,97607 101,17786 60,36648 99,28104

61,17558 101,18222 60,54207 99,28566

61,35118 101,18653 60,7087 99,29033

61,53644 101,19078 60,88395 99,29511

61,71514 101,195 61,06772 99,29957

61,87786 101,1992 61,26355 99,30346

62,04587 101,20335 61,44191 99,30782

62,22504 101,20745 61,60831 99,31166

62,39994 101,2115 61,80897 99,31587

62,56944 101,21551 61,98354 99,32035

62,73825 101,21947 62,15764 99,32461

62,90442 101,22337 62,33749 99,32885

63,08036 101,22724 62,52356 99,33285

63,2524 101,23106 62,68996 99,33733

63,41891 101,23483 62,86003 99,34179

63,58876 101,23856 63,02988 99,34597

63,75481 101,24225 63,20445 99,34965

63,92363 101,2459 63,37475 99,35346

64,09692 101,24951 63,5561 99,35741

64,26091 101,2531 63,73975 99,36157

64,44962 101,25664 63,9318 99,36538

64,59865 101,26014 64,12521 99,36942

64,78252 101,2636 64,27539 99,37338

64,96238 101,26703 64,44547 99,37713

65,1343 101,27045 64,63959 99,38113

65,28918 101,27386 64,81933 99,38504

65,45628 101,27724 64,98745 99,3887

65,63257 101,28057 65,16879 99,3925

65,79011 101,28386 65,34451 99,39598

65,96364 101,28712 65,51804 99,39998

66,14568 101,29035 65,6873 99,40413

66,28735 101,29357 65,84865 99,40794

66,47928 101,29676 66,0216 99,41176

66,64119 101,29996 66,20479 99,41523

66,80001 101,30314 66,3567 99,41898

66,96801 101,30631 66,51941 99,42221

67,13257 101,30946 66,66672 99,42541

67,3176 101,31261 66,81633 99,42891

67,48399 101,31573 67,0285 99,43259

67,66086 101,31883 67,22641 99,4361

67,8345 101,32193 67,40499 99,43999

67,99699 101,32501 67,58438 99,44338

68,1474 101,32808 67,7455 99,44675

68,3299 101,3311 67,92385 99,4501

68,48077 101,33412 68,07507 99,45388

68,64142 101,33714 68,22181 99,45744

68,82461 101,34016 68,38625 99,46

68,97089 101,34315 68,57541 99,46293

69,11313 101,34613 68,73653 99,46656

69,30023 101,34908 68,91799 99,47019

69,4718 101,35201 69,08036 99,47354

69,62981 101,35491 69,25286 99,47677

69,79666 101,35779 69,4334 99,48025

69,96548 101,36067 69,59968 99,48351

70,11742 101,36353 69,74802 99,48665

70,29517 101,36636 69,89902 99,48979

70,46453 101,36916 70,08041 99,49362

70,6193 101,37193 70,24875 99,49713

70,78178 101,3747 70,40834 99,50032

70,94907 101,37743 70,57874 99,50419

71,10867 101,38014 70,74421 99,50743

71,28184 101,38286 70,88611 99,51151

71,42799 101,38556 70,99848 99,51458

71,59598 101,38825 71,16635 99,51827

71,78017 101,39093 71,34975 99,5216

71,96527 101,39363 71,50889 99,52476

72,10351 101,39632 71,66079 99,52846

72,27127 101,399 71,8475 99,53208

72,42719 101,40168 72,0156 99,53518

72,58804 101,40435 72,17739 99,53894

72,75973 101,40701 72,3401 99,54236

72,92265 101,40967 72,49004 99,54613

73,09363 101,41233 72,67584 99,54941

73,2692 101,41498 72,83395 99,55328

73,44351 101,4176 72,99435 99,55694

73,60322 101,42021 73,17176 99,56021

73,76006 101,4228 73,32388 99,56358

73,91611 101,42536 73,48762 99,56677

74,07788 101,42791 73,67606 99,57022

74,23611 101,43045 73,84279 99,57398

74,41376 101,43297 73,98941 99,57746

74,58185 101,43547 74,16452 99,58121

74,75375 101,43796 74,29333 99,58511

74,91748 101,44043 74,47775 99,58932

75,09271 101,44287 74,65561 99,59291

75,26449 101,4453 74,81418 99,59664

75,43867 101,44771 74,98642 99,60006

75,61092 101,45011 75,12579 99,60413

75,77304 101,45251 75,27678 99,60724

75,93632 101,45489 75,41925 99,61075

76,10696 101,45728 75,61195 99,61454

76,26988 101,45966 75,78649 99,61843

76,44029 101,46204 75,93575 99,62216

76,61184 101,46441 76,13027 99,6264

76,79062 101,4668 76,29183 99,62996

76,95401 101,46915 76,42868 99,63298

77,10488 101,47148 76,59427 99,63659

77,27815 101,47381 76,74477 99,64067

77,44867 101,47613 76,92035 99,64492

77,60724 101,47844 77,0827 99,64862

77,77649 101,48074 77,26127 99,65175

77,9415 101,48304 77,44707 99,65526

78,11246 101,48535 77,61896 99,65871

78,28333 101,48765 77,77097 99,66247

78,45441 101,48991 77,90691 99,6668

78,60735 101,49215 78,05651 99,67089

78,78017 101,4944 78,25403 99,67454

78,94425 101,49664 78,40489 99,67819

79,12121 101,49885 78,57898 99,68192

79,27551 101,50107 78,74489 99,6859

79,44431 101,50327 78,92609 99,68891

79,61987 101,50547 79,09478 99,69252

79,80282 101,50764 79,27426 99,69584

79,9686 101,50981 79,42995 99,69955

80,13568 101,51196 79,59012 99,70333

80,30309 101,51409 79,76707 99,70717

80,44741 101,51618 79,95229 99,71056

80,61414 101,51825 80,12132 99,71401

80,79199 101,52028 80,27827 99,71763

80,93689 101,52231 80,44787 99,72113

81,12384 101,52432 80,59633 99,72497

81,27723 101,52631 80,76109 99,72851

81,44397 101,52828 80,95195 99,73148

81,61322 101,53024 81,12762 99,7348

81,78522 101,53219 81,28862 99,73782

81,96114 101,53412 81,45856 99,74115

82,14326 101,53603 81,64837 99,74455

82,28805 101,53792 81,81831 99,74771

82,43948 101,5398 81,97597 99,75135

82,64481 101,54167 82,17142 99,75429

82,79913 101,54353 82,33687 99,75695

82,97321 101,54538 82,49176 99,76001

83,13648 101,54722 82,66757 99,76307

83,29839 101,54903 82,85141 99,76617

83,48062 101,55083 83,04031 99,76862

83,6401 101,55263 83,20438 99,77192

83,8158 101,55441 83,37375 99,77494

83,98642 101,55617 83,54106 99,77834

84,16418 101,55792 83,72226 99,7809

84,3386 101,55964 83,88668 99,78434

84,49555 101,56133 84,05743 99,7873

84,66595 101,563 84,23587 99,79063

84,81579 101,56465 84,39731 99,79367

84,99354 101,56626 84,56438 99,79726

85,16257 101,56782 84,71444 99,80019

85,3201 101,56938 84,89978 99,80282

85,509 101,57094 85,05536 99,80601

85,68182 101,57247 85,22254 99,80888

85,8436 101,57398 85,38077 99,81219

86,02491 101,57545 85,55749 99,81431

86,18945 101,57692 85,7326 99,81752

86,35951 101,57837 85,89978 99,82083

86,51772 101,5798 86,07605 99,82362

86,6871 101,58118 86,24564 99,82641

86,83739 101,58251 86,42248 99,82913

87,01308 101,58386 86,57908 99,83179

87,18106 101,58517 86,7542 99,8342

87,34767 101,58648 86,91219 99,83721

87,52141 101,58778 87,08627 99,84008

87,6856 101,58906 87,25379 99,84267

87,8653 101,59032 87,40972 99,84559

88,03284 101,59156 87,55863 99,8485

88,2068 101,59278 87,74145 99,85108

88,36628 101,59399 87,90081 99,85349

88,54933 101,59518 88,08133 99,85608

88,71088 101,59636 88,2415 99,85787

88,86945 101,59752 88,40455 99,8601

89,03674 101,59866 88,60356 99,86235

89,21381 101,59978 88,75076 99,86434

89,38352 101,6009 88,94493 99,86669

89,55795 101,60198 89,12798 99,86936

89,72984 101,60305 89,26941 99,87111

89,9099 101,60409 89,4543 99,87271

90,07404 101,60509 89,64044 99,87498

90,24117 101,60607 89,79981 99,87744

90,39701 101,60701 89,97646 99,87951

90,56678 101,60794 90,1299 99,8817

90,73794 101,60886 90,2991 99,88418

90,92822 101,60979 90,46312 99,88672

91,0768 101,6107 90,62576 99,88853

91,25165 101,61162 90,79415 99,89093

91,41013 101,61253 90,97406 99,89337

91,57749 101,61343 91,13474 99,89561

91,75844 101,61432 91,31984 99,89767

91,91924 101,61521 91,47326 99,8998

92,10422 101,61609 91,67044 99,90182

92,26328 101,61694 91,85912 99,90426

92,43478 101,61777 92,04305 99,90657

92,6018 101,6186 92,21282 99,90845

92,76064 101,61939 92,38157 99,91107

92,92683 101,62017 92,53476 99,91256

93,08313 101,62094 92,71824 99,91449

93,26282 101,62168 92,87396 99,91629

93,43282 101,62239 93,03487 99,91778

93,59305 101,62311 93,16699 99,91955

93,77145 101,62383 93,33124 99,92148

93,93893 101,62451 93,48005 99,92352

94,117 101,62517 93,64637 99,9255

94,28423 101,62582 93,82111 99,92783

94,42706 101,62644 93,9986 99,92942

94,60041 101,62706 94,15455 99,93161

94,7695 101,62768 94,32007 99,93287

94,92719 101,62828 94,47614 99,93498

95,10456 101,62888 94,66412 99,93645

95,2331 101,62946 94,81004 99,93829

95,41498 101,63006 94,99619 99,93959

95,57979 101,63063 95,17862 99,94095

95,74381 101,6312 95,34771 99,94276

95,90253 101,63174 95,48293 99,94465

96,0791 101,6323 95,67436 99,94608

96,22952 101,63284 95,83561 99,94769

96,42177 101,63339 96,00597 99,9492

96,58798 101,63392 96,15144 99,95117

96,75234 101,63445 96,30843 99,95305

96,92683 101,63496 96,45206 99,95488

97,10007 101,63545 96,60214 99,95684

97,2551 101,63592 96,77065 99,95871

97,42626 101,63638 96,941 99,9603

97,5912 101,63684 97,11884 99,96213

97,75729 101,63731 97,29587 99,96403

97,93777 101,63778 97,43732 99,9653

98,10352 101,63825 97,58096 99,96699

98,25994 101,6387 97,76363 99,96839

98,43744 101,63917 97,9395 99,9704

98,60445 101,63964 98,10398 99,97203

98,75245 101,64012 98,28609 99,9737

98,92442 101,64061 98,45955 99,97535

99,10399 101,64108 98,63451 99,97671

99,26386 101,64153 98,80325 99,97748

99,43167 101,64197 98,95931 99,97842

99,60652 101,64238 99,11319 99,98014

99,77283 101,64278 99,25198 99,98162

99,9333 101,64315 99,44952 99,98264

100,10547 101,64349 99,60675 99,98386

100,25358 101,64383 99,79622 99,98546

100,43995 101,64415 99,94851 99,98641

100,61592 101,64447 100,14233 99,98778

100,77271 101,64478 100,30499 99,98872

100,92673 101,64508 100,47969 99,99005

101,10627 101,64537 100,65509 99,99114

101,26086 101,64564 100,81198 99,99155

101,42725 101,64591 100,9734 99,99268

101,60194 101,64619 101,16277 99,99374

101,76554 101,64648 101,32915 99,99428

101,9451 101,64677 101,4961 99,99476

102,10361 101,64705 101,66525 99,99502

102,29691 101,64732 101,87936 99,99575

102,44458 101,64759 102,01672 99,99687

102,62342 101,64786 102,212 99,99702

102,78679 101,64811 102,38797 99,99702

102,96115 101,64838 102,5645 99,99795

103,13457 101,64862 102,72417 99,99821

103,28801 101,64882 102,87219 99,9986

103,43325 101,64899 103,04584 99,99848

103,61186 101,64916 103,21926 99,99902

103,77179 101,64935 103,38577 99,99888

103,94139 101,64951 103,53445 99,99941

104,11921 101,64966 103,68501 100,00004

104,30209 101,6498 103,87553 99,99992

104,4552 101,64992 104,05173 99,99947

104,62621 101,65 104,18842 99,99931

104,80644 101,65006 104,3452 99,99894

104,9838 101,6501 104,52441 99,99908

105,14509 101,65013 104,69344 99,99865

105,31816 101,65015 104,87402 99,99822

105,50061 101,65016 105,02875 99,99729

105,67693 101,65018 105,23025 99,99623

105,84723 101,6502 105,39881 99,99623

106,00806 101,65023 105,56798 99,99534

106,16183 101,65025 105,71158 99,995

106,34439 101,65022 105,87761 99,99447

106,50928 101,65018 106,04953 99,99414

106,69563 101,65011 106,21349 99,99333

106,85474 101,65003 106,37513 99,99285

107,03082 101,64995 106,53815 99,99153

107,18866 101,64985 106,6872 99,99053

107,36057 101,64973 106,85312 99,98933

107,53307 101,64959 107,02077 99,98839

107,70245 101,64945 107,20228 99,98748

107,85151 101,64929 107,35965 99,9867

108,03683 101,64911 107,53608 99,98563

108,19963 101,64892 107,73434 99,98456

108,34797 101,64871 107,91412 99,9834

108,52672 101,64849 108,09598 99,9825

108,68987 101,64828 108,26733 99,98118

108,86549 101,64803 108,43729 99,97949

109,05566 101,64775 108,61245 99,97857

109,1972 101,64747 108,7674 99,97716

109,36844 101,64717 108,94752 99,97518

109,54959 101,64685 109,10938 99,97329

109,72002 101,64652 109,25936 99,97122

109,88397 101,64618 109,39188 99,96959

110,05212 101,64582 109,57815 99,96808

110,2336 101,64543 109,75214 99,96648

110,39639 101,64504 109,93771 99,96448

110,57115 101,64463 110,08533 99,96297

110,73499 101,64423 110,26228 99,96044

110,89651 101,64381 110,4389 99,95804

111,065 101,64338 110,60075 99,95568

111,2264 101,64294 110,74603 99,9541

111,39465 101,6425 110,92798 99,95203

111,55838 101,64204 111,08694 99,94935

111,73743 101,64159 111,2811 99,94692

111,92635 101,64116 111,44737 99,94425

112,09077 101,64073 111,63245 99,9416

112,26472 101,64031 111,79863 99,93858

112,44272 101,63987 111,95317 99,93588

112,59937 101,63943 112,09705 99,93217

112,77912 101,639 112,26229 99,92854

112,94853 101,63856 112,44017 99,92567

113,11052 101,63813 112,63629 99,92259

113,26891 101,63773 112,7804 99,91939

113,43065 101,63732 112,94772 99,91634

113,59158 101,6369 113,10947 99,91258

113,77853 101,63649 113,28667 99,90916

113,96503 101,63607 113,44552 99,90605

114,1235 101,63565 113,62607 99,90281

114,30072 101,63523 113,82115 99,89898

114,45341 101,63479 114,00449 99,89511

114,61667 101,63433 114,1674 99,89168

114,78296 101,63387 114,30384 99,88793

114,95132 101,63342 114,46014 99,8838

115,10434 101,63298 114,6494 99,88004

115,27563 101,63254 114,77969 99,87592

115,43982 101,6321 114,95689 99,87198

115,60378 101,63166 115,13442 99,86828

115,78248 101,6312 115,31732 99,86434

115,93657 101,63076 115,48347 99,86043

116,11876 101,63031 115,63803 99,85608

116,29792 101,62987 115,81035 99,8517

116,45665 101,62944 115,96501 99,84758

116,61922 101,62904 116,16218 99,84377

116,78748 101,62866 116,3244 99,83866

116,9491 101,62828 116,49846 99,83358

117,10309 101,62791 116,69017 99,82849

117,27911 101,62755 116,85981 99,82368

117,42786 101,62721 117,01228 99,81905

117,61225 101,62688 117,18169 99,81389

117,78713 101,62657 117,34309 99,80881

117,94748 101,62628 117,4879 99,80393

118,1162 101,62602 117,65823 99,79876

118,28097 101,62577 117,83392 99,79384

118,4498 101,62552 117,96735 99,78858

118,61597 101,62533 118,13664 99,78324

118,79897 101,62513 118,31814 99,77761

118,94318 101,62494 118,47455 99,77227

119,10783 101,62476 118,6824 99,76696

119,2639 101,62457 118,83752 99,76242

119,43541 101,62439 119,00566 99,7572

119,60389 101,62422 119,18018 99,75186

119,77203 101,62408 119,34449 99,746

119,9411 101,62394 119,49696 99,74074

120,12049 101,62381 119,69051 99,73544

120,27954 101,62368 119,84752 99,73017

120,45343 101,62355 120,02827 99,72479

120,63374 101,6234 120,21404 99,71947

120,79012 101,62326 120,36269 99,71424

120,96283 101,62311 120,53226 99,70886

121,13287 101,62296 120,6933 99,70449

121,30897 101,62279 120,90433 99,69963

121,47269 101,62264 121,08031 99,69461

121,64204 101,62246 121,24475 99,68954

121,80238 101,6223 121,4184 99,68463

121,9723 101,62213 121,58038 99,67963

122,16358 101,62199 121,75449 99,67473

122,31832 101,62183 121,91029 99,66994

122,47549 101,62167 122,0593 99,66485

122,65372 101,6215 122,21707 99,66024

122,80156 101,62133 122,37659 99,6558

122,9863 101,62118 122,54896 99,65109

123,16463 101,62106 122,70953 99,64687

123,35043 101,62092 122,88319 99,64226

123,52232 101,62079 122,98981 99,63798

123,67683 101,62064 123,15506 99,63355

123,85143 101,6205 123,27698 99,62919

123,99402 101,62037 123,42202 99,6246

124,18366 101,62024 123,55725 99,62016

124,35323 101,62013 123,71198 99,61583

124,53518 101,62001 123,84687 99,61107

124,71808 101,61991 123,98245 99,60726

124,88892 101,61982 124,14409 99,60365

125,0656 101,61972 124,2883 99,59928

125,24194 101,6196 124,45157 99,59459

125,40076 101,61948 124,61307 99,5911

125,58995 101,61936 124,76956 99,58791

125,76313 101,61926 124,9077 99,58462

125,92838 101,61915 125,0691 99,58087

126,09772 101,61904 125,24767 99,57663

126,27838 101,61894 125,40427 99,57303

126,43673 101,61884 125,56122 99,56933

126,61424 101,61872 125,72541 99,56646

126,77739 101,61859 125,86124 99,56313

126,95535 101,61846 126,02694 99,56003

127,10975 101,61833 126,17351 99,55709

127,27721 101,61815 126,3064 99,55416

127,4574 101,61797 126,47562 99,55146

127,63223 101,6178 126,66434 99,54868

127,79456 101,61762 126,86263 99,54577

127,96715 101,61743 127,00125 99,54234

128,14651 101,61726 127,15762 99,53993

128,30278 101,61708 127,29367 99,5378

128,46732 101,61688 127,45355 99,53551

128,64284 101,61666 127,64156 99,53282

128,81487 101,61644 127,82386 99,53027

128,98291 101,61621 127,97241 99,52795

129,15643 101,616 128,15331 99,52547

129,31551 101,6158 128,31805 99,52342

129,48706 101,6156 128,4624 99,52138

129,67087 101,61543 128,63794 99,51918

129,82584 101,61528 128,8208 99,51659

129,98167 101,61513 128,99799 99,5148

130,15439 101,61498 129,16614 99,51253

130,32385 101,61484 129,35391 99,51063

130,49908 101,61468 129,50365 99,50834

130,66537 101,61452 129,67181 99,50634

130,83565 101,61438 129,84009 99,50388

131,01886 101,61423 130,04137 99,50155

131,18634 101,61408 130,2392 99,49985

131,35474 101,61397 130,38869 99,49767

131,50459 101,61386 130,55273 99,49641

131,65154 101,61373 130,75545 99,4943

131,83636 101,61358 130,91635 99,49201

132,01569 101,61343 131,08264 99,48976

132,1828 101,61329 131,27266 99,48816

132,34863 101,61315 131,43002 99,48592

132,49686 101,61301 131,61804 99,48385

132,64105 101,61286 131,81734 99,48226

132,83437 101,6127 131,97833 99,48096

132,99432 101,61253 132,13138 99,47937

133,1787 101,61238 132,34137 99,4783

133,3409 101,61219 132,50729 99,47609

133,50226 101,61198 132,6817 99,47361

133,65468 101,61176 132,85881 99,47203

133,82944 101,61155 133,01865 99,4707

133,99983 101,61136 133,19481 99,46882

134,1779 101,61115 133,33301 99,46722

134,33855 101,61092 133,51974 99,46448

134,50989 101,61069 133,70825 99,46299

134,66853 101,61045 133,88475 99,46193

134,85399 101,6102 134,03084 99,46026

135,01488 101,60995 134,21793 99,45858

135,16144 101,60971 134,39127 99,45661

135,33267 101,60945 134,56671 99,45486

135,50882 101,60918 134,77856 99,45364

135,677 101,60892 134,99878 99,45229

135,84375 101,60868 135,17976 99,45056

135,99608 101,60844 135,31964 99,44852

136,17529 101,60822 135,487 99,44728

136,32185 101,60798 135,65891 99,44561

136,49908 101,60776 135,84164 99,44446

136,67114 101,60752 136,01172 99,44266

136,83354 101,60729 136,18047 99,44054

136,9982 101,60705 136,33723 99,43881

137,17459 101,60683 136,52573 99,43756

137,3376 101,6066 136,70624 99,43609

137,50273 101,60636 136,88382 99,43489

137,68347 101,60611 137,03824 99,43441

137,86502 101,60586 137,22533 99,43294

138,0144 101,6056 137,41759 99,43119

138,19714 101,6053 137,58833 99,43017

138,35263 101,60498 137,75122 99,42915

138,52069 101,60466 137,91504 99,42733

138,6938 101,60433 138,0851 99,42589

138,8871 101,60398 138,26009 99,42481

139,05341 101,60365 138,39809 99,42385

139,21275 101,60328 138,55568 99,42254

139,384 101,6029 138,73174 99,42187

139,5679 101,60253 138,87454 99,4209

139,71106 101,60215 139,03436 99,41919

139,88745 101,60174 139,23766 99,41824

140,05475 101,6013 139,41042 99,41706

140,21159 101,60088 139,59091 99,41601

140,37071 101,60045 139,74312 99,41503

140,55119 101,60002 139,90895 99,41408

140,70264 101,59958 140,08022 99,41324

140,87413 101,59914 140,224 99,41224

141,04918 101,59868 140,39763 99,41115

141,20865 101,59822 140,55402 99,41028

141,38322 101,59776 140,69556 99,40851

141,55861 101,59729 140,88191 99,4071

141,72221 101,5968 141,05981 99,40562

141,88948 101,59634 141,192 99,40505

142,06866 101,59588 141,37306 99,40454

142,22151 101,59541 141,54163 99,40383

142,39348 101,59493 141,70746 99,40341

142,56807 101,59444 141,84521 99,40285

142,72826 101,59392 142,0074 99,40225

142,89055 101,59341 142,16734 99,40254

143,06146 101,59289 142,33472 99,40192

143,22163 101,59236 142,51057 99,40122

143,37543 101,5918 142,69106 99,40096

143,54895 101,59123 142,86127 99,401

143,71906 101,59066 143,02014 99,40103

143,86658 101,59009 143,20239 99,40071

144,03442 101,58951 143,35254 99,40016

144,24168 101,58891 143,50291 99,40008

144,4079 101,58831 143,70181 99,40004

144,5807 101,5877 143,84746 99,40058

144,75526 101,58707 144,02769 99,40086

144,91084 101,58643 144,17784 99,40093

145,07928 101,58577 144,35382 99,40139

145,25256 101,5851 144,53253 99,40134

145,43385 101,58442 144,70276 99,4018

145,58708 101,58374 144,84758 99,40197

145,74866 101,58305 145,02463 99,40229

145,91722 101,58236 145,18126 99,40217

146,07999 101,58167 145,34651 99,40216

146,23758 101,58099 145,49924 99,4018

146,41791 101,58028 145,65872 99,40162

146,58824 101,57958 145,8333 99,40223

146,75623 101,57889 145,98851 99,40237

146,92583 101,57819 146,17476 99,40231

147,10077 101,57751 146,34038 99,40228

147,25964 101,57683 146,50386 99,40271

147,41083 101,57617 146,69437 99,40253

147,57196 101,57552 146,86586 99,40266

147,74594 101,5749 147,02827 99,40315

147,90552 101,57428 147,17902 99,40344

148,05154 101,57364 147,33755 99,40342

148,21832 101,57301 147,47954 99,40431

148,38464 101,57239 147,65764 99,40504

148,56041 101,57179 147,84119 99,40514

148,74028 101,57117 148,00586 99,40547

148,8902 101,57056 148,18385 99,4058

149,0592 101,56995 148,34662 99,40584

149,20416 101,56934 148,51778 99,40602

149,36444 101,56873 148,72046 99,40653

149,54434 101,56814 148,88594 99,40694

149,7175 101,56757 149,04315 99,40714

149,86977 101,56699 149,20569 99,40795

150,04089 101,56642 149,34625 99,40842

150,2048 101,56586 149,50269 99,40897

150,37856 101,56529 149,6599 99,40927

150,56667 101,56473 149,84427 99,40985

150,73166 101,56416 150,01849 99,41041

150,87779 101,5636 150,19638 99,41012

151,05594 101,56305 150,34737 99,41003

151,22176 101,56249 150,54295 99,41025

151,40479 101,56191 150,72383 99,41032

151,56763 101,56135 150,88705 99,41073

151,70972 101,5608 151,06245 99,41085

151,89072 101,56025 151,22366 99,41094

152,05392 101,55972 151,38568 99,41046

152,20526 101,55917 151,5649 99,41043

152,37038 101,55861 151,73355 99,41033

152,53891 101,55805 151,89877 99,41041

152,72395 101,5575 152,07623 99,41029

152,91064 101,55692 152,22282 99,41013

153,09116 101,55632 152,40738 99,41026

153,23694 101,55571 152,57817 99,40999

153,40869 101,55509 152,74103 99,40995

153,5795 101,55446 152,89261 99,40947

153,73439 101,55383 153,05275 99,40965

153,91942 101,55319 153,22093 99,40925

154,06958 101,55252 153,37239 99,4091

154,27396 101,55185 153,5687 99,40863

154,4502 101,55117 153,73357 99,40894

154,6312 101,55047 153,90494 99,40842

154,79062 101,54974 154,10161 99,40804

154,97612 101,54902 154,22697 99,40803

155,14038 101,54829 154,39279 99,4075

155,31226 101,54752 154,55766 99,40704

155,46123 101,54674 154,71021 99,40697

155,64223 101,54593 154,9008 99,40728

155,8186 101,54511 155,08109 99,40676

155,98347 101,54424 155,23811 99,40637

156,16577 101,54332 155,40193 99,40571

156,34096 101,54235 155,59752 99,40561

156,5069 101,54136 155,78088 99,40515

156,67757 101,54033 155,94894 99,405

156,82678 101,53928 156,13387 99,40447

157,00304 101,5382 156,27394 99,40393

157,17206 101,5371 156,42375 99,40334

157,32542 101,53597 156,59563 99,40331

157,49799 101,53484 156,75598 99,4027

157,66228 101,53367 156,9034 99,40268

157,8064 101,5325 157,09377 99,40188

157,98444 101,53129 157,25912 99,40156

158,19437 101,53006 157,41852 99,40075

158,33563 101,52876 157,60701 99,40015

158,51234 101,52744 157,7523 99,39998

158,67496 101,5261 157,92192 99,39936

158,8479 101,52472 158,06567 99,39863

159,01443 101,5233 158,25995 99,39799

159,18463 101,52186 158,43158 99,39748

159,35327 101,5204 158,59029 99,39657

159,50581 101,51892 158,74057 99,3961

159,66524 101,51742 158,88811 99,39577

159,84338 101,51591 159,08203 99,39538

159,99185 101,51437 159,2406 99,39505

160,15601 101,5128 159,39693 99,39416

160,31691 101,51121 159,56465 99,39377

160,50227 101,50961 159,74019 99,39315

160,65189 101,50798 159,89914 99,39287

160,79054 101,50632 160,04846 99,39245

160,96338 101,50463 160,17365 99,39191

161,12015 101,50292 160,3735 99,39103

161,27 101,50117 160,54349 99,38999

161,45203 101,49944 160,71503 99,38941

161,61877 101,49771 160,8855 99,38838

161,79437 101,49596 161,06084 99,38777

161,94849 101,49419 161,24605 99,38714

162,10919 101,4924 161,4196 99,38633

162,27618 101,49059 161,58234 99,38531

162,44177 101,48876 161,75446 99,38434

162,60735 101,48689 161,90192 99,38394

162,75099 101,48498 162,10095 99,38314

162,93123 101,48306 162,25784 99,38181

163,10049 101,48113 162,42329 99,38139

163,28822 101,47917 162,58209 99,3808

163,44463 101,4772 162,7522 99,37992

163,5766 101,47519 162,92754 99,37959

163,71324 101,47316 163,09526 99,37935

163,908 101,47113 163,26689 99,3787

164,06369 101,46907 163,44307 99,37754

164,22604 101,46699 163,59937 99,37687

164,41556 101,46491 163,78638 99,37591

164,56291 101,46284 163,94623 99,37532

164,72266 101,46076 164,12251 99,37391

164,87415 101,45867 164,2769 99,37271

165,05867 101,4566 164,45905 99,37138

165,21472 101,45448 164,61021 99,3702

165,37469 101,45236 164,7704 99,3691

165,5479 101,45021 164,94159 99,36809

165,71515 101,44805 165,10608 99,36682

165,89857 101,44587 165,29323 99,36585

166,0719 101,44365 165,43248 99,36515

166,23843 101,4414 165,61008 99,36459

166,38495 101,43913 165,78839 99,36316

166,53624 101,43684 165,93634 99,36266

166,70383 101,43457 166,11275 99,36138

166,8707 101,43229 166,26465 99,35977

167,02582 101,42997 166,40234 99,35882

167,19365 101,42763 166,59877 99,35744

167,37898 101,42529 166,76373 99,35577

167,55743 101,42292 166,93301 99,35447

167,70787 101,42054 167,10109 99,35283

167,87012 101,41812 167,29799 99,3516

168,04474 101,41568 167,44032 99,35061

168,22449 101,41321 167,60591 99,34883

168,39102 101,41072 167,7804 99,34726

168,53183 101,40822 167,96124 99,34597

168,70381 101,4057 168,15672 99,34463

168,85963 101,40313 168,34563 99,34293

169,02354 101,40056 168,49786 99,34115

169,18663 101,39798 168,63081 99,33875

169,37314 101,39538 168,7896 99,33693

169,52441 101,39276 168,94957 99,33514

169,69931 101,39013 169,12895 99,3333

169,83795 101,38745 169,32408 99,33135

170,02017 101,38472 169,50609 99,3295

170,16547 101,38196 169,64368 99,32711

170,34454 101,37921 169,82414 99,32507

170,51385 101,37645 169,99046 99,32307

170,68497 101,37366 170,17834 99,32097

170,86096 101,37087 170,34862 99,31915

171,01642 101,36811 170,49944 99,31692

171,21857 101,36535 170,6722 99,31403

171,38705 101,36256 170,84856 99,31178

171,55255 101,35975 171,00438 99,3091

171,72522 101,35691 171,14539 99,30615

171,88054 101,35407 171,29964 99,30327

172,05319 101,35121 171,50885 99,30037

172,24675 101,34834 171,68768 99,2976

172,41107 101,34546 171,87398 99,29476

172,58131 101,34255 172,02205 99,29152

172,75995 101,33964 172,18036 99,2886

172,92294 101,33668 172,35268 99,28625

173,09094 101,3337 172,50156 99,28339

173,25296 101,33069 172,65164 99,28062

173,40546 101,32767 172,83484 99,27775

173,57452 101,32463 172,99243 99,27534

173,75899 101,32157 173,14325 99,27209

173,92557 101,3185 173,32771 99,26805

174,09203 101,31542 173,48294 99,26464

174,26849 101,31233 173,65225 99,2607

174,46048 101,30922 173,83221 99,25777

174,63863 101,3061 174,00354 99,25368

174,77702 101,30298 174,19649 99,24951

174,93535 101,29984 174,34692 99,24563

175,12091 101,29668 174,52617 99,24116

175,29285 101,29349 174,72198 99,23778

175,45905 101,29029 174,89058 99,23396

175,6255 101,28708 175,04628 99,22933

175,78444 101,28385 175,2038 99,22475

175,94157 101,28062 175,37595 99,22039

176,12927 101,27737 175,53751 99,21554

176,28796 101,27409 175,69093 99,2114

176,45773 101,27082 175,86299 99,20679

176,64032 101,26753 176,03566 99,20132

176,7959 101,26425 176,2168 99,19591

176,98586 101,26094 176,38849 99,19082

177,10359 101,2576 176,53632 99,18519

177,29619 101,25426 176,70683 99,17986

177,4593 101,25092 176,87111 99,174

177,62776 101,24756 177,08282 99,1683

177,80246 101,24418 177,24557 99,16246

177,9707 101,24079 177,42049 99,15664

178,12842 101,23739 177,55913 99,15049

178,28198 101,234 177,72174 99,14441

178,45236 101,23063 177,9073 99,13802

178,62372 101,22727 178,08722 99,13082

178,79865 101,22391 178,27252 99,12427

178,97369 101,22051 178,44997 99,11737

179,11938 101,21708 178,62323 99,11002

179,30072 101,21366 178,77774 99,10277

179,47185 101,21019 178,95158 99,09574

179,62946 101,20669 179,12103 99,08854

179,79018 101,20316 179,26862 99,08156

179,96425 101,19963 179,42169 99,07426

180,11794 101,19609 179,59515 99,06731

180,30621 101,19253 179,74445 99,0597

180,46062 101,18895 179,92377 99,05229

180,62251 101,18536 180,07672 99,0442

180,80223 101,18178 180,25034 99,03555

180,98193 101,17817 180,4364 99,0273

181,16853 101,17456 180,60474 99,0191

181,32794 101,17094 180,7632 99,01055

181,48772 101,16732 180,9276 99,00205

181,65189 101,16369 181,12698 98,99341

181,82979 101,16006 181,29236 98,98463

182,00806 101,15642 181,42229 98,97543

182,17654 101,15275 181,5891 98,96595

182,34599 101,14906 181,77272 98,95649

182,51877 101,14534 181,92389 98,9479

182,66908 101,14162 182,0993 98,93804

182,83528 101,13787 182,2532 98,92847

183,02275 101,13409 182,43137 98,91681

183,19398 101,1303 182,61607 98,90491

183,37836 101,1265 182,75505 98,89277

183,52031 101,12268 182,93201 98,88059

183,68903 101,11885 183,11292 98,86815

183,85559 101,11501 183,29691 98,85387

184,02454 101,11113 183,42596 98,83978

184,22514 101,10724 183,61261 98,82554

184,38406 101,1033 183,77142 98,81172

184,56401 101,09934 183,96556 98,79689

184,73584 101,09535 184,12175 98,78134

184,8858 101,09134 184,27518 98,76482

185,05177 101,08727 184,42096 98,74773

185,22169 101,08316 184,60139 98,73055

185,40137 101,07901 184,77748 98,71366

185,58418 101,07485 184,91028 98,69496

185,74646 101,07068 185,08879 98,67641

185,91064 101,06644 185,27206 98,65662

186,07565 101,06216 185,41129 98,63679

186,2292 101,05783 185,57393 98,6169

186,38693 101,05347 185,77081 98,59642

186,58131 101,04906 185,92688 98,57592

186,75482 101,04458 186,09799 98,55481

186,935 101,04002 186,27208 98,53282

187,09787 101,03538 186,44221 98,51101

187,27411 101,03067 186,62549 98,48906

187,42883 101,02591 186,78539 98,46591

187,59959 101,02108 186,9375 98,44261

187,73511 101,01619 187,12317 98,4192

187,90192 101,01123 187,28365 98,39553

188,07375 101,0062 187,44783 98,37048

188,23195 101,0011 187,64307 98,34456

188,39722 100,99591 187,79889 98,31896

188,58301 100,99063 187,96915 98,29279

188,7516 100,98527 188,15565 98,26574

188,90968 100,97982 188,27759 98,23928

189,071 100,97429 188,45177 98,21163

189,24187 100,96866 188,63612 98,18396

189,42824 100,96288 188,80042 98,15646

189,5873 100,95701 188,93011 98,12828

189,75183 100,95103 189,12447 98,10057

189,91028 100,94494 189,30347 98,07211

190,09851 100,93873 189,46252 98,04312

190,25002 100,93241 189,6335 98,01441

190,40791 100,92596 189,79852 97,9856

190,5728 100,91939 189,94933 97,95669

190,72923 100,91271 190,11842 97,92659

190,89818 100,90594 190,26782 97,89609

191,08861 100,89904 190,4252 97,86486

191,25159 100,89202 190,58974 97,8339

191,42807 100,88485 190,78017 97,80264

191,60167 100,87756 190,93207 97,77244

191,7415 100,87015 191,11497 97,74265

191,91692 100,86258 191,27199 97,71237

192,07069 100,85487 191,42926 97,68182

192,24777 100,84703 191,61743 97,65153

192,43414 100,83903 191,78531 97,62221

192,589 100,83091 191,9483 97,59244

192,75464 100,82266 192,10852 97,56317

192,90999 100,81428 192,2881 97,53404

193,06882 100,80578 192,427 97,50486

193,23442 100,79715 192,59474 97,47607

193,40814 100,78838 192,79614 97,44772

193,59212 100,77951 192,96356 97,41959

193,73186 100,77051 193,13513 97,39116

193,90593 100,76139 193,32819 97,36248

194,06487 100,75212 193,49692 97,33485

194,23798 100,7427 193,66325 97,30687

194,41602 100,73318 193,81773 97,27856

194,57961 100,72352 193,97491 97,25043

194,75665 100,71374 194,16293 97,22216

194,92468 100,70386 194,34526 97,19424

195,08635 100,69386 194,53473 97,16532

195,25661 100,68377 194,6828 97,13625

195,43321 100,67355 194,84138 97,10774

195,59546 100,66325 194,97646 97,07878

195,75739 100,65287 195,1658 97,04954

195,92311 100,64238 195,32152 97,02105

196,09302 100,63183 195,48808 96,99223

196,27797 100,62119 195,64258 96,96316

196,44466 100,61048 195,80595 96,934

196,58748 100,59971 195,95663 96,90548

196,74318 100,58887 196,14276 96,87702

196,92383 100,578 196,29335 96,84795

197,08897 100,56708 196,50037 96,81913

197,22833 100,55612 196,65465 96,78928

197,38786 100,54512 196,82716 96,75958

197,55251 100,53408 197,01787 96,73046

197,72589 100,52303 197,15076 96,70132

197,8959 100,51196 197,34694 96,67212

198,05292 100,50088 197,5098 96,64293

198,24992 100,48978 197,67134 96,61295

198,42996 100,47868 197,79782 96,58374

198,57745 100,46758 197,952 96,55447

198,7674 100,45649 198,14252 96,52451

198,93134 100,44542 198,32808 96,49497

199,12128 100,43436 198,49965 96,46475

199,28476 100,42333 198,68425 96,4359

199,47507 100,41232 198,85083 96,40761

199,64093 100,40136 199,01474 96,37948

199,81371 100,39043 199,19098 96,35132

199,97725 100,37957 199,35875 96,32288

200,12787 100,36877 199,5462 96,29517

200,31297 100,35801 199,73688 96,26754

200,46387 100,34732 199,88554 96,24077

200,62083 100,3367 200,05724 96,21405

200,79974 100,32616 200,23232 96,18772

200,98232 100,31571 200,41789 96,16156

201,16074 100,30532 200,58321 96,13562

201,32272 100,29502 200,74232 96,10992

201,49579 100,28482 200,91849 96,08369

201,65454 100,27471 201,12419 96,05826

201,81641 100,26467 201,31487 96,03305

201,99602 100,25475 201,45825 96,00841

202,15382 100,24493 201,65158 95,98357

202,33679 100,2352 201,82855 95,95934

202,48351 100,22558 201,98674 95,93535

202,65776 100,2161 202,13702 95,91207

202,8194 100,20675 202,29773 95,88921

202,99626 100,19754 202,4503 95,86763

203,16264 100,18844 202,63953 95,84632

203,32404 100,17948 202,83142 95,82601

203,51163 100,17063 203,00876 95,80545

203,70111 100,16189 203,14479 95,78574

203,86703 100,15326 203,28986 95,76661

204,01483 100,14479 203,49901 95,74847

204,18767 100,13644 203,68098 95,73069

204,35786 100,1282 203,83739 95,71397

204,52032 100,12006 203,99042 95,69682

204,69017 100,11204 204,15158 95,68038

204,84247 100,10414 204,32237 95,66451

205,02104 100,09636 204,47876 95,64903

205,18585 100,08869 204,64478 95,63391

205,3313 100,08115 204,80544 95,61968

205,5009 100,07371 204,95956 95,60597

205,65395 100,06636 205,12488 95,59303

205,83783 100,05913 205,2852 95,58179

206,01173 100,05198 205,45895 95,57033

206,16811 100,04497 205,63918 95,55915

206,36391 100,03806 205,80746 95,5481

206,51613 100,03125 205,96884 95,53782

206,65846 100,02455 206,14381 95,52765

206,82045 100,01796 206,35356 95,51805

207,00281 100,01145 206,51755 95,50893

207,18552 100,00505 206,66884 95,50079

207,3432 99,99876 206,88799 95,49313

207,51079 99,99256 207,04413 95,48525

207,66608 99,98648 207,20171 95,47763

207,81523 99,98049 207,3768 95,47041

207,97255 99,97458 207,51935 95,46337

208,13464 99,96875 207,6642 95,45657

208,32162 99,96304 207,85095 95,45046

208,49399 99,95738 208,00244 95,44519

208,66122 99,95183 208,14133 95,43907

208,81987 99,94636 208,30273 95,43344

208,99435 99,94099 208,43837 95,42736

209,15631 99,93571 208,5938 95,42209

209,30688 99,9305 208,73137 95,41693

209,48352 99,92539 208,89099 95,41156

209,63942 99,92037 209,02187 95,40678

209,80865 99,91541 209,14323 95,40211

209,97836 99,91053 209,31604 95,39791

210,13373 99,90572 209,4585 95,39382

210,30267 99,90097 209,58557 95,38991

210,45941 99,89628 209,73935 95,38593

210,61981 99,89164 209,89011 95,38271

210,77524 99,88706 210,03093 95,38005

210,96384 99,88251 210,17091 95,37671

211,15073 99,878 210,32101 95,37378

211,31482 99,87353 210,50969 95,37114

211,47404 99,86907 210,60687 95,36826

211,62732 99,86468 210,75482 95,3658

211,80676 99,86029 210,88966 95,36307

211,97583 99,85597 211,02429 95,36122

212,16449 99,8517 211,17725 95,3592

212,28725 99,8475 211,33826 95,35745

212,46738 99,84331 211,49316 95,35543

212,61317 99,83915 211,63515 95,35385

212,78737 99,83508 211,76788 95,35179

212,97392 99,83106 211,92531 95,35026

213,15239 99,82708 212,09608 95,34894

213,32813 99,82316 212,28012 95,34822

213,49402 99,81926 212,42758 95,34724

213,64799 99,81539 212,58337 95,3463

213,83929 99,81157 212,75339 95,3457

214,02606 99,8078 212,90121 95,34558

214,16676 99,80407 213,05852 95,34486

214,36533 99,80038 213,19623 95,34417

214,5224 99,79678 213,35393 95,34389

214,69504 99,7932 213,52133 95,34376

214,84772 99,78966 213,70622 95,34391

215,03203 99,78619 213,88361 95,34341

215,20978 99,78276 214,04639 95,34347

215,37363 99,77939 214,20432 95,34416

215,54807 99,77608 214,38112 95,3443

215,73566 99,77278 214,5351 95,34375

215,89502 99,76954 214,71501 95,34399

216,05212 99,76631 214,88979 95,34409

216,21523 99,76313 215,06125 95,34513

216,39978 99,75996 215,25659 95,34619

216,55151 99,75683 215,40073 95,34734

216,71762 99,7537 215,58359 95,34852

216,89645 99,7506 215,74887 95,34996

217,0293 99,74752 215,92804 95,35152

217,21144 99,74446 216,1102 95,3534

217,38779 99,74142 216,27606 95,3548

217,52667 99,73842 216,45798 95,35651

217,66867 99,73541 216,6467 95,35818

217,85605 99,73245 216,82016 95,35973

218,03949 99,7295 217,01801 95,36168

218,2005 99,72654 217,1967 95,36337

218,36841 99,7236 217,36865 95,36499

218,53558 99,72068 217,53406 95,36669

218,70659 99,71776 217,71025 95,3682

218,89124 99,71486 217,8703 95,36987

219,05058 99,71199 218,06792 95,37132

219,21643 99,70916 218,23451 95,37239

219,38885 99,70637 218,42592 95,37332

219,52821 99,70358 218,59904 95,37496

219,6967 99,70085 218,78513 95,37629

219,85759 99,69814 218,97321 95,3776

220,03864 99,69547 219,155 95,37892

220,19415 99,69281 219,28381 95,38035

220,35768 99,6902 219,47035 95,38147

220,54253 99,68759 219,66154 95,38276

220,69788 99,68501 219,85748 95,38423

220,85632 99,68245 220,05522 95,38602

221,03322 99,67993 220,24884 95,38729

221,21095 99,67745 220,42529 95,3885

221,36157 99,67495 220,62646 95,39039

221,52451 99,67246 220,80315 95,39201

221,69482 99,66999 220,97128 95,39334

221,85242 99,66752 221,14677 95,39445

222,01935 99,6651 221,30237 95,396

222,21074 99,66269 221,47266 95,39758

222,34914 99,66032 221,63605 95,39912

222,52582 99,65796 221,82977 95,40074

222,69704 99,65563 222,00623 95,40266

222,86186 99,65329 222,18939 95,40412

223,02199 99,65096 222,33966 95,40533

223,18811 99,64864 222,50237 95,40648

223,34784 99,64635 222,69431 95,40845

223,5199 99,64403 222,84685 95,40993

223,67502 99,64173 223,02373 95,41191

223,85051 99,63942 223,17651 95,41343

224,02008 99,6371 223,35817 95,4154

224,16571 99,6348 223,53021 95,41714

224,327 99,6325 223,6768 95,41856

224,49928 99,6302 223,84186 95,42053

224,65442 99,6279 223,9957 95,42253

224,8248 99,62563 224,15671 95,42424

224,99744 99,62339 224,32936 95,42597

225,16122 99,62119 224,49219 95,42739

225,34262 99,619 224,62778 95,42834

225,52487 99,61682 224,80669 95,42965

225,67856 99,61468 224,97473 95,43122

225,84636 99,61255 225,1078 95,4331

225,99957 99,61041 225,28154 95,43483

226,17603 99,6083 225,45642 95,43629

226,35162 99,60618 225,60361 95,43795

226,51894 99,60409 225,75342 95,43949

226,70285 99,60203 225,9373 95,44112

226,86853 99,59999 226,07219 95,44244

227,01569 99,59797 226,20966 95,44415

227,17697 99,59599 226,39438 95,44604

227,35129 99,59404 226,56039 95,44765

227,52533 99,59212 226,72546 95,44949

227,67644 99,5902 226,87607 95,45183

227,83212 99,58829 227,01465 95,45382

228,01286 99,58643 227,16547 95,4558

228,15683 99,58459 227,33742 95,45759

228,32167 99,58278 227,50131 95,45936

228,51076 99,58098 227,66481 95,46145

228,68034 99,57921 227,82776 95,46389

228,86186 99,57745 228,00278 95,4659

229,02515 99,57573 228,13812 95,46791

229,17531 99,57402 228,29053 95,46995

229,36345 99,57231 228,45917 95,47167

229,53281 99,5706 228,63226 95,47417

229,70757 99,56889 228,79022 95,47628

229,87286 99,56721 228,9334 95,47808

230,01508 99,56555 229,08022 95,47999

230,21848 99,56392 229,24695 95,4825

230,39104 99,56233 229,42834 95,48513

230,55873 99,56074 229,57245 95,48704

230,72714 99,55916 229,73007 95,48869

230,88367 99,55757 229,88922 95,49054

231,03293 99,55601 230,08037 95,49266

231,21742 99,55446 230,25847 95,4947

231,39633 99,55294 230,42888 95,49655

231,56569 99,55142 230,56628 95,49892

231,7328 99,54994 230,7334 95,50167

231,88884 99,54845 230,92445 95,50429

232,03131 99,54698 231,09828 95,50692

232,21388 99,54554 231,24252 95,50896

232,38066 99,54413 231,41579 95,51102

232,53183 99,54273 231,60637 95,51268

232,73129 99,54137 231,75146 95,51455

232,8912 99,54005 231,90663 95,51645

233,04379 99,53876 232,08545 95,5179

233,20998 99,53751 232,25659 95,51977

233,37122 99,53629 232,44104 95,52113

233,5578 99,53507 232,62029 95,52257

233,72136 99,53385 232,82443 95,52453

233,88164 99,53268 232,99309 95,52653

234,06389 99,53153 233,15607 95,52789

234,22992 99,5304 233,33286 95,52939

234,38593 99,52928 233,52333 95,53102

234,5683 99,52818 233,71301 95,53289

234,73163 99,5271 233,89828 95,53529

234,88872 99,52605 234,07574 95,53744

235,05231 99,52501 234,26389 95,53953

235,21127 99,524 234,4395 95,54151

235,39775 99,52301 234,61487 95,54391

235,55083 99,52206 234,80688 95,54595

235,71356 99,5211 234,97083 95,54785

235,89804 99,52015 235,13828 95,54972

236,07593 99,51922 235,29713 95,55183

236,22144 99,51831 235,4677 95,55346

236,39125 99,51744 235,67006 95,55564

236,57349 99,51657 235,84802 95,55705

236,74756 99,51572 236,01266 95,55885

236,90251 99,51487 236,17554 95,56064

237,08014 99,51404 236,34384 95,56257

237,24985 99,51322 236,51099 95,56415

237,42145 99,51243 236,70085 95,56617

237,57207 99,51165 236,87151 95,56792

237,72173 99,5109 237,01678 95,56967

237,91254 99,51017 237,21318 95,57192

238,07492 99,50947 237,38843 95,57366

238,25526 99,50882 237,52946 95,5757

238,41745 99,50818 237,68361 95,57783

238,60674 99,50759 237,87883 95,58

238,74977 99,507 237,99887 95,58162

238,93576 99,50643 238,14311 95,58289

239,09831 99,50587 238,33818 95,58512

239,27521 99,50535 238,49774 95,58719

239,44891 99,50484 238,64386 95,58909

239,62244 99,50437 238,79745 95,59082

239,77939 99,50393 238,95111 95,59241

239,96095 99,50351 239,11174 95,59473

240,11823 99,50312 239,26953 95,59688

240,29189 99,50275 239,43361 95,59849

240,46107 99,50241 239,6322 95,59968

240,60907 99,50209 239,78589 95,60168

240,77173 99,50178 239,97527 95,60408

240,95593 99,50151 240,14601 95,60657

241,12009 99,50127 240,32297 95,60864

241,29553 99,50105 240,47588 95,61117

241,46425 99,50082 240,63934 95,61318

241,60519 99,5006 240,83203 95,61561

241,75539 99,50038 241,00275 95,61773

241,91582 99,50017 241,17863 95,6201

242,09674 99,49999 241,35561 95,62222

242,25986 99,49983 241,52751 95,6244

242,43622 99,49969 241,69543 95,62645

242,59734 99,49955 241,86418 95,62869

242,7664 99,49943 242,05377 95,63122

242,93681 99,49935 242,21608 95,63335

243,07387 99,49931 242,43034 95,63557

243,26007 99,4993 242,62514 95,63738

243,42883 99,4993 242,77641 95,63898

243,59955 99,49933 242,95062 95,64095

243,77264 99,49936 243,15073 95,64294

243,91815 99,4994 243,31653 95,64471

244,08948 99,49947 243,49594 95,64683

244,25165 99,49958 243,67877 95,64866

244,42107 99,49972 243,82874 95,65114

244,5835 99,49988 244,00639 95,65286

244,75867 99,50007 244,181 95,65472

244,94397 99,50028 244,37155 95,65634

245,09641 99,50051 244,56718 95,65852

245,25482 99,50074 244,75905 95,6606

245,41345 99,50101 244,93987 95,66291

245,59583 99,5013 245,1161 95,66421

245,76161 99,50161 245,2953 95,66603

245,90854 99,50194 245,46404 95,66784

246,09674 99,5023 245,65073 95,66952

246,26466 99,50268 245,81125 95,67094

246,44476 99,50306 245,97353 95,67271

246,61472 99,50347 246,1635 95,67391

246,77313 99,50387 246,35149 95,675

246,95029 99,5043 246,51459 95,67637

247,13734 99,50471 246,69086 95,67773

247,3007 99,50517 246,87578 95,67945

247,48586 99,50563 247,05168 95,68165

247,64954 99,50611 247,22055 95,68309

247,81982 99,50662 247,36969 95,68496

247,99045 99,50716 247,52751 95,68684

248,16293 99,50771 247,70612 95,68859

248,336 99,50826 247,86227 95,69001

248,49841 99,50881 248,02386 95,69132

248,66457 99,50936 248,20366 95,69266

248,81898 99,50995 248,38823 95,69437

248,97668 99,51056 248,5464 95,69605

249,14816 99,5112 248,70399 95,69752

249,33563 99,51186 248,8746 95,69848

249,48854 99,5125 249,037 95,69929

249,6633 99,51318 249,18442 95,70107

249,83661 99,51387 249,35468 95,70221

249,98566 99,51457 249,49054 95,70382

250,15646 99,5153 249,68251 95,70486

250,3396 99,51607 249,81982 95,70645

250,4928 99,51685 249,96005 95,70806

250,66415 99,51766 250,14357 95,70992

250,83994 99,51849 250,31256 95,71176

251,01152 99,51933 250,50812 95,71371

251,16508 99,52018 250,66261 95,71572

251,3313 99,52104 250,80547 95,71703

251,50636 99,52189 250,94897 95,71867

251,68552 99,52273 251,08632 95,72021

251,84425 99,52358 251,26877 95,72159

252,00964 99,52447 251,43413 95,72338

252,1777 99,52536 251,57684 95,72475

252,35468 99,52628 251,73895 95,72615

252,51115 99,52721 251,9131 95,72842

252,68671 99,52813 252,06596 95,72944

252,8508 99,52903 252,24713 95,73174

253,03711 99,52991 252,42151 95,73275

253,18378 99,53081 252,59108 95,73472

253,35663 99,53168 252,72949 95,73626

253,54016 99,53254 252,8757 95,73804

253,69218 99,53341 253,03896 95,74022

253,86914 99,53428 253,21758 95,74193

254,01587 99,53519 253,38681 95,74372

254,19511 99,53609 253,56384 95,7462

254,3548 99,53698 253,72784 95,74813

254,52667 99,53788 253,89592 95,74984

254,69899 99,53877 254,05768 95,75153

254,84413 99,53969 254,25824 95,75425

254,99818 99,54062 254,42946 95,75618

255,19208 99,54155 254,60327 95,75837

255,34998 99,54249 254,76349 95,76063

255,52602 99,54344 254,92433 95,76278

255,6935 99,54441 255,07675 95,76514

255,84766 99,54538 255,22421 95,76706

256,01047 99,54636 255,42511 95,769

256,17081 99,54735 255,59448 95,77109

256,31842 99,54834 255,78349 95,77321

256,50516 99,54933 255,9435 95,77484

256,67545 99,5503 256,09799 95,77683

256,83594 99,55129 256,24854 95,77964

257,0011 99,5523 256,4054 95,78168

257,16602 99,5533 256,5787 95,78366

257,32565 99,55431 256,75354 95,78583

257,49268 99,5553 256,92581 95,78738

257,65747 99,55628 257,06937 95,78946

257,83664 99,55728 257,2352 95,79136

258,00751 99,5583 257,37463 95,79345

258,15372 99,55929 257,51675 95,79458

258,33475 99,5603 257,69986 95,79681

258,47446 99,56132 257,86865 95,79857

258,66705 99,56236 258,03989 95,80109

258,8432 99,56339 258,21906 95,80269

258,9978 99,56441 258,40909 95,80459

259,15152 99,56542 258,60034 95,80635

259,32385 99,56643 258,76944 95,80797

259,48669 99,56744 258,93951 95,80956

259,65686 99,56846 259,09332 95,81077

259,82062 99,56951 259,25662 95,81239

260,009 99,57058 259,46063 95,81429

260,17468 99,57167 259,63956 95,81609

260,33508 99,57276 259,82764 95,81791

260,51929 99,57384 259,97104 95,81925

260,66431 99,57492 260,09726 95,82088

260,81735 99,576 260,28412 95,82269

260,98315 99,57711 260,47092 95,82384

261,15009 99,57823 260,66849 95,826

261,3389 99,57937 260,84161 95,8269

261,52698 99,58053 261,0105 95,82864

261,66614 99,58171 261,17471 95,83019

261,83655 99,58289 261,32251 95,83206

261,99634 99,58409 261,47629 95,83386

262,15515 99,58532 261,63885 95,83529

262,33392 99,58655 261,82886 95,83651

262,50336 99,58777 262,01178 95,83782

262,67838 99,58899 262,1597 95,83948

262,85477 99,59023 262,33914 95,8411

263,00967 99,59148 262,52264 95,84317

263,16284 99,59276 262,6691 95,84499

263,33405 99,59402 262,81082 95,84638

263,49347 99,59527 262,97214 95,84785

263,65454 99,59655 263,13657 95,84913

263,82571 99,59782 263,25284 95,85088

264,00912 99,59911 263,41833 95,85225

264,16028 99,60039 263,59183 95,85373

264,30646 99,60168 263,79141 95,85526

264,47473 99,60299 263,94351 95,85668

264,65857 99,6043 264,10028 95,85832

264,82071 99,60562 264,24841 95,85986

264,98294 99,60694 264,41928 95,86126

265,13913 99,60829 264,56754 95,86258

265,31876 99,60964 264,73172 95,8636

265,48462 99,61101 264,89365 95,86541

265,67038 99,61239 265,07739 95,86707

265,83514 99,61377 265,21252 95,86875

265,98523 99,61518 265,40076 95,87081

266,15927 99,61661 265,59717 95,87259

266,32715 99,61802 265,73792 95,87431

266,47742 99,61944 265,89795 95,8761

266,68536 99,62087 266,04724 95,87847

266,82803 99,62231 266,20749 95,88009

266,99829 99,62373 266,37332 95,88195

267,1619 99,62516 266,51828 95,88355

267,3252 99,62657 266,69827 95,8853

267,48859 99,628 266,86594 95,88679

267,66 99,62944 267,05005 95,88904

267,83328 99,63089 267,22717 95,89124

267,99258 99,63236 267,40482 95,89311

268,1774 99,63383 267,56207 95,89515

268,34543 99,63528 267,7337 95,89702

268,53278 99,63671 267,90118 95,89868

268,71008 99,63815 268,06622 95,90045

268,86377 99,63958 268,22333 95,90245

269,03723 99,64102 268,41135 95,90511

269,19971 99,64248 268,58487 95,90739

269,36115 99,64394 268,77057 95,90936

269,53986 99,64539 268,93018 95,91154

269,72302 99,64685 269,10413 95,91302

269,90283 99,64831 269,26636 95,91485

270,05493 99,64976 269,4679 95,91678

270,20447 99,6512 269,63138 95,91907

270,37537 99,65266 269,82666 95,92105

270,53561 99,65412 270,00485 95,92282

270,69781 99,65558 270,16293 95,92463

270,85547 99,65704 270,30075 95,92706

271,02844 99,6585 270,4567 95,92938

271,20258 99,65996 270,62064 95,93105

271,38626 99,66142 270,80176 95,93252

271,55627 99,6629 270,97556 95,9342

271,72681 99,66438 271,14041 95,93616

271,87534 99,66586 271,30331 95,93757

272,03412 99,66736 271,49408 95,93963

272,22314 99,66885 271,68295 95,94189

272,39081 99,67034 271,85434 95,94351

272,57727 99,67183 272,0686 95,94548

272,74643 99,67335 272,21667 95,94703

272,8992 99,67486 272,38626 95,94897

273,06268 99,67638 272,55505 95,95108

273,23154 99,6779 272,7319 95,95283

273,42145 99,67943 272,87415 95,95459

273,56714 99,68097 273,05383 95,95688

273,73309 99,68252 273,22223 95,95874

273,9136 99,68405 273,36853 95,96045

274,06964 99,6856 273,53662 95,96229

274,23535 99,68715 273,71671 95,96476

274,40112 99,68869 273,91431 95,96669

274,57285 99,69021 274,08438 95,96841

274,72449 99,69174 274,24716 95,97003

274,89929 99,69329 274,43558 95,97179

275,05191 99,69483 274,61075 95,97379

275,21143 99,69638 274,75024 95,97539

275,39755 99,69792 274,90491 95,97726

275,54987 99,69948 275,08786 95,97904

275,71509 99,70104 275,25818 95,98081

275,86346 99,70262 275,41821 95,98248

276,03705 99,70419 275,61716 95,98435

276,19333 99,70577 275,79816 95,98552

276,34879 99,70736 275,96838 95,98663

276,53989 99,70893 276,15326 95,98888

276,70023 99,71051 276,2981 95,99056

276,88913 99,71209 276,45044 95,99237

277,03693 99,71369 276,62006 95,99395

277,22256 99,71531 276,76462 95,99533

277,41379 99,71695 276,92392 95,99698

277,55371 99,71861 277,08426 95,99829

277,68781 99,72027 277,27429 95,99952

277,88098 99,72191 277,43933 96,00115

278,03787 99,72358 277,58047 96,00287

278,21619 99,72523 277,72342 96,00513

278,37653 99,72688 277,88254 96,00728

278,54221 99,72856 278,05084 96,00902

278,69653 99,73024 278,22903 96,01074

278,87497 99,73194 278,41483 96,0126

279,03925 99,73363 278,57126 96,01461

279,198 99,73533 278,72864 96,01648

279,38 99,73703 278,8862 96,01826

279,54221 99,73875 279,01849 96,01983

279,71704 99,74046 279,18222 96,02105

279,86475 99,74219 279,35367 96,02321

280,05078 99,74389 279,52271 96,02507

280,21497 99,74558 279,66162 96,02664

280,3912 99,74728 279,83087 96,02888

280,57153 99,74896 279,98456 96,03065

280,7139 99,75065 280,15384 96,03235

280,87503 99,75234 280,34009 96,03411

281,04755 99,75402 280,49667 96,03596

281,18747 99,7557 280,6423 96,03812

281,38727 99,75736 280,81308 96,04031

281,56833 99,75902 280,99231 96,04184

281,72525 99,76067 281,14966 96,04381

281,88043 99,76229 281,32996 96,04604

282,02719 99,76389 281,48685 96,04758

282,22653 99,7655 281,64795 96,04952

282,38611 99,76711 281,8334 96,05187

282,54565 99,76871 281,99991 96,05345

282,74155 99,77029 282,1535 96,05462

282,8974 99,77191 282,28036 96,05657

283,05212 99,77354 282,43823 96,05857

283,22803 99,77517 282,63211 96,0605

283,39478 99,77679 282,77274 96,06257

283,57745 99,77842 282,90808 96,06499

283,72861 99,78005 283,07755 96,06702

283,89554 99,78167 283,24487 96,06874

284,05966 99,78331 283,39905 96,07086

284,24884 99,78495 283,57874 96,07251

284,42456 99,7866 283,77182 96,07483

284,58459 99,78825 283,94214 96,07657

284,75516 99,78989 284,11719 96,07834

284,93799 99,79151 284,29733 96,08024

285,08679 99,79315 284,46964 96,08164

285,24661 99,7948 284,62671 96,08426

285,3981 99,79645 284,79559 96,08629

285,60876 99,79808 284,95508 96,08833

285,77161 99,79972 285,1283 96,09088

285,94177 99,80132 285,30914 96,0926

286,13602 99,80292 285,48615 96,09457

286,30811 99,80449 285,64725 96,09681

286,47681 99,80608 285,82776 96,09893

286,6423 99,80766 285,97934 96,10049

286,79193 99,80925 286,16641 96,10152

286,96945 99,81085 286,34671 96,10303

287,14587 99,81245 286,51495 96,10529

287,31204 99,81404 286,6929 96,10662

287,50729 99,81561 286,88748 96,10833

287,67914 99,8172 287,0408 96,10979

287,81967 99,81878 287,20688 96,1118

288,00858 99,82039 287,37442 96,1134

288,16724 99,82199 287,56165 96,11529

288,32959 99,82361 287,72455 96,11743

288,50247 99,82522 287,88043 96,1191

288,67545 99,82685 288,08459 96,12055

288,83047 99,82849 288,26346 96,12236

288,99115 99,83012 288,4314 96,12392

289,16379 99,83177 288,5881 96,12565

289,31772 99,83342 288,79944 96,12736

289,51056 99,83506 288,96524 96,12942

289,66885 99,83673 289,10333 96,13128

289,82288 99,83838 289,2688 96,13296

289,97922 99,84005 289,42596 96,13438

290,13672 99,84171 289,60312 96,13613

290,29443 99,84338 289,76697 96,13769

290,47104 99,84505 289,90979 96,13857

290,64236 99,84673 290,08987 96,14047

290,78455 99,84842 290,27155 96,14183

290,97446 99,85009 290,43082 96,14316

291,14114 99,85177 290,58215 96,14494

291,28613 99,85344 290,7439 96,14664

291,45859 99,85508 290,93579 96,14758

291,61313 99,85671 291,13409 96,14882

291,77991 99,85834 291,31137 96,15027

291,93829 99,85996 291,46213 96,15208

292,078 99,86157 291,61649 96,15333

292,24872 99,86318 291,76828 96,15484

292,41803 99,86479 291,93414 96,15645

292,59021 99,86638 292,09818 96,15791

292,74329 99,86798 292,27011 96,15967

292,9176 99,86956 292,45071 96,16124

293,11139 99,87112 292,64609 96,16294

293,28247 99,87268 292,80298 96,16463

293,46475 99,87426 292,96744 96,16641

293,61591 99,87583 293,13391 96,16792

293,77795 99,87738 293,29883 96,16924

293,93643 99,87891 293,46732 96,17081

294,11096 99,88045 293,62915 96,17231

294,28714 99,88196 293,79306 96,17404

294,45959 99,88348 293,95074 96,17644

294,61209 99,88501 294,11426 96,17827

294,77393 99,88655 294,2562 96,18011

294,95215 99,88808 294,45776 96,18207

295,09827 99,8896 294,63144 96,18415

295,27002 99,89111 294,79178 96,18626

295,45706 99,89261 294,96765 96,18817

295,63028 99,89409 295,1344 96,19011

295,79156 99,89555 295,28534 96,19251

295,96802 99,89703 295,43958 96,19447

296,13672 99,89849 295,62903 96,19666

296,31287 99,89993 295,79178 96,19842

296,47803 99,90134 295,92023 96,20061

296,64587 99,90273 296,10843 96,20268

296,82623 99,90411 296,2572 96,20448

296,99475 99,90548 296,44208 96,20662

297,17191 99,90684 296,61325 96,2087

297,33023 99,90819 296,78442 96,2109

297,50244 99,90954 296,95386 96,21271

297,66464 99,91087 297,07788 96,2143

297,80756 99,91221 297,24088 96,21654

297,9567 99,91356 297,40906 96,21859

298,15588 99,91489 297,57019 96,22059

298,31241 99,91623 297,69421 96,22263

298,50912 99,91756 297,8519 96,22468

298,67117 99,91891 298,04184 96,22704

298,83841 99,92025 298,22717 96,2292

298,98184 99,9216 298,383 96,23165

299,14551 99,92291 298,55646 96,23349

299,32205 99,92424 298,70169 96,23634

299,50623 99,92555 298,858 96,23872

299,67197 99,92686 299,00992 96,24094

299,82773 99,92816 299,18155 96,24323

300,00674 99,92947 299,36285 96,24593

300,17316 99,93076 299,56122 96,24809

300,3241 99,93206 299,70364 96,25013

300,47678 99,93335 299,87299 96,25216

300,64758 99,93465 300,06119 96,25388

300,82483 99,93592 300,20447 96,25592

300,98926 99,93719 300,3606 96,25803

301,14319 99,93845 300,53381 96,26026

301,29736 99,93972 300,70203 96,26265

301,4566 99,94098 300,89474 96,26439

301,62256 99,94225 301,0481 96,26641

301,80261 99,94352 301,22952 96,2685

301,95755 99,94478 301,39404 96,27081

302,11829 99,94602 301,56589 96,27326

302,27774 99,94727 301,70465 96,27509

302,44327 99,9485 301,88708 96,27716

302,62704 99,94974 302,04663 96,27955

302,80109 99,95098 302,19061 96,28108

302,93988 99,95221 302,349 96,28293

303,10336 99,95342 302,53897 96,28494

303,29272 99,95462 302,72498 96,28688

303,45981 99,95583 302,89453 96,28814

303,61581 99,95702 303,04379 96,28975

303,77405 99,95821 303,24512 96,29193

303,91785 99,9594 303,40399 96,2939

304,08878 99,96058 303,552 96,29527

304,27173 99,96174 303,71268 96,29699

304,42545 99,96288 303,90234 96,29864

304,60226 99,96402 304,08038 96,30035

304,75378 99,96514 304,27682 96,30206

304,93127 99,96625 304,42432 96,30396

305,07007 99,96737 304,60327 96,306

305,22525 99,96845 304,76196 96,30848

305,40881 99,96952 304,92642 96,31037

305,58984 99,97059 305,09131 96,31216

305,76404 99,97163 305,25064 96,31404

305,93759 99,97265 305,41272 96,31621

306,08368 99,97366 305,6001 96,31802

306,24213 99,97465 305,77469 96,31931

306,41812 99,97563 305,92859 96,32116

306,57608 99,97658 306,10233 96,32273

306,76129 99,97752 306,29126 96,32429

306,91907 99,97845 306,42868 96,32557

307,08557 99,97936 306,58365 96,32683

307,25305 99,98024 306,74918 96,32854

307,43753 99,98111 306,87744 96,33047

307,60205 99,98198 307,06348 96,33213

307,77881 99,98283 307,22839 96,33365

307,92484 99,98367 307,43158 96,33534

308,08408 99,98451 307,58228 96,33715

308,245 99,98535 307,74747 96,33866

308,3963 99,98617 307,91086 96,33998

308,58374 99,98699 308,09348 96,34171

308,77054 99,98778 308,24054 96,34331

308,94421 99,98855 308,40811 96,34536

309,10733 99,9893 308,58502 96,34718

309,27771 99,99002 308,73254 96,34957

309,45682 99,99073 308,92761 96,35155

309,56577 99,99141 309,10449 96,35318

309,73572 99,99208 309,28049 96,35438

309,90335 99,99272 309,44672 96,35618

310,07678 99,99334 309,5863 96,35804

310,27206 99,99393 309,74231 96,36009

310,42575 99,9945 309,92493 96,36143

310,58829 99,99505 310,07043 96,363

310,76602 99,99559 310,20209 96,36499

310,94141 99,99611 310,35748 96,36698

311,11249 99,99661 310,52252 96,3685

311,26828 99,99707 310,69434 96,37064

311,41968 99,99746 310,87964 96,37217

311,61243 99,99785 311,02408 96,37442

311,77496 99,99818 311,17575 96,37646

311,92432 99,99847 311,33136 96,37844

312,07913 99,99873 311,51529 96,38037

312,26324 99,99896 311,67288 96,38244

312,4158 99,99915 311,88312 96,38461

312,58173 99,99933 312,06702 96,38696

312,72913 99,99948 312,2529 96,38865

312,91846 99,99959 312,38293 96,39084

313,08719 99,99967 312,56744 96,39312

313,2486 99,99971 312,72501 96,39555

313,40811 99,99972 312,92386 96,39767

313,56317 99,99968 313,0896 96,39922

313,75076 99,99961 313,24869 96,40073

313,92731 99,9995 313,37836 96,4028

314,0946 99,99935 313,55783 96,4048

314,22543 99,99916 313,71143 96,40646

314,37762 99,99895 313,89352 96,4083

314,58096 99,99867 314,07309 96,41059

314,76456 99,99832 314,25223 96,41287

314,9187 99,99791 314,41498 96,41441

315,08252 99,99743 314,57605 96,41643

315,25403 99,9969 314,72562 96,41843

315,41901 99,99631 314,87039 96,42105

315,58508 99,99564 315,04446 96,42292

315,76157 99,99491 315,21732 96,42483

315,91669 99,99411 315,37158 96,4268

316,08524 99,99324 315,55988 96,42932

316,28085 99,9923 315,73853 96,43099

316,43616 99,99129 315,89136 96,43257

316,62057 99,99018 316,03003 96,43441

316,79123 99,98899 316,19104 96,43647

316,96109 99,98773 316,36267 96,43849

317,12244 99,9864 316,55472 96,4408

317,28281 99,98501 316,71585 96,44285

317,46362 99,98353 316,87045 96,44419

317,63843 99,98197 317,04895 96,44601

317,80966 99,98034 317,2218 96,44757

318,00391 99,97862 317,39517 96,44929

318,14401 99,97682 317,539 96,45139

318,28879 99,97491 317,71265 96,45363

318,46869 99,97291 317,88019 96,45574

318,6554 99,9708 318,04321 96,45767

318,80933 99,96857 318,23093 96,4596

318,96945 99,96623 318,37744 96,46166

319,16302 99,96377 318,5405 96,46417

319,35181 99,96122 318,70828 96,4658

319,51923 99,95855 318,86682 96,4674

319,66055 99,95578 319,02216 96,46915

319,84024 99,95291 319,1828 96,47062

320,0127 99,94994 319,371 96,47204

320,18652 99,94687 319,54565 96,47358

320,34412 99,94367 319,69528 96,47516

320,52997 99,94035 319,8562 96,47691

320,7121 99,93692 320,00583 96,47873

320,84909 99,93335 320,20255 96,48036

321,02161 99,92967 320,38004 96,48171

321,20596 99,92582 320,54251 96,48335

321,36081 99,92185 320,66882 96,48553

321,53174 99,91773 320,83398 96,48705

321,73816 99,91347 321,01822 96,48821

321,89951 99,90907 321,17673 96,48955

322,04688 99,90452 321,34857 96,49127

322,21008 99,89982 321,51367 96,49282

322,37793 99,89497 321,69025 96,49507

322,53268 99,88993 321,84256 96,49637

322,70306 99,88473 322,02942 96,49787

322,87311 99,87934 322,19955 96,49974

323,03329 99,87376 322,36057 96,5014

323,18939 99,86801 322,51654 96,50371

323,37051 99,86208 322,70865 96,50537

323,50488 99,85599 322,90536 96,5074

323,66818 99,84969 323,03796 96,50917

323,83194 99,84318 323,21771 96,51095

323,99829 99,83646 323,37912 96,5121

324,14804 99,82954 323,57486 96,51416

324,32401 99,82241 323,73312 96,51559

324,48877 99,81505 323,91528 96,51734

324,63098 99,80743 324,07596 96,51865

324,81641 99,79958 324,22278 96,52014

324,98376 99,7915 324,40598 96,52171

325,16187 99,78318 324,56653 96,52366

325,34641 99,77459 324,72742 96,52555

325,50101 99,76574 324,90582 96,52744

325,64929 99,75663 325,07541 96,52912

325,81821 99,74721 325,26038 96,53091

325,96021 99,73753 325,39914 96,53264

326,14651 99,72756 325,56046 96,53441

326,31311 99,71729 325,75668 96,53622

326,48199 99,70671 325,89801 96,53785

326,63318 99,69584 326,05569 96,53922

326,80847 99,68465 326,22986 96,54049

326,95349 99,67315 326,392 96,54202

327,11896 99,66134 326,5759 96,544

327,30298 99,64922 326,72681 96,54548

327,46484 99,63678 326,89163 96,54741

327,63745 99,624 327,04248 96,54821

327,80621 99,61088 327,21661 96,55021

327,98022 99,59741 327,39142 96,55166

328,13873 99,58357 327,56436 96,55343

328,31165 99,56938 327,71863 96,55539

328,47824 99,55481 327,89859 96,55742

328,63754 99,53987 328,07013 96,55881

328,7959 99,52457 328,21741 96,55969

328,97662 99,50889 328,39429 96,56137

329,16034 99,49281 328,58139 96,56302

329,33179 99,47633 328,7439 96,56457

329,50122 99,45945 328,87732 96,56591

329,65308 99,44217 329,05881 96,56734

329,8175 99,42448 329,20654 96,56821

329,95621 99,40637 329,3522 96,56974

330,16284 99,38786 329,5249 96,57176

330,31982 99,36891 329,68741 96,57283

330,49826 99,34954 329,85583 96,57363

330,66425 99,32971 330,01361 96,57508

330,82852 99,30947 330,20383 96,57606

331,0177 99,2888 330,35797 96,57754

331,19537 99,26769 330,50198 96,57887

331,36868 99,24617 330,69302 96,57993

331,53638 99,2242 330,84601 96,58122

331,69647 99,2018 331,02216 96,58162

331,88849 99,17891 331,19113 96,58296

332,0376 99,15557 331,35544 96,58344

332,17825 99,13177 331,52783 96,58464

332,3425 99,10753 331,70569 96,58557

332,52222 99,08284 331,87796 96,58703

332,66391 99,05772 332,05753 96,5878

332,85104 99,03212 332,20325 96,58894

333,0047 99,0061 332,39322 96,58958

333,16 98,97965 332,58765 96,59056

333,31821 98,95275 332,73285 96,59145

333,47256 98,92542 332,92685 96,59264

333,64783 98,89766 333,0983 96,59332

333,82022 98,86947 333,26627 96,5938

333,98358 98,84086 333,4252 96,59426

334,16409 98,81181 333,60229 96,59447

334,30725 98,78237 333,7854 96,59477

334,48019 98,75253 333,94901 96,59517

334,63776 98,72231 334,12631 96,59511

334,80286 98,69169 334,3035 96,59471

334,96542 98,66073 334,46478 96,59502

335,12399 98,62939 334,62418 96,59508

335,28259 98,59769 334,78503 96,59561

335,44162 98,56567 334,93277 96,59558

335,61255 98,53332 335,1134 96,59496

335,80423 98,50066 335,27347 96,59468

335,97754 98,46769 335,46423 96,59413

336,14227 98,43444 335,61829 96,59436

336,27692 98,40093 335,77444 96,59391

336,44128 98,36713 335,95447 96,59368

336,61472 98,3331 336,12585 96,59308

336,77567 98,29884 336,28668 96,59229

336,93289 98,26437 336,44092 96,59148

337,09991 98,22971 336,58972 96,59067

337,25735 98,19486 336,76941 96,59021

337,44678 98,15987 336,92731 96,589

337,61447 98,12476 337,10132 96,58777

337,77112 98,08953 337,26123 96,58637

337,93872 98,05422 337,44516 96,58523

338,12207 98,01885 337,63571 96,58318

338,29562 97,98342 337,77795 96,58174

338,45044 97,94794 337,94019 96,58013

338,65295 97,91245 338,12448 96,57814

338,80103 97,87698 338,27393 96,57583

338,96362 97,84155 338,4447 96,57373

339,13669 97,80621 338,58847 96,57145

339,3262 97,77096 338,74442 96,56882

339,51675 97,73581 338,9136 96,56577

339,69543 97,7008 339,06943 96,56286

339,86179 97,66594 339,23074 96,55944

340,02185 97,63126 339,39343 96,55624

340,20016 97,59679 339,54132 96,55295

340,37256 97,56255 339,68494 96,54923

340,51993 97,52857 339,85468 96,5461

340,7146 97,49487 340,04877 96,54163

340,8931 97,46146 340,18954 96,53734

341,06165 97,42838 340,353 96,53287

341,22998 97,39562 340,50992 96,5283

341,38223 97,36324 340,67065 96,52346

341,56482 97,33122 340,84448 96,51837

341,71652 97,2996 341,04218 96,51304

341,87747 97,26842 341,19128 96,50747

342,05713 97,23766 341,34305 96,50144

342,20593 97,20734 341,52127 96,49454

342,36493 97,1775 341,68066 96,48765

342,5188 97,14817 341,85263 96,48077

342,68738 97,11931 342,03989 96,47343

342,86438 97,091 342,19684 96,46508

343,03793 97,06318 342,35767 96,45626

343,2056 97,03591 342,51379 96,44744

343,35275 97,00917 342,6785 96,43829

343,5199 96,98299 342,83795 96,42766

343,69351 96,95737 343,01392 96,41658

343,85812 96,93233 343,16895 96,405

344,02811 96,90784 343,35233 96,39291

344,19794 96,88393 343,52792 96,37976

344,33643 96,8606 343,6817 96,36608

344,51138 96,83784 343,84058 96,35126

344,65433 96,81565 344,03485 96,3351

344,82382 96,79404 344,21362 96,31817

344,9769 96,77305 344,38748 96,30061

345,13864 96,75263 344,53897 96,28213

345,2883 96,73278 344,70923 96,26261

345,46722 96,71353 344,87213 96,24226

345,63406 96,69485 345,0369 96,22065

345,78943 96,67675 345,18668 96,19778

345,95706 96,65918 345,3902 96,17471

346,10013 96,64215 345,56006 96,14984

346,27939 96,62567 345,70789 96,12386

346,42987 96,60973 345,87268 96,09677

346,58771 96,59432 346,05811 96,06866

346,72534 96,57945 346,2417 96,03913

346,86401 96,5651 346,4006 96,00909

347,03394 96,55125 346,59195 95,97764

347,23611 96,5379 346,77493 95,94535

347,39035 96,52502 346,96277 95,91202

347,57507 96,51258 347,1712 95,87728

347,74423 96,50059 347,37234 95,84187

347,90002 96,48903 347,55014 95,80587

348,0697 96,4779 347,71823 95,76846

348,21243 96,46715 347,88873 95,73124

348,38257 96,45679 348,0459 95,69268

348,56357 96,44681 348,18329 95,6536

348,74353 96,43724 348,34296 95,61369

348,90118 96,42802 348,52783 95,57288

349,07007 96,41914 348,70013 95,53196

349,24609 96,41059 348,86255 95,49038

349,42804 96,40234 349,02521 95,44868

349,5802 96,39438 349,19022 95,40684

349,75967 96,38672 349,35992 95,36445

349,93655 96,37932 349,50354 95,3221

350,10339 96,37217 349,65811 95,27962

350,24475 96,36528 349,83319 95,2375

350,42017 96,35861 349,99863 95,19474

350,56067 96,35217 350,16525 95,15257

350,7388 96,34593 350,32947 95,10997

350,92041 96,33988 350,47433 95,06726

351,09302 96,33401 350,62671 95,02523

351,25592 96,32829 350,79749 94,98408

351,42337 96,32272 350,97571 94,94324

351,58066 96,31729 351,13818 94,90253

351,76233 96,31199 351,32193 94,86276

351,94122 96,30679 351,48343 94,8231

352,11078 96,30168 351,66547 94,78371

352,27441 96,29667 351,7915 94,74492

352,44693 96,29176 351,9552 94,70671

352,60361 96,28693 352,14111 94,66955

352,8009 96,28216 352,32697 94,6325

352,95215 96,27745 352,49854 94,59683

353,12521 96,27282 352,64648 94,56153

353,27808 96,26823 352,82556 94,52753

353,44827 96,26368 353,00024 94,49468

353,63568 96,25916 353,16364 94,46299

353,80362 96,25469 353,33557 94,43196

353,95587 96,25024 353,53198 94,40181

354,11774 96,24581 353,68195 94,37302

354,31299 96,24139 353,8465 94,34522

354,45737 96,23697 354,01013 94,31844

354,63193 96,23257 354,16333 94,29319

354,82013 96,22815 354,31683 94,2689

354,99796 96,22373 354,45908 94,24601

355,15387 96,21929 354,62939 94,22419

355,33652 96,21483 354,77164 94,20345

355,50818 96,21034 354,92395 94,18347

355,68674 96,20584 355,09985 94,16488

355,85596 96,20131 355,28647 94,14739

356,0372 96,19674 355,47113 94,13114

356,19208 96,19216 355,63196 94,11596

356,35468 96,18753 355,78796 94,10168

356,5191 96,18286 355,97238 94,08866

356,67285 96,17815 356,14612 94,07677

356,84998 96,17344 356,2999 94,06519

357,05801 96,16869 356,4668 94,05419

357,19687 96,16391 356,63943 94,04425

357,35852 96,15905 356,80579 94,03523

357,54517 96,15414 356,98239 94,02705

357,70618 96,14918 357,13602 94,01964

357,89758 96,14418 357,319 94,01308

358,03302 96,13913 357,47998 94,00678

358,2428 96,13406 357,63031 94,00068

358,39438 96,12892 357,78452 93,99534

358,56372 96,12372 357,94119 93,99038

358,74362 96,11848 358,09863 93,98575

358,90359 96,11315 358,29477 93,98132

359,06882 96,10776 358,47101 93,9771

359,23053 96,10231 358,62967 93,9727

359,41132 96,0968 358,80701 93,96965

359,5719 96,09123 358,97473 93,96648

359,7222 96,0856 359,10794 93,96311

359,87527 96,07992 359,30603 93,9598

360,05627 96,07419 359,47308 93,95698

360,19995 96,06839 359,63715 93,95384

360,37308 96,06254 359,80591 93,95023

360,54349 96,05665 359,9794 93,94806

360,68259 96,05071 360,15179 93,94589

360,85074 96,04471 360,31323 93,94386

360,99646 96,03865 360,47925 93,94184

361,16296 96,03252 360,65491 93,93988

361,33643 96,02634 360,83673 93,9376

361,48505 96,0201 360,98914 93,93556

361,64407 96,01381 361,14417 93,93399

361,79324 96,00745 361,30225 93,93198

361,94519 96,00103 361,45242 93,92973

362,12 95,99456 361,62378 93,92721

362,27216 95,98799 361,80054 93,92488

362,43539 95,98138 361,9613 93,92264

362,61774 95,9747 362,11426 93,91992

362,76331 95,96796 362,28421 93,91795

362,92346 95,96116 362,44678 93,91535

363,08362 95,95432 362,60086 93,91298

363,24329 95,9474 362,77545 93,9107

363,41852 95,94044 362,9176 93,90841

363,56039 95,9334 363,08188 93,90589

363,71689 95,92627 363,2543 93,90335

363,91238 95,91908 363,42612 93,90074

364,08008 95,91181 363,59644 93,89779

364,25818 95,90449 363,77045 93,89548

364,41953 95,89712 363,9559 93,89284

364,56268 95,88966 364,13947 93,8901

364,73163 95,88212 364,30859 93,88714

364,91614 95,8745 364,48508 93,88426

365,08337 95,8668 364,64737 93,8819

365,2298 95,85899 364,79156 93,87882

365,39783 95,8511 364,9812 93,87612

365,58594 95,84314 365,11954 93,87315

365,74783 95,83509 365,27713 93,87013

365,90717 95,82694 365,4386 93,86715

366,0896 95,81871 365,61676 93,86448

366,2623 95,81039 365,7843 93,86121

366,42981 95,80196 365,93188 93,85761

366,60046 95,79343 366,10129 93,85429

366,77454 95,78481 366,31042 93,85083

366,94641 95,77609 366,47754 93,84716

367,12366 95,76729 366,61218 93,84333

367,30884 95,7584 366,77441 93,83947

367,4823 95,74944 366,95755 93,83584

367,64102 95,74036 367,09863 93,83252

367,81039 95,73119 367,25909 93,82815

367,96878 95,72193 367,4082 93,82438

368,1759 95,71255 367,59924 93,82041

368,35526 95,70304 367,75989 93,81682

368,51831 95,69343 367,93564 93,81262

368,69678 95,68372 368,12262 93,80887

368,86603 95,6739 368,31326 93,80443

369,03806 95,66395 368,4856 93,79988

369,19846 95,65387 368,67529 93,79526

369,37573 95,64371 368,82108 93,78992

369,5517 95,63342 368,96902 93,7844

369,7164 95,62304 369,13446 93,77883

369,90558 95,61253 369,30664 93,77403

370,07144 95,60191 369,46472 93,76869

370,23959 95,59115 369,66568 93,76367

370,39096 95,58028 369,8511 93,7585

370,55533 95,56927 369,99146 93,7525

370,70789 95,55812 370,12027 93,74718

370,88281 95,54685 370,27856 93,74163

371,05228 95,53543 370,45444 93,73609

371,21799 95,52387 370,61063 93,73105

371,3674 95,51217 370,75488 93,72538

371,52121 95,50033 370,94199 93,71912

371,69077 95,48836 371,11404 93,71311

371,83759 95,47625 371,28781 93,70691

371,98813 95,464 371,44116 93,70075

372,18286 95,45163 371,60315 93,69466

372,3208 95,43915 371,74646 93,68821

372,47552 95,42655 371,90491 93,68168

372,633 95,41382 372,08255 93,67546

372,79449 95,40092 372,26447 93,66926

372,96735 95,38788 372,43774 93,66281

373,12201 95,37472 372,59967 93,65611

373,28479 95,36141 372,77237 93,65002

373,4201 95,34801 372,90616 93,64317

373,58347 95,33449 373,09274 93,63589

373,75812 95,32084 373,27185 93,62919

373,93463 95,30706 373,43588 93,62249

374,07098 95,29314 373,62152 93,61518

374,26288 95,27909 373,77014 93,60807

374,44003 95,26492 373,92746 93,60081

374,58334 95,25061 374,07483 93,59284

374,77271 95,23617 374,23047 93,58525

374,91702 95,22159 374,41943 93,57764

375,08939 95,20688 374,58289 93,56961

375,25961 95,19203 374,76563 93,56188

375,42102 95,17705 374,96216 93,55401

375,58682 95,16192 375,1076 93,5458

375,76074 95,14665 375,27469 93,53765

375,92542 95,13124 375,43594 93,52921

376,09671 95,11569 375,58719 93,5209

376,25851 95,1 375,75812 93,51226

376,41989 95,08415 375,92853 93,50326

376,59567 95,06819 376,10608 93,4945

376,76651 95,05209 376,27469 93,4852

376,92294 95,03584 376,42123 93,47611

377,10715 95,01946 376,61075 93,46647

377,29767 95,00293 376,78003 93,45752

377,46985 94,98625 376,95615 93,44759

377,63708 94,96941 377,11188 93,43815

377,79654 94,95241 377,29373 93,42832

377,98663 94,93527 377,41748 93,41887

378,16141 94,91795 377,59192 93,40965

378,32886 94,90049 377,76083 93,39982

378,51804 94,88287 377,96692 93,38993

378,68069 94,86511 378,13931 93,37949

378,85596 94,84718 378,29916 93,36897

379,01135 94,82912 378,46646 93,35958

379,20078 94,81093 378,62219 93,34905

379,36011 94,79259 378,76785 93,33781

379,54242 94,77412 378,94879 93,32654

379,72659 94,75548 379,07416 93,31505

379,90115 94,73672 379,26151 93,30354

380,06696 94,71779 379,39038 93,29255

380,23108 94,69873 379,56555 93,28056

380,39325 94,6795 379,77267 93,26894

380,56201 94,66011 379,98584 93,25707

380,7265 94,64056 380,16888 93,24536

380,90204 94,62085 380,35168 93,23388

381,05188 94,60094 380,52319 93,22206

381,21771 94,58089 380,66159 93,20981

381,39645 94,56068 380,84586 93,19771

381,5549 94,54031 381,02402 93,18528

381,71979 94,51978 381,2117 93,17293

381,8855 94,49907 381,35352 93,15991

382,0325 94,47819 381,53018 93,14667

382,21863 94,45712 381,68808 93,1336

382,38159 94,43589 381,86572 93,11998

382,54242 94,41448 382,04953 93,10632

382,6991 94,39289 382,21487 93,09303

382,88416 94,37113 382,38409 93,07953

383,07739 94,34919 382,51434 93,06598

383,23434 94,32706 382,67825 93,05209

383,40762 94,30476 382,83307 93,03821

383,57254 94,28231 382,98462 93,02437

383,74957 94,25965 383,17081 93,01041

383,90283 94,23681 383,32111 92,99572

384,08136 94,21379 383,4696 92,98116

384,24365 94,19058 383,65036 92,96651

384,42517 94,16718 383,82739 92,95124

384,58682 94,14359 383,98853 92,93599

384,73755 94,1198 384,14673 92,92046

384,91238 94,09582 384,31616 92,90543

385,08203 94,07164 384,48987 92,89024

385,26471 94,04727 384,67325 92,87473

385,44217 94,02268 384,83737 92,85891

385,61673 93,99791 384,98132 92,84286

385,77042 93,97294 385,17355 92,82745

385,93579 93,94777 385,332 92,81113

386,07477 93,92241 385,48477 92,79495

386,23233 93,8969 385,62378 92,77869

386,39395 93,87119 385,78152 92,76249

386,56915 93,8453 385,9718 92,7457

386,73883 93,81921 386,1398 92,7286

386,90588 93,79294 386,29419 92,71184

387,07068 93,76647 386,45093 92,69472

387,25815 93,7398 386,62329 92,67774

387,40186 93,71293 386,77914 92,66065

387,58783 93,68586 386,93683 92,64307

387,74527 93,65858 387,08838 92,62539

387,91882 93,63109 387,27844 92,60826

388,09131 93,6034 387,44366 92,59084

388,23743 93,5755 387,61435 92,57321

388,41931 93,54738 387,80679 92,5556

388,56052 93,51906 387,98111 92,53782

388,73715 93,49054 388,1554 92,51988

388,90588 93,46181 388,30118 92,50208

389,06165 93,43286 388,46564 92,48368

389,24316 93,40371 388,63983 92,46479

389,41626 93,37435 388,82059 92,44574

389,58447 93,34479 388,98959 92,42674

389,74338 93,31502 389,1629 92,40795

389,92517 93,28502 389,29733 92,38869

390,0972 93,25481 389,461 92,36922

390,25073 93,22439 389,65103 92,35031

390,42227 93,19376 389,82797 92,33084

390,60229 93,16291 389,99689 92,31136

390,74429 93,13183 390,16873 92,29196

390,9111 93,10056 390,36285 92,27217

391,06543 93,06904 390,5401 92,25211

391,24295 93,03732 390,71503 92,23214

391,42975 93,00538 390,88745 92,21156

391,5928 92,97325 391,0242 92,19094

391,76511 92,94089 391,19666 92,17025

391,90262 92,90831 391,37573 92,14964

392,04343 92,8755 391,53891 92,12859

392,19812 92,84248 391,71255 92,10744

392,37189 92,80924 391,86646 92,08628

392,54602 92,77579 392,01709 92,06525

392,6832 92,7421 392,2121 92,04421

392,83414 92,70822 392,3866 92,0229

392,97537 92,67409 392,51483 92,00186

393,10852 92,63976 392,66129 91,98039

393,25369 92,6052 392,82544 91,95858

393,42361 92,57041 392,99527 91,93635

393,56995 92,53541 393,15723 91,91447

393,72565 92,50017 393,30679 91,89266

393,87845 92,4647 393,47266 91,87018

394,01935 92,42898 393,6626 91,84751

394,1691 92,39304 393,81009 91,82523

394,31766 92,35686 393,96307 91,80283

394,47009 92,32039 394,13074 91,78012

394,62146 92,28367 394,29968 91,75729

394,73755 92,24668 394,42542 91,73446

394,91742 92,20944 394,60059 91,71163

395,05957 92,17193 394,75513 91,68839

395,20206 92,13414 394,93222 91,6651

395,3623 92,09609 395,11414 91,64146

395,51187 92,05779 395,25235 91,61772

395,66443 92,01921 395,39944 91,59347

395,83017 91,98035 395,57214 91,56966

395,99863 91,94122 395,72498 91,54561

396,15823 91,9018 395,90747 91,52129

396,30487 91,8621 396,07764 91,4971

396,43991 91,82213 396,22137 91,47232

396,62793 91,78184 396,39029 91,44766

396,79074 91,74126 396,54916 91,42295

396,95609 91,70036 396,70154 91,3974

397,12451 91,65917 396,88013 91,37209

397,28125 91,61765 397,06592 91,34689

397,43866 91,57583 397,21072 91,32179

397,59497 91,53366 397,4007 91,29595

397,78894 91,49117 397,57053 91,26988

397,95819 91,44836 397,74387 91,24361

398,15652 91,40523 397,9111 91,21763

398,34225 91,36175 398,05457 91,19061

398,49573 91,3179 398,26077 91,16388

398,66931 91,2737 398,42896 91,13771

398,84 91,22916 398,61029 91,11063

399,03564 91,18424 398,77798 91,08408

399,2135 91,13897 398,95178 91,0573

399,39764 91,0933 399,10907 91,02933

399,56949 91,04725 399,25424 91,00207

399,74353 91,00077 399,41412 90,97461

399,91211 90,95389 399,57202 90,94737

400,08752 90,9066 399,77786 90,91949

400,28772 90,85889 399,97479 90,89179

400,46213 90,81077 400,14661 90,86386

400,62878 90,76221 400,31503 90,83603

400,79938 90,71321 400,50745 90,8076

400,99414 90,66377 400,68918 90,77866

401,14758 90,61388 400,87311 90,7505

401,31119 90,56353 401,06824 90,72181

401,49838 90,51273 401,24646 90,69262

401,66809 90,46147 401,39438 90,66348

401,83008 90,40971 401,56149 90,6345

402,02478 90,35748 401,74768 90,60493

402,19104 90,30474 401,94998 90,5749

402,37927 90,25153 402,10458 90,54538

402,55264 90,19782 402,29736 90,51568

402,72913 90,14359 402,46518 90,48572

402,89404 90,08885 402,60815 90,45587

403,06445 90,03359 402,77591 90,42535

403,21024 89,97783 402,95609 90,39462

403,39639 89,92151 403,09473 90,36435

403,54385 89,86463 403,2616 90,33374

403,69598 89,80721 403,4505 90,30292

403,86374 89,74921 403,62305 90,27189

404,04642 89,69063 403,80176 90,2409

404,18848 89,63145 403,95459 90,20998

404,36752 89,57167 404,0976 90,17845

404,5119 89,51127 404,28711 90,14676

404,70642 89,45025 404,43054 90,115

404,86185 89,3886 404,59525 90,08294

405,04993 89,3263 404,74542 90,05079

405,22366 89,26336 404,91531 90,01909

405,36923 89,19978 405,09448 89,98626

405,53259 89,13551 405,24396 89,95357

405,70322 89,07057 405,42841 89,92112

405,84805 89,00495 405,59048 89,88867

406,01752 88,93863 405,76047 89,85583

406,19141 88,8716 405,91025 89,82244

406,36862 88,80384 406,06427 89,78974

406,52179 88,73535 406,22739 89,75643

406,68655 88,66611 406,38989 89,72359

406,85663 88,5961 406,54736 89,68973

406,98779 88,5253 406,70193 89,65601

407,16992 88,45375 406,84433 89,62221

407,3298 88,38139 406,99246 89,5879

407,48676 88,3082 407,16711 89,5538

407,64053 88,23419 407,29941 89,5195

407,81146 88,15933 407,46655 89,48455

407,96423 88,08365 407,65103 89,45104

408,13232 88,0071 407,83337 89,41658

408,29779 87,9297 408,01501 89,38155

408,47208 87,85141 408,15686 89,34615

408,62927 87,77223 408,29074 89,31056

408,78406 87,69212 408,44766 89,27466

408,93762 87,61108 408,58182 89,23902

409,10886 87,52908 408,74915 89,20348

409,26828 87,44614 408,91983 89,16735

409,41541 87,36219 409,09216 89,13134

409,59167 87,27723 409,25732 89,09466

409,77722 87,19125 409,4209 89,05821

409,92053 87,10423 409,57629 89,02207

410,07407 87,01613 409,72717 88,98539

410,28485 86,92696 409,91772 88,94817

410,44559 86,8367 410,0802 88,91105

410,5928 86,74534 410,27966 88,87412

410,78452 86,65287 410,43018 88,83639

410,95728 86,55923 410,64389 88,79917

411,13358 86,46444 410,83411 88,76112

411,28796 86,36848 410,94241 88,72266

411,45166 86,27132 411,1268 88,68397

411,63159 86,17296 411,3121 88,64539

411,79178 86,0734 411,46057 88,60652

411,9621 85,97262 411,64139 88,56759

412,10449 85,87061 411,81116 88,52826

412,29007 85,76736 412,00711 88,48862

412,46692 85,66287 412,15375 88,44875

412,63501 85,55712 412,33508 88,40864

412,81894 85,4501 412,48315 88,36842

412,98138 85,34181 412,65369 88,32806

413,17694 85,23222 412,83026 88,28792

413,35107 85,12134 413,01599 88,24763

413,5242 85,00914 413,17288 88,20694

413,69916 84,89563 413,34891 88,16576

413,86948 84,78082 413,51547 88,12514

414,03754 84,66466 413,68628 88,08406

414,2218 84,54718 413,85077 88,04219

414,40088 84,42835 414,01624 87,99994

414,56726 84,30816 414,18103 87,95796

414,72528 84,18662 414,3685 87,91587

414,88364 84,06374 414,54144 87,87323

415,04105 83,93951 414,70868 87,83055

415,22699 83,81395 414,86362 87,78715

415,37402 83,68705 415,02231 87,74379

415,55969 83,55882 415,21362 87,70115

415,72983 83,42924 415,39636 87,65777

415,92044 83,29835 415,55283 87,6138

416,09042 83,16615 415,72977 87,57014

416,27744 83,03263 415,90848 87,52549

416,41211 82,8978 416,07547 87,48105

416,59033 82,76168 416,20178 87,4358

416,74661 82,62425 416,3782 87,39056

416,92377 82,48554 416,54111 87,34515

417,08801 82,34557 416,70984 87,2998

417,271 82,20434 416,87952 87,2543

417,45834 82,06189 417,02344 87,20832

417,629 81,91824 417,1955 87,16175

417,80261 81,77339 417,3598 87,11441

417,97446 81,62738 417,52206 87,06776

418,14453 81,48025 417,68842 87,02105

418,27856 81,33201 417,86948 86,97398

418,43954 81,18268 418,02817 86,92611

418,6145 81,03231 418,19421 86,87802

418,78989 80,88093 418,32654 86,82976

418,93686 80,72856 418,50781 86,78191

419,09872 80,57526 418,68808 86,73368

419,27167 80,42101 418,87524 86,68472

419,42801 80,26587 419,00833 86,63611

419,61469 80,10987 419,15195 86,58625

419,78705 79,95304 419,32092 86,53719

419,93979 79,79543 419,48349 86,48806

420,10004 79,63708 419,62079 86,43813

420,26849 79,47801 419,80237 86,38807

420,43954 79,31829 419,97217 86,33771

420,60156 79,15793 420,16736 86,28697

420,78046 78,99694 420,34647 86,23563

420,94974 78,83539 420,53061 86,18501

421,08911 78,67333 420,69827 86,13392

421,2558 78,51078 420,86664 86,08227

421,44714 78,34781 421,00897 86,03107

421,60657 78,18445 421,18695 85,97909

421,77283 78,02074 421,35965 85,9266

421,90683 77,85672 421,49066 85,87423

422,06747 77,69244 421,66333 85,82104

422,21942 77,52794 421,82904 85,76796

422,38431 77,36326 421,99695 85,71446

422,54404 77,19848 422,1622 85,66102

422,71536 77,03363 422,30063 85,60753

422,89114 76,86876 422,48279 85,55392

423,0697 76,70394 422,64505 85,49964

423,22079 76,5392 422,82547 85,44481

423,3638 76,37458 422,96484 85,39021

423,53378 76,21015 423,13763 85,33539

423,70688 76,04593 423,31357 85,28021

423,87207 75,88196 423,49051 85,22487

424,06848 75,71832 423,64441 85,16867

424,21005 75,55505 423,8121 85,11256

424,3576 75,39219 424,00037 85,0561

424,50897 75,22977 424,15564 84,99878

424,67709 75,06787 424,32678 84,94239

424,85501 74,90651 424,49976 84,88601

425,00696 74,74576 424,62402 84,82841

425,19305 74,58565 424,78519 84,77087

425,38837 74,42623 424,97705 84,71344

425,53085 74,26752 425,15375 84,65546

425,71005 74,1096 425,31873 84,59743

425,87708 73,95247 425,48096 84,53977

426,03979 73,7962 425,64441 84,48176

426,24081 73,64085 425,81311 84,4233

426,40967 73,48643 425,96078 84,36402

426,59305 73,33296 426,08148 84,3047

426,7594 73,18051 426,2583 84,24535

426,90247 73,02909 426,45361 84,18603

427,07571 72,87874 426,62378 84,12672

427,26239 72,72948 426,79443 84,06682

427,44067 72,58135 426,94559 84,00685

427,61429 72,43436 427,10645 83,94608

427,76855 72,28858 427,2843 83,88576

427,93808 72,14404 427,45496 83,82459

428,09988 72,00074 427,64529 83,76367

428,2627 71,85873 427,82089 83,70238

428,41675 71,71799 427,98291 83,64075

428,61755 71,5786 428,10922 83,5798

428,77451 71,44054 428,27844 83,51877

428,93475 71,30384 428,46732 83,45695

429,07996 71,16854 428,63528 83,3949

429,2475 71,03468 428,78217 83,33305

429,39462 70,90225 428,95422 83,27046

429,58359 70,77126 429,12378 83,20845

429,72632 70,64174 429,28735 83,14617

429,90561 70,51367 429,46014 83,08326

430,05716 70,38709 429,60144 83,02038

430,21756 70,26202 429,76108 82,95784

430,38696 70,13846 429,94965 82,89474

430,55765 70,01641 430,13763 82,83209

430,71582 69,8959 430,29114 82,76871

430,87796 69,77691 430,46072 82,70537

431,0513 69,65945 430,64789 82,64174

431,22177 69,54352 430,8186 82,57817

431,38895 69,42912 431,00687 82,51437

431,55396 69,31626 431,19141 82,45076

431,73718 69,20492 431,35257 82,38713

431,88675 69,0951 431,50867 82,32288

432,05908 68,98681 431,69476 82,25899

432,21997 68,88003 431,8616 82,19485

432,38138 68,77476 432,0199 82,13098

432,53802 68,671 432,16711 82,06608

432,69321 68,56871 432,37061 82,00172

432,84232 68,46792 432,53622 81,9379

433,01001 68,3686 432,68631 81,87425

433,16693 68,27074 432,85956 81,80984

433,36133 68,17434 433,0119 81,74489

433,51987 68,07938 433,14212 81,68085

433,69312 67,98585 433,33084 81,61657

433,8511 67,89374 433,5018 81,55242

434,01068 67,80305 433,66956 81,48823

434,16507 67,71376 433,81384 81,42403

434,30835 67,62586 433,97791 81,35929

434,49744 67,53929 434,13721 81,29475

434,67068 67,45407 434,24854 81,23049

434,83032 67,37016 434,44165 81,1661

434,99594 67,28757 434,61606 81,10166

435,15585 67,20628 434,78564 81,03704

435,31824 67,12627 434,93604 80,97293

435,49564 67,04752 435,11322 80,90817

435,68915 66,97001 435,26483 80,84384

435,86121 66,89372 435,43588 80,77933

436,00302 66,81865 435,5892 80,71525

436,16687 66,74474 435,77475 80,65079

436,34872 66,672 435,95154 80,58714

436,5162 66,6004 436,09143 80,52319

436,70062 66,52993 436,22314 80,45918

436,85663 66,46058 436,40701 80,39502

437,01587 66,39231 436,58817 80,33112

437,18954 66,32512 436,74808 80,26713

437,345 66,25898 436,92938 80,20381

437,52283 66,19384 437,10394 80,14067

437,68613 66,12967 437,25549 80,07746

437,83588 66,0665 437,43555 80,01445

438,02371 66,00428 437,57938 79,95102

438,20715 65,94302 437,75076 79,88773

438,3468 65,88267 437,97919 79,82553

438,52429 65,82325 438,12292 79,76299

438,67725 65,7647 438,27881 79,70027

438,86551 65,70703 438,45908 79,63787

439,06531 65,65023 438,62537 79,57537

439,1915 65,59425 438,82886 79,5135

439,38193 65,53908 438,96753 79,45175

439,55432 65,48471 439,12656 79,39021

439,70926 65,43112 439,31894 79,32805

439,85828 65,37828 439,51819 79,26658

440,035 65,32618 439,68051 79,20521

440,19897 65,27483 439,84515 79,14404

440,37122 65,2242 440,02444 79,08279

440,53851 65,17426 440,19174 79,0218

440,69501 65,12502 440,35931 78,96145

440,88046 65,07645 440,52167 78,90087

441,03809 65,02856 440,68607 78,84136

441,22495 64,98133 440,83685 78,78151

441,41171 64,93473 440,9971 78,7218

441,55389 64,88874 441,14944 78,66178

441,70023 64,84337 441,34265 78,60273

441,86337 64,7986 441,51572 78,54423

442,03885 64,75441 441,69006 78,48561

442,19507 64,7108 441,85168 78,42744

442,35965 64,66776 442,0155 78,36875

442,53851 64,62526 442,17572 78,31048

442,68848 64,58327 442,32025 78,25254

442,84662 64,54181 442,50256 78,19473

443,02908 64,50086 442,68066 78,13715

443,20267 64,46041 442,8345 78,07989

443,35873 64,42045 442,98407 78,02306

443,53989 64,38095 443,12576 77,96666

443,70752 64,3419 443,2868 77,91028

443,88773 64,3033 443,46375 77,8541

444,04462 64,26516 443,60947 77,79854

444,21243 64,22745 443,77478 77,74302

444,39746 64,19018 443,96487 77,68774

444,56454 64,15333 444,11914 77,63298

444,73856 64,11687 444,30383 77,57865

444,91693 64,08081 444,46802 77,52411

445,08231 64,04512 444,63037 77,47005

445,2312 64,00982 444,79236 77,41607

445,40771 63,97489 444,95633 77,36268

445,59207 63,94032 445,10971 77,3093

445,75882 63,9061 445,30249 77,25664

445,92004 63,87222 445,48743 77,20401

446,09329 63,83871 445,67416 77,15175

446,25613 63,80556 445,86145 77,10089

446,41937 63,77273 446,03931 77,05013

446,58362 63,74023 446,19965 76,99966

446,72357 63,70803 446,37534 76,9496

446,89163 63,67616 446,56064 76,89928

447,03915 63,64459 446,71356 76,84973

447,22498 63,61332 446,89343 76,8005

447,38428 63,58233 447,06567 76,75157

447,5304 63,5516 447,25443 76,70326

447,72522 63,52117 447,42734 76,65491

447,8866 63,49102 447,60779 76,60713

448,05042 63,46114 447,74646 76,55991

448,22064 63,43155 447,89795 76,51407

448,37332 63,40223 448,06165 76,46818

448,51709 63,37316 448,20752 76,42274

448,68582 63,34434 448,39221 76,37728

448,84766 63,31575 448,5647 76,33258

449,02078 63,28739 448,74252 76,2879

449,20535 63,25927 448,92126 76,24337

449,37576 63,23138 449,08813 76,19966

449,5321 63,20368 449,27454 76,15577

449,71176 63,1762 449,43542 76,11273

449,87918 63,14892 449,5994 76,0696

450,05014 63,12186 449,74677 76,02714

450,19891 63,09499 449,89993 75,9843

450,37225 63,0683 450,0578 75,94224

450,55042 63,04181 450,21902 75,90001

450,70859 63,01549 450,38965 75,85787

450,88086 62,98933 450,59061 75,81605

451,04926 62,96335 450,75879 75,77433

451,2467 62,93753 450,92368 75,73305

451,40125 62,91186 451,11035 75,69159

451,55267 62,88636 451,24335 75,65064

451,73779 62,86099 451,40424 75,60948

451,90179 62,8358 451,56479 75,56876

452,07831 62,81076 451,7312 75,52719

452,24594 62,78589 451,90887 75,48658

452,42664 62,76116 452,05261 75,4453

452,60126 62,73657 452,20825 75,40406

452,74506 62,71209 452,36902 75,36307

452,89554 62,68774 452,54852 75,32147

453,07303 62,6635 452,70551 75,27982

453,24524 62,63942 452,88794 75,23809

453,41873 62,61545 453,07092 75,19609

453,57007 62,59161 453,26117 75,15365

453,73734 62,56786 453,431 75,111

453,89893 62,5442 453,59262 75,06815

454,0596 62,52064 453,77103 75,02514

454,21429 62,49715 453,92044 74,981

454,39984 62,47377 454,08426 74,93638

454,57162 62,45045 454,2301 74,89176

454,73465 62,42721 454,44046 74,84586

454,89331 62,40405 454,59857 74,7995

455,05283 62,38096 454,7803 74,75239

455,21576 62,35794 454,95444 74,7051

455,38696 62,33496 455,08969 74,65708

455,57108 62,31203 455,25635 74,60828

455,73685 62,28916 455,42532 74,55887

455,88525 62,26635 455,5831 74,50851

456,04297 62,2436 455,76019 74,45743

456,20361 62,22089 455,90952 74,40567

456,37817 62,19821 456,04779 74,35267

456,53983 62,17557 456,21179 74,29878

456,71167 62,15293 456,40277 74,24358

456,87161 62,13031 456,58115 74,1871

457,05618 62,10768 456,70984 74,12977

457,21112 62,08507 456,88184 74,07189

457,39838 62,06247 457,06204 74,01295

457,55969 62,03991 457,245 73,95292

457,73529 62,01733 457,40488 73,89228

457,90048 61,99476 457,55908 73,83016

458,06738 61,97219 457,71228 73,76758

458,24036 61,94961 457,84882 73,70329

458,4205 61,92703 458,00293 73,63802

458,56702 61,90442 458,19006 73,57149

458,73309 61,88178 458,34988 73,50396

458,91431 61,85913 458,50775 73,43476

459,10754 61,83644 458,67084 73,36514

459,26801 61,81373 458,86841 73,29372

459,40659 61,79101 459,04208 73,22155

459,58563 61,76827 459,21194 73,14817

459,77539 61,74549 459,37024 73,07352

459,93311 61,72269 459,54224 72,9977

460,1019 61,69984 459,67987 72,92013

460,27094 61,67696 459,86053 72,84212

460,44046 61,65402 460,01028 72,763

460,60852 61,631 460,14145 72,68236

460,78699 61,60791 460,33163 72,60001

460,92676 61,58474 460,52429 72,51653

461,1134 61,56147 460,69324 72,43191

461,27533 61,53812 460,89676 72,34587

461,43588 61,51468 461,04999 72,25912

461,63242 61,49115 461,20584 72,17042

461,8053 61,4675 461,39178 72,08086

461,96576 61,44373 461,52875 71,98981

462,14828 61,41985 461,71512 71,89764

462,30032 61,39585 461,90146 71,80406

462,45273 61,37174 462,07147 71,70926

462,65149 61,34751 462,21216 71,61292

462,83084 61,32316 462,39325 71,51631

463,00806 61,29864 462,56506 71,41779

463,18042 61,27396 462,74023 71,31778

463,35742 61,24913 462,91797 71,2165

463,50931 61,22411 463,1062 71,11403

463,69275 61,19892 463,27158 71,01004

463,84842 61,17356 463,41757 70,90431

464,01703 61,14803 463,57083 70,79724

464,19128 61,12229 463,71289 70,68957

464,36664 61,09634 463,91751 70,58041

464,51663 61,07016 464,07797 70,46968

464,69885 61,04374 464,2431 70,35737

464,84491 61,01708 464,41431 70,24415

464,98969 60,99018 464,56628 70,12942

465,15604 60,96302 464,7229 70,01368

465,32211 60,93562 464,92175 69,89662

465,51688 60,90795 465,08441 69,77839

465,68762 60,88003 465,26273 69,65857

465,83972 60,85184 465,41736 69,53787

465,99808 60,82334 465,56473 69,41563

466,14261 60,7945 465,74661 69,29206

466,32645 60,76535 465,92035 69,16644

466,47626 60,73584 466,08514 69,03961

466,66327 60,70599 466,25037 68,91148

466,8309 60,67579 466,41684 68,78173

466,99594 60,64523 466,56732 68,65045

467,16953 60,61429 466,70715 68,51836

467,32294 60,58298 466,87109 68,3848

467,49634 60,55124 466,99048 68,2493

467,66342 60,51907 467,15173 68,11243

467,82117 60,48648 467,33545 67,97449

467,98822 60,45341 467,51483 67,83518

468,13593 60,41986 467,7095 67,69411

468,29956 60,38584 467,85327 67,55113

468,48694 60,35128 468,02893 67,40693

468,63657 60,31622 468,19632 67,26174

468,80038 60,28064 468,36591 67,11481

468,98001 60,24451 468,53979 66,96531

469,14145 60,20783 468,69287 66,8157

469,31219 60,17057 468,88535 66,66374

469,46185 60,13274 469,01575 66,51047

469,61942 60,09431 469,17245 66,35596

469,78275 60,05527 469,3616 66,19979

469,94061 60,01561 469,53247 66,04178

470,12283 59,97531 469,68323 65,88231

470,27689 59,93436 469,86926 65,72034

470,45193 59,89275 470,03711 65,55748

470,62061 59,85046 470,21936 65,39258

470,78226 59,80747 470,40057 65,22619

470,95755 59,76378 470,54535 65,05761

471,13217 59,71934 470,69623 64,88776

471,28821 59,67414 470,85257 64,71547

471,47235 59,62819 471,0242 64,54181

471,64771 59,58148 471,19125 64,36575

471,82523 59,53395 471,32617 64,18781

471,98596 59,48563 471,52271 64,00804

472,1452 59,43648 471,66385 63,82661

472,32773 59,38647 471,8179 63,64296

472,49442 59,33559 472,01758 63,45732

472,6701 59,28384 472,20532 63,26945

472,87225 59,23121 472,35858 63,07943

473,03882 59,17767 472,5238 62,88793

473,21167 59,12321 472,70691 62,69372

473,39911 59,0678 472,88861 62,4973

473,59198 59,01144 473,0697 62,29915

473,75272 58,95407 473,22446 62,09812

473,93539 58,89568 473,37411 61,89472

474,09109 58,83628 473,54633 61,68863

474,26859 58,77582 473,72464 61,48009

474,46423 58,71429 473,88513 61,26837

474,6098 58,65169 474,0372 61,05431

474,77405 58,58799 474,2168 60,83277

474,96118 58,52319 474,35654 60,58304

475,13171 58,45728 474,57336 60,29227

475,29138 58,39022 474,74561 59,95328

475,46112 58,32198 474,89001 59,55972

475,61084 58,25254 475,0639 59,12171

475,77069 58,18189 475,2291 58,64469

475,94507 58,11002 475,39536 58,13631

476,12646 58,03694 475,60419 57,60273

476,27362 57,96261 475,74994 57,04852

476,4386 57,88699 475,90521 56,4784

476,5845 57,8101 476,07089 55,89564

476,75531 57,7319 476,25906 55,30346

476,95023 57,65239 476,427 54,70376

477,10437 57,57156 476,60687 54,09854

477,27972 57,48937 476,76654 53,48927

477,4458 57,4058 476,9415 52,87668

477,63348 57,32087 477,10596 52,26284

477,81531 57,23455 477,27011 51,64731

477,99783 57,14683 477,42419 51,03229

478,17621 57,05769 477,58694 50,41772

478,35876 56,96713 477,79797 49,80343

478,51965 56,87513 477,96667 49,19083

478,69345 56,78167 478,11948 48,57941

478,84897 56,68675 478,28906 47,97032

479,03275 56,59034 478,54642 47,3643

479,20496 56,49245 479,12646 46,75999

479,35214 56,39306 479,82776 46,15847

479,52737 56,29216 480,48047 45,56003

479,71265 56,18975 481,12378 44,96504

479,88245 56,08582 481,52274 44,37332

480,05017 55,98039 481,77426 43,78488

480,22348 55,8734 481,95731 43,19977

480,3739 55,76487 482,03699 42,61878

480,53833 55,65484 482,07202 42,04161

480,71567 55,54327 482,03748 41,46814

480,87958 55,43017 481,99316 40,89885

481,04813 55,31555 481,95844 40,33382

481,22806 55,1994 481,92737 39,77337

481,41684 55,08173 481,85492 39,21641

481,55942 54,96254 481,81839 38,66445

481,7435 54,84183 481,72845 38,11681

481,91815 54,71961 481,62762 37,57401

482,08813 54,5959 481,58899 37,03561

482,23657 54,47072 481,54236 36,50134

482,40457 54,34409 481,49881 35,97205

482,5853 54,21604 481,45477 35,44808

482,74545 54,08655 481,4346 34,92918

482,91116 53,95567 481,41815 34,41539

483,09207 53,82341 481,41425 33,90749

483,25116 53,6898 481,43719 33,40505

483,42407 53,55484 481,4473 32,91282

483,5853 53,41856 481,52435 32,45177

483,77417 53,281 481,57944 32,03428

483,93726 53,1422 481,6741 31,66765

484,11414 53,0022 481,76328 31,35886

484,27087 52,86104 481,87708 31,09743

484,45313 52,71872 481,99695 30,8775

484,63602 52,57529 482,15765 30,69222

484,77435 52,4308 482,29291 30,53566

484,95477 52,28528 482,50092 30,40236

485,11526 52,13878 482,71591 30,28848

485,26596 51,99134 482,88583 30,19042

485,45087 51,84303 483,12186 30,10521

485,62357 51,69386 483,35254 30,03045

485,81226 51,5439 483,56567 29,96514

485,94598 51,39321 483,85931 29,90704

486,12534 51,24183 484,0802 29,8557

486,3013 51,08979 484,31235 29,80925

486,49344 50,93715 484,58328 29,76757

486,65765 50,78396 484,83508 29,72936

486,82083 50,63029 485,09363 29,6938

487,01941 50,47618 485,36688 29,6614

487,16263 50,32171 485,62973 29,63187

487,35645 50,16691 485,87152 29,60399

487,51395 50,01184 486,13553 29,57782

487,68158 49,85656 486,39233 29,55284

487,83499 49,7011 486,62485 29,53016

488,01761 49,54555 486,88245 29,50844

488,20081 49,38995 487,13901 29,48791

488,34818 49,23437 487,38831 29,46802

488,49692 49,07889 487,6217 29,44901

488,66449 48,92358 487,84619 29,43061

488,8204 48,76847 488,08447 29,41314

488,99695 48,61366 488,32944 29,39678

489,16962 48,45916 488,54202 29,38067

489,34937 48,30504 488,76434 29,36515

489,50809 48,15137 488,974 29,35019

489,65887 47,9982 489,15311 29,33614

489,84329 47,84557 489,35507 29,32183

489,99158 47,69356 489,52557 29,3087

490,14014 47,5422 489,71008 29,29533

490,29272 47,39157 489,87378 29,28285

490,47437 47,24173 490,04453 29,27095

490,64789 47,09273 490,19321 29,25934

490,7955 46,9446 490,35364 29,24864

490,98245 46,79738 490,53583 29,23779

491,15381 46,65111 490,70337 29,22714

491,33276 46,50583 490,86307 29,21728

491,4935 46,3616 490,99539 29,20766

491,65286 46,21845 491,14853 29,19799

491,82172 46,07642 491,30292 29,18874

492,00467 45,93558 491,43866 29,17962

492,14462 45,79596 491,56824 29,17087

492,31293 45,65756 491,73447 29,16314

492,49179 45,52041 491,8743 29,15527

492,66879 45,38456 492,01453 29,14796

492,86194 45,25002 492,18924 29,14104

493,0181 45,11684 492,32104 29,13493

493,17737 44,98503 492,44812 29,12822

493,35272 44,85462 492,59027 29,12179

493,52844 44,72563 492,74008 29,11645

493,69617 44,59807 492,86288 29,11128

493,85223 44,47196 492,99463 29,10606

494,02588 44,34731 493,13315 29,1014

494,21313 44,22413 493,28232 29,09751

494,379 44,10242 493,4136 29,09332

494,5365 43,9822 493,56796 29,08908

494,70428 43,86348 493,71948 29,08493

494,88983 43,74627 493,88358 29,08102

495,05838 43,63058 494,022 29,07742

495,2356 43,5164 494,18817 29,07414

495,40375 43,40374 494,34183 29,07173

495,56357 43,2926 494,5032 29,06945

495,74686 43,18295 494,67725 29,06651

495,89978 43,07483 494,84784 29,06447

496,0509 42,96825 495,00427 29,06255

496,22275 42,86317 495,16003 29,06093

496,39197 42,75962 495,32141 29,05972

496,564 42,65757 495,51053 29,05875

496,75092 42,55701 495,6973 29,05764

496,92401 42,45791 495,8699 29,0566

497,06772 42,36027 496,05371 29,05617

497,26416 42,26407 496,21307 29,05607

497,42285 42,16929 496,38022 29,05614

497,58484 42,07592 496,52234 29,0555

497,73349 41,98395 496,68979 29,05547

497,91748 41,89333 496,87485 29,05578

498,09619 41,8041 497,04956 29,0566

498,2594 41,71624 497,24319 29,0571

498,43054 41,62971 497,41174 29,0579

498,62219 41,54451 497,56995 29,05932

498,79333 41,46061 497,71829 29,06048

498,95657 41,37799 497,87329 29,06088

499,13306 41,29662 498,07944 29,06131

499,2937 41,21648 498,25482 29,06199

499,45477 41,13758 498,45218 29,06242

499,63818 41,05988 498,60242 29,06401

499,80176 40,98339 498,77475 29,06538

499,9668 40,90808 498,9729 29,0665

500,13049 40,83393 499,13284 29,06794

500,31332 40,76091 499,28381 29,06952

500,47437 40,68899 499,46414 29,071

500,64056 40,61816 499,61404 29,07257

500,8103 40,5484 499,82156 29,07437

500,96048 40,4797 499,97668 29,0753

501,13431 40,41204 500,14697 29,07673

501,29379 40,3454 500,32947 29,07814

501,44397 40,27978 500,50766 29,07993

501,6098 40,2151 500,66809 29,08109

501,79755 40,15139 500,827 29,08348

501,96521 40,08861 501,00458 29,08584

502,1391 40,02674 501,18457 29,08737

502,29736 39,96576 501,37411 29,08837

502,45825 39,90565 501,53348 29,08969

502,61157 39,84641 501,71155 29,09114

502,80789 39,78803 501,8765 29,09281

502,96756 39,73048 502,06406 29,09444

503,12674 39,67376 502,19998 29,09666

503,31488 39,61782 502,3606 29,0985

503,47656 39,56266 502,54749 29,10123

503,66367 39,50827 502,71649 29,103

503,83447 39,45464 502,85803 29,1053

504,00443 39,40175 503,03796 29,10705

504,15765 39,34959 503,23779 29,10969

504,35754 39,29815 503,41336 29,11167

504,48563 39,24739 503,56946 29,11408

504,65457 39,1973 503,72778 29,11678

504,84329 39,14789 503,84998 29,11881

505,022 39,09913 504,0267 29,12077

505,18408 39,05101 504,1748 29,12284

505,34262 39,00353 504,32074 29,12526

505,51288 38,95668 504,49515 29,12798

505,71332 38,91044 504,65002 29,13

505,87192 38,86478 504,8114 29,13206

506,01947 38,81971 504,987 29,13457

506,19135 38,7752 505,10413 29,13698

506,37143 38,73126 505,26663 29,13966

506,53345 38,68789 505,4184 29,14167

506,72449 38,64505 505,57025 29,14337

506,88025 38,60271 505,74155 29,1454

507,03793 38,56088 505,90146 29,14751

507,18823 38,51954 506,05322 29,14909

507,37265 38,4787 506,21069 29,15108

507,51663 38,43835 506,39438 29,15314

507,69531 38,39845 506,56848 29,15548

507,86584 38,35901 506,70242 29,15797

508,05713 38,32002 506,87 29,16033

508,23303 38,28146 507,05286 29,16181

508,38794 38,24331 507,21225 29,16372

508,5687 38,20558 507,38217 29,16614

508,7478 38,16825 507,53647 29,16861

508,88916 38,13132 507,73862 29,1714

509,07211 38,09481 507,91434 29,17376

509,23978 38,05869 508,04056 29,17622

509,42947 38,02295 508,21008 29,17904

509,5748 37,98756 508,38675 29,18195

509,73645 37,95253 508,543 29,18544

509,91431 37,91784 508,70462 29,18839

510,08215 37,88348 508,84509 29,1914

510,26614 37,84947 509,03217 29,19433

510,42554 37,81581 509,19632 29,19738

510,57129 37,78246 509,32733 29,20025

510,74564 37,74946 509,51514 29,20311

510,92825 37,71677 509,69843 29,20648

511,08499 37,6844 509,89365 29,20967

511,24619 37,65235 510,07114 29,2124

511,4158 37,62058 510,2265 29,21488

511,59851 37,58909 510,3938 29,21771

511,76556 37,5579 510,57352 29,22019

511,90897 37,52701 510,73022 29,22288

512,07343 37,4964 510,93256 29,22527

512,21503 37,46606 511,09149 29,228

512,38721 37,43599 511,2562 29,23096

512,57861 37,40616 511,42166 29,23398

512,76361 37,37658 511,61728 29,23591

512,90991 37,34725 511,80981 29,23871

513,08191 37,31817 511,97137 29,24161

513,26129 37,28931 512,13916 29,24362

513,43195 37,26067 512,2887 29,2466

513,59528 37,23225 512,45056 29,24927

513,75952 37,20403 512,62531 29,25192

513,9599 37,17601 512,7757 29,25397

514,14722 37,14819 512,91638 29,25627

514,30212 37,12056 513,08521 29,2584

514,46277 37,09314 513,25574 29,26116

514,63513 37,06591 513,43719 29,2637

514,80298 37,03888 513,60815 29,26643

515,008 37,01203 513,77887 29,26888

515,18506 36,98537 513,94873 29,27156

515,35748 36,95886 514,10187 29,27465

515,5144 36,93256 514,26721 29,27751

515,68066 36,90642 514,44739 29,27997

515,85352 36,88046 514,58105 29,28229

516,01013 36,85466 514,77545 29,28503

516,17932 36,82901 514,93164 29,28778

516,35474 36,80352 515,09937 29,29095

516,54987 36,77819 515,26019 29,29399

516,74756 36,753 515,44495 29,29727

516,94354 36,72796 515,60791 29,30068

517,09082 36,70308 515,75299 29,30363

517,27618 36,67833 515,95361 29,30656

517,44287 36,65373 516,11743 29,30968

517,63904 36,62929 516,30328 29,31235

517,83356 36,60497 516,46484 29,31546

517,97626 36,58078 516,62524 29,31829

518,16534 36,5567 516,78082 29,32049

518,28473 36,53276 516,95215 29,32306

518,47485 36,50895 517,12915 29,32589

518,65894 36,48529 517,27185 29,32842

518,80688 36,46175 517,43677 29,331

518,99493 36,43833 517,60522 29,33376

519,17365 36,41501 517,78766 29,33611

519,33276 36,39182 517,96985 29,33848

519,51752 36,36873 518,14032 29,34137

519,65796 36,34575 518,29224 29,34408

519,82574 36,32287 518,45435 29,34698

519,97711 36,3001 518,61182 29,34993

520,12659 36,27746 518,75818 29,35333

520,27295 36,25494 518,91193 29,35626

520,44336 36,23252 519,08362 29,35896

520,59314 36,21018 519,25104 29,36188

520,73932 36,18795 519,41968 29,36483

520,91064 36,16583 519,59375 29,36815

521,06403 36,1438 519,76807 29,37134

521,22717 36,12187 519,95471 29,37388

521,40137 36,10004 520,0932 29,37718

521,55884 36,0783 520,24457 29,38027

521,73114 36,05666 520,40784 29,38309

521,90112 36,03514 520,55963 29,3862

522,03638 36,0137 520,70551 29,38895

522,20496 35,99238 520,88818 29,39164

522,39954 35,97116 521,0752 29,39452

522,54797 35,95005 521,20142 29,39795

522,73877 35,92904 521,34265 29,40128

522,90717 35,90814 521,5274 29,40459

523,07452 35,88732 521,70245 29,40721

523,23151 35,86662 521,88641 29,40986

523,3941 35,84601 522,07751 29,41287

523,53857 35,82549 522,2464 29,41538

523,71143 35,80506 522,3902 29,41858

523,92389 35,78477 522,56018 29,42103

524,10773 35,76455 522,72961 29,42363

524,25262 35,74442 522,90887 29,42658

524,4458 35,72439 523,04584 29,42948

524,60767 35,70444 523,21338 29,43214

524,77625 35,68458 523,3775 29,43521

524,94763 35,66484 523,5625 29,43756

525,12323 35,64519 523,71997 29,43998

525,26428 35,62564 523,88251 29,44267

525,43933 35,60619 524,05371 29,44535

525,58423 35,58684 524,23706 29,44816

525,75568 35,56759 524,45044 29,45129

525,91656 35,54844 524,60284 29,45447

526,1106 35,52937 524,79175 29,45762

526,25824 35,5104 525,00793 29,46102

526,42621 35,49155 525,19312 29,46424

526,55841 35,47281 525,34766 29,46751

526,7337 35,45418 525,51068 29,4711

526,92377 35,43564 525,69641 29,47386

527,0755 35,41719 525,86108 29,47716

527,23419 35,39884 526,02832 29,47994

527,41663 35,38059 526,20654 29,48269

527,56555 35,36245 526,38422 29,48529

527,74384 35,34442 526,55676 29,48855

527,89807 35,3265 526,72626 29,49156

528,08087 35,3087 526,8877 29,49423

528,24756 35,29101 527,04803 29,49727

528,40417 35,27341 527,22089 29,50012

528,56207 35,25592 527,38092 29,50258

528,72021 35,23852 527,56567 29,50571

528,90808 35,22122 527,73285 29,50905

529,05762 35,20404 527,89679 29,51226

529,21381 35,18696 528,05103 29,51473

529,41675 35,16997 528,24323 29,51758

529,58386 35,15309 528,40796 29,51968

529,76642 35,13633 528,6106 29,52274

529,93042 35,11967 528,78644 29,52551

530,09058 35,10313 528,9353 29,52829

530,2525 35,0867 529,0874 29,53126

530,43079 35,07036 529,2558 29,53486

530,60852 35,05412 529,43878 29,53817

530,79877 35,03799 529,59204 29,54114

530,97998 35,02196 529,76398 29,5444

531,17084 35,00604 529,93158 29,54799

531,34155 34,99022 530,09521 29,551

531,50653 34,9745 530,22083 29,55435

531,69562 34,9589 530,38568 29,55789

531,88788 34,94339 530,57886 29,56159

532,07275 34,928 530,75067 29,56474

532,22333 34,91273 530,91187 29,56818

532,42688 34,89756 531,08887 29,57094

532,60254 34,88247 531,28711 29,57388

532,76953 34,8675 531,45551 29,5771

532,94727 34,85265 531,63867 29,58024

533,10498 34,83791 531,78583 29,58338

533,27637 34,82328 531,95581 29,58678

533,43365 34,80876 532,15686 29,58998

533,59375 34,79435 532,30707 29,59314

533,78705 34,78006 532,48608 29,59651

533,94324 34,7659 532,63965 29,59936

534,125 34,75185 532,82813 29,6028

534,28027 34,7379 532,97552 29,60656

534,43701 34,72407 533,13385 29,6092

534,61914 34,71035 533,31311 29,61199

534,76111 34,69676 533,48383 29,61508

534,9317 34,68326 533,64496 29,61757

535,078 34,66987 533,84497 29,62077

535,25317 34,65658 534,00519 29,6235

535,39667 34,64339 534,1557 29,62642

535,56964 34,63033 534,3186 29,62906

535,73627 34,61738 534,5036 29,63173

535,89459 34,60454 534,66113 29,63468

536,04803 34,59181 534,78943 29,6376

536,19983 34,5792 534,95599 29,64071

536,39276 34,5667 535,11652 29,64373

536,54425 34,5543 535,28412 29,64638

536,71411 34,542 535,44855 29,64976

536,87994 34,5298 535,61945 29,65273

537,03577 34,51772 535,77808 29,65585

537,21686 34,50573 535,9292 29,65877

537,37329 34,49384 536,07056 29,66166

537,54102 34,48206 536,23718 29,66473

537,6972 34,47037 536,41339 29,66756

537,83911 34,45881 536,59894 29,67081

538,01904 34,44733 536,77368 29,67391

538,18896 34,43599 536,94922 29,67733

538,36554 34,42472 537,0871 29,68064

538,54834 34,41359 537,27509 29,6837

538,69452 34,40255 537,43042 29,68691

538,85883 34,39159 537,5705 29,68952

539,0166 34,38075 537,75684 29,69211

539,20026 34,37003 537,91528 29,69512

539,36304 34,35943 538,10175 29,69855

539,52069 34,34892 538,27368 29,70167

539,67517 34,33851 538,42865 29,70421

539,83862 34,32821 538,61542 29,70734

539,98047 34,31803 538,77997 29,71063

540,17731 34,30794 538,93616 29,71387

540,36133 34,29797 539,10651 29,71654

540,50439 34,28807 539,28491 29,71947

540,64777 34,27828 539,42596 29,72317

540,82788 34,26861 539,6156 29,72682

540,96741 34,25906 539,76447 29,73031

541,15924 34,2496 539,94385 29,73371

541,31787 34,24025 540,11517 29,73661

541,47931 34,23102 540,2677 29,73963

541,6524 34,22189 540,45599 29,74275

541,84155 34,21286 540,64008 29,74565

542,01624 34,20393 540,77618 29,74898

542,19238 34,1951 540,95947 29,75185

542,3584 34,18635 541,11292 29,75519

542,50946 34,1777 541,30054 29,75802

542,68286 34,16914 541,42902 29,76031

542,85785 34,16065 541,59058 29,76363

543,03143 34,15224 541,75171 29,76677

543,23785 34,14391 541,94 29,76942

543,40564 34,13569 542,10834 29,77194

543,55554 34,12756 542,26294 29,77465

543,72693 34,11952 542,37805 29,77795

543,9234 34,11159 542,55414 29,78073

544,0719 34,10373 542,7207 29,78341

544,25708 34,09596 542,85791 29,7871

544,41431 34,08828 543,01483 29,79013

544,57336 34,08069 543,17572 29,79331

544,73303 34,07319 543,33331 29,79633

544,89911 34,06578 543,45129 29,79881

545,05933 34,05845 543,60345 29,80194

545,21942 34,0512 543,78137 29,80475

545,41473 34,04404 543,96057 29,80767

545,565 34,03698 544,1261 29,81047

545,73901 34,03002 544,27417 29,81359

545,91315 34,02318 544,42993 29,8168

546,09119 34,01643 544,59668 29,81956

546,23395 34,00977 544,74567 29,82262

546,39966 34,00319 544,91559 29,82548

546,56372 33,99673 545,09204 29,8282

546,72107 33,99035 545,24634 29,83094

546,88641 33,98406 545,38452 29,83369

547,04974 33,97785 545,58649 29,83671

547,20477 33,97172 545,73114 29,83949

547,37585 33,96568 545,91125 29,84262

547,55518 33,95974 546,05017 29,84556

547,73578 33,95387 546,25366 29,84837

547,88397 33,94808 546,42719 29,8509

548,0332 33,94238 546,56873 29,85392

548,21307 33,93679 546,73578 29,85694

548,37061 33,93125 546,88501 29,86008

548,55835 33,92579 547,04834 29,86249

548,75122 33,92041 547,21246 29,86507

548,90698 33,91511 547,39203 29,86791

549,07159 33,9099 547,58258 29,87105

549,23511 33,90474 547,72662 29,87331

549,38409 33,89965 547,88696 29,87613

549,53992 33,89462 548,06128 29,87885

549,70215 33,88966 548,25555 29,88129

549,87677 33,88476 548,45123 29,88445

550,04663 33,87991 548,64801 29,88791

550,19232 33,87514 548,78864 29,89078

550,35468 33,87043 548,92926 29,89425

550,50726 33,8658 549,13525 29,89752

550,69287 33,86124 549,30414 29,90108

550,85748 33,85674 549,45544 29,90413

551,00024 33,85231 549,6402 29,90731

551,18597 33,84796 549,8241 29,91096

551,35614 33,84369 550,01398 29,91395

551,54919 33,8395 550,20245 29,91674

551,677 33,83538 550,37744 29,91994

551,84149 33,83132 550,55127 29,92316

552,03058 33,8273 550,74268 29,92606

552,18811 33,82334 550,92212 29,92904

552,36438 33,81943 551,10815 29,93249

552,56104 33,81557 551,24811 29,936

552,71277 33,81177 551,42017 29,93844

552,89441 33,80803 551,59222 29,94169

553,04016 33,80434 551,77045 29,94499

553,20447 33,8007 551,9104 29,9481

553,40601 33,79713 552,10358 29,9511

553,58569 33,79361 552,26355 29,95433

553,74347 33,79016 552,42993 29,95739

553,92432 33,78675 552,61841 29,96047

554,09216 33,78339 552,78143 29,96352

554,23645 33,78006 552,94238 29,96677

554,41791 33,77678 553,10046 29,9694

554,58167 33,77353 553,26392 29,97283

554,77771 33,77032 553,44257 29,97582

554,93896 33,76714 553,61224 29,97912

555,07904 33,76398 553,80475 29,98189

555,28192 33,76087 553,97925 29,98497

555,44977 33,75778 554,15485 29,98805

555,60883 33,75472 554,31561 29,99112

555,76154 33,7517 554,46112 29,99428

555,90479 33,74873 554,61475 29,99796

556,10376 33,74577 554,78461 30,00149

556,25171 33,74285 554,93829 30,00515

556,40887 33,73995 555,12561 30,00826

556,5976 33,73707 555,28198 30,01143

556,8009 33,73419 555,50037 30,01432

556,93781 33,73136 555,69702 30,01706

557,10956 33,72857 555,86572 30,0203

557,28809 33,7258 556,0119 30,02375

557,43042 33,72305 556,1756 30,02671

557,59302 33,72032 556,31641 30,02951

557,75177 33,71763 556,47876 30,03228

557,93549 33,71497 556,65967 30,0351

558,10864 33,71233 556,80762 30,03819

558,28156 33,7097 556,98529 30,04144

558,44897 33,70711 557,14783 30,0446

558,59473 33,70456 557,28412 30,0476

558,78467 33,70202 557,48499 30,05047

558,96631 33,6995 557,68329 30,05343

559,10571 33,69699 557,84131 30,0563

559,2774 33,6945 558,00989 30,05892

559,46362 33,69203 558,20972 30,06175

559,60547 33,68958 558,3454 30,0645

559,76019 33,68715 558,51636 30,067

559,91278 33,68475 558,703 30,0705

560,06537 33,68236 558,89313 30,0733

560,26843 33,67998 559,08118 30,07624

560,45325 33,67764 559,23572 30,07968

560,60419 33,67533 559,35913 30,083

560,76538 33,67305 559,52582 30,08618

560,93494 33,6708 559,70526 30,08857

561,10864 33,66858 559,84424 30,09128

561,29803 33,66637 559,99884 30,09464

561,43152 33,66418 560,17255 30,09755

561,61267 33,662 560,34442 30,10036

561,7569 33,65984 560,552 30,1034

561,93738 33,65768 560,70776 30,10677

562,09222 33,65554 560,88098 30,10967

562,26343 33,65339 561,04163 30,11265

562,42853 33,65125 561,20746 30,11533

562,61432 33,64912 561,37451 30,11826

562,74902 33,64699 561,52466 30,12144

562,92395 33,64487 561,70245 30,12426

563,10107 33,64277 561,86285 30,12687

563,27747 33,64068 562,00928 30,12951

563,44525 33,63862 562,17151 30,1322

563,60956 33,63659 562,34662 30,13584

563,77344 33,63456 562,52576 30,13867

563,92944 33,63256 562,69043 30,14164

564,07471 33,63058 562,85889 30,14455

564,25922 33,62863 563,04279 30,14758

564,44238 33,62668 563,18048 30,15021

564,61652 33,62476 563,38245 30,1529

564,75745 33,62287 563,5473 30,15553

564,92969 33,621 563,74249 30,15909

565,10474 33,61916 563,91107 30,16213

565,29022 33,61736 564,05225 30,1655

565,48798 33,61556 564,21161 30,1685

565,65405 33,61377 564,38055 30,17155

565,80951 33,61199 564,55682 30,1743

565,99591 33,61022 564,73413 30,17726

566,17841 33,60848 564,91003 30,1806

566,33978 33,60675 565,07666 30,18393

566,50909 33,60504 565,23596 30,18699

566,677 33,60333 565,40302 30,19017

566,86829 33,60166 565,58179 30,19303

567,03467 33,6 565,74902 30,19625

567,17822 33,59835 565,92383 30,19868

567,37769 33,59672 566,09747 30,20193

567,56897 33,5951 566,25525 30,20452

567,70636 33,59348 566,41089 30,20721

567,84894 33,59187 566,55798 30,20992

568,0376 33,59027 566,72638 30,21315

568,1853 33,58867 566,91968 30,21628

568,35754 33,58707 567,07928 30,2199

568,50757 33,58548 567,24933 30,22238

568,69977 33,5839 567,44849 30,22534

568,85535 33,58233 567,60663 30,22811

568,99158 33,58077 567,76849 30,23079

569,18201 33,57919 567,92621 30,23393

569,3559 33,57762 568,1217 30,23703

569,5144 33,57605 568,26648 30,24059

569,66119 33,5745 568,39948 30,24392

569,87598 33,57297 568,63135 30,24672

570,02545 33,57143 568,80798 30,24996

570,18713 33,56991 568,9726 30,25355

570,35199 33,5684 569,11914 30,25699

570,49506 33,56689 569,2998 30,26068

570,64856 33,56539 569,44873 30,26391

570,83612 33,56392 569,62183 30,26761

571,02527 33,56246 569,7785 30,2709

571,18573 33,56103 569,94287 30,27434

571,34198 33,55961 570,12451 30,27736

571,52289 33,55821 570,32556 30,28116

571,68384 33,55681 570,49969 30,2843

571,84717 33,55542 570,67188 30,2876

571,9873 33,55404 570,80377 30,29108

572,1825 33,55264 570,95844 30,29423

572,32904 33,55126 571,09894 30,29746

572,48364 33,54986 571,26245 30,30077

572,63849 33,54847 571,44116 30,30359

572,83765 33,54708 571,61084 30,30674

572,98792 33,54568 571,8045 30,31001

573,16333 33,54427 571,99927 30,31384

573,33252 33,54283 572,1925 30,31747

573,48651 33,54139 572,34662 30,32027

573,6543 33,53993 572,492 30,32343

573,81494 33,53848 572,66943 30,32604

573,98077 33,53703 572,78113 30,3292

574,16663 33,53559 572,95441 30,33205

574,33722 33,53419 573,12195 30,33481

574,47186 33,53277 573,30017 30,33819

574,64032 33,53135 573,492 30,34077

574,83594 33,52993 573,66516 30,34427

574,98413 33,52852 573,86029 30,34783

575,14673 33,52713 574,02301 30,35119

575,31372 33,52573 574,20624 30,35488

575,45886 33,52432 574,34351 30,35824

575,65833 33,52291 574,5144 30,36191

575,78662 33,52149 574,66675 30,3654

575,966 33,52008 574,81805 30,36899

576,12781 33,51869 574,97473 30,37265

576,31134 33,5173 575,14771 30,37639

576,46942 33,51592 575,34998 30,37971

576,62195 33,51456 575,53723 30,38308

576,77655 33,51321 575,69965 30,38588

576,96057 33,51186 575,87457 30,38946

577,14453 33,51051 576,03662 30,39296

577,28418 33,50916 576,21332 30,39666

577,45862 33,5078 576,38818 30,40016

577,62036 33,50645 576,55341 30,40292

577,79761 33,50511 576,72101 30,40628

577,98462 33,50377 576,89795 30,40911

578,13879 33,5024 577,06714 30,4129

578,31616 33,50102 577,24316 30,41627

578,47217 33,49966 577,40387 30,41936

578,65857 33,49831 577,55579 30,42213

578,81744 33,49695 577,75269 30,42512

578,98511 33,49559 577,93622 30,42811

579,16333 33,49424 578,09332 30,43155

579,30365 33,49287 578,23346 30,4344

579,47205 33,49149 578,37476 30,43763

579,67395 33,49009 578,5614 30,44092

579,83856 33,48867 578,73444 30,44398

579,99792 33,48723 578,92853 30,44711

580,1637 33,48579 579,09509 30,45076

580,3266 33,48433 579,27655 30,45421

580,49634 33,48287 579,44336 30,45802

580,65216 33,4814 579,64221 30,46109

580,81213 33,47992 579,797 30,46462

580,97168 33,47845 579,99438 30,4681

581,15942 33,47696 580,14307 30,472

581,3454 33,47544 580,3075 30,47573

581,50983 33,47394 580,46704 30,47913

581,65112 33,47244 580,6499 30,48214

581,82672 33,47094 580,80762 30,48527

581,9707 33,46943 580,99164 30,48889

582,15674 33,46791 581,18408 30,49208

582,33154 33,46635 581,349 30,49606

582,48901 33,46477 581,51367 30,49981

582,66663 33,46318 581,6792 30,50405

582,81335 33,46154 581,86389 30,50825

582,98071 33,4599 582,06165 30,51188

583,16205 33,45823 582,21204 30,51604

583,33325 33,45653 582,35925 30,51937

583,5163 33,45481 582,52728 30,52226

583,69507 33,45303 582,65942 30,52524

583,8822 33,45122 582,84259 30,52826

584,05359 33,4494 583,0296 30,53178

584,21356 33,44757 583,18408 30,5356

584,35394 33,44572 583,35669 30,53894

584,53143 33,44385 583,52814 30,54227

584,69574 33,44193 583,68878 30,54612

584,875 33,43999 583,86505 30,54929

585,02197 33,438 584,00128 30,5532

585,20654 33,43597 584,18671 30,55705

585,40491 33,43392 584,33936 30,56087

585,51465 33,43184 584,48352 30,56411

585,7229 33,42973 584,65173 30,56733

585,91241 33,42759 584,84381 30,57069

586,05688 33,42542 584,98169 30,57419

586,21405 33,42322 585,14545 30,57806

586,40967 33,42101 585,30957 30,58107

586,56476 33,41875 585,45898 30,58539

586,71118 33,4165 585,64368 30,58871

586,89856 33,41422 585,78601 30,59221

587,07678 33,41191 585,96265 30,59563

587,25531 33,40957 586,15948 30,5992

587,43042 33,40722 586,31177 30,60234

587,58862 33,40484 586,48608 30,60564

587,74939 33,40243 586,66034 30,6089

587,93945 33,39997 586,85352 30,61242

588,10864 33,39751 587,0274 30,6159

588,27466 33,39503 587,20538 30,61929

588,43835 33,39255 587,38147 30,62256

588,59094 33,39005 587,53076 30,62597

588,75165 33,38754 587,70404 30,62913

588,91492 33,38502 587,86047 30,63281

589,09985 33,38246 588,04071 30,63577

589,26416 33,37989 588,21808 30,63953

589,43445 33,3773 588,33752 30,64286

589,58374 33,37469 588,5072 30,64612

589,76709 33,37208 588,69104 30,64924

589,94043 33,36948 588,85919 30,65257

590,11963 33,36685 589,05859 30,65562

590,29614 33,3642 589,18958 30,65874

590,46014 33,36152 589,37402 30,66223

590,62128 33,35885 589,52686 30,66596

590,79303 33,35617 589,70319 30,66936

590,95001 33,3535 589,87329 30,67234

591,11578 33,35082 590,0274 30,67507

591,29126 33,34811 590,21289 30,67787

591,48022 33,34541 590,37177 30,68062

591,64722 33,3427 590,51489 30,68356

591,81366 33,33999 590,66473 30,68657

591,94885 33,33729 590,86328 30,69015

592,12878 33,33459 591,03937 30,69353

592,29553 33,33193 591,22241 30,69684

592,47595 33,32928 591,38489 30,70025

592,64758 33,32663 591,54419 30,70302

592,83191 33,32397 591,71521 30,70645

592,98688 33,32132 591,8916 30,70961

593,13208 33,31868 592,03564 30,71307

593,31543 33,31604 592,2168 30,716

593,479 33,31342 592,39343 30,71956

593,65033 33,3108 592,55115 30,72227

593,80341 33,30821 592,72723 30,72533

593,99481 33,30563 592,89258 30,72851

594,15094 33,30304 593,08862 30,73194

594,32019 33,30047 593,20947 30,73566

594,47516 33,29791 593,38354 30,73853

594,6344 33,29536 593,56299 30,74162

594,77539 33,29283 593,71277 30,74477

594,94775 33,29029 593,8764 30,74789

595,11078 33,28778 594,06 30,7507

595,28223 33,28526 594,23853 30,75305

595,46881 33,28274 594,4151 30,75638

595,63275 33,28023 594,59143 30,75976

595,76807 33,27771 594,76288 30,76287

595,9632 33,27521 594,92621 30,76636

596,12427 33,2727 595,07507 30,76971

596,25934 33,27022 595,23065 30,77272

596,43073 33,26776 595,4032 30,77601

596,58466 33,2653 595,57941 30,77946

596,76819 33,26286 595,75262 30,78278

596,93182 33,26042 595,88574 30,78618

597,08832 33,25798 596,08923 30,78964

597,24756 33,25554 596,2572 30,79291

597,39136 33,2531 596,42493 30,79606

597,53955 33,25069 596,59326 30,79924

597,6731 33,24828 596,76074 30,80221

597,8645 33,24587 596,93372 30,80538

598,02466 33,24346 597,10699 30,80899

598,20074 33,24104 597,26556 30,81232

598,35748 33,23862 597,4187 30,81527

598,51935 33,2362 597,58917 30,81849

598,69244 33,2338 597,76563 30,82197

598,87366 33,23142 597,94604 30,8248

599,06641 33,22903 598,1413 30,82753

599,19971 33,22663 598,31775 30,83043

599,38483 33,22424 598,45941 30,83436

599,54785 33,22185 598,64575 30,83801

599,72015 33,21948 598,83551 30,84169

599,89948 33,21711 599,02142 30,84541

600,06659 33,21477 599,20093 30,84889

600,24255 33,21242 599,36078 30,85248

600,40363 33,21008 599,54089 30,85544

600,59143 33,20774 599,74084 30,85904

600,75848 33,2054 599,89502 30,86246

600,91956 33,20305 600,00311 30,86639

601,09058 33,2007 600,17682 30,87022

601,23889 33,19835 600,36542 30,87364

601,40405 33,19598 600,51221 30,8772

601,58545 33,19362 600,67554 30,88062

601,76221 33,19127 600,84265 30,88406

601,92755 33,18892 601,03265 30,88744

602,11011 33,18659 601,18695 30,89059

602,27002 33,18425 601,30768 30,89379

602,4433 33,1819 601,4765 30,89722

602,62665 33,17956 601,64594 30,90063

602,80188 33,17723 601,8147 30,90405

602,93994 33,17491 601,97644 30,90768

603,11914 33,17258 602,14099 30,91097

603,3158 33,17024 602,32056 30,91436

603,47266 33,1679 602,48041 30,91833

603,62518 33,16556 602,62939 30,92133

603,80804 33,16321 602,78955 30,92506

603,9613 33,16087 602,98114 30,92858

604,10278 33,15853 603,11865 30,93183

604,27716 33,15618 603,29321 30,93575

604,44507 33,15382 603,47607 30,93943

604,60254 33,15147 603,65796 30,94312

604,77893 33,14912 603,82831 30,94702

604,93286 33,14676 604,00757 30,95068

605,09644 33,1444 604,15588 30,95401

605,25818 33,14203 604,31573 30,95776

605,39966 33,13968 604,49103 30,96155

605,55615 33,13733 604,6651 30,96499

605,73584 33,13499 604,81763 30,96864

605,88116 33,13264 604,99524 30,97221

606,04144 33,13031 605,14624 30,97597

606,18115 33,12797 605,29541 30,97974

606,34607 33,12565 605,48053 30,98365

606,51038 33,12334 605,64502 30,98719

606,71167 33,12103 605,82153 30,99086

606,85962 33,11872 605,94855 30,99475

607,00684 33,11642 606,12708 30,99828

607,18988 33,11412 606,27222 31,00133

607,36115 33,11181 606,46344 31,00494

607,53326 33,10952 606,61646 31,00866

607,69891 33,10725 606,8147 31,01229

607,85913 33,10499 607,00781 31,01567

608,02179 33,10275 607,16229 31,0193

608,19775 33,10052 607,33881 31,02261

608,34808 33,09829 607,49731 31,02545

608,52393 33,09605 607,66162 31,02925

608,70251 33,0938 607,81445 31,03256

608,88983 33,09156 607,99976 31,03614

609,07111 33,08932 608,16913 31,03946

609,24249 33,08709 608,32819 31,04299

609,39557 33,08487 608,48718 31,04594

609,54791 33,08266 608,65204 31,04928

609,73309 33,08048 608,81982 31,05221

609,90918 33,07833 608,99487 31,05519

610,03394 33,07617 609,17017 31,05839

610,21198 33,07401 609,31824 31,06159

610,40863 33,07185 609,51379 31,06536

610,55469 33,06967 609,68701 31,06897

610,70612 33,0675 609,84479 31,07265

610,88654 33,06535 610,00555 31,07672

611,0791 33,0632 610,19397 31,08038

611,21814 33,06104 610,34406 31,08402

611,39484 33,05889 610,51904 31,08793

611,57214 33,05676 610,68671 31,09175

611,7453 33,05465 610,85675 31,09512

611,89886 33,05256 611,01288 31,09849

612,05609 33,05049 611,17139 31,10288

612,23395 33,04843 611,31622 31,1069

612,396 33,04639 611,49225 31,1105

612,5802 33,04439 611,68048 31,11449

612,73682 33,0424 611,8288 31,11783

612,90527 33,04044 612,00854 31,1211

613,08325 33,03849 612,20416 31,12429

613,26996 33,03656 612,3772 31,12755

613,45111 33,03464 612,55414 31,13083

613,57269 33,03275 612,68494 31,13444

613,76367 33,03086 612,85297 31,1375

613,94556 33,02899 613,07385 31,14107

614,11316 33,02713 613,24878 31,1441

614,27063 33,0253 613,46625 31,14738

614,42285 33,02347 613,61377 31,15063

614,58722 33,02167 613,77472 31,15407

614,73969 33,01988 613,922 31,15773

614,94043 33,01811 614,09058 31,16125

615,10352 33,01636 614,24756 31,16452

615,25201 33,01461 614,43286 31,16812

615,40381 33,01292 614,61743 31,17131

615,58984 33,01123 614,74438 31,17533

615,75751 33,00956 614,92529 31,17922

615,92725 33,00791 615,11542 31,18282

616,08087 33,00628 615,27075 31,18585

616,25836 33,00465 615,44946 31,19002

616,45721 33,00304 615,60321 31,19323

616,58386 33,00144 615,77216 31,19673

616,77069 32,99986 615,96973 31,19991

616,95868 32,99831 616,1311 31,20362

617,09662 32,99681 616,26709 31,20702

617,27026 32,99536 616,427 31,21057

617,44379 32,99394 616,58649 31,2141

617,60919 32,99254 616,72217 31,21809

617,78815 32,99118 616,89551 31,22175

617,94012 32,98985 617,04333 31,22524

618,10724 32,98854 617,20227 31,22888

618,3042 32,98723 617,37061 31,23257

618,46777 32,98594 617,52692 31,23663

618,61047 32,98468 617,69904 31,24041

618,78979 32,98343 617,87152 31,2443

618,95038 32,98223 618,01563 31,24741

619,13501 32,98107 618,20117 31,25134

619,29895 32,97992 618,35535 31,25531

619,4624 32,97879 618,50439 31,25815

619,61884 32,97768 618,66711 31,26162

619,81421 32,97662 618,86511 31,26462

619,97534 32,97556 619,0448 31,26755

620,16003 32,97452 619,19397 31,27058

620,32001 32,97349 619,32184 31,27424

620,48273 32,97246 619,46606 31,27774

620,65656 32,97146 619,65881 31,28079

620,836 32,97046 619,83228 31,28416

621,00909 32,96947 619,96649 31,28727

621,1947 32,96848 620,16693 31,28995

621,37006 32,96748 620,34869 31,29302

621,49866 32,9665 620,56506 31,29649

621,65088 32,96551 620,68707 31,29951

621,82935 32,96453 620,7984 31,30285

622,01489 32,96356 620,97839 31,30595

622,19031 32,96258 621,19159 31,30915

622,37793 32,96163 621,35303 31,31258

622,53021 32,96068 621,52386 31,31568

622,67987 32,95974 621,71338 31,31919

622,85339 32,95882 621,89996 31,32266

623,03088 32,9579 622,0509 31,32601

623,20557 32,95699 622,24048 31,32897

623,37921 32,95606 622,38287 31,33247

623,51392 32,95512 622,53174 31,33548

623,70581 32,95421 622,69824 31,33876

623,87177 32,95329 622,90997 31,34204

624,05402 32,95235 623,05713 31,3458

624,203 32,9514 623,24969 31,34913

624,36487 32,95045 623,39618 31,35239

624,51886 32,94949 623,573 31,35596

624,68958 32,94852 623,7132 31,35932

624,84808 32,94756 623,8869 31,36245

625,00098 32,94659 624,06018 31,36579

625,17163 32,9456 624,22461 31,36951

625,31934 32,9446 624,39862 31,37339

625,46002 32,94357 624,55273 31,37767

625,67303 32,94248 624,71899 31,38097

625,85425 32,94134 624,89819 31,38443

626,05103 32,94014 625,07764 31,38815

626,21661 32,93892 625,23981 31,39119

626,36206 32,93764 625,39825 31,39467

626,51642 32,93633 625,54889 31,39856

626,68304 32,93497 625,67725 31,4024

626,85089 32,93357 625,89197 31,40629

627,03461 32,93215 626,06885 31,40969

627,19409 32,9307 626,23773 31,41332

627,33899 32,92922 626,4306 31,41672

627,51807 32,92767 626,58392 31,42033

627,69495 32,92608 626,75861 31,42416

627,85724 32,92448 626,95056 31,42768

628,00623 32,92283 627,09344 31,43122

628,1983 32,92112 627,29321 31,435

628,36285 32,91935 627,44116 31,43891

628,51019 32,91754 627,59235 31,44266

628,69867 32,9157 627,75421 31,44685

628,87726 32,9138 627,91748 31,45042

629,0152 32,91185 628,09082 31,45396

629,17761 32,90983 628,23303 31,45771

629,35901 32,90775 628,44293 31,46155

629,51532 32,90562 628,60498 31,46574

629,67981 32,90345 628,80493 31,46986

629,83984 32,90123 629,02771 31,47387

630,01978 32,89897 629,28894 31,47797

630,17645 32,89667 629,59705 31,48207

630,33722 32,8943 629,90125 31,48628

630,52722 32,89188 630,17841 31,4907

630,66583 32,8894 630,45209 31,49498

630,83484 32,88685 630,69061 31,49931

630,97021 32,88423 630,97516 31,50335

631,14862 32,88153 631,25391 31,50703

631,31689 32,87875 631,53711 31,5111

631,47681 32,87589 631,78992 31,51537

631,6499 32,87295 632,06128 31,51961

631,81641 32,86995 632,30249 31,52284

631,96991 32,8669 632,54254 31,52721

632,15009 32,86377 632,76532 31,53124

632,29407 32,86058 632,98712 31,53485

632,48138 32,85733 633,21198 31,53873

632,65997 32,85402 633,42017 31,54196

632,80054 32,85065 633,6286 31,546

632,98279 32,84721 633,85345 31,5497

633,1394 32,8437 634,03503 31,55389

633,31995 32,84011 634,21484 31,55753

633,48566 32,83643 634,40204 31,56148

633,65289 32,83267 634,59576 31,56513

633,82391 32,82882 634,78314 31,56872

634,00519 32,82492 634,94873 31,57197

634,16797 32,82096 635,09973 31,57581

634,34656 32,81693 635,22931 31,57993

634,52777 32,81283 635,3916 31,58348

634,67755 32,80867 635,52881 31,58767

634,85883 32,80444 635,67847 31,5915

635,03369 32,80016 635,84338 31,59508

635,20154 32,79583 635,98181 31,59828

635,35754 32,79144 636,12964 31,60168

635,54352 32,78697 636,26166 31,60533

635,67017 32,78243 636,40765 31,60901

635,82471 32,77783 636,54688 31,61321

636,00983 32,7732 636,70874 31,61746

636,18555 32,7685 636,8421 31,62077

636,33447 32,76374 636,95154 31,62469

636,50153 32,75895 637,12927 31,62906

636,65424 32,75412 637,30255 31,6321

636,82251 32,74927 637,44867 31,63601

637,00598 32,74436 637,6062 31,63985

637,16699 32,73939 637,75421 31,64345

637,32385 32,73439 637,90405 31,64732

637,48181 32,72935 638,0647 31,65091

637,63678 32,72431 638,21783 31,65426

637,81287 32,71924 638,38196 31,65748

637,97485 32,71414 638,54498 31,6608

638,12012 32,709 638,7193 31,66417

638,27991 32,70382 638,86774 31,66773

638,4278 32,69861 639,07263 31,67097

638,60315 32,69337 639,22571 31,67484

638,77985 32,68812 639,38336 31,67817

638,93689 32,68286 639,53271 31,68131

639,10431 32,67758 639,6936 31,68463

639,28296 32,67227 639,86279 31,68807

639,43988 32,66695 640,00183 31,69156

639,57574 32,66164 640,15186 31,69477

639,71704 32,65634 640,35803 31,6984

639,88995 32,65105 640,54645 31,70196

640,04749 32,64575 640,70435 31,70517

640,24365 32,64046 640,89246 31,70833

640,40973 32,63516 641,06372 31,71157

640,5769 32,62985 641,20502 31,71526

640,73267 32,62455 641,35602 31,71885

640,93506 32,61924 641,5249 31,72157

641,10199 32,61394 641,7099 31,7252

641,24353 32,60864 641,802 31,72851

641,44092 32,60336 641,97925 31,73164

641,62384 32,59809 642,16528 31,73441

641,80597 32,59285 642,34906 31,73771

641,95984 32,58765 642,51508 31,74093

642,14453 32,58248 642,66968 31,7436

642,29901 32,57734 642,85242 31,74678

642,48499 32,57223 642,99054 31,75012

642,63208 32,56715 643,19458 31,75346

642,82971 32,56211 643,35913 31,75659

642,97638 32,55714 643,54913 31,76002

643,14294 32,55221 643,68634 31,76291

643,3324 32,54732 643,85132 31,76586

643,48206 32,54246 644,00439 31,76896

643,66797 32,53762 644,16803 31,772

643,82568 32,53282 644,31396 31,77487

644,00513 32,52806 644,49225 31,77815

644,16772 32,52336 644,66345 31,78116

644,34662 32,5187 644,84827 31,78353

644,47131 32,51406 644,9939 31,78587

644,66071 32,50949 645,15088 31,78875

644,83752 32,50497 645,34802 31,79121

645,01465 32,50048 645,48828 31,79372

645,17896 32,49601 645,67535 31,7972

645,34613 32,4916 645,84167 31,80015

645,5105 32,48723 645,995 31,80305

645,65857 32,48294 646,15625 31,80602

645,83038 32,4787 646,32867 31,8088

646,00403 32,47452 646,48895 31,81212

646,1427 32,47039 646,66547 31,8154

646,32666 32,46631 646,84192 31,81877

646,48175 32,4623 646,97418 31,82222

646,65454 32,45835 647,11481 31,82572

646,82318 32,45446 647,27899 31,82887

646,9845 32,45063 647,44458 31,83228

647,13013 32,44685 647,63165 31,83532

647,31812 32,44313 647,79401 31,83847

647,47021 32,43947 647,94531 31,84178

647,64899 32,43585 648,10712 31,84507

647,79645 32,4323 648,25415 31,84819

647,92816 32,42881 648,41992 31,85171

648,14557 32,4254 648,55475 31,85582

648,29321 32,42202 648,72778 31,85872

648,46198 32,4187 648,88971 31,86169

648,62183 32,41544 649,04486 31,86531

648,78418 32,41225 649,23737 31,8688

648,99927 32,40912 649,38177 31,87208

649,15161 32,40605 649,55151 31,87521

649,28937 32,40305 649,72339 31,87855

649,45538 32,40012 649,88751 31,88238

649,64136 32,39724 650,06659 31,88562

649,79364 32,39444 650,24817 31,88879

649,95624 32,39168 650,39594 31,89235

650,14673 32,38898 650,55859 31,8958

650,31274 32,38636 650,72241 31,89919

650,46191 32,38378 650,85779 31,90273

650,64124 32,38127 650,98901 31,90646

650,81366 32,37884 651,20538 31,91042

650,99036 32,37647 651,38745 31,91466

651,1709 32,37418 651,58978 31,91826

651,31836 32,37197 651,71216 31,92174

651,50586 32,36981 651,86719 31,92515

651,67017 32,36773 652,04932 31,92911

651,83301 32,36571 652,19818 31,93318

652,02197 32,36377 652,35376 31,93694

652,17596 32,36191 652,54724 31,94064

652,36639 32,36012 652,70123 31,94481

652,53375 32,35841 652,8844 31,9484

652,67285 32,35678 653,05908 31,95205

652,79675 32,35521 653,25818 31,95573

652,96753 32,3537 653,4411 31,95944

653,15497 32,35226 653,60284 31,96332

653,3111 32,35088 653,78992 31,9667

653,47211 32,34957 653,92682 31,97072

653,65192 32,34833 654,08716 31,97438

653,80756 32,34716 654,25232 31,97809

653,96796 32,34606 654,41187 31,98157

654,13843 32,34502 654,58539 31,98472

654,32031 32,34404 654,7486 31,98843

654,48511 32,34311 654,92041 31,99178

654,62292 32,34225 655,08374 31,99507

654,78461 32,34145 655,24072 31,99858

654,94885 32,34071 655,38953 32,00155

655,13861 32,34004 655,55988 32,00454

655,31738 32,33944 655,76373 32,00802

655,45007 32,3389 655,93311 32,01113

655,63654 32,33841 656,0459 32,01407

655,82477 32,33797 656,2229 32,0176

655,96979 32,33759 656,39783 32,02083

656,14783 32,3373 656,49744 32,02453

656,29022 32,33707 656,65186 32,0281

656,48956 32,33692 656,82617 32,03116

656,67151 32,33681 656,99054 32,03451

656,80487 32,33678 657,12524 32,03762

656,96558 32,33681 657,26318 32,04082

657,12866 32,33689 657,4599 32,04388

657,29742 32,33704 657,62891 32,0475

657,46558 32,33723 657,81293 32,05052

657,6394 32,33749 657,98047 32,0538

657,79266 32,3378 658,13245 32,05704

657,94769 32,33816 658,30176 32,06005

658,12775 32,33858 658,4519 32,06341

658,30017 32,33906 658,61896 32,06644

658,46588 32,33962 658,79248 32,06925

658,63989 32,34022 658,95581 32,07283

658,81946 32,34086 659,12402 32,07576

658,98187 32,34156 659,28815 32,0784

659,13623 32,34231 659,43994 32,08171

659,32922 32,34311 659,58447 32,08439

659,50598 32,34395 659,69952 32,08663

659,64941 32,34484 659,89722 32,08972

659,81616 32,34575 660,10077 32,09268

660,03766 32,34669 660,24585 32,09614

660,18188 32,34766 660,4082 32,09918

660,34918 32,34868 660,60278 32,10206

660,51599 32,34973 660,78571 32,10448

660,68408 32,35084 660,94818 32,10786

660,83026 32,352 661,09851 32,11105

661,00946 32,35319 661,27655 32,11411

661,18933 32,3544 661,48297 32,11712

661,3501 32,35565 661,61182 32,12058

661,4917 32,35695 661,78888 32,12349

661,67773 32,3583 661,94781 32,1263

661,85059 32,35967 662,10718 32,12988

662,02441 32,36109 662,32422 32,13245

662,1626 32,36255 662,50439 32,13543

662,31226 32,36404 662,66669 32,13859

662,48193 32,36557 662,81366 32,14152

662,64374 32,36711 662,9845 32,14504

662,82617 32,36867 663,1579 32,14824

663,01282 32,37025 663,32288 32,15137

663,20062 32,37186 663,4903 32,15485

663,36182 32,37349 663,65222 32,15832

663,51428 32,37514 663,82312 32,16186

663,64258 32,37681 663,98706 32,16563

663,83563 32,37849 664,16388 32,1688

664,02441 32,38019 664,3092 32,17192

664,19556 32,38193 664,43457 32,17565

664,36145 32,3837 664,5835 32,17847

664,52234 32,38549 664,74976 32,18164

664,68402 32,38726 664,92975 32,18416

664,84424 32,38907 665,04053 32,18752

665,00745 32,39089 665,23621 32,19088

665,17676 32,39274 665,43732 32,19437

665,35663 32,39461 665,61572 32,19853

665,5058 32,39649 665,76593 32,20275

665,6676 32,39838 665,91705 32,2066

665,84393 32,4003 666,11292 32,21002

666,01019 32,40224 666,24969 32,21381

666,18988 32,40419 666,4184 32,2171

666,34888 32,40618 666,6026 32,22111

666,52014 32,40818 666,77014 32,22548

666,65765 32,4102 666,89404 32,22952

666,81494 32,41224 667,05542 32,23363

666,99799 32,41426 667,27515 32,23774

667,18524 32,41633 667,45917 32,24181

667,34741 32,41842 667,61023 32,24582

667,50519 32,42052 667,79468 32,25006

667,66559 32,42263 667,95862 32,25338

667,81396 32,42475 668,10553 32,25691

668,01123 32,42689 668,271 32,26027

668,17883 32,42903 668,44977 32,26378

668,36664 32,4312 668,59314 32,2674

668,5202 32,43339 668,71381 32,27065

668,64642 32,4356 668,87018 32,27382

668,83789 32,43779 669,06207 32,27735

669,01135 32,44002 669,23975 32,28176

669,20001 32,44223 669,40991 32,28551

669,34406 32,44444 669,58795 32,28833

669,51379 32,44668 669,74567 32,29203

669,69385 32,44893 669,88135 32,29586

669,84521 32,45121 670,04535 32,2992

670,00641 32,45349 670,22345 32,30306

670,16699 32,45578 670,35254 32,30698

670,3299 32,45808 670,54358 32,3109

670,52405 32,46039 670,66956 32,31514

670,70313 32,46272 670,86108 32,31881

670,85034 32,46506 671,02905 32,32179

671,00934 32,4674 671,17542 32,32574

671,17468 32,46979 671,36639 32,32957

671,35614 32,4722 671,52991 32,33328

671,49176 32,47465 671,66846 32,33709

671,6637 32,47711 671,85645 32,34079

671,82678 32,47959 672,03223 32,34422

671,96881 32,48208 672,21436 32,34781

672,12573 32,48459 672,35486 32,35208

672,3147 32,48713 672,53333 32,3566

672,50153 32,48969 672,63513 32,36125

672,6637 32,49227 672,81976 32,36544

672,81897 32,49488 673,00049 32,3699

672,96912 32,49751 673,15533 32,37443

673,14746 32,50017 673,34131 32,37869

673,31519 32,50284 673,51929 32,38255

673,47211 32,50552 673,65912 32,38627

673,62372 32,5082 673,83167 32,38997

673,78381 32,51087 674,00391 32,39369

673,93976 32,51356 674,16241 32,39727

674,15497 32,51627 674,32104 32,40191

674,30499 32,51898 674,50891 32,40625

674,47577 32,5217 674,64709 32,41029

674,62952 32,52444 674,83167 32,41475

674,81592 32,52721 674,974 32,41879

675,00311 32,53001 675,12573 32,42227

675,12616 32,53281 675,30487 32,42613

675,29163 32,53562 675,45648 32,43007

675,48108 32,53845 675,62189 32,43507

675,63129 32,54129 675,7926 32,43918

675,77136 32,54414 675,94995 32,44356

675,94519 32,547 676,11475 32,44765

676,13324 32,54989 676,25378 32,4526

676,28705 32,55279 676,41437 32,45725

676,4306 32,5557 676,55396 32,4621

676,59894 32,55864 676,70825 32,46648

676,75616 32,5616 676,8938 32,47051

676,95453 32,56458 677,05841 32,47457

677,11792 32,56758 677,25116 32,47928

677,2749 32,57062 677,34814 32,48363

677,44635 32,57367 677,54248 32,48753

677,58197 32,57675 677,69769 32,49154

677,771 32,57984 677,83014 32,49607

677,96228 32,58294 678,00104 32,50008

678,13245 32,58605 678,17664 32,50374

678,29388 32,58915 678,33276 32,50782

678,45239 32,59226 678,48138 32,51143

678,63147 32,5954 678,64801 32,51521

678,78247 32,59856 678,85278 32,51895

678,9505 32,60174 679,02539 32,5228

679,1181 32,60491 679,19403 32,52671

679,28674 32,6081 679,34906 32,53094

679,46118 32,61129 679,51837 32,53506

679,63361 32,61446 679,67096 32,53919

679,78735 32,61764 679,82727 32,54327

679,97974 32,62081 680,01324 32,54702

680,13312 32,62399 680,19397 32,55127

680,31506 32,62718 680,36664 32,55479

680,51605 32,63037 680,52832 32,55852

680,70758 32,63357 680,69159 32,56176

680,84692 32,63677 680,85352 32,56504

680,99115 32,63998 681,04932 32,56854

681,15649 32,64318 681,1969 32,57163

681,33588 32,64637 681,34961 32,57565

681,48639 32,64958 681,54352 32,57886

681,67639 32,65278 681,71381 32,58294

681,83539 32,656 681,8764 32,58644

681,97473 32,65923 682,01093 32,59031

682,15479 32,6625 682,19971 32,59418

682,32794 32,6658 682,38165 32,59782

682,48022 32,66911 682,55322 32,6012

682,64954 32,67243 682,72827 32,6039

682,83167 32,67578 682,8678 32,60717

682,97424 32,67914 683,02783 32,61083

683,15796 32,68251 683,17792 32,61392

683,31281 32,68585 683,375 32,6172

683,47913 32,68919 683,55518 32,62049

683,63354 32,69255 683,69141 32,6237

683,78387 32,69592 683,84113 32,62719

683,94507 32,69931 684,01947 32,63

684,1311 32,70271 684,19348 32,63366

684,29761 32,7061 684,37268 32,63685

684,44531 32,70951 684,58575 32,64034

684,59143 32,71291 684,75122 32,64336

684,74524 32,71631 684,90613 32,64619

684,91425 32,71969 685,05566 32,64931

685,11121 32,72308 685,1991 32,65249

685,27527 32,72647 685,34149 32,65609

685,41571 32,72987 685,5058 32,65948

685,57764 32,73326 685,68188 32,66222

685,74969 32,73664 685,83466 32,66552

685,91809 32,74003 686,0083 32,66912

686,11121 32,74342 686,16656 32,67222

686,3288 32,74683 686,30829 32,67589

686,48254 32,75024 686,45642 32,67898

686,62805 32,75365 686,62854 32,6827

686,82336 32,75707 686,80011 32,6863

686,97687 32,76049 686,96216 32,69047

687,14185 32,7639 687,16797 32,69428

687,30804 32,7673 687,30859 32,69742

687,48334 32,77069 687,48102 32,70067

687,6543 32,77408 687,68512 32,70384

687,82874 32,77747 687,80658 32,70754

687,98083 32,78087 687,92499 32,71085

688,16272 32,78428 688,13 32,7141

688,34003 32,78769 688,26837 32,7178

688,50366 32,79111 688,45026 32,72146

688,68042 32,79453 688,6015 32,72425

688,83655 32,79794 688,76086 32,72778

688,97961 32,80136 688,96082 32,7314

689,14551 32,80479 689,13367 32,7345

689,34747 32,80824 689,31006 32,73759

689,50739 32,81168 689,4519 32,74016

689,63446 32,81512 689,62402 32,74353

689,77917 32,81858 689,76172 32,74623

689,95459 32,82204 689,94061 32,7493

690,14038 32,8255 690,13812 32,75195

690,32086 32,82895 690,28387 32,75496

690,48303 32,83239 690,46875 32,75849

690,65009 32,83584 690,60999 32,76108

690,80884 32,83927 690,78296 32,76394

690,96484 32,84269 690,92242 32,7672

691,13898 32,8461 691,07568 32,76986

691,29523 32,8495 691,20966 32,77285

691,47931 32,85288 691,37952 32,77585

691,63232 32,85625 691,53314 32,77892

691,80396 32,85963 691,68451 32,78182

691,9859 32,86301 691,83936 32,7851

692,13666 32,86638 692,01672 32,7885

692,30084 32,86976 692,19592 32,79159

692,47736 32,87312 692,35101 32,79451

692,65222 32,87648 692,46002 32,79676

692,80444 32,87985 692,66302 32,80034

692,95959 32,88322 692,84052 32,8037

693,14911 32,88659 693,03589 32,80677

693,32898 32,88998 693,19012 32,81034

693,49561 32,89338 693,36926 32,81318

693,65527 32,89679 693,52472 32,81646

693,80334 32,90017 693,67316 32,81987

693,97253 32,90355 693,83087 32,82372

694,1449 32,90694 694,03687 32,82718

694,31683 32,91033 694,24811 32,83043

694,48682 32,91372 694,39319 32,83331

694,66577 32,91711 694,54382 32,83638

694,81726 32,92049 694,70581 32,83939

694,98035 32,92388 694,85272 32,84269

695,12012 32,92727 695,02502 32,84587

695,2865 32,93067 695,17249 32,84854

695,45544 32,93405 695,34509 32,85182

695,61261 32,93744 695,55054 32,85512

695,8269 32,94084 695,70752 32,85857

695,94946 32,94427 695,87854 32,86166

696,10315 32,94769 696,03723 32,86546

696,27094 32,95112 696,17444 32,86871

696,44287 32,95454 696,33887 32,87282

696,61938 32,95797 696,51581 32,87635

696,79187 32,9614 696,71338 32,87949

696,93091 32,96482 696,85175 32,88321

697,11652 32,96826 697,02515 32,88644

697,27734 32,97173 697,198 32,89022

697,46039 32,97519 697,37476 32,89348

697,63055 32,97865 697,53027 32,89777

697,82275 32,98211 697,69305 32,90138

697,9668 32,98556 697,85858 32,90525

698,15472 32,98902 698,02344 32,9087

698,2937 32,99247 698,15717 32,91222

698,4787 32,99592 698,35669 32,91617

698,65753 32,99938 698,51819 32,92024

698,81866 33,00285 698,66125 32,92395

698,97046 33,00631 698,82037 32,92694

699,16699 33,00978 698,97522 32,93041

699,32227 33,01324 699,10315 32,93399

699,4859 33,01671 699,23657 32,93734

699,64325 33,0202 699,40344 32,94111

699,7948 33,0237 699,56787 32,9446

699,97498 33,02722 699,74066 32,94784

700,16772 33,03075 699,89661 32,95142

700,31598 33,0343 700,08997 32,95441

700,45331 33,03785 700,25 32,95763

700,60278 33,0414 700,39105 32,961

700,78668 33,04495 700,55591 32,96445

700,96796 33,04851 700,75452 32,96783

701,12292 33,05209 700,91553 32,97186

701,29865 33,05565 701,10645 32,97467

701,45508 33,05921 701,27362 32,97765

701,63446 33,06279 701,4563 32,98117

701,8114 33,06637 701,62622 32,98418

701,96497 33,06994 701,7569 32,98717

702,12524 33,07351 701,88617 32,9906

702,26025 33,0771 702,09106 32,99369

702,43359 33,08067 702,24384 32,99593

702,60266 33,08424 702,44263 32,99943

702,76105 33,08781 702,56494 33,00289

702,95166 33,09138 702,76349 33,00667

703,10529 33,09493 702,91602 33,01019

703,2652 33,09849 703,06311 33,01374

703,43811 33,10205 703,20105 33,01761

703,60278 33,1056 703,37671 33,02117

703,77905 33,10916 703,60114 33,02529

703,94214 33,11271 703,77209 33,02857

704,09961 33,11626 703,93561 33,03182

704,28058 33,11979 704,11688 33,03508

704,40753 33,12331 704,26654 33,03898

704,61127 33,12683 704,43494 33,04283

704,80127 33,13035 704,55762 33,04662

704,98993 33,13388 704,71777 33,05003

705,14453 33,13741 704,89087 33,05369

705,29248 33,14094 705,05176 33,05728

705,46417 33,14447 705,22687 33,06074

705,65503 33,14799 705,41937 33,06498

705,8429 33,15149 705,57819 33,06855

705,9939 33,15498 705,73151 33,0717

706,17944 33,15848 705,91687 33,07459

706,32227 33,16197 706,0647 33,07807

706,48541 33,16549 706,22388 33,08204

706,65857 33,169 706,40009 33,08578

706,83441 33,17253 706,51831 33,08915

707,00012 33,17605 706,6922 33,09274

707,16693 33,17955 706,84314 33,09573

707,33801 33,18307 707,01514 33,09906

707,51294 33,1866 707,16138 33,1029

707,69098 33,19013 707,36005 33,10665

707,81396 33,19368 707,51056 33,10993

707,97815 33,19722 707,69434 33,11372

708,13916 33,20076 707,82446 33,11761

708,32556 33,20431 708,00562 33,12139

708,49664 33,20785 708,17334 33,12486

708,66217 33,21137 708,32495 33,12848

708,84821 33,21487 708,45856 33,13259

708,99658 33,21837 708,63647 33,13578

709,15002 33,22188 708,78772 33,13929

709,33868 33,22538 708,96686 33,14311

709,51221 33,22887 709,14252 33,14646

709,67316 33,23233 709,29883 33,14983

709,84253 33,2358 709,48505 33,15421

710,01862 33,23926 709,64014 33,15771

710,18268 33,24271 709,841 33,16176

710,31018 33,24616 709,99579 33,16579

710,4668 33,2496 710,16962 33,16974

710,65063 33,25305 710,3078 33,17301

710,82715 33,25651 710,45923 33,17671

710,96417 33,26 710,61676 33,1807

711,14014 33,26351 710,76538 33,18396

711,29248 33,26703 710,95886 33,18753

711,46222 33,27055 711,10626 33,1909

711,62677 33,27409 711,2901 33,19424

711,78186 33,27763 711,45709 33,19762

711,93677 33,28117 711,62195 33,2009

712,10919 33,28471 711,7926 33,20469
[truncated: 72,106 more chars]
